# Supplementary material for: Comparing the accuracy of two diagnostic methods for detection of light Schistosoma haematobium infection in an elimination setting in Wolaita Zone, South Western Ethiopia
Source: PLoS One. 2022 Apr 29;17(4):e0267378. doi: 10.1371/journal.pone.0267378 (PMC9053789; doi:10.1371/journal.pone.0267378)
Supplement: S1 Appendix — (RTF) [file pone.0267378.s001.rtf]

S1 Appendix: Results of number of individuals detected by POC-CCA test in Wolaita Zone, Ethiopia.
Urine-CCA test result	Negative	Trace	1+	2+	3+	
	3475(79.4%)	315(7.2)	247(5.6%)	194(3.7%)	126(2.9%)	
