# Supplementary material for: Comparing the accuracy of two diagnostic methods for detection of light Schistosoma haematobium infection in an elimination setting in Wolaita Zone, South Western Ethiopia
Source: PLoS One. 2022 Apr 29;17(4):e0267378. doi: 10.1371/journal.pone.0267378 (PMC9053789; doi:10.1371/journal.pone.0267378)
Supplement: S2 Appendix — (DOCX) [file pone.0267378.s002.docx]

| sex | agecat | haemastix | sHaematobiu | POC_CCA | Time |
| --- | --- | --- | --- | --- | --- |
| Female | 1 to 4 | 0 | #NUM! | 0 | Baseline |
| Male | 22 to 35 | 0 | #NUM! | 0 | Baseline |
| Female | 22 to 35 | 0 | #NUM! | 0 | Baseline |
| Male | 35+ | 0 | #NUM! | 0 | Baseline |
| Male | 5 to 14 | 0 | #NUM! | 0 | Baseline |
| Female | 22 to 35 | 0 | #NUM! | 0 | Baseline |
| Male | 15 to 21 | 0 | #NUM! | 0 | Baseline |
| Male | 35+ | 0 | #NUM! | 0 | Baseline |
| Male | 35+ | 0 | #NUM! | 0 | Baseline |
| Male | 22 to 35 | 0 | #NUM! | 0 | Baseline |
| Female | 5 to 14 | 0 | #NUM! | 0 | Baseline |
| Male | 1 to 4 | 0 | #NUM! | 0 | Baseline |
| Female | 35+ | 0 | #NUM! | 0 | Baseline |
| Male | 5 to 14 | 0 | #NUM! | 0 | Baseline |
| Male | 5 to 14 | 0 | #NUM! | 0 | Baseline |
| Female | 22 to 35 | 0 | #NUM! | 0 | Baseline |
| Female | 15 to 21 | 0 | #NUM! | 0 | Baseline |
| Male | 22 to 35 | 0 | #NUM! | 0 | Baseline |
| Male | 5 to 14 | 0 | #NUM! | 0 | Baseline |
| Male | 35+ | 0 | #NUM! | 0 | Baseline |
| Female | 5 to 14 | 0 | #NUM! | 0 | Baseline |
| Male | 35+ | 0 | #NUM! | 0 | Baseline |
| Female | 35+ | 0 | #NUM! | 0 | Baseline |
| Female | 22 to 35 | 0 | #NUM! | 0 | Baseline |
| Male | 1 to 4 | 0 | #NUM! | 0 | Baseline |
| Female | 5 to 14 | 0 | #NUM! | 0 | Baseline |
| Female | 5 to 14 | 0 | #NUM! | 0 | Baseline |
| Female | 5 to 14 | 0 | #NUM! | 0 | Baseline |
| Male | 5 to 14 | 0 | #NUM! | 0 | Baseline |
| Female | 5 to 14 | 0 | #NUM! | 0 | Baseline |
| Female | 15 to 21 | 0 | #NUM! | 0 | Baseline |
| Male | 15 to 21 | 0 | #NUM! | 0 | Baseline |
| Female | 35+ | 0 | #NUM! | 0 | Baseline |
| Female | 35+ | 0 | #NUM! | 0 | Baseline |
| Male | 35+ | 0 | #NUM! | 0 | Baseline |
| Female | 22 to 35 | 0 | #NUM! | 0 | Baseline |
| Female | 22 to 35 | 0 | #NUM! | 0 | Baseline |
| Female | 35+ | 0 | #NUM! | 0 | Baseline |
| Female | 35+ | 0 | #NUM! | 0 | Baseline |
| Female | 35+ | 0 | #NUM! | 0 | Baseline |
| Male | 22 to 35 | 0 | #NUM! | 0 | Baseline |
| Female | 5 to 14 | 0 | #NUM! | 0 | Baseline |
| Female | 1 to 4 | 0 | #NUM! | 0 | Baseline |
| Female | 15 to 21 | 0 | #NUM! | 0 | Baseline |
| Female | 22 to 35 | 0 | #NUM! | 0 | Baseline |
| Female | 22 to 35 | 0 | #NUM! | 0 | Baseline |
| Female | 35+ | 0 | #NUM! | 0 | Baseline |
| Female | 35+ | 0 | #NUM! | 0 | Baseline |
| Male | 22 to 35 | 0 | #NUM! | 0 | Baseline |

| Female | 5 to 14 | 0 | #NUM! | 0 | Baseline |
| --- | --- | --- | --- | --- | --- |
| Male | 5 to 14 | 0 | #NUM! | 0 | Baseline |
| Male | 15 to 21 | 0 | #NUM! | 0 | Baseline |
| Male | 5 to 14 | 0 | #NUM! | 0 | Baseline |
| Male | 5 to 14 | 0 | #NUM! | 0 | Baseline |
| Male | 5 to 14 | 0 | #NUM! | 0 | Baseline |
| Male | 5 to 14 | 0 | #NUM! | 0 | Baseline |
| Male | 15 to 21 | 0 | #NUM! | 0 | Baseline |
| Male | 15 to 21 | 0 | #NUM! | 0 | Baseline |
| Male | 15 to 21 | 0 | #NUM! | 0 | Baseline |
| Female | 35+ | 0 | #NUM! | 0 | Baseline |
| Male | 35+ | 0 | #NUM! | 0 | Baseline |
| Female | 22 to 35 | 0 | #NUM! | 0 | Baseline |
| Female | 22 to 35 | 0 | #NUM! | 0 | Baseline |
| Female | 5 to 14 | 0 | #NUM! | 0 | Baseline |
| Female | 35+ | 0 | #NUM! | 0 | Baseline |
| Male | 1 to 4 | 0 | #NUM! | 0 | Baseline |
| Male | 15 to 21 | 0 | #NUM! | 0 | Baseline |
| Male | 35+ | 0 | #NUM! | 0 | Baseline |
| Female | 35+ | 0 | #NUM! | 0 | Baseline |
| Male | 22 to 35 | 0 | #NUM! | 0 | Baseline |
| Female | 35+ | 0 | #NUM! | 0 | Baseline |
| Male | 1 to 4 | 0 | #NUM! | 0 | Baseline |
| Female | 1 to 4 | 0 | #NUM! | 0 | Baseline |
| Male | 35+ | 0 | #NUM! | 0 | Baseline |
| Male | 35+ | 0 | #NUM! | 0 | Baseline |
| Male | 35+ | 0 | #NUM! | 0 | Baseline |
| Male | 22 to 35 | 0 | #NUM! | 0 | Baseline |
| Male | 15 to 21 | 0 | #NUM! | 0 | Baseline |
| Female | 15 to 21 | 0 | #NUM! | 0 | Baseline |
| Female | 22 to 35 | 0 | #NUM! | 0 | Baseline |
| Male | 1 to 4 |  | #NUM! | 0 | Baseline |
| Female | 1 to 4 |  | #NUM! | 0 | Baseline |
| Female | 22 to 35 | 0 | #NUM! | 0 | Baseline |
| Female | 5 to 14 | 0 | #NUM! | 0 | Baseline |
| Male | 1 to 4 | 0 | #NUM! | 0 | Baseline |
| Female | 22 to 35 | 0 | #NUM! | 0 | Baseline |
| Female | 35+ | 0 | #NUM! | 0 | Baseline |
| Male | 5 to 14 | 0 | #NUM! | 0 | Baseline |
| Female | 5 to 14 | 0 | #NUM! | 0 | Baseline |
| Female | 5 to 14 | 0 | #NUM! | 0 | Baseline |
| Female | 5 to 14 |  | #NUM! | 0 | Baseline |
| Female | 22 to 35 | 0 | #NUM! | 0 | Baseline |
| Female | 35+ |  | #NUM! | 0 | Baseline |
| Female | 35+ |  | #NUM! | 0 | Baseline |
| Male | 35+ |  | #NUM! | 0 | Baseline |
| Female | 1 to 4 |  | #NUM! | 0 | Baseline |
| Female | 1 to 4 |  | #NUM! | 0 | Baseline |
| Female | 22 to 35 |  | #NUM! | 0 | Baseline |
| Female | 22 to 35 |  | #NUM! | 0 | Baseline |

| Male | 15 to 21 | 0 | #NUM! | 0 | Baseline |
| --- | --- | --- | --- | --- | --- |
| Male | 15 to 21 | 0 | #NUM! | 0 | Baseline |
| Male | 15 to 21 | 0 | #NUM! | 0 | Baseline |
| Male | 15 to 21 | 0 | #NUM! | 0 | Baseline |
| Female | 5 to 14 | 0 | #NUM! | 0 | Baseline |
| Female | 15 to 21 | 0 | #NUM! | 0 | Baseline |
| Female | 15 to 21 | 0 | #NUM! | 0 | Baseline |
| Male | 15 to 21 | 0 | #NUM! | 0 | Baseline |
| Female | 5 to 14 | 0 | #NUM! | 0 | Baseline |
| Male | 5 to 14 | 0 | #NUM! | 0 | Baseline |
| Female | 15 to 21 | 0 | #NUM! | 0 | Baseline |
| Male | 5 to 14 | 0 | #NUM! | 0 | Baseline |
| Female | 15 to 21 | 0 | #NUM! | 0 | Baseline |
| Male | 5 to 14 | 0 | #NUM! | 0 | Baseline |
| Female | 35+ | 0 | #NUM! | 0 | Baseline |
| Male | 5 to 14 | 0 | #NUM! | 0 | Baseline |
| Female | 35+ | 0 | #NUM! | 0 | Baseline |
| Male | 1 to 4 | 0 | #NUM! | 0 | Baseline |
| Female | 15 to 21 | 0 | #NUM! | 0 | Baseline |
| Female | 22 to 35 | 0 | #NUM! | 0 | Baseline |
| Female | 22 to 35 | 0 | #NUM! | 0 | Baseline |
| Male | 5 to 14 | 0 | #NUM! | 0 | Baseline |
| Male | 35+ | 0 | #NUM! | 0 | Baseline |
| Male | 35+ | 0 | #NUM! | 0 | Baseline |
| Female | 22 to 35 | 0 | #NUM! | 0 | Baseline |
| Male | 35+ | 0 | #NUM! | 0 | Baseline |
| Female | 1 to 4 | 0 | #NUM! | 0 | Baseline |
| Female | 15 to 21 | 0 | #NUM! | 0 | Baseline |
| Female | 22 to 35 | 0 | #NUM! | 0 | Baseline |
| Male | 15 to 21 | 0 | #NUM! | 0 | Baseline |
| Female | 5 to 14 | 0 | #NUM! | 0 | Baseline |
| Male | 35+ | 0 | #NUM! | 0 | Baseline |
| Male | 22 to 35 | 0 | #NUM! | 0 | Baseline |
| Female | 15 to 21 | 0 | #NUM! | 0 | Baseline |
| Female | 15 to 21 | 0 | #NUM! | 0 | Baseline |
| Male | 5 to 14 | 0 | #NUM! | 0 | Baseline |
| Female | 15 to 21 | 0 | #NUM! | 0 | Baseline |
| Female | 5 to 14 | 0 | #NUM! | 0 | Baseline |
| Female | 22 to 35 | 0 | #NUM! | 0 | Baseline |
| Female | 22 to 35 | 0 | #NUM! | 0 | Baseline |
| Male | 15 to 21 | 0 | #NUM! | 0 | Baseline |
| Male | 22 to 35 | 0 | #NUM! | 0 | Baseline |
| Female | 22 to 35 | 0 | #NUM! | 0 | Baseline |
| Male | 15 to 21 | 0 | #NUM! | 0 | Baseline |
| Male | 1 to 4 | 0 | #NUM! | 0 | Baseline |
| Female | 15 to 21 | 0 | #NUM! | 0 | Baseline |
| Male | 22 to 35 | 0 | #NUM! | 0 | Baseline |
| Female | 22 to 35 | 0 | #NUM! | 0 | Baseline |
| Male | 35+ | 0 | #NUM! | 0 | Baseline |
| Male | 35+ | 0 | #NUM! | 0 | Baseline |

| Male | 35+ | 0 | #NUM! | 0 | Baseline |
| --- | --- | --- | --- | --- | --- |
| Female | 5 to 14 | 0 | #NUM! | 0 | Baseline |
| Female | 35+ | 0 | #NUM! | 0 | Baseline |
| Female | 22 to 35 | 0 | #NUM! | 0 | Baseline |
| Female | 22 to 35 | 0 | #NUM! | 0 | Baseline |
| Male | 35+ | 0 | #NUM! | 0 | Baseline |
| Female | 1 to 4 | 0 | #NUM! | 0 | Baseline |
| Female | 5 to 14 | 0 | #NUM! | 0 | Baseline |
| Female | 5 to 14 | 0 | #NUM! | 0 | Baseline |
| Male | 1 to 4 | 0 | #NUM! | 0 | Baseline |
| Female | 1 to 4 | 0 | #NUM! | 0 | Baseline |
| Male | 22 to 35 | 0 | #NUM! | 0 | Baseline |
| Male | 22 to 35 | 0 | #NUM! | 0 | Baseline |
| Male | 15 to 21 | 0 | #NUM! | 0 | Baseline |
| Male | 22 to 35 | 0 | #NUM! | 0 | Baseline |
| Female | 15 to 21 | 0 | #NUM! | 0 | Baseline |
| Male | 22 to 35 | 0 | #NUM! | 0 | Baseline |
| Female | 22 to 35 | 0 | #NUM! | 0 | Baseline |
| Female | 22 to 35 | 0 | #NUM! | 0 | Baseline |
| Male | 5 to 14 | 0 | #NUM! | 0 | Baseline |
| Female | 5 to 14 | 0 | #NUM! | 0 | Baseline |
| Male | 5 to 14 | 0 | #NUM! | 0 | Baseline |
| Female | 5 to 14 | 0 | #NUM! | 0 | Baseline |
| Male | 5 to 14 | 0 | #NUM! | 0 | Baseline |
| Male | 5 to 14 | 0 | #NUM! | 0 | Baseline |
| Male | 5 to 14 | 0 | #NUM! | 0 | Baseline |
| Male | 5 to 14 | 0 | #NUM! | 0 | Baseline |
| Male | 35+ | 0 | #NUM! | 0 | Baseline |
| Female | 22 to 35 | 0 | #NUM! | 0 | Baseline |
| Male | 1 to 4 | 0 | #NUM! | 0 | Baseline |
| Female | 1 to 4 | 0 | #NUM! | 0 | Baseline |
| Female | 5 to 14 | 0 | #NUM! | 0 | Baseline |
| Female | 22 to 35 | 0 | #NUM! | 0 | Baseline |
| Female | 5 to 14 | 0 | #NUM! | 0 | Baseline |
| Female | 5 to 14 | 0 | #NUM! | 0 | Baseline |
| Male | 1 to 4 | 0 | #NUM! | 0 | Baseline |
| Male | 1 to 4 | 0 | #NUM! | 0 | Baseline |
| Female | 5 to 14 | 0 | #NUM! | 0 | Baseline |
| Female | 22 to 35 | 0 | #NUM! | 0 | Baseline |
| Female | 35+ | 0 | #NUM! | 0 | Baseline |
| Male | 22 to 35 | 0 | #NUM! | 0 | Baseline |
| Male | 15 to 21 | 0 | #NUM! | 0 | Baseline |
| Male | 35+ | 0 | #NUM! | 0 | Baseline |
| Female | 35+ | 0 | #NUM! | 0 | Baseline |
| Female | 15 to 21 | 0 | #NUM! | 0 | Baseline |
| Male | 22 to 35 | 0 | #NUM! | 0 | Baseline |
| Male | 35+ | 0 | #NUM! | 0 | Baseline |
| Female | 22 to 35 | 0 | #NUM! | 0 | Baseline |
| Male | 35+ | 0 | #NUM! | 0 | Baseline |
| Female | 35+ | 0 | #NUM! | 0 | Baseline |

| Female | 22 to 35 | 0 | #NUM! | 0 | Baseline |
| --- | --- | --- | --- | --- | --- |
| Male | 5 to 14 | 0 | #NUM! | 0 | Baseline |
| Female | 5 to 14 | 0 | #NUM! | 0 | Baseline |
| Male | 5 to 14 | 0 | #NUM! | 0 | Baseline |
| Female | 5 to 14 | 0 | #NUM! | 0 | Baseline |
| Male | 5 to 14 | 0 | #NUM! | 0 | Baseline |
| Male | 5 to 14 | 0 | #NUM! | 0 | Baseline |
| Female | 5 to 14 | 0 | #NUM! | 0 | Baseline |
| Female | 5 to 14 | 0 | #NUM! | 0 | Baseline |
| Male | 5 to 14 | 0 | #NUM! | 0 | Baseline |
| Male | 15 to 21 | 0 | #NUM! | 0 | Baseline |
| Male | 5 to 14 | 0 | #NUM! | 0 | Baseline |
| Male | 5 to 14 | 0 | #NUM! | 0 | Baseline |
| Female | 5 to 14 | 0 | #NUM! | 0 | Baseline |
| Male | 5 to 14 | 0 | #NUM! | 0 | Baseline |
| Female | 22 to 35 | 0 | #NUM! | 0 | Baseline |
| Female | 22 to 35 | 0 | #NUM! | 0 | Baseline |
| Male | 5 to 14 | 0 | #NUM! | 0 | Baseline |
| Female | 5 to 14 | 0 | #NUM! | 0 | Baseline |
| Female | 5 to 14 | 0 | #NUM! | 0 | Baseline |
| Male | 5 to 14 | 0 | #NUM! | 0 | Baseline |
| Female | 5 to 14 | 0 | #NUM! | 0 | Baseline |
| Male | 5 to 14 | 0 | #NUM! | 0 | Baseline |
| Female | 5 to 14 | 0 | #NUM! | 0 | Baseline |
| Female | 5 to 14 | 0 | #NUM! | 0 | Baseline |
| Female | 5 to 14 | 0 | #NUM! | 0 | Baseline |
| Male | 5 to 14 | 0 | #NUM! | 0 | Baseline |
| Male | 5 to 14 | 0 | #NUM! | 0 | Baseline |
| Male | 15 to 21 | 0 | #NUM! | 0 | Baseline |
| Female | 15 to 21 | 0 | #NUM! | 0 | Baseline |
| Male | 35+ | 0 | #NUM! | 0 | Baseline |
| Female | 1 to 4 | 0 | #NUM! | 0 | Baseline |
| Female | 15 to 21 | 0 | #NUM! | 0 | Baseline |
| Male | 1 to 4 | 0 | #NUM! | 0 | Baseline |
| Female | 22 to 35 | 0 | #NUM! | 0 | Baseline |
| Male | 15 to 21 | 0 | #NUM! | 0 | Baseline |
| Male | 35+ | 0 | #NUM! | 0 | Baseline |
| Male | 22 to 35 | 0 | #NUM! | 0 | Baseline |
| Male | 1 to 4 | 0 | #NUM! | 0 | Baseline |
| Female | 22 to 35 | 0 | #NUM! | 0 | Baseline |
| Female | 35+ | 0 | #NUM! | 0 | Baseline |
| Male | 35+ | 0 | #NUM! | 0 | Baseline |
| Female | 22 to 35 | 0 | #NUM! | 0 | Baseline |
| Male | 5 to 14 | 0 | #NUM! | 0 | Baseline |
| Male | 15 to 21 | 0 | #NUM! | 0 | Baseline |
| Male | 15 to 21 | 0 | #NUM! | 0 | Baseline |
| Female | 15 to 21 | 0 | #NUM! | 0 | Baseline |
| Female | 35+ | 0 | #NUM! | 0 | Baseline |
| Male | 35+ | 0 | #NUM! | 0 | Baseline |
| Male | 1 to 4 | 0 | #NUM! | 0 | Baseline |

| Female | 35+ | 0 | #NUM! | 0 | Baseline |
| --- | --- | --- | --- | --- | --- |
| Female | 35+ | 0 | #NUM! | 0 | Baseline |
| Male | 35+ | 0 | #NUM! | 0 | Baseline |
| Male | 15 to 21 | 0 | #NUM! | 0 | Baseline |
| Female | 1 to 4 | 0 | #NUM! | 0 | Baseline |
| Male | 35+ | 0 | #NUM! | 0 | Baseline |
| Male | 1 to 4 | 0 | #NUM! | 0 | Baseline |
| Female | 35+ | 0 | #NUM! | 0 | Baseline |
| Female | 35+ | 0 | #NUM! | 0 | Baseline |
| Male | 22 to 35 | 0 | #NUM! | 0 | Baseline |
| Female | 15 to 21 | 0 | #NUM! | 0 | Baseline |
| Male | 15 to 21 | 0 | #NUM! | 0 | Baseline |
| Male | 22 to 35 | 0 | #NUM! | 0 | Baseline |
| Male | 5 to 14 | 0 | #NUM! | 0 | Baseline |
| Female | 15 to 21 | 0 | #NUM! | 0 | Baseline |
| Female | 5 to 14 | 0 | #NUM! | 0 | Baseline |
| Male | 1 to 4 | 0 | #NUM! | 0 | Baseline |
| Female | 22 to 35 | 0 | #NUM! | 0 | Baseline |
| Female | 15 to 21 | 0 | #NUM! | 0 | Baseline |
| Male | 35+ | 0 | #NUM! | 0 | Baseline |
| Female | 22 to 35 | 0 | #NUM! | 0 | Baseline |
| Female | 1 to 4 | 0 | #NUM! | 0 | Baseline |
| Female | 35+ | 0 | #NUM! | 0 | Baseline |
| Female | 1 to 4 | 0 | #NUM! | 0 | Baseline |
| Female | 22 to 35 | 0 | #NUM! | 0 | Baseline |
| Male | 15 to 21 | 0 | #NUM! | 0 | Baseline |
| Female | 1 to 4 | 0 | #NUM! | 0 | Baseline |
| Male | 15 to 21 | 0 | #NUM! | 0 | Baseline |
| Male | 15 to 21 | 0 | #NUM! | 0 | Baseline |
| Female | 5 to 14 | 0 | #NUM! | 0 | Baseline |
| Male | 15 to 21 | 0 | #NUM! | 0 | Baseline |
| Male | 22 to 35 | 0 | #NUM! | 0 | Baseline |
| Male | 22 to 35 | 0 | #NUM! | 0 | Baseline |
| Male | 35+ | 0 | #NUM! | 0 | Baseline |
| Male | 22 to 35 | 0 | #NUM! | 0 | Baseline |
| Female | 1 to 4 | 0 | #NUM! | 0 | Baseline |
| Female | 22 to 35 | 0 | #NUM! | 0 | Baseline |
| Female | 15 to 21 | 0 | #NUM! | 0 | Baseline |
| Male | 35+ | 0 | #NUM! | 0 | Baseline |
| Female | 1 to 4 | 0 | #NUM! | 0 | Baseline |
| Female | 22 to 35 | 0 | #NUM! | 0 | Baseline |
| Female | 35+ | 0 | #NUM! | 0 | Baseline |
| Male | 1 to 4 | 0 | #NUM! | 0 | Baseline |
| Female | 1 to 4 | 0 | #NUM! | 0 | Baseline |
| Male | 22 to 35 | 0 | #NUM! | 0 | Baseline |
| Female | 22 to 35 | 0 | #NUM! | 0 | Baseline |
| Female | 1 to 4 | 0 | #NUM! | 0 | Baseline |
| Female | 1 to 4 | 0 | #NUM! | 0 | Baseline |
| Male | 1 to 4 | 0 | #NUM! | 0 | Baseline |
| Male | 35+ |  | #NUM! | 0 | Baseline |

| Female | 22 to 35 | 0 | #NUM! | 0 | Baseline |
| --- | --- | --- | --- | --- | --- |
| Female | 22 to 35 | 0 | #NUM! | 0 | Baseline |
| Female | 5 to 14 | 0 | #NUM! | 0 | Baseline |
| Female | 35+ | 0 | #NUM! | 0 | Baseline |
| Male | 1 to 4 | 0 | #NUM! | 0 | Baseline |
| Male | 22 to 35 | 0 | #NUM! | 0 | Baseline |
| Male | 35+ | 0 | #NUM! | 0 | Baseline |
| Female | 22 to 35 | 0 | #NUM! | 0 | Baseline |
| Female | 5 to 14 | 0 | #NUM! | 0 | Baseline |
| Male | 5 to 14 | 0 | #NUM! | 0 | Baseline |
| Male | 22 to 35 | 0 | #NUM! | 0 | Baseline |
| Female | 22 to 35 | 0 | #NUM! | 0 | Baseline |
| Male | 5 to 14 | 0 | #NUM! | 0 | Baseline |
| Male | 22 to 35 | 0 | #NUM! | 0 | Baseline |
| Female | 22 to 35 | 0 | #NUM! | 0 | Baseline |
| Female | 35+ | 0 | #NUM! | 0 | Baseline |
| Male | 5 to 14 | 0 | #NUM! | 0 | Baseline |
| Female | 1 to 4 | 5 | 0 | 0 | Baseline |
| Female | 22 to 35 | 0 | #NUM! | 0 | Baseline |
| Male | 5 to 14 | 0 | #NUM! | 0 | Baseline |
| Female | 5 to 14 | 0 | #NUM! | 0 | Baseline |
| Female | 15 to 21 | 0 | #NUM! | 0 | Baseline |
| Female | 15 to 21 | 0 | #NUM! | 0 | Baseline |
| Male | 35+ | 0 | #NUM! | 0 | Baseline |
| Male | 5 to 14 | 0 | #NUM! | 0 | Baseline |
| Male | 5 to 14 | 0 | #NUM! | 0 | Baseline |
| Female | 35+ | 0 | #NUM! | 0 | Baseline |
| Male | 5 to 14 | 0 | #NUM! | 0 | Baseline |
| Female | 15 to 21 | 0 | #NUM! | 0 | Baseline |
| Male | 15 to 21 | 0 | #NUM! | 0 | Baseline |
| Female | 15 to 21 | 0 | #NUM! | 0 | Baseline |
| Female | 15 to 21 | 0 | #NUM! | 0 | Baseline |
| Female | 15 to 21 | 0 | #NUM! | 0 | Baseline |
| Female | 35+ |  | #NUM! | 0 | Baseline |
| Female | 22 to 35 | 0 | #NUM! | 0 | Baseline |
| Male | 35+ | 0 | #NUM! | 0 | Baseline |
| Female | 22 to 35 | 0 | #NUM! | 0 | Baseline |
| Female | 35+ | 0 | #NUM! | 0 | Baseline |
| Male | 35+ | 0 | #NUM! | 0 | Baseline |
| Male | 35+ | 0 | #NUM! | 0 | Baseline |
| Female | 1 to 4 | 4 | 0 | 0 | Baseline |
| Female | 22 to 35 | 0 | #NUM! | 0 | Baseline |
| Male | 35+ | 0 | #NUM! | 0 | Baseline |
| Female | 1 to 4 | 0 | #NUM! | 0 | Baseline |
| Female | 22 to 35 | 0 | #NUM! | 0 | Baseline |
| Female | 35+ | 0 | #NUM! | 0 | Baseline |
| Female | 35+ | 0 | #NUM! | 0 | Baseline |
| Male | 1 to 4 | 0 | #NUM! | 0 | Baseline |
| Female | 35+ | 0 | #NUM! | 0 | Baseline |
| Male | 5 to 14 | 0 | #NUM! | 0 | Baseline |

| Female | 35+ | 0 | #NUM! | 0 | Baseline |
| --- | --- | --- | --- | --- | --- |
| Female | 22 to 35 | 0 | #NUM! | 0 | Baseline |
| Male | 5 to 14 | 0 | #NUM! | 0 | Baseline |
| Male | 5 to 14 | 0 | #NUM! | 0 | Baseline |
| Male | 5 to 14 | 0 | #NUM! | 0 | Baseline |
| Female | 5 to 14 | 0 | #NUM! | 0 | Baseline |
| Female | 35+ | 0 | #NUM! | 0 | Baseline |
| Male | 5 to 14 | 0 | #NUM! | 0 | Baseline |
| Female | 22 to 35 | 0 | #NUM! | 0 | Baseline |
| Male | 5 to 14 | 0 | #NUM! | 0 | Baseline |
| Female | 22 to 35 | 0 | #NUM! | 0 | Baseline |
| Male | 15 to 21 | 0 | #NUM! | 0 | Baseline |
| Male | 15 to 21 | 0 | #NUM! | 0 | Baseline |
| Male | 35+ | 0 | #NUM! | 0 | Baseline |
| Female | 5 to 14 | 0 | #NUM! | 0 | Baseline |
| Male | 5 to 14 | 0 | #NUM! | 0 | Baseline |
| Female | 22 to 35 | 0 | #NUM! | 0 | Baseline |
| Female | 22 to 35 | 0 | #NUM! | 0 | Baseline |
| Male | 15 to 21 | 0 | #NUM! | 0 | Baseline |
| Male | 15 to 21 | 0 | #NUM! | 0 | Baseline |
| Female | 15 to 21 | 0 | #NUM! | 0 | Baseline |
| Female | 22 to 35 | 0 | #NUM! | 0 | Baseline |
| Female | 22 to 35 | 0 | #NUM! | 0 | Baseline |
| Female | 5 to 14 | 0 | #NUM! | 0 | Baseline |
| Male | 5 to 14 | 0 | #NUM! | 0 | Baseline |
| Female | 22 to 35 | 0 | #NUM! | 0 | Baseline |
| Male | 1 to 4 | 0 | #NUM! | 0 | Baseline |
| Male | 35+ | 0 | #NUM! | 0 | Baseline |
| Female | 1 to 4 | 0 | #NUM! | 0 | Baseline |
| Female | 22 to 35 | 0 | #NUM! | 0 | Baseline |
| Male | 5 to 14 | 0 | #NUM! | 0 | Baseline |
| Female | 5 to 14 | 0 | #NUM! | 0 | Baseline |
| Female | 35+ | 0 | #NUM! | 0 | Baseline |
| Female | 35+ | 0 | #NUM! | 0 | Baseline |
| Female | 1 to 4 | 0 | #NUM! | 0 | Baseline |
| Female | 22 to 35 | 0 | #NUM! | 0 | Baseline |
| Female | 22 to 35 | 0 | #NUM! | 0 | Baseline |
| Female | 35+ | 0 | #NUM! | 0 | Baseline |
| Female | 22 to 35 | 0 | #NUM! | 0 | Baseline |
| Male | 5 to 14 | 0 | #NUM! | 0 | Baseline |
| Male | 22 to 35 | 0 | #NUM! | 0 | Baseline |
| Female | 15 to 21 | 0 | #NUM! | 0 | Baseline |
| Female | 22 to 35 | 0 | #NUM! | 0 | Baseline |
| Female | 22 to 35 | 0 | #NUM! | 0 | Baseline |
| Female | 5 to 14 | 0 | #NUM! | 0 | Baseline |
| Female | 22 to 35 | 0 | #NUM! | 0 | Baseline |
| Male | 35+ | 0 | #NUM! | 0 | Baseline |
| Male | 5 to 14 | 5 | 0 | 0 | Baseline |
| Male | 5 to 14 | 0 | #NUM! | 0 | Baseline |
| Male | 22 to 35 | 0 | #NUM! | 0 | Baseline |

| Female | 5 to 14 | 0 | #NUM! | 0 | Baseline |
| --- | --- | --- | --- | --- | --- |
| Female | 35+ | 0 | #NUM! | 0 | Baseline |
| Female | 35+ | 0 | #NUM! | 0 | Baseline |
| Male | 5 to 14 | 0 | #NUM! | 0 | Baseline |
| Female | 22 to 35 | 0 | #NUM! | 0 | Baseline |
| Female | 35+ | 0 | #NUM! | 0 | Baseline |
| Female | 35+ | 0 | #NUM! | 0 | Baseline |
| Male | 35+ | 0 | #NUM! | 0 | Baseline |
| Female | 35+ | 5 | 0 | 0 | Baseline |
| Female | 22 to 35 | 0 | #NUM! | 0 | Baseline |
| Female | 5 to 14 | 0 | #NUM! | 0 | Baseline |
| Female | 22 to 35 | 0 | #NUM! | 0 | Baseline |
| Male | 5 to 14 | 0 | #NUM! | 0 | Baseline |
| Male | 15 to 21 | 0 | #NUM! | 0 | Baseline |
| Male | 5 to 14 | 0 | #NUM! | 0 | Baseline |
| Female | 5 to 14 | 0 | #NUM! | 0 | Baseline |
| Female | 5 to 14 | 0 | #NUM! | 0 | Baseline |
| Male | 5 to 14 | 0 | #NUM! | 0 | Baseline |
| Female | 5 to 14 | 0 | #NUM! | 0 | Baseline |
| Female | 22 to 35 | 0 | #NUM! | 0 | Baseline |
| Male | 5 to 14 | 0 | #NUM! | 0 | Baseline |
| Male | 1 to 4 | 0 | #NUM! | 0 | Baseline |
| Female | 22 to 35 | 0 | #NUM! | 0 | Baseline |
| Female | 5 to 14 | 0 | #NUM! | 0 | Baseline |
| Male | 5 to 14 | 0 | #NUM! | 0 | Baseline |
| Female | 22 to 35 | 0 | #NUM! | 0 | Baseline |
| Male | 5 to 14 | 0 | #NUM! | 0 | Baseline |
| Female | 15 to 21 | 0 | #NUM! | 0 | Baseline |
| Male | 5 to 14 | 0 | #NUM! | 0 | Baseline |
| Male | 15 to 21 | 0 | #NUM! | 0 | Baseline |
| Female | 15 to 21 | 0 | #NUM! | 0 | Baseline |
| Female | 35+ | 0 | #NUM! | 0 | Baseline |
| Male | 15 to 21 | 0 | #NUM! | 0 | Baseline |
| Female | 15 to 21 | 0 | #NUM! | 0 | Baseline |
| Male | 15 to 21 | 0 | #NUM! | 0 | Baseline |
| Female | 15 to 21 | 0 | #NUM! | 0 | Baseline |
| Male | 15 to 21 | 3 | 0 | 0 | Baseline |
| Male | 15 to 21 | 0 | #NUM! | 0 | Baseline |
| Female | 35+ | 0 | #NUM! | 0 | Baseline |
| Female | 35+ | 0 | #NUM! | 0 | Baseline |
| Female | 5 to 14 | 0 | #NUM! | 0 | Baseline |
| Male | 5 to 14 | 0 | #NUM! | 0 | Baseline |
| Male | 5 to 14 | 0 | #NUM! | 0 | Baseline |
| Female | 5 to 14 | 0 | #NUM! | 0 | Baseline |
| Male | 5 to 14 | 0 | #NUM! | 0 | Baseline |
| Female | 5 to 14 | 0 | #NUM! | 0 | Baseline |
| Female | 1 to 4 | 0 | #NUM! | 0 | Baseline |
| Male | 5 to 14 | 0 | #NUM! | 0 | Baseline |
| Male | 5 to 14 | 0 | #NUM! | 0 | Baseline |
| Male | 15 to 21 | 0 | #NUM! | 0 | Baseline |

| Male | 5 to 14 | 0 | #NUM! | 0 | Baseline |
| --- | --- | --- | --- | --- | --- |
| Male | 5 to 14 | 0 | #NUM! | 0 | Baseline |
| Female | 5 to 14 | 0 | #NUM! | 0 | Baseline |
| Male | 5 to 14 | 0 | #NUM! | 0 | Baseline |
| Male | 15 to 21 | 0 | #NUM! | 0 | Baseline |
| Female | 5 to 14 | 0 | #NUM! | 0 | Baseline |
| Male | 5 to 14 | 0 | #NUM! | 0 | Baseline |
| Female | 5 to 14 | 0 | #NUM! | 0 | Baseline |
| Male | 5 to 14 | 0 | #NUM! | 0 | Baseline |
| Female | 5 to 14 | 0 | #NUM! | 0 | Baseline |
| Male | 5 to 14 | 0 | #NUM! | 0 | Baseline |
| Male | 1 to 4 | 0 | #NUM! | 0 | Baseline |
| Female | 5 to 14 | 0 | #NUM! | 0 | Baseline |
| Male | 15 to 21 | 0 | #NUM! | 0 | Baseline |
| Male | 5 to 14 | 0 | #NUM! | 0 | Baseline |
| Male | 5 to 14 | 0 | #NUM! | 0 | Baseline |
| Female | 15 to 21 | 0 | #NUM! | 0 | Baseline |
| Male | 5 to 14 | 0 | #NUM! | 0 | Baseline |
| Female | 22 to 35 | 0 | #NUM! | 0 | Baseline |
| Female | 22 to 35 | 4 | 0 | 0 | Baseline |
| Male | 22 to 35 | 0 | #NUM! | 0 | Baseline |
| Male | 35+ | 0 | #NUM! | 0 | Baseline |
| Male | 35+ | 0 | #NUM! | 0 | Baseline |
| Female | 22 to 35 | 0 | #NUM! | 0 | Baseline |
| Male | 35+ | 0 | #NUM! | 0 | Baseline |
| Female | 15 to 21 | 0 | #NUM! | 0 | Baseline |
| Male | 35+ | 0 | #NUM! | 0 | Baseline |
| Female | 22 to 35 | 0 | #NUM! | 0 | Baseline |
| Female | 35+ | 0 | #NUM! | 0 | Baseline |
| Female | 22 to 35 | 0 | #NUM! | 0 | Baseline |
| Female | 15 to 21 | 0 | #NUM! | 0 | Baseline |
| Male | 35+ | 0 | #NUM! | 0 | Baseline |
| Female | 22 to 35 | 0 | #NUM! | 0 | Baseline |
| Male | 22 to 35 | 0 | #NUM! | 0 | Baseline |
| Male | 35+ | 0 | #NUM! | 0 | Baseline |
| Female | 22 to 35 | 0 | #NUM! | 0 | Baseline |
| Female | 15 to 21 | 0 | #NUM! | 0 | Baseline |
| Female | 22 to 35 | 0 | #NUM! | 0 | Baseline |
| Female | 22 to 35 | 0 | #NUM! | 0 | Baseline |
| Male | 5 to 14 | 0 | #NUM! | 0 | Baseline |
| Male | 35+ | 0 | #NUM! | 0 | Baseline |
| Male | 22 to 35 | 0 | #NUM! | 0 | Baseline |
| Female | 35+ | 0 | #NUM! | 0 | Baseline |
| Male | 5 to 14 | 0 | #NUM! | 0 | Baseline |
| Male | 22 to 35 | 0 | #NUM! | 0 | Baseline |
| Male | 5 to 14 | 0 | #NUM! | 0 | Baseline |
| Female | 22 to 35 | 0 | #NUM! | 0 | Baseline |
| Female | 1 to 4 | 0 | #NUM! | 0 | Baseline |
| Male | 35+ | 0 | #NUM! | 0 | Baseline |
| Male | 35+ | 0 | #NUM! | 0 | Baseline |

| Female | 5 to 14 | 0 | #NUM! | 0 | Baseline |
| --- | --- | --- | --- | --- | --- |
| Male | 22 to 35 | 0 | #NUM! | 0 | Baseline |
| Male | 22 to 35 | 0 | #NUM! | 0 | Baseline |
| Male | 22 to 35 | 0 | #NUM! | 0 | Baseline |
| Female | 22 to 35 | 0 | #NUM! | 0 | Baseline |
| Female | 22 to 35 | 0 | #NUM! | 0 | Baseline |
| Female | 22 to 35 | 0 | #NUM! | 0 | Baseline |
| Male | 35+ | 0 | #NUM! | 0 | Baseline |
| Male | 22 to 35 | 0 | #NUM! | 0 | Baseline |
| Male | 5 to 14 | 0 | #NUM! | 0 | Baseline |
| Female | 15 to 21 | 0 | #NUM! | 0 | Baseline |
| Male | 5 to 14 | 0 | #NUM! | 0 | Baseline |
| Female | 5 to 14 | 0 | #NUM! | 0 | Baseline |
| Male | 15 to 21 | 0 | #NUM! | 0 | Baseline |
| Female | 22 to 35 | 0 | #NUM! | 0 | Baseline |
| Male | 15 to 21 | 0 | #NUM! | 0 | Baseline |
| Male | 1 to 4 | 0 | #NUM! | 0 | Baseline |
| Female | 5 to 14 | 0 | #NUM! | 0 | Baseline |
| Female | 1 to 4 | 0 | #NUM! | 0 | Baseline |
| Female | 5 to 14 | 0 | #NUM! | 0 | Baseline |
| Male | 5 to 14 | 0 | #NUM! | 0 | Baseline |
| Male | 5 to 14 | 0 | #NUM! | 0 | Baseline |
| Female | 15 to 21 | 0 | #NUM! | 0 | Baseline |
| Female | 15 to 21 | 0 | #NUM! | 0 | Baseline |
| Male | 5 to 14 | 0 | #NUM! | 0 | Baseline |
| Male | 5 to 14 | 0 | #NUM! | 0 | Baseline |
| Female | 5 to 14 | 0 | #NUM! | 0 | Baseline |
| Female | 22 to 35 | 0 | #NUM! | 0 | Baseline |
| Female | 5 to 14 | 0 | #NUM! | 0 | Baseline |
| Female | 1 to 4 | 0 | #NUM! | 0 | Baseline |
| Female | 1 to 4 | 0 | #NUM! | 0 | Baseline |
| Male | 1 to 4 | 0 | #NUM! | 0 | Baseline |
| Female | 5 to 14 | 0 | #NUM! | 0 | Baseline |
| Female | 22 to 35 | 0 | #NUM! | 0 | Baseline |
| Male | 22 to 35 | 0 | #NUM! | 0 | Baseline |
| Male | 15 to 21 | 0 | #NUM! | 0 | Baseline |
| Male | 1 to 4 | 0 | #NUM! | 0 | Baseline |
| Male | 35+ | 0 | #NUM! | 0 | Baseline |
| Male | 5 to 14 | 0 | #NUM! | 0 | Baseline |
| Female | 22 to 35 | 0 | #NUM! | 0 | Baseline |
| Female | 22 to 35 | 0 | #NUM! | 0 | Baseline |
| Male | 35+ | 0 | #NUM! | 0 | Baseline |
| Female | 5 to 14 | 0 | #NUM! | 0 | Baseline |
| Female | 15 to 21 | 0 | #NUM! | 0 | Baseline |
| Female | 22 to 35 | 0 | #NUM! | 0 | Baseline |
| Male | 22 to 35 | 0 | #NUM! | 0 | Baseline |
| Female | 22 to 35 | 0 | #NUM! | 0 | Baseline |
| Male | 15 to 21 | 0 | #NUM! | 0 | Baseline |
| Female | 1 to 4 | 0 | #NUM! | 0 | Baseline |
| Male | 15 to 21 | 0 | #NUM! | 0 | Baseline |

| Female | 5 to 14 | 0 | #NUM! | 0 | Baseline |
| --- | --- | --- | --- | --- | --- |
| Male | 5 to 14 | 0 | #NUM! | 0 | Baseline |
| Male | 15 to 21 | 0 | #NUM! | 0 | Baseline |
| Male | 15 to 21 | 0 | #NUM! | 0 | Baseline |
| Female | 15 to 21 | 0 | #NUM! | 0 | Baseline |
| Female | 5 to 14 | 0 | #NUM! | 0 | Baseline |
| Male | 5 to 14 | 0 | #NUM! | 0 | Baseline |
| Female | 35+ | 0 | #NUM! | 0 | Baseline |
| Male | 22 to 35 | 0 | #NUM! | 0 | Baseline |
| Female | 22 to 35 | 0 | #NUM! | 0 | Baseline |
| Male | 15 to 21 | 0 | #NUM! | 0 | Baseline |
| Male | 35+ | 0 | #NUM! | 0 | Baseline |
| Male | 5 to 14 | 0 | #NUM! | 0 | Baseline |
| Female | 22 to 35 | 0 | #NUM! | 0 | Baseline |
| Female | 22 to 35 | 0 | #NUM! | 0 | Baseline |
| Female | 35+ | 0 | #NUM! | 0 | Baseline |
| Male | 35+ | 0 | #NUM! | 0 | Baseline |
| Male | 15 to 21 | 0 | #NUM! | 0 | Baseline |
| Male | 1 to 4 | 0 | #NUM! | 0 | Baseline |
| Male | 35+ | 0 | #NUM! | 0 | Baseline |
| Male | 35+ | 0 | #NUM! | 0 | Baseline |
| Female | 22 to 35 | 0 | #NUM! | 0 | Baseline |
| Female | 35+ | 0 | #NUM! | 0 | Baseline |
| Female | 35+ | 0 | #NUM! | 0 | Baseline |
| Female | 22 to 35 | 0 | #NUM! | 0 | Baseline |
| Female | 22 to 35 | 0 | #NUM! | 0 | Baseline |
| Female | 35+ | 0 | #NUM! | 0 | Baseline |
| Female | 35+ | 0 | #NUM! | 0 | Baseline |
| Female | 35+ | 0 | #NUM! | 0 | Baseline |
| Female | 35+ | 0 | #NUM! | 0 | Baseline |
| Female | 15 to 21 | 0 | #NUM! | 0 | Baseline |
| Female | 35+ | 0 | #NUM! | 0 | Baseline |
| Female | 15 to 21 | 0 | #NUM! | 0 | Baseline |
| Male | 22 to 35 | 0 | #NUM! | 0 | Baseline |
| Female | 15 to 21 | 0 | #NUM! | 0 | Baseline |
| Female | 22 to 35 | 0 | #NUM! | 0 | Baseline |
| Male | 15 to 21 | 0 | #NUM! | 0 | Baseline |
| Male | 15 to 21 | 0 | #NUM! | 0 | Baseline |
| Male | 15 to 21 | 0 | #NUM! | 0 | Baseline |
| Male | 35+ | 0 | #NUM! | 0 | Baseline |
| Male | 15 to 21 | 0 | #NUM! | 0 | Baseline |
| Female | 35+ | 0 | #NUM! | 0 | Baseline |
| Male | 5 to 14 | 0 | #NUM! | 0 | Baseline |
| Male | 15 to 21 | 0 | #NUM! | 0 | Baseline |
| Male | 15 to 21 | 0 | #NUM! | 0 | Baseline |
| Male | 15 to 21 | 0 | #NUM! | 0 | Baseline |
| Female | 15 to 21 | 0 | #NUM! | 0 | Baseline |
| Female | 35+ | 0 | #NUM! | 0 | Baseline |
| Female | 35+ | 0 | #NUM! | 0 | Baseline |
| Male | 22 to 35 | 0 | #NUM! | 0 | Baseline |

| Male | 35+ | 0 | #NUM! | 0 | Baseline |
| --- | --- | --- | --- | --- | --- |
| Female | 35+ | 0 | #NUM! | 0 | Baseline |
| Male | 22 to 35 | 0 | #NUM! | 0 | Baseline |
| Female | 35+ | 0 | #NUM! | 0 | Baseline |
| Female | 22 to 35 | 0 | #NUM! | 0 | Baseline |
| Female | 15 to 21 | 0 | #NUM! | 0 | Baseline |
| Female | 35+ | 0 | #NUM! | 0 | Baseline |
| Female | 22 to 35 | 0 | #NUM! | 0 | Baseline |
| Female | 15 to 21 | 0 | #NUM! | 0 | Baseline |
| Female | 22 to 35 | 0 | #NUM! | 0 | Baseline |
| Female | 35+ | 1 | 0 | 0 | Baseline |
| Female | 15 to 21 | 0 | #NUM! | 0 | Baseline |
| Male | 5 to 14 | 0 | #NUM! | 0 | Baseline |
| Male | 22 to 35 | 0 | #NUM! | 0 | Baseline |
| Male | 35+ | 0 | #NUM! | 0 | Baseline |
| Male | 15 to 21 | 0 | #NUM! | 0 | Baseline |
| Female | 5 to 14 | 0 | #NUM! | 0 | Baseline |
| Male | 35+ | 0 | #NUM! | 0 | Baseline |
| Female | 5 to 14 | 0 | #NUM! | 0 | Baseline |
| Male | 1 to 4 | 0 | #NUM! | 0 | Baseline |
| Male | 1 to 4 | 0 | #NUM! | 0 | Baseline |
| Male | 1 to 4 | 0 | #NUM! | 0 | Baseline |
| Male | 1 to 4 | 0 | #NUM! | 0 | Baseline |
| Male | 1 to 4 | 0 | #NUM! | 0 | Baseline |
| Male | 1 to 4 | 0 | #NUM! | 0 | Baseline |
| Male | 1 to 4 | 0 | #NUM! | 0 | Baseline |
| Male | 1 to 4 | 0 | #NUM! | 0 | Baseline |
| Female | 22 to 35 | 0 | #NUM! | 0 | Baseline |
| Male | 1 to 4 | 0 | #NUM! | 0 | Baseline |
| Male | 15 to 21 | 0 | #NUM! | 0 | Baseline |
| Female | 5 to 14 | 0 | #NUM! | 0 | Baseline |
| Female | 15 to 21 | 0 | #NUM! | 0 | Baseline |
| Female | 5 to 14 | 0 | #NUM! | 0 | Baseline |
| Male | 5 to 14 | 0 | #NUM! | 0 | Baseline |
| Female | 1 to 4 | 0 | #NUM! | 0 | Baseline |
| Male | 22 to 35 | 0 | #NUM! | 0 | Baseline |
| Male | 1 to 4 | 0 | #NUM! | 0 | Baseline |
| Male | 1 to 4 | 0 | #NUM! | 0 | Baseline |
| Male | 22 to 35 | 0 | #NUM! | 0 | Baseline |
| Female | 35+ | 0 | #NUM! | 0 | Baseline |
| Male | 22 to 35 | 0 | #NUM! | 0 | Baseline |
| Male | 15 to 21 | 0 | #NUM! | 0 | Baseline |
| Male | 22 to 35 | 0 | #NUM! | 0 | Baseline |
| Male | 22 to 35 | 0 | #NUM! | 0 | Baseline |
| Female | 5 to 14 | 0 | #NUM! | 0 | Baseline |
| Female | 22 to 35 | 0 | #NUM! | 0 | Baseline |
| Male | 15 to 21 | 0 | #NUM! | 0 | Baseline |
| Male | 1 to 4 | 0 | #NUM! | 0 | Baseline |
| Female | 5 to 14 | 0 | #NUM! | 0 | Baseline |
| Female | 22 to 35 | 0 | #NUM! | 0 | Baseline |

| Male | 5 to 14 | 0 | #NUM! | 0 | Baseline |
| --- | --- | --- | --- | --- | --- |
| Male | 35+ | 0 | #NUM! | 0 | Baseline |
| Female | 22 to 35 | 0 | #NUM! | 0 | Baseline |
| Male | 1 to 4 | 0 | #NUM! | 0 | Baseline |
| Female | 35+ | 0 | #NUM! | 0 | Baseline |
| Female | 15 to 21 | 0 | #NUM! | 0 | Baseline |
| Female | 22 to 35 | 0 | #NUM! | 0 | Baseline |
| Male | 35+ | 0 | #NUM! | 0 | Baseline |
| Female | 35+ | 0 | #NUM! | 0 | Baseline |
| Male | 35+ | 0 | #NUM! | 0 | Baseline |
| Female | 15 to 21 | 0 | #NUM! | 0 | Baseline |
| Female | 5 to 14 | 0 | #NUM! | 0 | Baseline |
| Male | 35+ | 0 | #NUM! | 0 | Baseline |
| Female | 15 to 21 | 0 | #NUM! | 0 | Baseline |
| Male | 15 to 21 | 0 | #NUM! | 0 | Baseline |
| Male | 1 to 4 | 0 | #NUM! | 0 | Baseline |
| Female | 35+ | 0 | #NUM! | 0 | Baseline |
| Female | 22 to 35 | 0 | #NUM! | 0 | Baseline |
| Male | 1 to 4 | 0 | #NUM! | 0 | Baseline |
| Female | 35+ | 0 | #NUM! | 0 | Baseline |
| Female | 35+ | 0 | #NUM! | 0 | Baseline |
| Male | 1 to 4 | 0 | #NUM! | 0 | Baseline |
| Female | 5 to 14 | 0 | #NUM! | 0 | Baseline |
| Female | 22 to 35 | 0 | #NUM! | 0 | Baseline |
| Female | 22 to 35 | 0 | #NUM! | 0 | Baseline |
| Female | 15 to 21 | 0 | #NUM! | 0 | Baseline |
| Female | 15 to 21 | 0 | #NUM! | 0 | Baseline |
| Female | 22 to 35 | 0 | #NUM! | 0 | Baseline |
| Male | 15 to 21 | 0 | #NUM! | 0 | Baseline |
| Male | 15 to 21 | 0 | #NUM! | 0 | Baseline |
| Male | 35+ | 0 | #NUM! | 0 | Baseline |
| Female | 5 to 14 | 0 | #NUM! | 0 | Baseline |
| Female | 22 to 35 | 0 | #NUM! | 0 | Baseline |
| Male | 1 to 4 | 0 | #NUM! | 0 | Baseline |
| Female | 22 to 35 | 0 | #NUM! | 0 | Baseline |
| Male | 35+ |  | #NUM! | 0 | Baseline |
| Male | 35+ |  | #NUM! | 0 | Baseline |
| Male | 35+ |  | #NUM! | 0 | Baseline |
| Female | 5 to 14 |  | #NUM! | 0 | Baseline |
| Male | 35+ |  | #NUM! | 0 | Baseline |
| Female | 15 to 21 |  | #NUM! | 0 | Baseline |
| Male | 35+ |  | #NUM! | 0 | Baseline |
| Male | 35+ |  | #NUM! | 0 | Baseline |
| Male | 35+ |  | #NUM! | 0 | Baseline |
| Female | 22 to 35 |  | #NUM! | 0 | Baseline |
| Female | 15 to 21 |  | #NUM! | 0 | Baseline |
| Male | 35+ |  | #NUM! | 0 | Baseline |
| Male | 1 to 4 | 0 | #NUM! | 0 | Baseline |
| Male | 5 to 14 | 0 | #NUM! | 0 | Baseline |
| Female | 15 to 21 | 0 | #NUM! | 0 | Baseline |

| Male | 5 to 14 | 0 | #NUM! | 0 | Baseline |
| --- | --- | --- | --- | --- | --- |
| Male | 15 to 21 | 0 | #NUM! | 0 | Baseline |
| Female | 15 to 21 | 0 | #NUM! | 0 | Baseline |
| Male | 5 to 14 | 0 | #NUM! | 0 | Baseline |
| Male | 5 to 14 | 0 | #NUM! | 0 | Baseline |
| Male | 15 to 21 | 0 | #NUM! | 0 | Baseline |
| Male | 5 to 14 | 0 | #NUM! | 0 | Baseline |
| Male | 5 to 14 | 0 | #NUM! | 0 | Baseline |
| Female | 5 to 14 | 0 | #NUM! | 0 | Baseline |
| Male | 15 to 21 | 0 | #NUM! | 0 | Baseline |
| Female | 5 to 14 | 0 | #NUM! | 0 | Baseline |
| Female | 15 to 21 | 0 | #NUM! | 0 | Baseline |
| Male | 5 to 14 |  | #NUM! | 0 | Baseline |
| Male | 15 to 21 |  | #NUM! | 0 | Baseline |
| Male | 5 to 14 |  | #NUM! | 0 | Baseline |
| Male | 5 to 14 |  | #NUM! | 0 | Baseline |
| Female | 5 to 14 |  | #NUM! | 0 | Baseline |
| Male | 5 to 14 |  | #NUM! | 0 | Baseline |
| Male | 1 to 4 | 0 | #NUM! | 0 | Baseline |
| Female | 35+ | 0 | #NUM! | 0 | Baseline |
| Male | 22 to 35 | 0 | #NUM! | 0 | Baseline |
| Female | 15 to 21 | 0 | #NUM! | 0 | Baseline |
| Female | 22 to 35 | 0 | #NUM! | 0 | Baseline |
| Female | 15 to 21 | 0 | #NUM! | 0 | Baseline |
| Male | 35+ |  | #NUM! | 0 | Baseline |
| Male | 5 to 14 | 0 | #NUM! | 0 | Baseline |
| Male | 1 to 4 | 0 | #NUM! | 0 | Baseline |
| Female | 5 to 14 |  | #NUM! | 0 | Baseline |
| Male | 1 to 4 | 0 | #NUM! | 0 | Baseline |
| Female | 35+ |  | #NUM! | 0 | Baseline |
| Male | 22 to 35 |  | #NUM! | 0 | Baseline |
| Female | 1 to 4 | 0 | #NUM! | 0 | Baseline |
| Female | 22 to 35 | 0 | #NUM! | 0 | Baseline |
| Female | 35+ | 0 | #NUM! | 0 | Baseline |
| Female | 22 to 35 | 0 | #NUM! | 0 | Baseline |
| Male | 35+ |  | #NUM! | 0 | Baseline |
| Male | 35+ |  | #NUM! | 0 | Baseline |
| Female | 35+ |  | #NUM! | 0 | Baseline |
| Male | 1 to 4 |  | #NUM! | 0 | Baseline |
| Male | 5 to 14 | 0 | #NUM! | 0 | Baseline |
| Male | 35+ | 0 | #NUM! | 0 | Baseline |
| Female | 22 to 35 | 0 | #NUM! | 0 | Baseline |
| Male | 35+ | 0 | #NUM! | 0 | Baseline |
| Female | 22 to 35 | 0 | #NUM! | 0 | Baseline |
| Female | 22 to 35 | 0 | #NUM! | 0 | Baseline |
| Female | 5 to 14 | 0 | #NUM! | 0 | Baseline |
| Female | 22 to 35 | 0 | #NUM! | 0 | Baseline |
| Male | 22 to 35 | 0 | #NUM! | 0 | Baseline |
| Female | 22 to 35 | 0 | #NUM! | 0 | Baseline |
| Female | 22 to 35 | 0 | #NUM! | 0 | Baseline |

| Male | 15 to 21 | 0 | #NUM! | 0 | Baseline |
| --- | --- | --- | --- | --- | --- |
| Male | 35+ | 0 | #NUM! | 0 | Baseline |
| Female | 35+ | 0 | #NUM! | 0 | Baseline |
| Male | 22 to 35 | 0 | #NUM! | 0 | Baseline |
| Male | 22 to 35 | 0 | #NUM! | 0 | Baseline |
| Male | 22 to 35 | 0 | #NUM! | 0 | Baseline |
| Female | 22 to 35 | 0 | #NUM! | 0 | Baseline |
| Male | 22 to 35 | 0 | #NUM! | 0 | Baseline |
| Female | 35+ | 0 | #NUM! | 0 | Baseline |
| Female | 35+ | 0 | #NUM! | 0 | Baseline |
| Female | 35+ | 0 | #NUM! | 0 | Baseline |
| Male | 35+ | 0 | #NUM! | 0 | Baseline |
| Female | 22 to 35 | 0 | #NUM! | 0 | Baseline |
| Female | 5 to 14 | 0 | #NUM! | 0 | Baseline |
| Female | 22 to 35 | 0 | #NUM! | 0 | Baseline |
| Male | 5 to 14 | 0 | #NUM! | 0 | Baseline |
| Female | 15 to 21 | 0 | #NUM! | 0 | Baseline |
| Female | 35+ | 0 | #NUM! | 0 | Baseline |
| Female | 22 to 35 | 0 | #NUM! | 0 | Baseline |
| Female | 1 to 4 | 0 | #NUM! | 0 | Baseline |
| Male | 35+ | 0 | #NUM! | 0 | Baseline |
| Female | 35+ | 0 | #NUM! | 0 | Baseline |
| Female | 35+ | 0 | #NUM! | 0 | Baseline |
| Male | 15 to 21 | 0 | #NUM! | 0 | Baseline |
| Female | 22 to 35 | 0 | #NUM! | 0 | Baseline |
| Female | 35+ | 0 | #NUM! | 0 | Baseline |
| Male | 5 to 14 | 0 | #NUM! | 0 | Baseline |
| Female | 22 to 35 | 0 | #NUM! | 0 | Baseline |
| Male | 5 to 14 | 0 | #NUM! | 0 | Baseline |
| Female | 5 to 14 | 0 | #NUM! | 0 | Baseline |
| Female | 5 to 14 | 0 | #NUM! | 0 | Baseline |
| Female | 1 to 4 | 0 | #NUM! | 0 | Baseline |
| Female | 15 to 21 | 0 | #NUM! | 0 | Baseline |
| Male | 5 to 14 | 0 | #NUM! | 0 | Baseline |
| Female | 22 to 35 | 0 | #NUM! | 0 | Baseline |
| Female | 5 to 14 | 0 | #NUM! | 0 | Baseline |
| Male | 5 to 14 | 0 | #NUM! | 0 | Baseline |
| Female | 1 to 4 | 0 | #NUM! | 0 | Baseline |
| Male | 5 to 14 | 0 | #NUM! | 0 | Baseline |
| Male | 5 to 14 | 0 | #NUM! | 0 | Baseline |
| Female | 5 to 14 | 0 | #NUM! | 0 | Baseline |
| Female | 1 to 4 | 0 | #NUM! | 0 | Baseline |
| Male | 15 to 21 | 0 | #NUM! | 0 | Baseline |
| Female | 22 to 35 | 0 | #NUM! | 0 | Baseline |
| Female | 35+ | 0 | #NUM! | 0 | Baseline |
| Male | 35+ | 0 | #NUM! | 0 | Baseline |
| Female | 35+ | 0 | #NUM! | 0 | Baseline |
| Male | 35+ | 0 | #NUM! | 0 | Baseline |
| Male | 22 to 35 | 0 | #NUM! | 0 | Baseline |
| Male | 5 to 14 | 0 | #NUM! | 0 | Baseline |

| Female | 5 to 14 | 0 | #NUM! | 0 | Baseline |
| --- | --- | --- | --- | --- | --- |
| Female | 35+ | 0 | #NUM! | 0 | Baseline |
| Male | 22 to 35 | 0 | #NUM! | 0 | Baseline |
| Female | 22 to 35 | 1 | 0 | 0 | Baseline |
| Male | 1 to 4 | 4 | 0 | 0 | Baseline |
| Male | 35+ | 0 | #NUM! | 0 | Baseline |
| Female | 22 to 35 | 0 | #NUM! | 0 | Baseline |
| Male | 5 to 14 | 0 | #NUM! | 0 | Baseline |
| Male | 35+ | 0 | #NUM! | 0 | Baseline |
| Male | 5 to 14 | 0 | #NUM! | 0 | Baseline |
| Female | 35+ | 0 | #NUM! | 0 | Baseline |
| Female | 15 to 21 | 0 | #NUM! | 0 | Baseline |
| Male | 35+ | 0 | #NUM! | 0 | Baseline |
| Male | 35+ | 0 | #NUM! | 0 | Baseline |
| Male | 5 to 14 | 0 | #NUM! | 0 | Baseline |
| Male | 22 to 35 | 0 | #NUM! | 0 | Baseline |
| Male | 5 to 14 | 0 | #NUM! | 0 | Baseline |
| Male | 22 to 35 | 0 | #NUM! | 0 | Baseline |
| Male | 35+ | 0 | #NUM! | 0 | Baseline |
| Female | 35+ | 0 | #NUM! | 0 | Baseline |
| Female | 15 to 21 | 0 | #NUM! | 0 | Baseline |
| Male | 5 to 14 | 0 | #NUM! | 0 | Baseline |
| Male | 5 to 14 | 0 | #NUM! | 0 | Baseline |
| Male | 15 to 21 | 0 | #NUM! | 0 | Baseline |
| Male | 15 to 21 | 0 | #NUM! | 0 | Baseline |
| Male | 5 to 14 | 0 | #NUM! | 0 | Baseline |
| Male | 5 to 14 | 0 | #NUM! | 0 | Baseline |
| Female | 5 to 14 | 0 | #NUM! | 0 | Baseline |
| Male | 22 to 35 | 0 | #NUM! | 0 | Baseline |
| Female | 5 to 14 | 0 | #NUM! | 0 | Baseline |
| Female | 15 to 21 | 0 | #NUM! | 0 | Baseline |
| Female | 5 to 14 | 0 | #NUM! | 0 | Baseline |
| Male | 5 to 14 | 0 | #NUM! | 0 | Baseline |
| Male | 15 to 21 | 0 | #NUM! | 0 | Baseline |
| Female | 5 to 14 | 0 | #NUM! | 0 | Baseline |
| Female | 5 to 14 | 0 | #NUM! | 0 | Baseline |
| Female | 22 to 35 | 0 | #NUM! | 0 | Baseline |
| Female | 22 to 35 | 0 | #NUM! | 0 | Baseline |
| Male | 5 to 14 | 0 | #NUM! | 0 | Baseline |
| Female | 15 to 21 | 0 | #NUM! | 0 | Baseline |
| Female | 35+ | 0 | #NUM! | 0 | Baseline |
| Female | 5 to 14 | 0 | #NUM! | 0 | Baseline |
| Female | 15 to 21 | 0 | #NUM! | 0 | Baseline |
| Male | 22 to 35 | 0 | #NUM! | 0 | Baseline |
| Male | 15 to 21 | 0 | #NUM! | 0 | Baseline |
| Female | 5 to 14 | 0 | #NUM! | 0 | Baseline |
| Male | 5 to 14 | 0 | #NUM! | 0 | Baseline |
| Male | 5 to 14 | 0 | #NUM! | 0 | Baseline |
| Female | 5 to 14 | 0 | #NUM! | 0 | Baseline |
| Female | 15 to 21 | 0 | #NUM! | 0 | Baseline |

| Male | 15 to 21 | 0 | #NUM! | 0 | Baseline |
| --- | --- | --- | --- | --- | --- |
| Male | 5 to 14 | 0 | #NUM! | 0 | Baseline |
| Female | 22 to 35 | 0 | #NUM! | 0 | Baseline |
| Female | 22 to 35 | 0 | #NUM! | 0 | Baseline |
| Female | 22 to 35 | 0 | #NUM! | 0 | Baseline |
| Male | 5 to 14 | 0 | #NUM! | 0 | Baseline |
| Male | 22 to 35 | 0 | #NUM! | 0 | Baseline |
| Female | 15 to 21 | 0 | #NUM! | 0 | Baseline |
| Female | 5 to 14 | 0 | #NUM! | 0 | Baseline |
| Female | 35+ | 0 | #NUM! | 0 | Baseline |
| Female | 5 to 14 | 0 | #NUM! | 0 | Baseline |
| Female | 35+ | 0 | #NUM! | 0 | Baseline |
| Female | 22 to 35 | 0 | #NUM! | 0 | Baseline |
| Male | 5 to 14 | 0 | #NUM! | 0 | Baseline |
| Male | 5 to 14 | 0 | #NUM! | 0 | Baseline |
| Female | 5 to 14 | 0 | #NUM! | 0 | Baseline |
| Male | 1 to 4 | 0 | #NUM! | 0 | Baseline |
| Male | 5 to 14 | 0 | #NUM! | 0 | Baseline |
| Male | 1 to 4 | 0 | #NUM! | 0 | Baseline |
| Male | 5 to 14 | 0 | #NUM! | 0 | Baseline |
| Female | 5 to 14 | 0 | #NUM! | 0 | Baseline |
| Female | 15 to 21 | 0 | #NUM! | 0 | Baseline |
| Female | 15 to 21 | 0 | #NUM! | 0 | Baseline |
| Male | 15 to 21 | 0 | #NUM! | 0 | Baseline |
| Female | 5 to 14 | 0 | #NUM! | 0 | Baseline |
| Female | 15 to 21 | 0 | #NUM! | 0 | Baseline |
| Female | 5 to 14 | 0 | #NUM! | 0 | Baseline |
| Male | 15 to 21 | 0 | #NUM! | 0 | Baseline |
| Male | 5 to 14 | 0 | #NUM! | 0 | Baseline |
| Male | 15 to 21 | 0 | #NUM! | 0 | Baseline |
| Male | 5 to 14 | 0 | #NUM! | 0 | Baseline |
| Female | 5 to 14 | 0 | #NUM! | 0 | Baseline |
| Male | 5 to 14 | 0 | #NUM! | 0 | Baseline |
| Female | 15 to 21 | 0 | #NUM! | 0 | Baseline |
| Female | 15 to 21 | 0 | #NUM! | 0 | Baseline |
| Female | 5 to 14 | 0 | #NUM! | 0 | Baseline |
| Male | 15 to 21 | 0 | #NUM! | 0 | Baseline |
| Male | 15 to 21 | 0 | #NUM! | 0 | Baseline |
| Female | 15 to 21 | 0 | #NUM! | 0 | Baseline |
| Male | 15 to 21 | 0 | #NUM! | 0 | Baseline |
| Female | 22 to 35 | 0 | #NUM! | 0 | Baseline |
| Male | 22 to 35 | 0 | #NUM! | 0 | Baseline |
| Male | 35+ | 0 | #NUM! | 0 | Baseline |
| Female | 35+ | 0 | #NUM! | 0 | Baseline |
| Female | 35+ | 0 | #NUM! | 0 | Baseline |
| Male | 22 to 35 | 0 | #NUM! | 0 | Baseline |
| Male | 35+ | 0 | #NUM! | 0 | Baseline |
| Female | 22 to 35 | 5 | 0 | 0 | Baseline |
| Female | 35+ | 1 | 0 | 0 | Baseline |
| Female | 22 to 35 | 0 | #NUM! | 0 | Baseline |

| Male | 35+ | 1 | 0 | 0 | Baseline |
| --- | --- | --- | --- | --- | --- |
| Female | 35+ | 0 | #NUM! | 0 | Baseline |
| Male | 22 to 35 | 0 | #NUM! | 0 | Baseline |
| Male | 5 to 14 | 0 | #NUM! | 0 | Baseline |
| Female | 5 to 14 | 0 | #NUM! | 0 | Baseline |
| Female | 5 to 14 | 0 | #NUM! | 0 | Baseline |
| Male | 1 to 4 | 2 | 0 | 0 | Baseline |
| Male | 5 to 14 | 2 | 0 | 0 | Baseline |
| Female | 1 to 4 | 1 | 0 | 0 | Baseline |
| Male | 5 to 14 | 0 | #NUM! | 0 | Baseline |
| Female | 5 to 14 | 0 | #NUM! | 0 | Baseline |
| Female | 5 to 14 | 0 | #NUM! | 0 | Baseline |
| Male | 5 to 14 | 0 | #NUM! | 0 | Baseline |
| Male | 5 to 14 | 0 | #NUM! | 0 | Baseline |
| Female | 15 to 21 | 0 | #NUM! | 0 | Baseline |
| Male | 5 to 14 | 0 | #NUM! | 0 | Baseline |
| Female | 15 to 21 | 0 | #NUM! | 0 | Baseline |
| Male | 1 to 4 | 0 | #NUM! | 0 | Baseline |
| Female | 35+ | 0 | #NUM! | 0 | Baseline |
| Male | 5 to 14 | 0 | #NUM! | 0 | Baseline |
| Male | 15 to 21 | 0 | #NUM! | 0 | Baseline |
| Male | 1 to 4 | 0 | #NUM! | 0 | Baseline |
| Female | 5 to 14 | 0 | #NUM! | 0 | Baseline |
| Female | 5 to 14 | 0 | #NUM! | 0 | Baseline |
| Female | 15 to 21 | 0 | #NUM! | 0 | Baseline |
| Female | 5 to 14 | 0 | #NUM! | 0 | Baseline |
| Female | 5 to 14 | 0 | #NUM! | 0 | Baseline |
| Female | 15 to 21 | 0 | #NUM! | 0 | Baseline |
| Female | 22 to 35 | 0 | #NUM! | 0 | Baseline |
| Male | 35+ | 0 | #NUM! | 0 | Baseline |
| Female | 22 to 35 | 0 | #NUM! | 0 | Baseline |
| Male | 35+ | 0 | #NUM! | 0 | Baseline |
| Female | 22 to 35 | 0 | #NUM! | 0 | Baseline |
| Female | 35+ | 0 | #NUM! | 0 | Baseline |
| Female | 22 to 35 | 0 | #NUM! | 0 | Baseline |
| Female | 22 to 35 | 4 | 0 | 0 | Baseline |
| Female | 22 to 35 | 0 | #NUM! | 0 | Baseline |
| Male | 35+ | 0 | #NUM! | 0 | Baseline |
| Male | 22 to 35 | 0 | #NUM! | 0 | Baseline |
| Female | 22 to 35 | 0 | #NUM! | 0 | Baseline |
| Male | 22 to 35 | 0 | #NUM! | 0 | Baseline |
| Female | 35+ | 0 | #NUM! | 0 | Baseline |
| Female | 35+ | 0 | #NUM! | 0 | Baseline |
| Female | 35+ | 0 | #NUM! | 0 | Baseline |
| Female | 35+ | 0 | #NUM! | 0 | Baseline |
| Female | 35+ | 0 | #NUM! | 0 | Baseline |
| Female | 15 to 21 | 0 | #NUM! | 0 | Baseline |
| Male | 1 to 4 | 0 | #NUM! | 0 | Baseline |
| Male | 35+ | 0 | #NUM! | 0 | Baseline |
| Female | 35+ | 0 | #NUM! | 0 | Baseline |

| Male | 35+ | 0 | #NUM! | 0 | Baseline |
| --- | --- | --- | --- | --- | --- |
| Female | 35+ | 0 | #NUM! | 0 | Baseline |
| Male | 15 to 21 | 0 | #NUM! | 0 | Baseline |
| Female | 22 to 35 | 0 | #NUM! | 0 | Baseline |
| Female | 22 to 35 | 0 | #NUM! | 0 | Baseline |
| Female | 22 to 35 | 0 | #NUM! | 0 | Baseline |
| Male | 22 to 35 | 5 | #NUM! | 0 | Baseline |
| Male | 22 to 35 | 0 | #NUM! | 0 | Baseline |
| Male | 22 to 35 | 0 | #NUM! | 0 | Baseline |
| Male | 35+ | 3 | #NUM! | 0 | Baseline |
| Male | 35+ | 2 | #NUM! | 0 | Baseline |
| Female | 35+ | 0 | #NUM! | 0 | Baseline |
| Female | 5 to 14 | 0 | #NUM! | 0 | Baseline |
| Female | 5 to 14 | 0 | #NUM! | 0 | Baseline |
| Male | 1 to 4 | 0 | #NUM! | 0 | Baseline |
| Male | 5 to 14 | 0 | #NUM! | 0 | Baseline |
| Male | 5 to 14 |  | #NUM! | 0 | Baseline |
| Male | 1 to 4 |  | #NUM! | 0 | Baseline |
| Female | 5 to 14 |  | #NUM! | 0 | Baseline |
| Female | 5 to 14 |  | #NUM! | 0 | Baseline |
| Female | 5 to 14 |  | #NUM! | 0 | Baseline |
| Female | 5 to 14 |  | #NUM! | 0 | Baseline |
| Male | 5 to 14 |  | #NUM! | 0 | Baseline |
| Male | 5 to 14 |  | #NUM! | 0 | Baseline |
| Female | 5 to 14 |  | #NUM! | 0 | Baseline |
| Male | 5 to 14 |  | #NUM! | 0 | Baseline |
| Male | 5 to 14 |  | #NUM! | 0 | Baseline |
| Male | 35+ |  | #NUM! | 0 | Baseline |
| Female | 15 to 21 |  | #NUM! | 0 | Baseline |
| Male | 35+ |  | #NUM! | 0 | Baseline |
| Female | 22 to 35 | 0 | #NUM! | 0 | Baseline |
| Male | 15 to 21 | 0 | #NUM! | 0 | Baseline |
| Male | 15 to 21 | 1 | #NUM! | 0 | Baseline |
| Male | 1 to 4 |  | #NUM! | 0 | Baseline |
| Female | 35+ | 0 | #NUM! | 0 | Baseline |
| Male | 15 to 21 |  | #NUM! | 0 | Baseline |
| Male | 15 to 21 |  | #NUM! | 0 | Baseline |
| Male | 22 to 35 |  | #NUM! | 0 | Baseline |
| Male | 22 to 35 |  | #NUM! | 0 | Baseline |
| Male | 22 to 35 |  | #NUM! | 0 | Baseline |
| Female | 15 to 21 |  | #NUM! | 0 | Baseline |
| Female | 15 to 21 |  | #NUM! | 0 | Baseline |
| Female | 15 to 21 |  | #NUM! | 0 | Baseline |
| Male | 22 to 35 |  | #NUM! | 0 | Baseline |
| Male | 22 to 35 |  | #NUM! | 0 | Baseline |
| Male | 15 to 21 |  | #NUM! | 0 | Baseline |
| Female | 15 to 21 |  | #NUM! | 0 | Baseline |
| Female | 15 to 21 |  | #NUM! | 0 | Baseline |
| Female | 5 to 14 |  | #NUM! | 0 | Baseline |
| Male | 5 to 14 |  | #NUM! | 0 | Baseline |

| Female | 1 to 4 |  | #NUM! | 0 | Baseline |
| --- | --- | --- | --- | --- | --- |
| Male | 5 to 14 |  | #NUM! | 0 | Baseline |
| Female | 5 to 14 |  | #NUM! | 0 | Baseline |
| Male | 35+ | 0 | #NUM! | 0 | Baseline |
| Female | 22 to 35 | 0 | #NUM! | 0 | Baseline |
| Female | 5 to 14 | 0 | #NUM! | 0 | Baseline |
| Male | 1 to 4 | 0 | #NUM! | 0 | Baseline |
| Male | 35+ | 0 | #NUM! | 0 | Baseline |
| Male | 35+ | 0 | #NUM! | 0 | Baseline |
| Male | 5 to 14 | 0 | #NUM! | 0 | Baseline |
| Female | 22 to 35 | 0 | #NUM! | 0 | Baseline |
| Female | 22 to 35 | 0 | #NUM! | 0 | Baseline |
| Male | 5 to 14 | 0 | #NUM! | 0 | Baseline |
| Male | 35+ | 0 | #NUM! | 0 | Baseline |
| Female | 15 to 21 | 0 | #NUM! | 0 | Baseline |
| Female | 5 to 14 | 0 | #NUM! | 0 | Baseline |
| Male | 35+ | 0 | #NUM! | 0 | Baseline |
| Female | 1 to 4 | 0 | #NUM! | 0 | Baseline |
| Female | 1 to 4 | 0 | #NUM! | 0 | Baseline |
| Male | 1 to 4 | 0 | #NUM! | 0 | Baseline |
| Male | 22 to 35 | 0 | #NUM! | 0 | Baseline |
| Female | 5 to 14 | 0 | #NUM! | 0 | Baseline |
| Female | 15 to 21 | 0 | #NUM! | 0 | Baseline |
| Female | 15 to 21 | 0 | #NUM! | 0 | Baseline |
| Female | 15 to 21 | 0 | #NUM! | 0 | Baseline |
| Male | 22 to 35 | 0 | #NUM! | 0 | Baseline |
| Male | 22 to 35 | 0 | #NUM! | 0 | Baseline |
| Male | 22 to 35 | 0 | #NUM! | 0 | Baseline |
| Female | 15 to 21 | 1 | 0 | 0 | Baseline |
| Male | 22 to 35 | 0 | #NUM! | 0 | Baseline |
| Male | 22 to 35 | 0 | #NUM! | 0 | Baseline |
| Male | 22 to 35 | 0 | #NUM! | 0 | Baseline |
| Female | 5 to 14 | 0 | #NUM! | 0 | Baseline |
| Female | 5 to 14 | 0 | #NUM! | 0 | Baseline |
| Male | 22 to 35 | 0 | #NUM! | 0 | Baseline |
| Male | 22 to 35 | 0 | #NUM! | 0 | Baseline |
| Male | 15 to 21 | 0 | #NUM! | 0 | Baseline |
| Male | 15 to 21 | 0 | #NUM! | 0 | Baseline |
| Male | 15 to 21 | 0 | #NUM! | 0 | Baseline |
| Male | 15 to 21 | 0 | #NUM! | 0 | Baseline |
| Male | 15 to 21 | 0 | #NUM! | 0 | Baseline |
| Female | 5 to 14 | 0 | #NUM! | 0 | Baseline |
| Male | 35+ | 0 | #NUM! | 0 | Baseline |
| Male | 15 to 21 | 0 | #NUM! | 0 | Baseline |
| Female | 15 to 21 | 0 | #NUM! | 0 | Baseline |
| Female | 15 to 21 | 0 | #NUM! | 0 | Baseline |
| Female | 15 to 21 | 0 | #NUM! | 0 | Baseline |
| Female | 5 to 14 | 0 | #NUM! | 0 | Baseline |
| Male | 5 to 14 | 0 | #NUM! | 0 | Baseline |
| Male | 5 to 14 | 0 | #NUM! | 0 | Baseline |

| Male | 5 to 14 | 0 | #NUM! | 0 | Baseline |
| --- | --- | --- | --- | --- | --- |
| Male | 5 to 14 | 0 | #NUM! | 0 | Baseline |
| Female | 5 to 14 | 0 | #NUM! | 0 | Baseline |
| Female | 5 to 14 | 1 | 0 | 0 | Baseline |
| Male | 5 to 14 | 0 | #NUM! | 0 | Baseline |
| Male | 15 to 21 | 0 | #NUM! | 0 | Baseline |
| Female | 5 to 14 | 0 | #NUM! | 0 | Baseline |
| Female | 5 to 14 | 0 | #NUM! | 0 | Baseline |
| Male | 5 to 14 | 0 | #NUM! | 0 | Baseline |
| Female | 5 to 14 | 0 | #NUM! | 0 | Baseline |
| Male | 5 to 14 | 0 | #NUM! | 0 | Baseline |
| Male | 15 to 21 | 0 | #NUM! | 0 | Baseline |
| Female | 22 to 35 | 0 | #NUM! | 0 | Baseline |
| Female | 35+ | 0 | #NUM! | 0 | Baseline |
| Male | 1 to 4 | 0 | #NUM! | 0 | Baseline |
| Male | 35+ | 0 | #NUM! | 0 | Baseline |
| Female | 22 to 35 | 0 | #NUM! | 0 | Baseline |
| Female | 35+ | 0 | #NUM! | 0 | Baseline |
| Female | 35+ | 0 | #NUM! | 0 | Baseline |
| Male | 35+ | 0 | #NUM! | 0 | Baseline |
| Male | 35+ | 0 | #NUM! | 0 | Baseline |
| Female | 35+ | 0 | #NUM! | 0 | Baseline |
| Male | 35+ | 0 | #NUM! | 0 | Baseline |
| Male | 35+ | 0 | #NUM! | 0 | Baseline |
| Male | 5 to 14 | 0 | #NUM! | 0 | Baseline |
| Female | 35+ | 0 | #NUM! | 0 | Baseline |
| Male | 1 to 4 | 0 | #NUM! | 0 | Baseline |
| Male | 1 to 4 | 0 | #NUM! | 0 | Baseline |
| Female | 35+ | 0 | #NUM! | 0 | Baseline |
| Female | 35+ | 0 | #NUM! | 0 | Baseline |
| Male | 5 to 14 | 0 | #NUM! | 0 | Baseline |
| Male | 5 to 14 | 0 | #NUM! | 0 | Baseline |
| Male | 35+ | 0 | #NUM! | 0 | Baseline |
| Female | 35+ | 0 | #NUM! | 0 | Baseline |
| Female | 35+ | 0 | #NUM! | 0 | Baseline |
| Female | 35+ | 0 | #NUM! | 0 | Baseline |
| Male | 1 to 4 | 0 | #NUM! | 0 | Baseline |
| Female | 35+ | 0 | #NUM! | 0 | Baseline |
| Female | 35+ | 0 | #NUM! | 0 | Baseline |
| Female | 35+ | 0 | #NUM! | 0 | Baseline |
| Female | 35+ | 5 | 0 | 0 | Baseline |
| Male | 35+ | 0 | #NUM! | 0 | Baseline |
| Male | 35+ | 0 | #NUM! | 0 | Baseline |
| Female | 35+ | 0 | #NUM! | 0 | Baseline |
| Male | 35+ | 0 | #NUM! | 0 | Baseline |
| Male | 5 to 14 | 0 | #NUM! | 0 | Baseline |
| Male | 5 to 14 | 0 | #NUM! | 0 | Baseline |
| Male | 1 to 4 | 0 | #NUM! | 0 | Baseline |
| Male | 5 to 14 | 0 | #NUM! | 0 | Baseline |
| Male | 5 to 14 | 0 | #NUM! | 0 | Baseline |

| Male | 5 to 14 | 0 | #NUM! | 0 | Baseline |
| --- | --- | --- | --- | --- | --- |
| Male | 5 to 14 | 0 | #NUM! | 0 | Baseline |
| Male | 5 to 14 | 0 | #NUM! | 0 | Baseline |
| Female | 5 to 14 | 0 | #NUM! | 0 | Baseline |
| Female | 5 to 14 | 0 | #NUM! | 0 | Baseline |
| Female | 5 to 14 | 0 | #NUM! | 0 | Baseline |
| Female | 5 to 14 | 0 | #NUM! | 0 | Baseline |
| Female | 5 to 14 | 0 | #NUM! | 0 | Baseline |
| Female | 5 to 14 | 0 | #NUM! | 0 | Baseline |
| Female | 5 to 14 | 0 | #NUM! | 0 | Baseline |
| Female | 5 to 14 | 0 | #NUM! | 0 | Baseline |
| Female | 5 to 14 | 0 | #NUM! | 0 | Baseline |
| Female | 5 to 14 | 0 | #NUM! | 0 | Baseline |
| Female | 5 to 14 | 0 | #NUM! | 0 | Baseline |
| Male | 5 to 14 | 0 | #NUM! | 0 | Baseline |
| Female | 5 to 14 | 0 | #NUM! | 0 | Baseline |
| Male | 5 to 14 | 0 | #NUM! | 0 | Baseline |
| Male | 5 to 14 | 0 | #NUM! | 0 | Baseline |
| Male | 5 to 14 | 0 | #NUM! | 0 | Baseline |
| Male | 5 to 14 | 0 | #NUM! | 0 | Baseline |
| Male | 1 to 4 | 0 | #NUM! | 0 | Baseline |
| Female | 5 to 14 | 0 | #NUM! | 0 | Baseline |
| Female | 5 to 14 | 0 | #NUM! | 0 | Baseline |
| Female | 5 to 14 | 0 | #NUM! | 0 | Baseline |
| Male | 15 to 21 | 0 | #NUM! | 0 | Baseline |
| Male | 15 to 21 | 0 | #NUM! | 0 | Baseline |
| Male | 15 to 21 | 0 | #NUM! | 0 | Baseline |
| Male | 15 to 21 | 0 | #NUM! | 0 | Baseline |
| Female | 15 to 21 | 0 | #NUM! | 0 | Baseline |
| Female | 15 to 21 | 0 | #NUM! | 0 | Baseline |
| Female | 15 to 21 | 5 | 0 | 0 | Baseline |
| Female | 15 to 21 | 0 | #NUM! | 0 | Baseline |
| Female | 15 to 21 | 0 | #NUM! | 0 | Baseline |
| Male | 22 to 35 | 0 | #NUM! | 0 | Baseline |
| Male | 15 to 21 | 0 | #NUM! | 0 | Baseline |
| Male | 15 to 21 | 0 | #NUM! | 0 | Baseline |
| Male | 15 to 21 | 0 | #NUM! | 0 | Baseline |
| Female | 15 to 21 | 0 | #NUM! | 0 | Baseline |
| Female | 15 to 21 | 0 | #NUM! | 0 | Baseline |
| Male | 15 to 21 | 0 | #NUM! | 0 | Baseline |
| Male | 15 to 21 | 0 | #NUM! | 0 | Baseline |
| Male | 22 to 35 | 0 | #NUM! | 0 | Baseline |
| Female | 22 to 35 | 0 | #NUM! | 0 | Baseline |
| Female | 22 to 35 | 0 | #NUM! | 0 | Baseline |
| Female | 22 to 35 | 0 | #NUM! | 0 | Baseline |
| Female | 22 to 35 | 0 | #NUM! | 0 | Baseline |
| Female | 22 to 35 | 0 | #NUM! | 0 | Baseline |
| Male | 22 to 35 | 0 | #NUM! | 0 | Baseline |
| Male | 22 to 35 | 0 | #NUM! | 0 | Baseline |
| Male | 1 to 4 | 0 | #NUM! | 0 | Baseline |

| Male | 1 to 4 | 0 | #NUM! | 0 | Baseline |
| --- | --- | --- | --- | --- | --- |
| Male | 1 to 4 | 0 | #NUM! | 0 | Baseline |
| Male | 1 to 4 | 0 | #NUM! | 0 | Baseline |
| Male | 1 to 4 | 0 | #NUM! | 0 | Baseline |
| Male | 1 to 4 | 0 | #NUM! | 0 | Baseline |
| Male | 1 to 4 | 0 | #NUM! | 0 | Baseline |
| Male | 1 to 4 | 0 | #NUM! | 0 | Baseline |
| Male | 1 to 4 | 0 | #NUM! | 0 | Baseline |
| Male | 1 to 4 | 0 | #NUM! | 0 | Baseline |
| Male | 1 to 4 | 0 | #NUM! | 0 | Baseline |
| Male | 1 to 4 | 0 | #NUM! | 0 | Baseline |
| Male | 1 to 4 | 0 | #NUM! | 0 | Baseline |
| Female | 22 to 35 | 0 | #NUM! | 0 | Baseline |
| Male | 5 to 14 | 0 | #NUM! | 0 | Baseline |
| Female | 5 to 14 | 0 | #NUM! | 0 | Baseline |
| Female | 35+ | 0 | #NUM! | 0 | Baseline |
| Female | 22 to 35 | 0 | #NUM! | 0 | Baseline |
| Female | 22 to 35 | 0 | #NUM! | 0 | Baseline |
| Female | 35+ | 0 | #NUM! | 0 | Baseline |
| Female | 22 to 35 | 0 | #NUM! | 0 | Baseline |
| Male | 1 to 4 | 0 | #NUM! | 0 | Baseline |
| Male | 22 to 35 | 0 | #NUM! | 0 | Baseline |
| Male | 5 to 14 | 0 | #NUM! | 0 | Baseline |
| Female | 5 to 14 | 0 | #NUM! | 0 | Baseline |
| Male | 35+ | 0 | #NUM! | 0 | Baseline |
| Male | 1 to 4 | 0 | #NUM! | 0 | Baseline |
| Female | 22 to 35 | 0 | #NUM! | 0 | Baseline |
| Male | 1 to 4 | 0 | #NUM! | 0 | Baseline |
| Male | 5 to 14 | 0 | #NUM! | 0 | Baseline |
| Female | 22 to 35 | 0 | #NUM! | 0 | Baseline |
| Female | 15 to 21 | 0 | #NUM! | 0 | Baseline |
| Female | 1 to 4 | 0 | #NUM! | 0 | Baseline |
| Female | 22 to 35 | 0 | #NUM! | 0 | Baseline |
| Female | 22 to 35 | 0 | #NUM! | 0 | Baseline |
| Male | 5 to 14 | 0 | #NUM! | 0 | Baseline |
| Female | 22 to 35 | 0 | #NUM! | 0 | Baseline |
| Female | 22 to 35 | 0 | #NUM! | 0 | Baseline |
| Female | 35+ | 0 | #NUM! | 0 | Baseline |
| Male | 1 to 4 | 0 | #NUM! | 0 | Baseline |
| Male | 1 to 4 | 0 | #NUM! | 0 | Baseline |
| Male | 1 to 4 | 0 | #NUM! | 0 | Baseline |
| Male | 5 to 14 | 0 | #NUM! | 0 | Baseline |
| Female | 5 to 14 | 0 | #NUM! | 0 | Baseline |
| Female | 22 to 35 | 0 | #NUM! | 0 | Baseline |
| Female | 22 to 35 | 0 | #NUM! | 0 | Baseline |
| Male | 15 to 21 | 0 | #NUM! | 0 | Baseline |
| Male | 22 to 35 | 0 | #NUM! | 0 | Baseline |
| Male | 5 to 14 | 0 | #NUM! | 0 | Baseline |
| Male | 1 to 4 | 0 | #NUM! | 0 | Baseline |
| Male | 5 to 14 | 0 | #NUM! | 0 | Baseline |

| Male | 1 to 4 | 0 | #NUM! | 0 | Baseline |
| --- | --- | --- | --- | --- | --- |
| Male | 1 to 4 | 0 | #NUM! | 0 | Baseline |
| Male | 35+ | 0 | #NUM! | 0 | Baseline |
| Female | 22 to 35 | 0 | #NUM! | 0 | Baseline |
| Female | 22 to 35 | 0 | #NUM! | 0 | Baseline |
| Female | 5 to 14 | 0 | #NUM! | 0 | Baseline |
| Female | 22 to 35 | 0 | #NUM! | 0 | Baseline |
| Male | 35+ | 0 | #NUM! | 0 | Baseline |
| Female | 22 to 35 | 0 | #NUM! | 0 | Baseline |
| Female | 35+ | 0 | #NUM! | 0 | Baseline |
| Male | 35+ | 0 | #NUM! | 0 | Baseline |
| Female | 22 to 35 | 0 | #NUM! | 0 | Baseline |
| Female | 22 to 35 | 0 | #NUM! | 0 | Baseline |
| Female | 35+ | 0 | #NUM! | 0 | Baseline |
| Male | 22 to 35 | 0 | #NUM! | 0 | Baseline |
| Female | 22 to 35 | 0 | #NUM! | 0 | Baseline |
| Male | 15 to 21 | 0 | #NUM! | 0 | Baseline |
| Female | 22 to 35 | 0 | #NUM! | 0 | Baseline |
| Female | 35+ | 0 | #NUM! | 0 | Baseline |
| Female | 5 to 14 | 0 | #NUM! | 0 | Baseline |
| Female | 5 to 14 | 0 | #NUM! | 0 | Baseline |
| Male | 5 to 14 | 0 | #NUM! | 0 | Baseline |
| Female | 35+ | 0 | #NUM! | 0 | Baseline |
| Female | 22 to 35 | 0 | #NUM! | 0 | Baseline |
| Female | 15 to 21 | 0 | #NUM! | 0 | Baseline |
| Female | 22 to 35 | 0 | #NUM! | 0 | Baseline |
| Male | 35+ | 0 | #NUM! | 0 | Baseline |
| Male | 35+ | 0 | #NUM! | 0 | Baseline |
| Male | 5 to 14 | 0 | #NUM! | 0 | Baseline |
| Female | 15 to 21 | 0 | #NUM! | 0 | Baseline |
| Female | 5 to 14 | 0 | #NUM! | 0 | Baseline |
| Male | 5 to 14 | 0 | #NUM! | 0 | Baseline |
| Male | 5 to 14 | 0 | #NUM! | 0 | Baseline |
| Female | 5 to 14 | 0 | #NUM! | 0 | Baseline |
| Male | 35+ | 0 | #NUM! | 0 | Baseline |
| Male | 5 to 14 | 0 | #NUM! | 0 | Baseline |
| Female | 5 to 14 | 0 | #NUM! | 0 | Baseline |
| Male | 5 to 14 | 0 | #NUM! | 0 | Baseline |
| Male | 5 to 14 | 0 | #NUM! | 0 | Baseline |
| Male | 35+ | 0 | #NUM! | 0 | Baseline |
| Female | 35+ | 0 | #NUM! | 0 | Baseline |
| Female | 22 to 35 |  | #NUM! | 0 | Baseline |
| Male | 35+ | 0 | #NUM! | 0 | Baseline |
| Female | 35+ | 0 | #NUM! | 0 | Baseline |
| Female | 22 to 35 | 0 | #NUM! | 0 | Baseline |
| Male | 35+ | 0 | #NUM! | 0 | Baseline |
| Female | 35+ | 0 | #NUM! | 0 | Baseline |
| Female | 5 to 14 | 0 | #NUM! | 0 | Baseline |
| Female | 15 to 21 | 0 | #NUM! | 0 | Baseline |
| Female | 22 to 35 | 0 | #NUM! | 0 | Baseline |

| Female | 22 to 35 | 0 | #NUM! | 0 | Baseline |
| --- | --- | --- | --- | --- | --- |
| Male | 1 to 4 | 0 | #NUM! | 0 | Baseline |
| Female | 15 to 21 | 0 | #NUM! | 0 | Baseline |
| Female | 15 to 21 | 0 | #NUM! | 0 | Baseline |
| Male | 35+ | 0 | #NUM! | 0 | Baseline |
| Female | 22 to 35 | 0 | #NUM! | 0 | Baseline |
| Male | 22 to 35 | 0 | #NUM! | 0 | Baseline |
| Female | 22 to 35 | 0 | #NUM! | 0 | Baseline |
| Female | 22 to 35 | 0 | #NUM! | 0 | Baseline |
| Female | 5 to 14 | 0 | #NUM! | 0 | Baseline |
| Male | 5 to 14 | 0 | #NUM! | 0 | Baseline |
| Male | 5 to 14 | 0 | #NUM! | 0 | Baseline |
| Female | 5 to 14 | 0 | #NUM! | 0 | Baseline |
| Female | 5 to 14 | 0 | #NUM! | 0 | Baseline |
| Male | 22 to 35 | 0 | #NUM! | 0 | Baseline |
| Male | 5 to 14 | 0 | #NUM! | 0 | Baseline |
| Male | 5 to 14 | 0 | #NUM! | 0 | Baseline |
| Female | 5 to 14 | 0 | #NUM! | 0 | Baseline |
| Male | 5 to 14 | 0 | #NUM! | 0 | Baseline |
| Female | 5 to 14 | 0 | #NUM! | 0 | Baseline |
| Male | 5 to 14 | 0 | #NUM! | 0 | Baseline |
| Female | 5 to 14 | 0 | #NUM! | 0 | Baseline |
| Male | 5 to 14 | 0 | #NUM! | 0 | Baseline |
| Female | 15 to 21 | 0 | #NUM! | 0 | Baseline |
| Female | 5 to 14 | 0 | #NUM! | 0 | Baseline |
| Male | 5 to 14 | 0 | #NUM! | 0 | Baseline |
| Male | 5 to 14 | 0 | #NUM! | 0 | Baseline |
| Male | 5 to 14 | 0 | #NUM! | 0 | Baseline |
| Male | 5 to 14 | 0 | #NUM! | 0 | Baseline |
| Male | 5 to 14 | 0 | #NUM! | 0 | Baseline |
| Male | 5 to 14 | 0 | #NUM! | 0 | Baseline |
| Male | 5 to 14 | 0 | #NUM! | 0 | Baseline |
| Male | 22 to 35 | 0 | #NUM! | 0 | Baseline |
| Female | 35+ | 0 | #NUM! | 0 | Baseline |
| Female | 22 to 35 | 0 | #NUM! | 0 | Baseline |
| Female | 22 to 35 | 0 | #NUM! | 0 | Baseline |
| Female | 22 to 35 | 0 | #NUM! | 0 | Baseline |
| Female | 35+ | 0 | #NUM! | 0 | Baseline |
| Female | 22 to 35 | 0 | #NUM! | 0 | Baseline |
| Female | 22 to 35 | 0 | #NUM! | 0 | Baseline |
| Male | 1 to 4 | 0 | #NUM! | 0 | Baseline |
| Female | 22 to 35 | 0 | #NUM! | 0 | Baseline |
| Female | 35+ | 0 | #NUM! | 0 | Baseline |
| Female | 15 to 21 | 0 | #NUM! | 0 | Baseline |
| Female | 15 to 21 | 0 | #NUM! | 0 | Baseline |
| Female | 22 to 35 | 0 | #NUM! | 0 | Baseline |
| Female | 5 to 14 | 0 | #NUM! | 0 | Baseline |
| Female | 22 to 35 | 0 | #NUM! | 0 | Baseline |
| Male | 1 to 4 | 0 | #NUM! | 0 | Baseline |
| Female | 22 to 35 | 0 | #NUM! | 0 | Baseline |

| Female | 22 to 35 | 0 | #NUM! | 0 | Baseline |
| --- | --- | --- | --- | --- | --- |
| Female | 15 to 21 | 0 | #NUM! | 0 | Baseline |
| Male | 35+ | 0 | #NUM! | 0 | Baseline |
| Female | 22 to 35 | 0 | #NUM! | 0 | Baseline |
| Male | 5 to 14 | 0 | #NUM! | 0 | Baseline |
| Female | 5 to 14 | 0 | #NUM! | 0 | Baseline |
| Female | 35+ | 0 | #NUM! | 0 | Baseline |
| Male | 5 to 14 | 0 | #NUM! | 0 | Baseline |
| Female | 5 to 14 | 0 | #NUM! | 0 | Baseline |
| Female | 35+ | 0 | #NUM! | 0 | Baseline |
| Male | 1 to 4 | 0 | #NUM! | 0 | Baseline |
| Male | 5 to 14 | 0 | #NUM! | 0 | Baseline |
| Male | 22 to 35 | 0 | #NUM! | 0 | Baseline |
| Male | 15 to 21 | 3 | 0 | 0 | Baseline |
| Male | 35+ | 0 | #NUM! | 0 | Baseline |
| Female | 5 to 14 | 0 | #NUM! | 0 | Baseline |
| Female | 35+ | 0 | #NUM! | 0 | Baseline |
| Female | 35+ | 0 | #NUM! | 0 | Baseline |
| Female | 22 to 35 | 0 | #NUM! | 0 | Baseline |
| Female | 35+ | 0 | #NUM! | 0 | Baseline |
| Male | 1 to 4 | 5 | 0 | 0 | Baseline |
| Female | 35+ | 0 | #NUM! | 0 | Baseline |
| Female | 35+ | 0 | #NUM! | 0 | Baseline |
| Male | 22 to 35 | 0 | #NUM! | 0 | Baseline |
| Female | 22 to 35 | 0 | #NUM! | 0 | Baseline |
| Female | 22 to 35 | 0 | #NUM! | 0 | Baseline |
| Male | 5 to 14 | 0 | #NUM! | 0 | Baseline |
| Male | 35+ | 0 | #NUM! | 0 | Baseline |
| Female | 22 to 35 | 0 | #NUM! | 0 | Baseline |
| Male | 22 to 35 | 0 | #NUM! | 0 | Baseline |
| Female | 22 to 35 | 0 | #NUM! | 0 | Baseline |
| Female | 35+ | 0 | #NUM! | 0 | Baseline |
| Female | 35+ | 0 | #NUM! | 0 | Baseline |
| Female | 22 to 35 | 0 | #NUM! | 0 | Baseline |
| Female | 15 to 21 | 0 | #NUM! | 0 | Baseline |
| Female | 5 to 14 | 0 | #NUM! | 0 | Baseline |
| Male | 5 to 14 | 0 | #NUM! | 0 | Baseline |
| Male | 5 to 14 | 0 | #NUM! | 0 | Baseline |
| Female | 22 to 35 | 0 | #NUM! | 0 | Baseline |
| Female | 22 to 35 | 0 | #NUM! | 0 | Baseline |
| Female | 22 to 35 | 0 | #NUM! | 0 | Baseline |
| Female | 35+ | 0 | #NUM! | 0 | Baseline |
| Female | 35+ | 0 | #NUM! | 0 | Baseline |
| Female | 35+ | 0 | #NUM! | 0 | Baseline |
| Male | 35+ | 0 | #NUM! | 0 | Baseline |
| Male | 5 to 14 | 0 | #NUM! | 0 | Baseline |
| Male | 35+ | 0 | #NUM! | 0 | Baseline |
| Female | 22 to 35 | 0 | #NUM! | 0 | Baseline |
| Male | 22 to 35 | 0 | #NUM! | 0 | Baseline |
| Male | 15 to 21 | 0 | #NUM! | 0 | Baseline |

| Male | 5 to 14 | 0 | #NUM! | 0 | Baseline |
| --- | --- | --- | --- | --- | --- |
| Female | 35+ | 0 | #NUM! | 0 | Baseline |
| Male | 5 to 14 | 0 | #NUM! | 0 | Baseline |
| Female | 5 to 14 | 0 | #NUM! | 0 | Baseline |
| Female | 5 to 14 | 0 | #NUM! | 0 | Baseline |
| Male | 5 to 14 | 0 | #NUM! | 0 | Baseline |
| Male | 5 to 14 | 0 | #NUM! | 0 | Baseline |
| Female | 5 to 14 | 0 | #NUM! | 0 | Baseline |
| Male | 15 to 21 | 0 | #NUM! | 0 | Baseline |
| Male | 5 to 14 | 0 | #NUM! | 0 | Baseline |
| Male | 5 to 14 | 0 | #NUM! | 0 | Baseline |
| Female | 15 to 21 | 0 | #NUM! | 0 | Baseline |
| Female | 35+ | 0 | #NUM! | 0 | Baseline |
| Female | 35+ | 0 | #NUM! | 0 | Baseline |
| Female | 22 to 35 | 0 | #NUM! | 0 | Baseline |
| Female | 35+ | 0 | #NUM! | 0 | Baseline |
| Female | 22 to 35 | 0 | #NUM! | 0 | Baseline |
| Male | 5 to 14 | 0 | #NUM! | 0 | Baseline |
| Male | 5 to 14 | 0 | #NUM! | 0 | Baseline |
| Male | 15 to 21 | 0 | #NUM! | 0 | Baseline |
| Female | 5 to 14 | 0 | #NUM! | 0 | Baseline |
| Female | 22 to 35 | 0 | #NUM! | 0 | Baseline |
| Female | 5 to 14 | 0 | #NUM! | 0 | Baseline |
| Male | 15 to 21 | 0 | #NUM! | 0 | Baseline |
| Female | 15 to 21 | 0 | #NUM! | 0 | Baseline |
| Male | 5 to 14 | 0 | #NUM! | 0 | Baseline |
| Male | 15 to 21 | 0 | #NUM! | 0 | Baseline |
| Male | 15 to 21 | 0 | #NUM! | 0 | Baseline |
| Female | 15 to 21 | 0 | #NUM! | 0 | Baseline |
| Female | 35+ | 0 | #NUM! | 0 | Baseline |
| Male | 5 to 14 | 0 | #NUM! | 0 | Baseline |
| Male | 1 to 4 | 0 | #NUM! | 0 | Baseline |
| Female | 5 to 14 | 0 | #NUM! | 0 | Baseline |
| Female | 35+ | 0 | #NUM! | 0 | Baseline |
| Female | 22 to 35 | 0 | #NUM! | 0 | Baseline |
| Female | 1 to 4 | 0 | #NUM! | 0 | Baseline |
| Female | 22 to 35 | 0 | #NUM! | 0 | Baseline |
| Male | 1 to 4 | 0 | #NUM! | 0 | Baseline |
| Male | 5 to 14 | 0 | #NUM! | 0 | Baseline |
| Female | 5 to 14 | 0 | #NUM! | 0 | Baseline |
| Female | 35+ | 0 | #NUM! | 0 | Baseline |
| Female | 22 to 35 | 0 | #NUM! | 0 | Baseline |
| Male | 1 to 4 | 0 | #NUM! | 0 | Baseline |
| Female | 22 to 35 | 0 | #NUM! | 0 | Baseline |
| Female | 22 to 35 | 0 | #NUM! | 0 | Baseline |
| Female | 15 to 21 | 0 | #NUM! | 0 | Baseline |
| Female | 22 to 35 | 0 | #NUM! | 0 | Baseline |
| Female | 22 to 35 | 0 | #NUM! | 0 | Baseline |
| Male | 22 to 35 | 0 | #NUM! | 0 | Baseline |
| Female | 22 to 35 | 0 | #NUM! | 0 | Baseline |

| Female | 5 to 14 | 0 | #NUM! | 0 | Baseline |
| --- | --- | --- | --- | --- | --- |
| Female | 22 to 35 | 0 | #NUM! | 0 | Baseline |
| Male | 35+ | 0 | #NUM! | 0 | Baseline |
| Female | 35+ | 0 | #NUM! | 0 | Baseline |
| Female | 35+ | 0 | #NUM! | 0 | Baseline |
| Female | 22 to 35 | 0 | #NUM! | 0 | Baseline |
| Female | 35+ | 0 | #NUM! | 0 | Baseline |
| Female | 22 to 35 | 0 | #NUM! | 0 | Baseline |
| Female | 22 to 35 | 0 | #NUM! | 0 | Baseline |
| Female | 5 to 14 | 0 | #NUM! | 0 | Baseline |
| Female | 35+ | 0 | #NUM! | 0 | Baseline |
| Female | 35+ | 0 | #NUM! | 0 | Baseline |
| Female | 22 to 35 | 0 | #NUM! | 0 | Baseline |
| Female | 22 to 35 | 0 | #NUM! | 0 | Baseline |
| Female | 5 to 14 | 0 | #NUM! | 0 | Baseline |
| Female | 35+ | 0 | #NUM! | 0 | Baseline |
| Female | 22 to 35 | 0 | #NUM! | 0 | Baseline |
| Female | 5 to 14 | 0 | #NUM! | 0 | Baseline |
| Female | 22 to 35 | 0 | #NUM! | 0 | Baseline |
| Female | 5 to 14 | 0 | #NUM! | 0 | Baseline |
| Male | 15 to 21 | 0 | #NUM! | 0 | Baseline |
| Female | 22 to 35 | 0 | #NUM! | 0 | Baseline |
| Female | 22 to 35 | 0 | #NUM! | 0 | Baseline |
| Female | 22 to 35 | 0 | #NUM! | 0 | Baseline |
| Male | 5 to 14 | 0 | #NUM! | 0 | Baseline |
| Female | 22 to 35 | 0 | #NUM! | 0 | Baseline |
| Male | 15 to 21 | 0 | #NUM! | 0 | Baseline |
| Female | 22 to 35 | 0 | #NUM! | 0 | Baseline |
| Female | 22 to 35 | 0 | #NUM! | 0 | Baseline |
| Female | 22 to 35 | 0 | #NUM! | 0 | Baseline |
| Male | 22 to 35 | 0 | #NUM! | 0 | Baseline |
| Male | 5 to 14 | 0 | #NUM! | 0 | Baseline |
| Male | 5 to 14 | 0 | #NUM! | 0 | Baseline |
| Male | 35+ | 0 | #NUM! | 0 | Baseline |
| Male | 35+ | 0 | #NUM! | 0 | Baseline |
| Female | 22 to 35 | 0 | #NUM! | 0 | Baseline |
| Male | 15 to 21 | 0 | #NUM! | 0 | Baseline |
| Female | 5 to 14 | 0 | #NUM! | 0 | Baseline |
| Female | 5 to 14 | 0 | #NUM! | 0 | Baseline |
| Female | 5 to 14 | 0 | #NUM! | 0 | Baseline |
| Male | 5 to 14 | 0 | #NUM! | 0 | Baseline |
| Male | 5 to 14 | 0 | #NUM! | 0 | Baseline |
| Male | 15 to 21 | 0 | #NUM! | 0 | Baseline |
| Male | 5 to 14 | 0 | #NUM! | 0 | Baseline |
| Female | 15 to 21 | 0 | #NUM! | 0 | Baseline |
| Male | 5 to 14 | 0 | #NUM! | 0 | Baseline |
| Male | 5 to 14 | 0 | #NUM! | 0 | Baseline |
| Female | 15 to 21 | 0 | #NUM! | 0 | Baseline |
| Male | 15 to 21 | 0 | #NUM! | 0 | Baseline |
| Female | 15 to 21 | 0 | #NUM! | 0 | Baseline |

| Male | 5 to 14 | 0 | #NUM! | 0 | Baseline |
| --- | --- | --- | --- | --- | --- |
| Male | 15 to 21 | 0 | #NUM! | 0 | Baseline |
| Female | 5 to 14 | 0 | #NUM! | 0 | Baseline |
| Male | 5 to 14 | 0 | #NUM! | 0 | Baseline |
| Male | 5 to 14 | 0 | #NUM! | 0 | Baseline |
| Male | 22 to 35 | 0 | #NUM! | 0 | Baseline |
| Male | 15 to 21 | 0 | #NUM! | 0 | Baseline |
| Female | 5 to 14 | 0 | #NUM! | 0 | Baseline |
| Female | 5 to 14 | 0 | #NUM! | 0 | Baseline |
| Male | 15 to 21 | 0 | #NUM! | 0 | Baseline |
| Male | 35+ | 0 | #NUM! | 0 | Baseline |
| Male | 15 to 21 | 0 | #NUM! | 0 | Baseline |
| Female | 5 to 14 | 0 | #NUM! | 0 | Baseline |
| Male | 5 to 14 | 0 | #NUM! | 0 | Baseline |
| Female | 5 to 14 | 0 | #NUM! | 0 | Baseline |
| Female | 5 to 14 | 0 | #NUM! | 0 | Baseline |
| Female | 5 to 14 | 0 | #NUM! | 0 | Baseline |
| Female | 5 to 14 | 0 | #NUM! | 0 | Baseline |
| Male | 15 to 21 | 0 | #NUM! | 0 | Baseline |
| Female | 5 to 14 | 0 | #NUM! | 0 | Baseline |
| Male | 15 to 21 | 0 | #NUM! | 0 | Baseline |
| Female | 5 to 14 | 0 | #NUM! | 0 | Baseline |
| Male | 15 to 21 | 0 | #NUM! | 0 | Baseline |
| Male | 15 to 21 | 0 | #NUM! | 0 | Baseline |
| Female | 5 to 14 | 0 | #NUM! | 0 | Baseline |
| Male | 5 to 14 | 0 | #NUM! | 0 | Baseline |
| Female | 5 to 14 | 0 | #NUM! | 0 | Baseline |
| Male | 35+ | 0 | #NUM! | 0 | Baseline |
| Female | 22 to 35 | 0 | #NUM! | 0 | Baseline |
| Female | 5 to 14 | 0 | #NUM! | 0 | Baseline |
| Male | 22 to 35 | 0 | #NUM! | 0 | Baseline |
| Female | 22 to 35 | 0 | #NUM! | 0 | Baseline |
| Female | 5 to 14 | 0 | #NUM! | 0 | Baseline |
| Female | 22 to 35 | 0 | #NUM! | 0 | Baseline |
| Female | 5 to 14 | 0 | #NUM! | 0 | Baseline |
| Female | 22 to 35 | 0 | #NUM! | 0 | Baseline |
| Male | 22 to 35 | 0 | #NUM! | 0 | Baseline |
| Male | 22 to 35 | 0 | #NUM! | 0 | Baseline |
| Female | 15 to 21 | 0 | #NUM! | 0 | Baseline |
| Female | 22 to 35 | 0 | #NUM! | 0 | Baseline |
| Female | 22 to 35 | 0 | #NUM! | 0 | Baseline |
| Male | 35+ | 0 | #NUM! | 0 | Baseline |
| Female | 35+ | 0 | #NUM! | 0 | Baseline |
| Female | 15 to 21 | 0 | #NUM! | 0 | Baseline |
| Female | 35+ | 0 | #NUM! | 0 | Baseline |
| Male | 5 to 14 | 0 | #NUM! | 0 | Baseline |
| Female | 22 to 35 | 0 | #NUM! | 0 | Baseline |
| Male | 15 to 21 | 0 | #NUM! | 0 | Baseline |
| Female | 22 to 35 | 0 | #NUM! | 0 | Baseline |
| Female | 15 to 21 | 0 | #NUM! | 0 | Baseline |

| Female | 5 to 14 | 0 | #NUM! | 0 | Baseline |
| --- | --- | --- | --- | --- | --- |
| Female | 15 to 21 | 0 | #NUM! | 0 | Baseline |
| Male | 15 to 21 | 0 | #NUM! | 0 | Baseline |
| Male | 15 to 21 | 0 | #NUM! | 0 | Baseline |
| Female | 15 to 21 | 0 | #NUM! | 0 | Baseline |
| Male | 5 to 14 | 0 | #NUM! | 0 | Baseline |
| Female | 15 to 21 | 0 | #NUM! | 0 | Baseline |
| Male | 5 to 14 | 0 | #NUM! | 0 | Baseline |
| Female | 15 to 21 | 0 | #NUM! | 0 | Baseline |
| Male | 15 to 21 | 0 | #NUM! | 0 | Baseline |
| Female | 5 to 14 | 0 | #NUM! | 0 | Baseline |
| Female | 15 to 21 | 0 | #NUM! | 0 | Baseline |
| Female | 15 to 21 | 0 | #NUM! | 0 | Baseline |
| Female | 5 to 14 | 0 | #NUM! | 0 | Baseline |
| Female | 15 to 21 | 0 | #NUM! | 0 | Baseline |
| Male | 15 to 21 | 0 | #NUM! | 0 | Baseline |
| Female | 35+ | 0 | #NUM! | 0 | Baseline |
| Female | 35+ | 0 | #NUM! | 0 | Baseline |
| Female | 35+ | 0 | #NUM! | 0 | Baseline |
| Male | 1 to 4 | 0 | #NUM! | 0 | Baseline |
| Female | 35+ | 0 | #NUM! | 0 | Baseline |
| Male | 1 to 4 | 0 | #NUM! | 0 | Baseline |
| Female | 22 to 35 | 0 | #NUM! | 0 | Baseline |
| Female | 35+ | 0 | #NUM! | 0 | Baseline |
| Female | 22 to 35 | 0 | #NUM! | 0 | Baseline |
| Male | 1 to 4 | 0 | #NUM! | 0 | Baseline |
| Male | 35+ | 0 | #NUM! | 0 | Baseline |
| Male | 1 to 4 | 0 | #NUM! | 0 | Baseline |
| Female | 22 to 35 | 0 | #NUM! | 0 | Baseline |
| Female | 35+ | 0 | #NUM! | 0 | Baseline |
| Male | 35+ | 0 | #NUM! | 0 | Baseline |
| Female | 35+ | 0 | #NUM! | 0 | Baseline |
| Female | 22 to 35 | 0 | #NUM! | 0 | Baseline |
| Male | 22 to 35 | 5 | 0 | 0 | Baseline |
| Female | 22 to 35 | 0 | #NUM! | 0 | Baseline |
| Female | 35+ | 0 | #NUM! | 0 | Baseline |
| Female | 22 to 35 | 0 | #NUM! | 0 | Baseline |
| Female | 1 to 4 | 0 | #NUM! | 0 | Baseline |
| Male | 35+ | 0 | #NUM! | 0 | Baseline |
| Female | 1 to 4 | 0 | #NUM! | 0 | Baseline |
| Male | 35+ | 0 | #NUM! | 0 | Baseline |
| Female | 5 to 14 | 0 | #NUM! | 0 | Baseline |
| Female | 35+ | 0 | #NUM! | 0 | Baseline |
| Female | 5 to 14 | 0 | #NUM! | 0 | Baseline |
| Female | 35+ | 0 | #NUM! | 0 | Baseline |
| Female | 35+ | 0 | #NUM! | 0 | Baseline |
| Female | 35+ | 0 | #NUM! | 0 | Baseline |
| Female | 1 to 4 | 0 | #NUM! | 0 | Baseline |
| Female | 22 to 35 | 0 | #NUM! | 0 | Baseline |
| Male | 5 to 14 | 0 | #NUM! | 0 | Baseline |

| Male | 5 to 14 | 0 | #NUM! | 0 | Baseline |
| --- | --- | --- | --- | --- | --- |
| Male | 22 to 35 | 0 | #NUM! | 0 | Baseline |
| Female | 22 to 35 | 0 | #NUM! | 0 | Baseline |
| Male | 22 to 35 | 0 | #NUM! | 0 | Baseline |
| Male | 35+ | 0 | #NUM! | 0 | Baseline |
| Male | 5 to 14 | 0 | #NUM! | 0 | Baseline |
| Male | 1 to 4 | 0 | #NUM! | 0 | Baseline |
| Female | 22 to 35 | 0 | #NUM! | 0 | Baseline |
| Female | 35+ | 0 | #NUM! | 0 | Baseline |
| Male | 35+ | 0 | #NUM! | 0 | Baseline |
| Female | 22 to 35 | 0 | #NUM! | 0 | Baseline |
| Male | 5 to 14 | 0 | #NUM! | 0 | Baseline |
| Male | 5 to 14 | 0 | #NUM! | 0 | Baseline |
| Male | 5 to 14 | 0 | #NUM! | 0 | Baseline |
| Male | 5 to 14 | 0 | #NUM! | 0 | Baseline |
| Male | 1 to 4 | 0 | #NUM! | 0 | Baseline |
| Male | 5 to 14 | 0 | #NUM! | 0 | Baseline |
| Male | 5 to 14 | 0 | #NUM! | 0 | Baseline |
| Male | 5 to 14 | 0 | #NUM! | 0 | Baseline |
| Male | 5 to 14 | 0 | #NUM! | 0 | Baseline |
| Female | 5 to 14 | 0 | #NUM! | 0 | Baseline |
| Female | 5 to 14 | 0 | #NUM! | 0 | Baseline |
| Female | 5 to 14 | 0 | #NUM! | 0 | Baseline |
| Female | 5 to 14 | 0 | #NUM! | 0 | Baseline |
| Female | 5 to 14 | 0 | #NUM! | 0 | Baseline |
| Female | 5 to 14 | 0 | #NUM! | 0 | Baseline |
| Female | 5 to 14 | 0 | #NUM! | 0 | Baseline |
| Female | 5 to 14 | 0 | #NUM! | 0 | Baseline |
| Female | 5 to 14 | 0 | #NUM! | 0 | Baseline |
| Male | 1 to 4 | 0 | #NUM! | 0 | Baseline |
| Male | 22 to 35 | 0 | #NUM! | 0 | Baseline |
| Male | 22 to 35 | 0 | #NUM! | 0 | Baseline |
| Female | 35+ | 0 | #NUM! | 0 | Baseline |
| Male | 35+ | 0 | #NUM! | 0 | Baseline |
| Female | 22 to 35 | 0 | #NUM! | 0 | Baseline |
| Female | 22 to 35 | 0 | #NUM! | 0 | Baseline |
| Female | 22 to 35 | 0 | #NUM! | 0 | Baseline |
| Female | 22 to 35 | 0 | #NUM! | 0 | Baseline |
| Female | 15 to 21 | 0 | #NUM! | 0 | Baseline |
| Female | 22 to 35 | 0 | #NUM! | 0 | Baseline |
| Female | 15 to 21 | 0 | #NUM! | 0 | Baseline |
| Female | 35+ | 0 | #NUM! | 0 | Baseline |
| Female | 15 to 21 | 0 | #NUM! | 0 | Baseline |
| Female | 15 to 21 | 0 | #NUM! | 0 | Baseline |
| Female | 15 to 21 | 0 | #NUM! | 0 | Baseline |
| Female | 15 to 21 | 0 | #NUM! | 0 | Baseline |
| Female | 22 to 35 | 0 | #NUM! | 0 | Baseline |
| Female | 15 to 21 | 0 | #NUM! | 0 | Baseline |
| Male | 1 to 4 | 0 | #NUM! | 0 | Baseline |
| Female | 15 to 21 | 0 | #NUM! | 0 | Baseline |

| Female | 1 to 4 | 0 | #NUM! | 0 | Baseline |
| --- | --- | --- | --- | --- | --- |
| Male | 15 to 21 | 0 | #NUM! | 0 | Baseline |
| Female | 15 to 21 | 0 | #NUM! | 0 | Baseline |
| Male | 22 to 35 | 0 | #NUM! | 0 | Baseline |
| Male | 15 to 21 | 0 | #NUM! | 0 | Baseline |
| Male | 15 to 21 | 0 | #NUM! | 0 | Baseline |
| Female | 1 to 4 | 0 | #NUM! | 0 | Baseline |
| Male | 1 to 4 | 0 | #NUM! | 0 | Baseline |
| Female | 22 to 35 | 0 | #NUM! | 0 | Baseline |
| Male | 1 to 4 | 0 | #NUM! | 0 | Baseline |
| Female | 35+ | 0 | #NUM! | 0 | Baseline |
| Male | 5 to 14 | 0 | #NUM! | 0 | Baseline |
| Male | 35+ | 0 | #NUM! | 0 | Baseline |
| Female | 35+ | 0 | #NUM! | 0 | Baseline |
| Male | 1 to 4 | 0 | #NUM! | 0 | Baseline |
| Male | 1 to 4 | 0 | #NUM! | 0 | Baseline |
| Male | 1 to 4 | 0 | #NUM! | 0 | Baseline |
| Female | 15 to 21 | 0 | #NUM! | 0 | Baseline |
| Female | 35+ | 0 | #NUM! | 0 | Baseline |
| Female | 35+ | 0 | #NUM! | 0 | Baseline |
| Male | 5 to 14 | 0 | #NUM! | 0 | Baseline |
| Male | 1 to 4 | 0 | #NUM! | 0 | Baseline |
| Female | 22 to 35 | 0 | #NUM! | 0 | Baseline |
| Male | 35+ | 0 | #NUM! | 0 | Baseline |
| Male | 1 to 4 | 0 | #NUM! | 0 | Baseline |
| Female | 22 to 35 | 0 | #NUM! | 0 | Baseline |
| Male | 1 to 4 | 0 | #NUM! | 0 | Baseline |
| Female | 1 to 4 | 0 | #NUM! | 0 | Baseline |
| Female | 1 to 4 | 0 | #NUM! | 0 | Baseline |
| Female | 1 to 4 | 0 | #NUM! | 0 | Baseline |
| Male | 1 to 4 | 0 | #NUM! | 0 | Baseline |
| Male | 5 to 14 | 0 | #NUM! | 0 | Baseline |
| Male | 1 to 4 | 0 | #NUM! | 0 | Baseline |
| Female | 1 to 4 | 0 | #NUM! | 0 | Baseline |
| Female | 15 to 21 | 0 | #NUM! | 0 | Baseline |
| Male | 1 to 4 | 0 | #NUM! | 0 | Baseline |
| Female | 35+ | 0 | #NUM! | 0 | Baseline |
| Female | 22 to 35 | 0 | #NUM! | 0 | Baseline |
| Male | 1 to 4 | 0 | #NUM! | 0 | Baseline |
| Male | 1 to 4 | 0 | #NUM! | 0 | Baseline |
| Male | 1 to 4 | 0 | #NUM! | 0 | Baseline |
| Female | 35+ | 0 | #NUM! | 0 | Baseline |
| Male | 5 to 14 | 0 | #NUM! | 0 | Baseline |
| Female | 35+ | 0 | #NUM! | 0 | Baseline |
| Male | 22 to 35 | 0 | #NUM! | 0 | Baseline |
| Male | 22 to 35 | 0 | #NUM! | 0 | Baseline |
| Female | 35+ | 0 | #NUM! | 0 | Baseline |
| Female | 35+ | 0 | #NUM! | 0 | Baseline |
| Male | 5 to 14 | 0 | #NUM! | 0 | Baseline |
| Male | 5 to 14 | 0 | #NUM! | 0 | Baseline |

| Female | 5 to 14 | 0 | #NUM! | 0 | Baseline |
| --- | --- | --- | --- | --- | --- |
| Male | 5 to 14 | 0 | #NUM! | 0 | Baseline |
| Male | 15 to 21 | 0 | #NUM! | 0 | Baseline |
| Male | 1 to 4 | 0 | #NUM! | 0 | Baseline |
| Male | 5 to 14 | 0 | #NUM! | 0 | Baseline |
| Male | 5 to 14 | 0 | #NUM! | 0 | Baseline |
| Male | 5 to 14 | 0 | #NUM! | 0 | Baseline |
| Male | 5 to 14 | 0 | #NUM! | 0 | Baseline |
| Male | 5 to 14 | 0 | #NUM! | 0 | Baseline |
| Male | 5 to 14 | 0 | #NUM! | 0 | Baseline |
| Female | 5 to 14 | 0 | #NUM! | 0 | Baseline |
| Female | 5 to 14 | 0 | #NUM! | 0 | Baseline |
| Female | 5 to 14 | 0 | #NUM! | 0 | Baseline |
| Female | 5 to 14 | 0 | #NUM! | 0 | Baseline |
| Male | 5 to 14 | 0 | #NUM! | 0 | Baseline |
| Female | 5 to 14 | 0 | #NUM! | 0 | Baseline |
| Female | 5 to 14 | 0 | #NUM! | 0 | Baseline |
| Male | 5 to 14 | 0 | #NUM! | 0 | Baseline |
| Male | 5 to 14 | 0 | #NUM! | 0 | Baseline |
| Female | 15 to 21 | 0 | #NUM! | 0 | Baseline |
| Female | 15 to 21 | 0 | #NUM! | 0 | Baseline |
| Male | 15 to 21 | 0 | #NUM! | 0 | Baseline |
| Male | 35+ | 0 | #NUM! | 0 | Baseline |
| Male | 35+ | 0 | #NUM! | 0 | Baseline |
| Female | 35+ | 0 | #NUM! | 0 | Baseline |
| Male | 35+ | 0 | #NUM! | 0 | Baseline |
| Female | 22 to 35 | 0 | #NUM! | 0 | Baseline |
| Male | 35+ | 0 | #NUM! | 0 | Baseline |
| Male | 35+ | 0 | #NUM! | 0 | Baseline |
| Male | 1 to 4 | 0 | #NUM! | 0 | Baseline |
| Male | 1 to 4 | 0 | #NUM! | 0 | Baseline |
| Male | 35+ | 0 | #NUM! | 0 | Baseline |
| Female | 35+ | 0 | #NUM! | 0 | Baseline |
| Male | 35+ | 0 | #NUM! | 0 | Baseline |
| Male | 22 to 35 | 0 | #NUM! | 0 | Baseline |
| Female | 5 to 14 | 0 | #NUM! | 0 | Baseline |
| Female | 22 to 35 | 0 | #NUM! | 0 | Baseline |
| Male | 35+ | 0 | #NUM! | 0 | Baseline |
| Male | 1 to 4 | 0 | #NUM! | 0 | Baseline |
| Female | 15 to 21 | 0 | #NUM! | 0 | Baseline |
| Male | 15 to 21 | 0 | #NUM! | 0 | Baseline |
| Female | 15 to 21 | 0 | #NUM! | 0 | Baseline |
| Male | 15 to 21 | 0 | #NUM! | 0 | Baseline |
| Female | 15 to 21 | 0 | #NUM! | 0 | Baseline |
| Male | 15 to 21 | 0 | #NUM! | 0 | Baseline |
| Male | 15 to 21 | 0 | #NUM! | 0 | Baseline |
| Female | 15 to 21 | 0 | #NUM! | 0 | Baseline |
| Female | 15 to 21 | 0 | #NUM! | 0 | Baseline |
| Female | 15 to 21 | 0 | #NUM! | 0 | Baseline |
| Male | 15 to 21 | 0 | #NUM! | 0 | Baseline |

| Male | 15 to 21 | 0 | #NUM! | 0 | Baseline |
| --- | --- | --- | --- | --- | --- |
| Female | 15 to 21 | 0 | #NUM! | 0 | Baseline |
| Female | 35+ | 0 | #NUM! | 0 | Baseline |
| Male | 35+ | 0 | #NUM! | 0 | Baseline |
| Female | 5 to 14 | 0 | #NUM! | 0 | Baseline |
| Male | 1 to 4 | 0 | #NUM! | 0 | Baseline |
| Male | 5 to 14 | 0 | #NUM! | 0 | Baseline |
| Female | 5 to 14 | 0 | #NUM! | 0 | Baseline |
| Male | 35+ | 0 | #NUM! | 0 | Baseline |
| Female | 1 to 4 | 0 | #NUM! | 0 | Baseline |
| Female | 5 to 14 | 0 | #NUM! | 0 | Baseline |
| Male | 1 to 4 | 0 | #NUM! | 0 | Baseline |
| Female | 35+ | 0 | #NUM! | 0 | Baseline |
| Male | 35+ | 0 | #NUM! | 0 | Baseline |
| Male | 1 to 4 | 0 | #NUM! | 0 | Baseline |
| Female | 22 to 35 | 0 | #NUM! | 0 | Baseline |
| Male | 35+ | 0 | #NUM! | 0 | Baseline |
| Male | 1 to 4 | 0 | #NUM! | 0 | Baseline |
| Male | 1 to 4 | 0 | #NUM! | 0 | Baseline |
| Female | 35+ | 0 | #NUM! | 0 | Baseline |
| Male | 35+ | 0 | #NUM! | 0 | Baseline |
| Female | 35+ | 0 | #NUM! | 0 | Baseline |
| Female | 35+ | 0 | #NUM! | 0 | Baseline |
| Male | 1 to 4 | 0 | #NUM! | 0 | Baseline |
| Male | 35+ | 0 | #NUM! | 0 | Baseline |
| Male | 1 to 4 | 0 | #NUM! | 0 | Baseline |
| Male | 1 to 4 | 0 | #NUM! | 0 | Baseline |
| Male | 5 to 14 | 0 | #NUM! | 0 | Baseline |
| Female | 1 to 4 | 0 | #NUM! | 0 | Baseline |
| Male | 22 to 35 | 0 | #NUM! | 0 | Baseline |
| Male | 35+ | 0 | #NUM! | 0 | Baseline |
| Female | 22 to 35 | 0 | #NUM! | 0 | Baseline |
| Female | 35+ | 0 | #NUM! | 0 | Baseline |
| Male | 22 to 35 | 0 | #NUM! | 0 | Baseline |
| Female | 5 to 14 | 0 | #NUM! | 0 | Baseline |
| Female | 5 to 14 | 0 | #NUM! | 0 | Baseline |
| Male | 15 to 21 | 0 | #NUM! | 0 | Baseline |
| Female | 15 to 21 | 0 | #NUM! | 0 | Baseline |
| Female | 5 to 14 | 0 | #NUM! | 0 | Baseline |
| Male | 5 to 14 | 0 | #NUM! | 0 | Baseline |
| Male | 15 to 21 | 0 | #NUM! | 0 | Baseline |
| Female | 5 to 14 | 0 | #NUM! | 0 | Baseline |
| Male | 15 to 21 | 0 | #NUM! | 0 | Baseline |
| Female | 5 to 14 | 0 | #NUM! | 0 | Baseline |
| Male | 5 to 14 | 0 | #NUM! | 0 | Baseline |
| Male | 15 to 21 | 0 | #NUM! | 0 | Baseline |
| Female | 15 to 21 | 0 | #NUM! | 0 | Baseline |
| Female | 5 to 14 | 0 | #NUM! | 0 | Baseline |
| Male | 5 to 14 | 0 | #NUM! | 0 | Baseline |
| Male | 5 to 14 | 0 | #NUM! | 0 | Baseline |

| Female | 5 to 14 | 0 | #NUM! | 0 | Baseline |
| --- | --- | --- | --- | --- | --- |
| Male | 1 to 4 | 0 | #NUM! | 0 | Baseline |
| Male | 1 to 4 | 0 | #NUM! | 0 | Baseline |
| Female | 22 to 35 | 0 | #NUM! | 0 | Baseline |
| Female | 35+ | 0 | #NUM! | 0 | Baseline |
| Male | 35+ | 0 | #NUM! | 0 | Baseline |
| Female | 22 to 35 | 0 | #NUM! | 0 | Baseline |
| Female | 22 to 35 | 0 | #NUM! | 0 | Baseline |
| Female | 35+ | 0 | #NUM! | 0 | Baseline |
| Male | 1 to 4 | 0 | #NUM! | 0 | Baseline |
| Female | 22 to 35 | 0 | #NUM! | 0 | Baseline |
| Female | 35+ | 4 | 0 | 0 | Baseline |
| Female | 22 to 35 | 0 | #NUM! | 0 | Baseline |
| Female | 22 to 35 | 0 | #NUM! | 0 | Baseline |
| Female | 35+ | 0 | #NUM! | 0 | Baseline |
| Female | 22 to 35 | 0 | #NUM! | 0 | Baseline |
| Male | 5 to 14 | 0 | #NUM! | 0 | Baseline |
| Female | 1 to 4 | 0 | #NUM! | 0 | Baseline |
| Female | 1 to 4 | 0 | #NUM! | 0 | Baseline |
| Male | 1 to 4 | 0 | #NUM! | 0 | Baseline |
| Female | 1 to 4 | 0 | #NUM! | 0 | Baseline |
| Female | 1 to 4 | 0 | #NUM! | 0 | Baseline |
| Female | 15 to 21 | 0 | #NUM! | 0 | Baseline |
| Male | 15 to 21 | 0 | #NUM! | 0 | Baseline |
| Female | 5 to 14 | 0 | #NUM! | 0 | Baseline |
| Male | 5 to 14 | 0 | #NUM! | 0 | Baseline |
| Male | 5 to 14 | 0 | #NUM! | 0 | Baseline |
| Male | 5 to 14 | 0 | #NUM! | 0 | Baseline |
| Female | 15 to 21 | 0 | #NUM! | 0 | Baseline |
| Male | 5 to 14 | 0 | #NUM! | 0 | Baseline |
| Male | 5 to 14 | 0 | #NUM! | 0 | Baseline |
| Male | 5 to 14 | 0 | #NUM! | 0 | Baseline |
| Female | 5 to 14 | 0 | #NUM! | 0 | Baseline |
| Female | 15 to 21 | 0 | #NUM! | 0 | Baseline |
| Female | 15 to 21 | 0 | #NUM! | 0 | Baseline |
| Male | 5 to 14 | 0 | #NUM! | 0 | Baseline |
| Female | 15 to 21 | 0 | #NUM! | 0 | Baseline |
| Female | 1 to 4 | 0 | #NUM! | 0 | Baseline |
| Female | 1 to 4 | 0 | #NUM! | 0 | Baseline |
| Male | 1 to 4 | 0 | #NUM! | 0 | Baseline |
| Male | 1 to 4 | 0 | #NUM! | 0 | Baseline |
| Male | 5 to 14 | 0 | #NUM! | 0 | Baseline |
| Male | 1 to 4 | 0 | #NUM! | 0 | Baseline |
| Male | 1 to 4 | 0 | #NUM! | 0 | Baseline |
| Male | 1 to 4 | 0 | #NUM! | 0 | Baseline |
| Male | 5 to 14 | 0 | #NUM! | 0 | Baseline |
| Male | 5 to 14 | 0 | #NUM! | 0 | Baseline |
| Female | 5 to 14 | 0 | #NUM! | 0 | Baseline |
| Female | 15 to 21 | 0 | #NUM! | 0 | Baseline |
| Female | 15 to 21 | 0 | #NUM! | 0 | Baseline |

| Male | 1 to 4 | 0 | #NUM! | 0 | Baseline |
| --- | --- | --- | --- | --- | --- |
| Male | 1 to 4 | 0 | #NUM! | 0 | Baseline |
| Male | 5 to 14 | 0 | #NUM! | 0 | Baseline |
| Female | 1 to 4 | 0 | #NUM! | 0 | Baseline |
| Male | 5 to 14 | 0 | #NUM! | 0 | Baseline |
| Male | 15 to 21 | 0 | #NUM! | 0 | Baseline |
| Male | 1 to 4 | 0 | #NUM! | 0 | Baseline |
| Female | 5 to 14 | 0 | #NUM! | 0 | Baseline |
| Male | 15 to 21 | 0 | #NUM! | 0 | Baseline |
| Female | 5 to 14 | 0 | #NUM! | 0 | Baseline |
| Male | 15 to 21 | 0 | #NUM! | 0 | Baseline |
| Female | 5 to 14 | 0 | #NUM! | 0 | Baseline |
| Female | 5 to 14 | 0 | #NUM! | 0 | Baseline |
| Male | 15 to 21 | 0 | #NUM! | 0 | Baseline |
| Male | 15 to 21 | 0 | #NUM! | 0 | Baseline |
| Male | 15 to 21 | 0 | #NUM! | 0 | Baseline |
| Female | 15 to 21 | 0 | #NUM! | 0 | Baseline |
| Male | 15 to 21 | 0 | #NUM! | 0 | Baseline |
| Male | 15 to 21 | 0 | #NUM! | 0 | Baseline |
| Female | 15 to 21 | 0 | #NUM! | 0 | Baseline |
| Female | 15 to 21 | 0 | #NUM! | 0 | Baseline |
| Female | 15 to 21 | 0 | #NUM! | 0 | Baseline |
| Female | 22 to 35 | 0 | #NUM! | 0 | Baseline |
| Female | 22 to 35 | 0 | #NUM! | 0 | Baseline |
| Female | 35+ | 0 | #NUM! | 0 | Baseline |
| Female | 22 to 35 | 0 | #NUM! | 0 | Baseline |
| Male | 1 to 4 | 0 | #NUM! | 0 | Baseline |
| Male | 35+ | 0 | #NUM! | 0 | Baseline |
| Female | 35+ | 0 | #NUM! | 0 | Baseline |
| Female | 35+ | 0 | #NUM! | 0 | Baseline |
| Female | 22 to 35 | 0 | #NUM! | 0 | Baseline |
| Female | 22 to 35 | 0 | #NUM! | 0 | Baseline |
| Male | 1 to 4 | 0 | #NUM! | 0 | Baseline |
| Female | 22 to 35 | 0 | #NUM! | 0 | Baseline |
| Male | 35+ | 0 | #NUM! | 0 | Baseline |
| Male | 22 to 35 | 0 | #NUM! | 0 | Baseline |
| Male | 35+ | 0 | #NUM! | 0 | Baseline |
| Female | 35+ | 0 | #NUM! | 0 | Baseline |
| Female | 22 to 35 | 0 | #NUM! | 0 | Baseline |
| Female | 35+ | 0 | #NUM! | 0 | Baseline |
| Female | 22 to 35 | 0 | #NUM! | 0 | Baseline |
| Male | 1 to 4 | 0 | #NUM! | 0 | Baseline |
| Female | 22 to 35 | 0 | #NUM! | 0 | Baseline |
| Female | 22 to 35 | 0 | #NUM! | 0 | Baseline |
| Male | 35+ | 0 | #NUM! | 0 | Baseline |
| Male | 35+ | 0 | #NUM! | 0 | Baseline |
| Female | 22 to 35 | 0 | #NUM! | 0 | Baseline |
| Male | 35+ | 0 | #NUM! | 0 | Baseline |
| Male | 35+ | 1 | 0 | 0 | Baseline |
| Male | 35+ | 0 | #NUM! | 0 | Baseline |

| Male | 22 to 35 | 1 | 0 | 0 | Baseline |
| --- | --- | --- | --- | --- | --- |
| Male | 1 to 4 | 0 | #NUM! | 0 | Baseline |
| Female | 35+ | 0 | #NUM! | 0 | Baseline |
| Female | 35+ | 0 | #NUM! | 0 | Baseline |
| Male | 35+ | 0 | #NUM! | 0 | Baseline |
| Male | 35+ | 0 | #NUM! | 0 | Baseline |
| Male | 1 to 4 | 0 | #NUM! | 0 | Baseline |
| Female | 15 to 21 | 0 | #NUM! | 0 | Baseline |
| Female | 1 to 4 | 0 | #NUM! | 0 | Baseline |
| Female | 1 to 4 | 0 | #NUM! | 0 | Baseline |
| Male | 35+ | 0 | #NUM! | 0 | Baseline |
| Female | 35+ | 0 | #NUM! | 0 | Baseline |
| Male | 22 to 35 | 0 | #NUM! | 0 | Baseline |
| Male | 1 to 4 | 0 | #NUM! | 0 | Baseline |
| Female | 22 to 35 | 0 | #NUM! | 0 | Baseline |
| Female | 15 to 21 | 0 | #NUM! | 0 | Baseline |
| Male | 1 to 4 | 0 | #NUM! | 0 | Baseline |
| Male | 1 to 4 | 0 | #NUM! | 0 | Baseline |
| Male | 1 to 4 | 0 | #NUM! | 0 | Baseline |
| Female | 35+ | 0 | #NUM! | 0 | Baseline |
| Female | 15 to 21 | 0 | #NUM! | 0 | Baseline |
| Male | 15 to 21 | 0 | #NUM! | 0 | Baseline |
| Male | 15 to 21 | 0 | #NUM! | 0 | Baseline |
| Female | 15 to 21 | 0 | #NUM! | 0 | Baseline |
| Female | 15 to 21 | 0 | #NUM! | 0 | Baseline |
| Male | 15 to 21 | 0 | #NUM! | 0 | Baseline |
| Male | 1 to 4 | 0 | #NUM! | 0 | Baseline |
| Female | 5 to 14 | 0 | #NUM! | 0 | Baseline |
| Female | 5 to 14 | 0 | #NUM! | 0 | Baseline |
| Female | 5 to 14 | 0 | #NUM! | 0 | Baseline |
| Female | 15 to 21 | 0 | #NUM! | 0 | Baseline |
| Female | 35+ | 0 | #NUM! | 0 | Baseline |
| Male | 22 to 35 | 0 | #NUM! | 0 | Baseline |
| Male | 5 to 14 | 0 | #NUM! | 0 | Baseline |
| Female | 5 to 14 | 0 | #NUM! | 0 | Baseline |
| Female | 5 to 14 | 0 | #NUM! | 0 | Baseline |
| Male | 15 to 21 | 0 | #NUM! | 0 | Baseline |
| Female | 35+ | 0 | #NUM! | 0 | Baseline |
| Female | 35+ | 0 | #NUM! | 0 | Baseline |
| Female | 35+ | 0 | #NUM! | 0 | Baseline |
| Female | 5 to 14 | 0 | #NUM! | 0 | Baseline |
| Female | 15 to 21 | 0 | #NUM! | 0 | Baseline |
| Female | 15 to 21 | 0 | #NUM! | 0 | Baseline |
| Female | 35+ | 0 | #NUM! | 0 | Baseline |
| Male | 15 to 21 | 0 | #NUM! | 0 | Baseline |
| Male | 15 to 21 | 0 | #NUM! | 0 | Baseline |
| Female | 5 to 14 | 0 | #NUM! | 0 | Baseline |
| Male | 5 to 14 | 0 | #NUM! | 0 | Baseline |
| Female | 5 to 14 | 0 | #NUM! | 0 | Baseline |
| Male | 5 to 14 | 0 | #NUM! | 0 | Baseline |

| Male | 15 to 21 | 0 | #NUM! | 0 | Baseline |
| --- | --- | --- | --- | --- | --- |
| Male | 35+ | 0 | #NUM! | 0 | Baseline |
| Male | 1 to 4 | 0 | #NUM! | 0 | Baseline |
| Male | 1 to 4 | 0 | #NUM! | 0 | Baseline |
| Male | 1 to 4 | 0 | #NUM! | 0 | Baseline |
| Male | 22 to 35 | 0 | #NUM! | 0 | Baseline |
| Female | 35+ | 0 | #NUM! | 0 | Baseline |
| Male | 35+ | 0 | #NUM! | 0 | Baseline |
| Female | 1 to 4 | 0 | #NUM! | 0 | Baseline |
| Male | 35+ | 0 | #NUM! | 0 | Baseline |
| Female | 35+ | 0 | #NUM! | 0 | Baseline |
| Female | 5 to 14 | 0 | #NUM! | 0 | Baseline |
| Female | 15 to 21 | 0 | #NUM! | 0 | Baseline |
| Male | 1 to 4 | 0 | #NUM! | 0 | Baseline |
| Female | 5 to 14 | 0 | #NUM! | 0 | Baseline |
| Male | 5 to 14 | 0 | #NUM! | 0 | Baseline |
| Female | 5 to 14 | 0 | #NUM! | 0 | Baseline |
| Female | 5 to 14 | 0 | #NUM! | 0 | Baseline |
| Male | 5 to 14 | 0 | #NUM! | 0 | Baseline |
| Male | 5 to 14 | 0 | #NUM! | 0 | Baseline |
| Female | 5 to 14 | 0 | #NUM! | 0 | Baseline |
| Female | 5 to 14 | 0 | #NUM! | 0 | Baseline |
| Female | 5 to 14 | 0 | #NUM! | 0 | Baseline |
| Male | 5 to 14 | 0 | #NUM! | 0 | Baseline |
| Male | 5 to 14 | 0 | #NUM! | 0 | Baseline |
| Male | 1 to 4 | 0 | #NUM! | 0 | Baseline |
| Male | 1 to 4 | 0 | #NUM! | 0 | Baseline |
| Female | 15 to 21 | 0 | #NUM! | 0 | Baseline |
| Male | 5 to 14 | 0 | #NUM! | 0 | Baseline |
| Female | 35+ | 0 | #NUM! | 0 | Baseline |
| Male | 35+ | 0 | #NUM! | 0 | Baseline |
| Male | 15 to 21 | 0 | #NUM! | 0 | Baseline |
| Female | 22 to 35 | 0 | #NUM! | 0 | Baseline |
| Female | 35+ | 0 | #NUM! | 0 | Baseline |
| Male | 1 to 4 | 0 | #NUM! | 0 | Baseline |
| Male | 1 to 4 | 0 | #NUM! | 0 | Baseline |
| Male | 15 to 21 | 0 | #NUM! | 0 | Baseline |
| Female | 22 to 35 | 0 | #NUM! | 0 | Baseline |
| Female | 22 to 35 | 0 | #NUM! | 0 | Baseline |
| Female | 35+ | 0 | #NUM! | 0 | Baseline |
| Male | 22 to 35 | 0 | #NUM! | 0 | Baseline |
| Female | 22 to 35 | 0 | #NUM! | 0 | Baseline |
| Female | 22 to 35 | 0 | #NUM! | 0 | Baseline |
| Female | 22 to 35 | 0 | #NUM! | 0 | Baseline |
| Male | 15 to 21 | 0 | #NUM! | 0 | Baseline |
| Female | 22 to 35 | 0 | #NUM! | 0 | Baseline |
| Female | 15 to 21 | 0 | #NUM! | 0 | Baseline |
| Male | 35+ | 0 | #NUM! | 0 | Baseline |
| Male | 22 to 35 | 0 | #NUM! | 0 | Baseline |
| Female | 35+ | 0 | #NUM! | 0 | Baseline |

| Female | 15 to 21 | 0 | #NUM! | 0 | Baseline |
| --- | --- | --- | --- | --- | --- |
| Male | 22 to 35 | 0 | #NUM! | 0 | Baseline |
| Male | 15 to 21 | 0 | #NUM! | 0 | Baseline |
| Male | 22 to 35 | 0 | #NUM! | 0 | Baseline |
| Male | 35+ | 0 | #NUM! | 0 | Baseline |
| Male | 15 to 21 | 0 | #NUM! | 0 | Baseline |
| Male | 35+ | 0 | #NUM! | 0 | Baseline |
| Female | 5 to 14 | 0 | #NUM! | 0 | Baseline |
| Male | 5 to 14 | 0 | #NUM! | 0 | Baseline |
| Male | 5 to 14 | 0 | #NUM! | 0 | Baseline |
| Female | 5 to 14 | 0 | #NUM! | 0 | Baseline |
| Female | 5 to 14 | 0 | #NUM! | 0 | Baseline |
| Female | 5 to 14 | 0 | #NUM! | 0 | Baseline |
| Male | 5 to 14 | 0 | #NUM! | 0 | Baseline |
| Male | 1 to 4 | 0 | #NUM! | 0 | Baseline |
| Female | 5 to 14 | 0 | #NUM! | 0 | Baseline |
| Female | 5 to 14 | 0 | #NUM! | 0 | Baseline |
| Female | 5 to 14 | 0 | #NUM! | 0 | Baseline |
| Male | 5 to 14 | 0 | #NUM! | 0 | Baseline |
| Male | 5 to 14 | 0 | #NUM! | 0 | Baseline |
| Male | 5 to 14 | 0 | #NUM! | 0 | Baseline |
| Male | 5 to 14 | 0 | #NUM! | 0 | Baseline |
| Female | 5 to 14 | 0 | #NUM! | 0 | Baseline |
| Male | 5 to 14 | 0 | #NUM! | 0 | Baseline |
| Male | 5 to 14 | 0 | #NUM! | 0 | Baseline |
| Female | 5 to 14 | 0 | #NUM! | 0 | Baseline |
| Female | 22 to 35 | 0 | #NUM! | 0 | Baseline |
| Female | 22 to 35 | 0 | #NUM! | 0 | Baseline |
| Male | 22 to 35 | 0 | #NUM! | 0 | Baseline |
| Male | 15 to 21 | 0 | #NUM! | 0 | Baseline |
| Female | 22 to 35 | 0 | #NUM! | 0 | Baseline |
| Female | 22 to 35 | 0 | #NUM! | 0 | Baseline |
| Male | 22 to 35 | 0 | #NUM! | 0 | Baseline |
| Male | 22 to 35 | 0 | #NUM! | 0 | Baseline |
| Female | 22 to 35 | 0 | #NUM! | 0 | Baseline |
| Female | 22 to 35 | 0 | #NUM! | 0 | Baseline |
| Female | 22 to 35 | 0 | #NUM! | 0 | Baseline |
| Female | 22 to 35 | 0 | #NUM! | 0 | Baseline |
| Female | 22 to 35 | 0 | #NUM! | 0 | Baseline |
| Male | 35+ | 0 | #NUM! | 0 | Baseline |
| Female | 35+ | 0 | #NUM! | 0 | Baseline |
| Female | 35+ | 0 | #NUM! | 0 | Baseline |
| Female | 35+ | 0 | #NUM! | 0 | Baseline |
| Female | 35+ | 0 | #NUM! | 0 | Baseline |
| Female | 15 to 21 | 0 | #NUM! | 0 | Baseline |
| Male | 15 to 21 | 0 | #NUM! | 0 | Baseline |
| Male | 35+ | 0 | #NUM! | 0 | Baseline |
| Female | 15 to 21 | 0 | #NUM! | 0 | Baseline |
| Male | 22 to 35 | 0 | #NUM! | 0 | Baseline |
| Male | 35+ | 0 | #NUM! | 0 | Baseline |

| Male | 1 to 4 | 0 | #NUM! | 0 | Baseline |
| --- | --- | --- | --- | --- | --- |
| Male | 22 to 35 | 0 | #NUM! | 0 | Baseline |
| Male | 22 to 35 | 0 | #NUM! | 0 | Baseline |
| Female | 35+ | 0 | #NUM! | 0 | Baseline |
| Male | 35+ | 0 | #NUM! | 0 | Baseline |
| Female | 35+ | 0 | #NUM! | 0 | Baseline |
| Male | 22 to 35 | 0 | #NUM! | 0 | Baseline |
| Female | 35+ | 0 | #NUM! | 0 | Baseline |
| Male | 35+ | 0 | #NUM! | 0 | Baseline |
| Female | 35+ | 0 | #NUM! | 0 | Baseline |
| Female | 35+ | 0 | #NUM! | 0 | Baseline |
| Male | 22 to 35 | 0 | #NUM! | 0 | Baseline |
| Male | 1 to 4 | 0 | #NUM! | 0 | Baseline |
| Male | 22 to 35 | 0 | #NUM! | 0 | Baseline |
| Female | 35+ | 0 | #NUM! | 0 | Baseline |
| Female | 35+ | 0 | #NUM! | 0 | Baseline |
| Female | 22 to 35 | 0 | #NUM! | 0 | Baseline |
| Male | 15 to 21 | 0 | #NUM! | 0 | Baseline |
| Female | 22 to 35 | 0 | #NUM! | 0 | Baseline |
| Male | 35+ | 0 | #NUM! | 0 | Baseline |
| Male | 1 to 4 | 0 | #NUM! | 0 | Baseline |
| Male | 1 to 4 | 0 | #NUM! | 0 | Baseline |
| Female | 1 to 4 | 0 | #NUM! | 0 | Baseline |
| Female | 1 to 4 | 0 | #NUM! | 0 | Baseline |
| Male | 1 to 4 | 0 | #NUM! | 0 | Baseline |
| Male | 1 to 4 | 0 | #NUM! | 0 | Baseline |
| Male | 1 to 4 | 0 | #NUM! | 0 | Baseline |
| Female | 1 to 4 | 0 | #NUM! | 0 | Baseline |
| Male | 1 to 4 | 0 | #NUM! | 0 | Baseline |
| Female | 1 to 4 | 0 | #NUM! | 0 | Baseline |
| Female | 1 to 4 | 0 | #NUM! | 0 | Baseline |
| Male | 1 to 4 | 0 | #NUM! | 0 | Baseline |
| Male | 5 to 14 | 0 | #NUM! | 0 | Baseline |
| Male | 5 to 14 | 0 | #NUM! | 0 | Baseline |
| Male | 5 to 14 | 0 | #NUM! | 0 | Baseline |
| Male | 5 to 14 | 0 | #NUM! | 0 | Baseline |
| Female | 35+ | 0 | #NUM! | 0 | Baseline |
| Female | 22 to 35 | 0 | #NUM! | 0 | Baseline |
| Male | 1 to 4 | 0 | #NUM! | 0 | Baseline |
| Male | 35+ | 0 | #NUM! | 0 | Baseline |
| Male | 1 to 4 | 0 | #NUM! | 0 | Baseline |
| Female | 35+ | 0 | #NUM! | 0 | Baseline |
| Female | 5 to 14 | 0 | #NUM! | 0 | Baseline |
| Male | 1 to 4 | 0 | #NUM! | 0 | Baseline |
| Female | 35+ | 0 | #NUM! | 0 | Baseline |
| Male | 35+ | 0 | #NUM! | 0 | Baseline |
| Female | 15 to 21 | 0 | #NUM! | 0 | Baseline |
| Male | 22 to 35 | 0 | #NUM! | 0 | Baseline |
| Female | 22 to 35 | 0 | #NUM! | 0 | Baseline |
| Male | 35+ | 0 | #NUM! | 0 | Baseline |

| Female | 15 to 21 | 0 | #NUM! | 0 | Baseline |
| --- | --- | --- | --- | --- | --- |
| Female | 5 to 14 | 0 | #NUM! | 0 | Baseline |
| Male | 15 to 21 | 0 | #NUM! | 0 | Baseline |
| Male | 35+ |  | #NUM! | 0 | Baseline |
| Female | 35+ | 0 | #NUM! | 0 | Baseline |
| Female | 35+ | 0 | #NUM! | 0 | Baseline |
| Male | 35+ | 0 | #NUM! | 0 | Baseline |
| Female | 5 to 14 | 0 | #NUM! | 0 | Baseline |
| Female | 5 to 14 | 1 | #NUM! | 0 | Baseline |
| Male | 5 to 14 | 0 | #NUM! | 0 | Baseline |
| Male | 35+ | 0 | #NUM! | 0 | Baseline |
| Female | 22 to 35 | 0 | #NUM! | 0 | Baseline |
| Female | 35+ | 0 | #NUM! | 0 | Baseline |
| Male | 5 to 14 | 0 | #NUM! | 0 | Baseline |
| Male | 1 to 4 | 0 | #NUM! | 0 | Baseline |
| Male | 5 to 14 | 0 | #NUM! | 0 | Baseline |
| Female | 35+ | 0 | #NUM! | 0 | Baseline |
| Female | 15 to 21 | 0 | #NUM! | 0 | Baseline |
| Female | 35+ | 0 | #NUM! | 0 | Baseline |
| Male | 22 to 35 | 0 | #NUM! | 0 | Baseline |
| Male | 35+ | 0 | #NUM! | 0 | Baseline |
| Male | 5 to 14 | 0 | #NUM! | 0 | Baseline |
| Male | 22 to 35 | 0 | #NUM! | 0 | Baseline |
| Male | 1 to 4 | 0 | #NUM! | 0 | Baseline |
| Male | 22 to 35 | 0 | #NUM! | 0 | Baseline |
| Male | 1 to 4 | 0 | #NUM! | 0 | Baseline |
| Female | 22 to 35 | 0 | #NUM! | 0 | Baseline |
| Male | 22 to 35 | 0 | #NUM! | 0 | Baseline |
| Female | 1 to 4 | 0 | #NUM! | 0 | Baseline |
| Female | 22 to 35 | 0 | #NUM! | 0 | Baseline |
| Male | 1 to 4 | 0 | #NUM! | 0 | Baseline |
| Female | 1 to 4 | 0 | #NUM! | 0 | Baseline |
| Male | 1 to 4 | 0 | #NUM! | 0 | Baseline |
| Female | 22 to 35 | 0 | #NUM! | 0 | Baseline |
| Male | 22 to 35 | 0 | #NUM! | 0 | Baseline |
| Male | 22 to 35 | 0 | #NUM! | 0 | Baseline |
| Male | 1 to 4 | 0 | #NUM! | 0 | Baseline |
| Female | 22 to 35 | 0 | #NUM! | 0 | Baseline |
| Male | 22 to 35 | 0 | #NUM! | 0 | Baseline |
| Female | 1 to 4 | 0 | #NUM! | 0 | Baseline |
| Female | 22 to 35 | 0 | #NUM! | 0 | Baseline |
| Female | 1 to 4 | 0 | #NUM! | 0 | Baseline |
| Female | 1 to 4 | 0 | #NUM! | 0 | Baseline |
| Male | 22 to 35 | 0 | #NUM! | 0 | Baseline |
| Male | 1 to 4 | 0 | #NUM! | 0 | Baseline |
| Female | 22 to 35 | 0 | #NUM! | 0 | Baseline |
| Male | 5 to 14 | 0 | #NUM! | 0 | Baseline |
| Male | 5 to 14 | 0 | #NUM! | 0 | Baseline |
| Male | 5 to 14 | 0 | #NUM! | 0 | Baseline |
| Male | 5 to 14 | 0 | #NUM! | 0 | Baseline |

| Female | 5 to 14 | 0 | #NUM! | 0 | Baseline |
| --- | --- | --- | --- | --- | --- |
| Male | 5 to 14 | 0 | #NUM! | 0 | Baseline |
| Male | 1 to 4 | 0 | #NUM! | 0 | Baseline |
| Male | 1 to 4 | 0 | #NUM! | 0 | Baseline |
| Female | 1 to 4 | 0 | #NUM! | 0 | Baseline |
| Male | 1 to 4 | 0 | #NUM! | 0 | Baseline |
| Male | 1 to 4 | 0 | #NUM! | 0 | Baseline |
| Male | 1 to 4 | 0 | #NUM! | 0 | Baseline |
| Female | 1 to 4 | 0 | #NUM! | 0 | Baseline |
| Male | 1 to 4 | 0 | #NUM! | 0 | Baseline |
| Female | 1 to 4 | 0 | #NUM! | 0 | Baseline |
| Male | 1 to 4 | 0 | #NUM! | 0 | Baseline |
| Female | 22 to 35 | 0 | #NUM! | 0 | Baseline |
| Female | 1 to 4 | 0 | #NUM! | 0 | Baseline |
| Female | 22 to 35 | 0 | #NUM! | 0 | Baseline |
| Male | 22 to 35 | 0 | #NUM! | 0 | Baseline |
| Male | 1 to 4 | 0 | #NUM! | 0 | Baseline |
| Female | 5 to 14 | 0 | #NUM! | 0 | Baseline |
| Male | 22 to 35 | 0 | #NUM! | 0 | Baseline |
| Female | 1 to 4 | 0 | #NUM! | 0 | Baseline |
| Female | 1 to 4 | 0 | #NUM! | 0 | Baseline |
| Female | 22 to 35 | 0 | #NUM! | 0 | Baseline |
| Male | 15 to 21 | 0 | #NUM! | 0 | Baseline |
| Male | 1 to 4 | 0 | #NUM! | 0 | Baseline |
| Male | 1 to 4 | 0 | #NUM! | 0 | Baseline |
| Male | 1 to 4 | 0 | #NUM! | 0 | Baseline |
| Male | 1 to 4 | 0 | #NUM! | 0 | Baseline |
| Female | 1 to 4 | 0 | #NUM! | 0 | Baseline |
| Male | 5 to 14 | 0 | #NUM! | 0 | Baseline |
| Male | 5 to 14 | 0 | #NUM! | 0 | Baseline |
| Male | 1 to 4 | 0 | #NUM! | 0 | Baseline |
| Male | 1 to 4 | 0 | #NUM! | 0 | Baseline |
| Female | 1 to 4 | 0 | #NUM! | 0 | Baseline |
| Male | 1 to 4 | 0 | #NUM! | 0 | Baseline |
| Male | 5 to 14 | 0 | #NUM! | 0 | Baseline |
| Female | 1 to 4 | 0 | #NUM! | 0 | Baseline |
| Male | 1 to 4 | 0 | #NUM! | 0 | Baseline |
| Male | 1 to 4 | 0 | #NUM! | 0 | Baseline |
| Male | 1 to 4 | 0 | #NUM! | 0 | Baseline |
| Male | 5 to 14 | 0 | #NUM! | 0 | Baseline |
| Male | 1 to 4 | 0 | #NUM! | 0 | Baseline |
| Female | 1 to 4 | 0 | #NUM! | 0 | Baseline |
| Male | 5 to 14 | 0 | #NUM! | 0 | Baseline |
| Female | 1 to 4 | 0 | #NUM! | 0 | Baseline |
| Male | 15 to 21 | 0 | #NUM! | 0 | Baseline |
| Male | 15 to 21 | 0 | #NUM! | 0 | Baseline |
| Female | 22 to 35 | 0 | #NUM! | 0 | Baseline |
| Male | 22 to 35 | 0 | #NUM! | 0 | Baseline |
| Male | 1 to 4 | 0 | #NUM! | 0 | Baseline |
| Female | 35+ | 0 | #NUM! | 0 | Baseline |

| Female | 22 to 35 | 0 | #NUM! | 0 | Baseline |
| --- | --- | --- | --- | --- | --- |
| Male | 35+ | 0 | #NUM! | 0 | Baseline |
| Female | 22 to 35 | 0 | #NUM! | 0 | Baseline |
| Male | 35+ | 0 | #NUM! | 0 | Baseline |
| Female | 22 to 35 | 0 | #NUM! | 0 | Baseline |
| Male | 15 to 21 | 0 | #NUM! | 0 | Baseline |
| Male | 15 to 21 | 0 | #NUM! | 0 | Baseline |
| Female | 35+ | 0 | #NUM! | 0 | Baseline |
| Male | 35+ | 0 | #NUM! | 0 | Baseline |
| Female | 35+ | 0 | #NUM! | 0 | Baseline |
| Male | 22 to 35 | 0 | #NUM! | 0 | Baseline |
| Male | 1 to 4 | 0 | #NUM! | 0 | Baseline |
| Male | 1 to 4 | 0 | #NUM! | 0 | Baseline |
| Male | 1 to 4 | 0 | #NUM! | 0 | Baseline |
| Male | 1 to 4 | 0 | #NUM! | 0 | Baseline |
| Male | 1 to 4 | 0 | #NUM! | 0 | Baseline |
| Male | 1 to 4 | 0 | #NUM! | 0 | Baseline |
| Male | 1 to 4 | 0 | #NUM! | 0 | Baseline |
| Male | 1 to 4 | 0 | #NUM! | 0 | Baseline |
| Male | 1 to 4 | 0 | #NUM! | 0 | Baseline |
| Male | 1 to 4 | 0 | #NUM! | 0 | Baseline |
| Male | 1 to 4 | 0 | #NUM! | 0 | Baseline |
| Male | 1 to 4 | 0 | #NUM! | 0 | Baseline |
| Male | 1 to 4 | 0 | #NUM! | 0 | Baseline |
| Male | 1 to 4 | 0 | #NUM! | 0 | Baseline |
| Female | 1 to 4 | 0 | #NUM! | 0 | Baseline |
| Female | 1 to 4 | 0 | #NUM! | 0 | Baseline |
| Male | 1 to 4 | 0 | #NUM! | 0 | Baseline |
| Male | 1 to 4 | 0 | #NUM! | 0 | Baseline |
| Female | 1 to 4 | 0 | #NUM! | 0 | Baseline |
| Male | 1 to 4 | 0 | #NUM! | 0 | Baseline |
| Female | 15 to 21 | 0 | #NUM! | 0 | Baseline |
| Female | 35+ | 0 | #NUM! | 0 | Baseline |
| Female | 22 to 35 | 0 | #NUM! | 0 | Baseline |
| Female | 35+ | 0 | #NUM! | 0 | Baseline |
| Male | 35+ | 0 | #NUM! | 0 | Baseline |
| Female | 35+ | 0 | #NUM! | 0 | Baseline |
| Female | 15 to 21 | 0 | #NUM! | 0 | Baseline |
| Female | 22 to 35 | 0 | #NUM! | 0 | Baseline |
| Female | 35+ | 0 | #NUM! | 0 | Baseline |
| Male | 35+ | 0 | #NUM! | 0 | Baseline |
| Female | 15 to 21 | 0 | #NUM! | 0 | Baseline |
| Male | 35+ | 0 | #NUM! | 0 | Baseline |
| Male | 35+ | 0 | #NUM! | 0 | Baseline |
| Male | 15 to 21 | 0 | #NUM! | 0 | Baseline |
| Female | 22 to 35 | 0 | #NUM! | 0 | Baseline |
| Male | 15 to 21 | 0 | #NUM! | 0 | Baseline |
| Female | 22 to 35 | 0 | #NUM! | 0 | Baseline |
| Male | 22 to 35 | 0 | #NUM! | 0 | Baseline |
| Female | 1 to 4 | 0 | #NUM! | 0 | Baseline |

| Male | 1 to 4 | 0 | #NUM! | 0 | Baseline |
| --- | --- | --- | --- | --- | --- |
| Male | 1 to 4 | 0 | #NUM! | 0 | Baseline |
| Male | 1 to 4 | 0 | #NUM! | 0 | Baseline |
| Male | 5 to 14 | 0 | #NUM! | 0 | Baseline |
| Male | 5 to 14 | 0 | #NUM! | 0 | Baseline |
| Male | 5 to 14 | 0 | #NUM! | 0 | Baseline |
| Male | 5 to 14 | 0 | #NUM! | 0 | Baseline |
| Male | 5 to 14 | 0 | #NUM! | 0 | Baseline |
| Male | 15 to 21 | 0 | #NUM! | 0 | Baseline |
| Male | 15 to 21 | 0 | #NUM! | 0 | Baseline |
| Male | 15 to 21 | 0 | #NUM! | 0 | Baseline |
| Female | 5 to 14 | 0 | #NUM! | 0 | Baseline |
| Female | 5 to 14 | 0 | #NUM! | 0 | Baseline |
| Female | 5 to 14 | 0 | #NUM! | 0 | Baseline |
| Female | 5 to 14 | 0 | #NUM! | 0 | Baseline |
| Female | 5 to 14 | 0 | #NUM! | 0 | Baseline |
| Female | 5 to 14 | 0 | #NUM! | 0 | Baseline |
| Female | 15 to 21 | 0 | #NUM! | 0 | Baseline |
| Female | 15 to 21 | 0 | #NUM! | 0 | Baseline |
| Female | 15 to 21 | 0 | #NUM! | 0 | Baseline |
| Male | 15 to 21 | 0 | #NUM! | 0 | Baseline |
| Male | 15 to 21 | 0 | #NUM! | 0 | Baseline |
| Female | 15 to 21 | 0 | #NUM! | 0 | Baseline |
| Female | 15 to 21 | 0 | #NUM! | 0 | Baseline |
| Female | 15 to 21 | 0 | #NUM! | 0 | Baseline |
| Female | 15 to 21 | 0 | #NUM! | 0 | Baseline |
| Female | 15 to 21 | 0 | #NUM! | 0 | Baseline |
| Female | 22 to 35 | 0 | #NUM! | 0 | Baseline |
| Female | 35+ | 5 | 0 | 0 | Baseline |
| Male | 5 to 14 | 0 | #NUM! | 0 | Baseline |
| Male | 5 to 14 | 0 | #NUM! | 0 | Baseline |
| Male | 5 to 14 | 0 | #NUM! | 0 | Baseline |
| Female | 5 to 14 | 0 | #NUM! | 0 | Baseline |
| Female | 1 to 4 | 0 | #NUM! | 0 | Baseline |
| Female | 5 to 14 | 0 | #NUM! | 0 | Baseline |
| Female | 5 to 14 | 0 | #NUM! | 0 | Baseline |
| Male | 1 to 4 | 0 | #NUM! | 0 | Baseline |
| Male | 5 to 14 | 0 | #NUM! | 0 | Baseline |
| Female | 5 to 14 | 0 | #NUM! | 0 | Baseline |
| Female | 5 to 14 | 0 | #NUM! | 0 | Baseline |
| Female | 5 to 14 | 0 | #NUM! | 0 | Baseline |
| Male | 5 to 14 | 0 | #NUM! | 0 | Baseline |
| Female | 1 to 4 | 0 | #NUM! | 0 | Baseline |
| Female | 1 to 4 | 0 | #NUM! | 0 | Baseline |
| Male | 35+ | 0 | #NUM! | 0 | Baseline |
| Female | 35+ | 0 | #NUM! | 0 | Baseline |
| Female | 35+ | 0 | #NUM! | 0 | Baseline |
| Male | 35+ | 0 | #NUM! | 0 | Baseline |
| Male | 35+ | 0 | #NUM! | 0 | Baseline |
| Female | 35+ | 4 | 0 | 0 | Baseline |

| Female | 35+ | 0 | #NUM! | 0 | Baseline |
| --- | --- | --- | --- | --- | --- |
| Male | 35+ | 0 | #NUM! | 0 | Baseline |
| Male | 35+ | 0 | #NUM! | 0 | Baseline |
| Male | 1 to 4 | 0 | #NUM! | 0 | Baseline |
| Male | 22 to 35 | 0 | #NUM! | 0 | Baseline |
| Male | 22 to 35 | 0 | #NUM! | 0 | Baseline |
| Female | 22 to 35 | 0 | #NUM! | 0 | Baseline |
| Male | 22 to 35 | 0 | #NUM! | 0 | Baseline |
| Male | 22 to 35 | 0 | #NUM! | 0 | Baseline |
| Male | 22 to 35 | 0 | #NUM! | 0 | Baseline |
| Female | 22 to 35 | 0 | #NUM! | 0 | Baseline |
| Female | 22 to 35 | 0 | #NUM! | 0 | Baseline |
| Female | 22 to 35 | 5 | 0 | 0 | Baseline |
| Male | 22 to 35 | 0 | #NUM! | 0 | Baseline |
| Male | 22 to 35 | 0 | #NUM! | 0 | Baseline |
| Male | 22 to 35 | 0 | #NUM! | 0 | Baseline |
| Male | 22 to 35 | 0 | #NUM! | 0 | Baseline |
| Male | 22 to 35 | 0 | #NUM! | 0 | Baseline |
| Female | 22 to 35 | 0 | #NUM! | 0 | Baseline |
| Female | 22 to 35 | 0 | #NUM! | 0 | Baseline |
| Female | 22 to 35 | 0 | #NUM! | 0 | Baseline |
| Female | 35+ | 0 | #NUM! | 0 | Baseline |
| Female | 35+ | 0 | #NUM! | 0 | Baseline |
| Male | 35+ | 0 | #NUM! | 0 | Baseline |
| Male | 35+ | 0 | #NUM! | 0 | Baseline |
| Female | 35+ | 0 | #NUM! | 0 | Baseline |
| Male | 22 to 35 | 0 | #NUM! | 0 | Baseline |
| Female | 22 to 35 | 0 | #NUM! | 0 | Baseline |
| Female | 22 to 35 | 0 | #NUM! | 0 | Baseline |
| Female | 22 to 35 | 0 | #NUM! | 0 | Baseline |
| Male | 5 to 14 | 0 | #NUM! | 0 | Baseline |
| Male | 5 to 14 | 0 | #NUM! | 0 | Baseline |
| Female | 5 to 14 | 3 | 0 | 0 | Baseline |
| Female | 5 to 14 | 0 | #NUM! | 0 | Baseline |
| Male | 5 to 14 | 5 | 0 | 0 | Baseline |
| Female | 5 to 14 | 1 | 0 | 0 | Baseline |
| Female | 15 to 21 | 0 | #NUM! | 0 | Baseline |
| Female | 15 to 21 | 0 | #NUM! | 0 | Baseline |
| Female | 15 to 21 | 0 | #NUM! | 0 | Baseline |
| Female | 15 to 21 | 0 | #NUM! | 0 | Baseline |
| Female | 15 to 21 | 0 | #NUM! | 0 | Baseline |
| Male | 5 to 14 | 0 | #NUM! | 0 | Baseline |
| Male | 5 to 14 | 0 | #NUM! | 0 | Baseline |
| Male | 22 to 35 | 0 | #NUM! | 0 | Baseline |
| Male | 5 to 14 | 0 | #NUM! | 0 | Baseline |
| Male | 5 to 14 | 0 | #NUM! | 0 | Baseline |
| Male | 22 to 35 | 0 | #NUM! | 0 | Baseline |
| Male | 22 to 35 | 0 | #NUM! | 0 | Baseline |
| Male | 22 to 35 | 0 | #NUM! | 0 | Baseline |
| Male | 1 to 4 | 0 | #NUM! | 0 | Baseline |

| Female | 5 to 14 | 0 | #NUM! | 0 | Baseline |
| --- | --- | --- | --- | --- | --- |
| Female | 15 to 21 | 0 | #NUM! | 0 | Baseline |
| Male | 22 to 35 | 0 | #NUM! | 0 | Baseline |
| Male | 15 to 21 | 0 | #NUM! | 0 | Baseline |
| Female | 15 to 21 | 0 | #NUM! | 0 | Baseline |
| Male | 15 to 21 | 1 | 0 | 0 | Baseline |
| Male | 15 to 21 | 0 | #NUM! | 0 | Baseline |
| Female | 15 to 21 | 0 | #NUM! | 0 | Baseline |
| Male | 5 to 14 | 0 | #NUM! | 0 | Baseline |
| Female | 35+ | 0 | #NUM! | 0 | Baseline |
| Female | 35+ | 0 | #NUM! | 0 | Baseline |
| Female | 35+ | 0 | #NUM! | 0 | Baseline |
| Female | 35+ | 0 | #NUM! | 0 | Baseline |
| Female | 35+ | 0 | #NUM! | 0 | Baseline |
| Female | 35+ | 0 | #NUM! | 0 | Baseline |
| Male | 35+ | 0 | #NUM! | 0 | Baseline |
| Male | 35+ | 0 | #NUM! | 0 | Baseline |
| Male | 35+ | 0 | #NUM! | 0 | Baseline |
| Male | 35+ | 0 | #NUM! | 0 | Baseline |
| Male | 35+ | 4 | 0 | 0 | Baseline |
| Male | 1 to 4 | 0 | #NUM! | 0 | Baseline |
| Female | 5 to 14 | 0 | #NUM! | 0 | Baseline |
| Male | 5 to 14 | 0 | #NUM! | 0 | Baseline |
| Female | 1 to 4 | 0 | #NUM! | 0 | Baseline |
| Male | 5 to 14 | 0 | #NUM! | 0 | Baseline |
| Male | 1 to 4 | 0 | #NUM! | 0 | Baseline |
| Female | 15 to 21 | 0 | #NUM! | 0 | Baseline |
| Female | 22 to 35 | 0 | #NUM! | 0 | Baseline |
| Female | 15 to 21 | 0 | #NUM! | 0 | Baseline |
| Female | 15 to 21 | 0 | #NUM! | 0 | Baseline |
| Male | 15 to 21 | 0 | #NUM! | 0 | Baseline |
| Female | 22 to 35 | 0 | #NUM! | 0 | Baseline |
| Female | 22 to 35 | 0 | #NUM! | 0 | Baseline |
| Female | 22 to 35 | 0 | #NUM! | 0 | Baseline |
| Male | 22 to 35 | 0 | #NUM! | 0 | Baseline |
| Female | 22 to 35 | 0 | #NUM! | 0 | Baseline |
| Male | 15 to 21 | 0 | #NUM! | 0 | Baseline |
| Female | 22 to 35 | 0 | #NUM! | 0 | Baseline |
| Female | 15 to 21 | 0 | #NUM! | 0 | Baseline |
| Male | 15 to 21 | 0 | #NUM! | 0 | Baseline |
| Female | 15 to 21 | 0 | #NUM! | 0 | Baseline |
| Male | 15 to 21 | 0 | #NUM! | 0 | Baseline |
| Female | 22 to 35 | 0 | #NUM! | 0 | Baseline |
| Male | 15 to 21 | 0 | #NUM! | 0 | Baseline |
| Female | 22 to 35 | 0 | #NUM! | 0 | Baseline |
| Male | 22 to 35 | 5 | 0 | 0 | Baseline |
| Female | 35+ | 5 | 0 | 0 | Baseline |
| Male | 22 to 35 | 5 | 0 | 0 | Baseline |
| Male | 22 to 35 | 0 | #NUM! | 0 | Baseline |
| Male | 35+ | 0 | #NUM! | 0 | Baseline |

| Male | 22 to 35 | 1 | 0 | 0 | Baseline |
| --- | --- | --- | --- | --- | --- |
| Male | 22 to 35 | 0 | #NUM! | 0 | Baseline |
| Male | 35+ | 0 | #NUM! | 0 | Baseline |
| Female | 35+ | 0 | #NUM! | 0 | Baseline |
| Female | 35+ | 0 | #NUM! | 0 | Baseline |
| Male | 35+ | 0 | #NUM! | 0 | Baseline |
| Male | 35+ | 0 | #NUM! | 0 | Baseline |
| Male | 35+ | 0 | #NUM! | 0 | Baseline |
| Female | 35+ | 0 | #NUM! | 0 | Baseline |
| Male | 22 to 35 | 0 | #NUM! | 0 | Baseline |
| Male | 35+ | 0 | #NUM! | 0 | Baseline |
| Female | 35+ | 0 | #NUM! | 0 | Baseline |
| Female | 35+ | 0 | #NUM! | 0 | Baseline |
| Male | 22 to 35 | 0 | #NUM! | 0 | Baseline |
| Female | 35+ | 0 | #NUM! | 0 | Baseline |
| Male | 22 to 35 | 0 | #NUM! | 0 | Baseline |
| Male | 35+ | 0 | #NUM! | 0 | Baseline |
| Female | 35+ | 0 | #NUM! | 0 | Baseline |
| Male | 35+ | 0 | #NUM! | 0 | Baseline |
| Female | 35+ | 0 | #NUM! | 0 | Baseline |
| Male | 35+ | 0 | #NUM! | 0 | Baseline |
| Female | 35+ | 0 | #NUM! | 0 | Baseline |
| Male | 5 to 14 | 0 | #NUM! | 0 | Baseline |
| Male | 5 to 14 | 0 | #NUM! | 0 | Baseline |
| Male | 5 to 14 | 0 | #NUM! | 0 | Baseline |
| Female | 5 to 14 | 0 | #NUM! | 0 | Baseline |
| Male | 5 to 14 | 0 | #NUM! | 0 | Baseline |
| Female | 5 to 14 | 0 | #NUM! | 0 | Baseline |
| Male | 5 to 14 | 0 | #NUM! | 0 | Baseline |
| Male | 5 to 14 | 0 | #NUM! | 0 | Baseline |
| Female | 5 to 14 | 4 | 0 | 0 | Baseline |
| Female | 5 to 14 | 0 | #NUM! | 0 | Baseline |
| Male | 5 to 14 | 0 | #NUM! | 0 | Baseline |
| Male | 5 to 14 | 0 | #NUM! | 0 | Baseline |
| Female | 5 to 14 | 0 | #NUM! | 0 | Baseline |
| Male | 5 to 14 | 0 | #NUM! | 0 | Baseline |
| Male | 5 to 14 | 0 | #NUM! | 0 | Baseline |
| Female | 5 to 14 | 0 | #NUM! | 0 | Baseline |
| Male | 22 to 35 | 0 | #NUM! | 0 | Baseline |
| Female | 1 to 4 | 0 | #NUM! | 0 | Baseline |
| Male | 5 to 14 | 0 | #NUM! | 0 | Baseline |
| Female | 22 to 35 | 4 | 0 | 0 | Baseline |
| Female | 1 to 4 | 0 | #NUM! | 0 | Baseline |
| Female | 22 to 35 | 0 | #NUM! | 0 | Baseline |
| Male | 35+ | 4 | 0 | 0 | Baseline |
| Male | 22 to 35 | 0 | #NUM! | 0 | Baseline |
| Female | 5 to 14 | 0 | #NUM! | 0 | Baseline |
| Male | 5 to 14 | 0 | #NUM! | 0 | Baseline |
| Female | 5 to 14 | 0 | #NUM! | 0 | Baseline |
| Female | 5 to 14 | 0 | #NUM! | 0 | Baseline |

| Male | 5 to 14 | 0 | #NUM! | 0 | Baseline |
| --- | --- | --- | --- | --- | --- |
| Male | 1 to 4 | 0 | #NUM! | 0 | Baseline |
| Male | 5 to 14 | 0 | #NUM! | 0 | Baseline |
| Female | 5 to 14 | 0 | #NUM! | 0 | Baseline |
| Female | 22 to 35 | 0 | #NUM! | 0 | Baseline |
| Male | 5 to 14 | 0 | #NUM! | 0 | Baseline |
| Male | 5 to 14 | 0 | #NUM! | 0 | Baseline |
| Female | 5 to 14 | 0 | #NUM! | 0 | Baseline |
| Female | 5 to 14 | 0 | #NUM! | 0 | Baseline |
| Male | 22 to 35 | 0 | #NUM! | 0 | Baseline |
| Male | 5 to 14 | 0 | #NUM! | 0 | Baseline |
| Male | 22 to 35 | 0 | #NUM! | 0 | Baseline |
| Female | 22 to 35 | 0 | #NUM! | 0 | Baseline |
| Male | 5 to 14 | 0 | #NUM! | 0 | Baseline |
| Female | 5 to 14 | 0 | #NUM! | 0 | Baseline |
| Female | 5 to 14 | 0 | #NUM! | 0 | Baseline |
| Male | 22 to 35 | 0 | #NUM! | 0 | Baseline |
| Male | 1 to 4 | 0 | #NUM! | 0 | Baseline |
| Female | 22 to 35 | 0 | #NUM! | 0 | Baseline |
| Female | 22 to 35 | 0 | #NUM! | 0 | Baseline |
| Female | 1 to 4 | 0 | #NUM! | 0 | Baseline |
| Male | 5 to 14 | 0 | #NUM! | 0 | Baseline |
| Male | 5 to 14 | 0 | #NUM! | 0 | Baseline |
| Female | 5 to 14 | 0 | #NUM! | 0 | Baseline |
| Female | 5 to 14 | 0 | #NUM! | 0 | Baseline |
| Female | 5 to 14 | 0 | #NUM! | 0 | Baseline |
| Female | 22 to 35 | 0 | #NUM! | 0 | Baseline |
| Female | 5 to 14 | 0 | #NUM! | 0 | Baseline |
| Male | 1 to 4 | 1 | 0 | 0 | Baseline |
| Female | 22 to 35 | 0 | #NUM! | 0 | Baseline |
| Male | 5 to 14 | 0 | #NUM! | 0 | Baseline |
| Male | 5 to 14 | 0 | #NUM! | 0 | Baseline |
| Female | 1 to 4 | 0 | #NUM! | 0 | Baseline |
| Male | 5 to 14 | 0 | #NUM! | 0 | Baseline |
| Male | 5 to 14 | 0 | #NUM! | 0 | Baseline |
| Male | 5 to 14 | 0 | #NUM! | 0 | Baseline |
| Female | 5 to 14 | 0 | #NUM! | 0 | Baseline |
| Female | 5 to 14 | 0 | #NUM! | 0 | Baseline |
| Female | 5 to 14 | 0 | #NUM! | 0 | Baseline |
| Female | 5 to 14 | 4 | 0 | 0 | Baseline |
| Male | 5 to 14 | 0 | #NUM! | 0 | Baseline |
| Male | 15 to 21 | 0 | #NUM! | 0 | Baseline |
| Female | 15 to 21 | 0 | #NUM! | 0 | Baseline |
| Male | 5 to 14 | 0 | #NUM! | 0 | Baseline |
| Male | 5 to 14 | 0 | #NUM! | 0 | Baseline |
| Male | 15 to 21 | 0 | #NUM! | 0 | Baseline |
| Male | 15 to 21 | 0 | #NUM! | 0 | Baseline |
| Female | 5 to 14 | 0 | #NUM! | 0 | Baseline |
| Female | 5 to 14 | 0 | #NUM! | 0 | Baseline |
| Female | 5 to 14 | 0 | #NUM! | 0 | Baseline |

| Female | 5 to 14 | 0 | #NUM! | 0 | Baseline |
| --- | --- | --- | --- | --- | --- |
| Female | 5 to 14 | 0 | #NUM! | 0 | Baseline |
| Male | 5 to 14 | 0 | #NUM! | 0 | Baseline |
| Female | 5 to 14 | 0 | #NUM! | 0 | Baseline |
| Female | 5 to 14 | 0 | #NUM! | 0 | Baseline |
| Male | 5 to 14 | 0 | #NUM! | 0 | Baseline |
| Male | 5 to 14 | 0 | #NUM! | 0 | Baseline |
| Female | 15 to 21 | 0 | #NUM! | 0 | Baseline |
| Male | 15 to 21 | 0 | #NUM! | 0 | Baseline |
| Female | 15 to 21 | 0 | #NUM! | 0 | Baseline |
| Female | 15 to 21 | 0 | #NUM! | 0 | Baseline |
| Male | 15 to 21 | 0 | #NUM! | 0 | Baseline |
| Female | 15 to 21 | 0 | #NUM! | 0 | Baseline |
| Female | 15 to 21 | 0 | #NUM! | 0 | Baseline |
| Female | 15 to 21 | 0 | #NUM! | 0 | Baseline |
| Male | 15 to 21 | 0 | #NUM! | 0 | Baseline |
| Male | 15 to 21 | 0 | #NUM! | 0 | Baseline |
| Male | 15 to 21 | 0 | #NUM! | 0 | Baseline |
| Female | 22 to 35 | 0 | #NUM! | 0 | Baseline |
| Female | 22 to 35 | 0 | #NUM! | 0 | Baseline |
| Female | 22 to 35 | 0 | #NUM! | 0 | Baseline |
| Female | 22 to 35 | 0 | #NUM! | 0 | Baseline |
| Female | 22 to 35 | 0 | #NUM! | 0 | Baseline |
| Male | 22 to 35 | 1 | 0 | 0 | Baseline |
| Female | 22 to 35 | 0 | #NUM! | 0 | Baseline |
| Female | 22 to 35 | 0 | #NUM! | 0 | Baseline |
| Male | 22 to 35 | 0 | #NUM! | 0 | Baseline |
| Male | 22 to 35 | 3 | 0 | 0 | Baseline |
| Female | 22 to 35 | 0 | #NUM! | 0 | Baseline |
| Male | 22 to 35 | 0 | #NUM! | 0 | Baseline |
| Male | 22 to 35 | 0 | #NUM! | 0 | Baseline |
| Male | 22 to 35 | 0 | #NUM! | 0 | Baseline |
| Male | 22 to 35 | 0 | #NUM! | 0 | Baseline |
| Female | 35+ | 0 | #NUM! | 0 | Baseline |
| Female | 35+ | 0 | #NUM! | 0 | Baseline |
| Male | 22 to 35 | 0 | #NUM! | 0 | Baseline |
| Female | 35+ | 0 | #NUM! | 0 | Baseline |
| Female | 35+ | 0 | #NUM! | 0 | Baseline |
| Female | 35+ | 0 | #NUM! | 0 | Baseline |
| Female | 35+ | 0 | #NUM! | 0 | Baseline |
| Male | 35+ | 1 | 0 | 0 | Baseline |
| Male | 35+ | 0 | #NUM! | 0 | Baseline |
| Male | 35+ | 0 | #NUM! | 0 | Baseline |
| Male | 35+ | 0 | #NUM! | 0 | Baseline |
| Male | 35+ | 0 | #NUM! | 0 | Baseline |
| Male | 35+ | 0 | #NUM! | 0 | Baseline |
| Female | 35+ | 0 | #NUM! | 0 | Baseline |
| Female | 35+ | 0 | #NUM! | 0 | Baseline |
| Female | 35+ | 0 | #NUM! | 0 | Baseline |
| Male | 35+ | 0 | #NUM! | 0 | Baseline |

| Male | 35+ | 0 | #NUM! | 0 | Baseline |
| --- | --- | --- | --- | --- | --- |
| Male | 35+ | 0 | #NUM! | 0 | Baseline |
| Female | 35+ | 0 | #NUM! | 0 | Baseline |
| Male | 35+ | 0 | #NUM! | 0 | Baseline |
| Male | 1 to 4 | 0 | #NUM! | 0 | Baseline |
| Male | 1 to 4 | 0 | #NUM! | 0 | Baseline |
| Male | 1 to 4 | 0 | #NUM! | 0 | Baseline |
| Male | 1 to 4 | 0 | #NUM! | 0 | Baseline |
| Male | 1 to 4 | 0 | #NUM! | 0 | Baseline |
| Male | 1 to 4 | 0 | #NUM! | 0 | Baseline |
| Male | 1 to 4 | 0 | #NUM! | 0 | Baseline |
| Male | 1 to 4 | 0 | #NUM! | 0 | Baseline |
| Male | 1 to 4 | 0 | #NUM! | 0 | Baseline |
| Male | 1 to 4 | 0 | #NUM! | 0 | Baseline |
| Male | 1 to 4 | 0 | #NUM! | 0 | Baseline |
| Male | 1 to 4 | 0 | #NUM! | 0 | Baseline |
| Male | 1 to 4 | 0 | #NUM! | 0 | Baseline |
| Male | 1 to 4 | 0 | #NUM! | 0 | Baseline |
| Male | 1 to 4 | 0 | #NUM! | 0 | Baseline |
| Male | 1 to 4 | 0 | #NUM! | 0 | Baseline |
| Male | 1 to 4 | 0 | #NUM! | 0 | Baseline |
| Male | 1 to 4 | 0 | #NUM! | 0 | Baseline |
| Male | 1 to 4 | 0 | #NUM! | 0 | Baseline |
| Male | 1 to 4 | 0 | #NUM! | 0 | Baseline |
| Male | 1 to 4 | 0 | #NUM! | 0 | Baseline |
| Male | 1 to 4 | 0 | #NUM! | 0 | Baseline |
| Male | 1 to 4 | 0 | #NUM! | 0 | Baseline |
| Male | 1 to 4 | 0 | #NUM! | 0 | Baseline |
| Male | 1 to 4 | 0 | #NUM! | 0 | Baseline |
| Male | 1 to 4 | 0 | #NUM! | 0 | Baseline |
| Male | 1 to 4 | 0 | #NUM! | 0 | Baseline |
| Male | 1 to 4 | 0 | #NUM! | 0 | Baseline |
| Male | 1 to 4 | 0 | #NUM! | 0 | Baseline |
| Male | 1 to 4 | 0 | #NUM! | 0 | Baseline |
| Male | 1 to 4 | 0 | #NUM! | 0 | Baseline |
| Male | 1 to 4 | 0 | #NUM! | 0 | Baseline |
| Male | 1 to 4 | 0 | #NUM! | 0 | Baseline |
| Male | 1 to 4 | 0 | #NUM! | 0 | Baseline |
| Male | 1 to 4 | 0 | #NUM! | 0 | Baseline |
| Male | 1 to 4 | 0 | #NUM! | 0 | Baseline |
| Male | 1 to 4 | 0 | #NUM! | 0 | Baseline |
| Male | 1 to 4 | 0 | #NUM! | 0 | Baseline |
| Male | 1 to 4 | 0 | #NUM! | 0 | Baseline |
| Male | 1 to 4 | 0 | #NUM! | 0 | Baseline |
| Male | 1 to 4 | 0 | #NUM! | 0 | Baseline |
| Male | 1 to 4 | 0 | #NUM! | 0 | Baseline |
| Male | 1 to 4 | 0 | #NUM! | 0 | Baseline |
| Male | 1 to 4 | 0 | #NUM! | 0 | Baseline |
| Male | 1 to 4 | 0 | #NUM! | 0 | Baseline |
| Male | 1 to 4 | 0 | #NUM! | 0 | Baseline |

| Male | 1 to 4 | 0 | #NUM! | 0 | Baseline |
| --- | --- | --- | --- | --- | --- |
| Male | 1 to 4 | 0 | #NUM! | 0 | Baseline |
| Male | 1 to 4 | 0 | #NUM! | 0 | Baseline |
| Male | 1 to 4 | 0 | #NUM! | 0 | Baseline |
| Male | 1 to 4 | 0 | #NUM! | 0 | Baseline |
| Male | 1 to 4 | 0 | #NUM! | 0 | Baseline |
| Male | 1 to 4 | 0 | #NUM! | 0 | Baseline |
| Male | 1 to 4 | 0 | #NUM! | 0 | Baseline |
| Male | 1 to 4 | 0 | #NUM! | 0 | Baseline |
| Male | 1 to 4 | 0 | #NUM! | 0 | Baseline |
| Male | 1 to 4 | 0 | #NUM! | 0 | Baseline |
| Male | 1 to 4 | 0 | #NUM! | 0 | Baseline |
| Male | 1 to 4 | 0 | #NUM! | 0 | Baseline |
| Male | 1 to 4 | 0 | #NUM! | 0 | Baseline |
| Male | 1 to 4 | 0 | #NUM! | 0 | Baseline |
| Male | 1 to 4 | 0 | #NUM! | 0 | Baseline |
| Male | 1 to 4 | 0 | #NUM! | 0 | Baseline |
| Male | 1 to 4 | 0 | #NUM! | 0 | Baseline |
| Male | 35+ | 0 | #NUM! | 0 | Baseline |
| Female | 35+ | 0 | #NUM! | 0 | Baseline |
| Female | 22 to 35 | 0 | #NUM! | 0 | Baseline |
| Female | 35+ | 0 | #NUM! | 0 | Baseline |
| Female | 35+ | 0 | #NUM! | 0 | Baseline |
| Female | 22 to 35 | 0 | #NUM! | 0 | Baseline |
| Female | 22 to 35 | 0 | #NUM! | 0 | Baseline |
| Female | 22 to 35 | 0 | #NUM! | 0 | Baseline |
| Male | 35+ | 0 | #NUM! | 0 | Baseline |
| Female | 35+ | 0 | #NUM! | 0 | Baseline |
| Male | 35+ | 0 | #NUM! | 0 | Baseline |
| Male | 35+ | 0 | #NUM! | 0 | Baseline |
| Male | 22 to 35 | 0 | #NUM! | 0 | Baseline |
| Female | 22 to 35 | 0 | #NUM! | 0 | Baseline |
| Female | 22 to 35 | 0 | #NUM! | 0 | Baseline |
| Female | 22 to 35 | 0 | #NUM! | 0 | Baseline |
| Female | 22 to 35 | 0 | #NUM! | 0 | Baseline |
| Male | 35+ | 0 | #NUM! | 0 | Baseline |
| Female | 22 to 35 | 0 | #NUM! | 0 | Baseline |
| Female | 22 to 35 | 0 | #NUM! | 0 | Baseline |
| Female | 22 to 35 | 0 | #NUM! | 0 | Baseline |
| Male | 22 to 35 | 0 | #NUM! | 0 | Baseline |
| Male | 35+ | 0 | #NUM! | 0 | Baseline |
| Female | 35+ | 0 | #NUM! | 0 | Baseline |
| Female | 22 to 35 | 0 | #NUM! | 0 | Baseline |
| Male | 22 to 35 | 0 | #NUM! | 0 | Baseline |
| Female | 35+ | 0 | #NUM! | 0 | Baseline |
| Female | 22 to 35 | 0 | #NUM! | 0 | Baseline |
| Male | 22 to 35 | 0 | #NUM! | 0 | Baseline |
| Female | 35+ | 0 | #NUM! | 0 | Baseline |
| Female | 35+ | 0 | #NUM! | 0 | Baseline |
| Female | 35+ | 0 | #NUM! | 0 | Baseline |

| Male | 35+ | 0 | #NUM! | 0 | Baseline |
| --- | --- | --- | --- | --- | --- |
| Female | 22 to 35 | 0 | #NUM! | 0 | Baseline |
| Female | 22 to 35 | 0 | #NUM! | 0 | Baseline |
| Male | 35+ | 0 | #NUM! | 0 | Baseline |
| Male | 35+ | 0 | #NUM! | 0 | Baseline |
| Female | 35+ | 0 | #NUM! | 0 | Baseline |
| Male | 35+ | 0 | #NUM! | 0 | Baseline |
| Female | 22 to 35 | 0 | #NUM! | 0 | Baseline |
| Female | 15 to 21 | 0 | #NUM! | 0 | Baseline |
| Male | 15 to 21 | 0 | #NUM! | 0 | Baseline |
| Female | 15 to 21 | 0 | #NUM! | 0 | Baseline |
| Female | 5 to 14 | 0 | #NUM! | 0 | Baseline |
| Female | 5 to 14 | 0 | #NUM! | 0 | Baseline |
| Male | 15 to 21 | 0 | #NUM! | 0 | Baseline |
| Male | 1 to 4 | 0 | #NUM! | 0 | Baseline |
| Male | 5 to 14 | 0 | #NUM! | 0 | Baseline |
| Female | 15 to 21 | 0 | #NUM! | 0 | Baseline |
| Male | 1 to 4 | 0 | #NUM! | 0 | Baseline |
| Male | 5 to 14 | 0 | #NUM! | 0 | Baseline |
| Female | 5 to 14 | 0 | #NUM! | 0 | Baseline |
| Male | 15 to 21 | 0 | #NUM! | 0 | Baseline |
| Female | 5 to 14 | 0 | #NUM! | 0 | Baseline |
| Male | 1 to 4 | 0 | #NUM! | 0 | Baseline |
| Female | 1 to 4 | 0 | #NUM! | 0 | Baseline |
| Male | 5 to 14 | 0 | #NUM! | 0 | Baseline |
| Male | 1 to 4 | 0 | #NUM! | 0 | Baseline |
| Female | 5 to 14 | 0 | #NUM! | 0 | Baseline |
| Male | 5 to 14 | 0 | #NUM! | 0 | Baseline |
| Male | 1 to 4 | 0 | #NUM! | 0 | Baseline |
| Female | 5 to 14 | 0 | #NUM! | 0 | Baseline |
| Female | 1 to 4 | 0 | #NUM! | 0 | Baseline |
| Female | 5 to 14 | 0 | #NUM! | 0 | Baseline |
| Male | 1 to 4 | 0 | #NUM! | 0 | Baseline |
| Male | 5 to 14 | 0 | #NUM! | 0 | Baseline |
| Female | 5 to 14 | 0 | #NUM! | 0 | Baseline |
| Male | 5 to 14 | 0 | #NUM! | 0 | Baseline |
| Male | 15 to 21 | 0 | #NUM! | 0 | Baseline |
| Male | 15 to 21 | 0 | #NUM! | 0 | Baseline |
| Female | 15 to 21 | 0 | #NUM! | 0 | Baseline |
| Female | 15 to 21 | 0 | #NUM! | 0 | Baseline |
| Female | 15 to 21 | 0 | #NUM! | 0 | Baseline |
| Male | 5 to 14 | 0 | #NUM! | 0 | Baseline |
| Female | 5 to 14 | 0 | #NUM! | 0 | Baseline |
| Female | 5 to 14 | 0 | #NUM! | 0 | Baseline |
| Male | 5 to 14 | 0 | #NUM! | 0 | Baseline |
| Male | 5 to 14 | 0 | #NUM! | 0 | Baseline |
| Male | 5 to 14 | 0 | #NUM! | 0 | Baseline |
| Male | 15 to 21 | 0 | #NUM! | 0 | Baseline |
| Female | 15 to 21 | 0 | #NUM! | 0 | Baseline |
| Male | 5 to 14 | 0 | #NUM! | 0 | Baseline |

| Female | 5 to 14 | 0 | #NUM! | 0 | Baseline |
| --- | --- | --- | --- | --- | --- |
| Male | 15 to 21 | 0 | #NUM! | 0 | Baseline |
| Male | 1 to 4 | 0 | #NUM! | 0 | Baseline |
| Male | 15 to 21 | 0 | #NUM! | 0 | Baseline |
| Male | 1 to 4 | 0 | #NUM! | 0 | Baseline |
| Female | 5 to 14 | 0 | #NUM! | 0 | Baseline |
| Male | 5 to 14 | 0 | #NUM! | 0 | Baseline |
| Male | 15 to 21 | 0 | #NUM! | 0 | Baseline |
| Female | 1 to 4 | 0 | #NUM! | 0 | Baseline |
| Male | 5 to 14 | 0 | #NUM! | 0 | Baseline |
| Female | 35+ | 0 | #NUM! | 0 | Baseline |
| Male | 22 to 35 | 0 | #NUM! | 0 | Baseline |
| Female | 22 to 35 | 0 | #NUM! | 0 | Baseline |
| Female | 35+ | 0 | #NUM! | 0 | Baseline |
| Female | 22 to 35 | 0 | #NUM! | 0 | Baseline |
| Female | 22 to 35 | 0 | #NUM! | 0 | Baseline |
| Male | 35+ | 0 | #NUM! | 0 | Baseline |
| Female | 35+ | 0 | #NUM! | 0 | Baseline |
| Female | 35+ | 0 | #NUM! | 0 | Baseline |
| Female | 22 to 35 | 0 | #NUM! | 0 | Baseline |
| Female | 35+ | 0 | #NUM! | 0 | Baseline |
| Male | 22 to 35 | 0 | #NUM! | 0 | Baseline |
| Female | 35+ | 0 | #NUM! | 0 | Baseline |
| Female | 35+ | 0 | #NUM! | 0 | Baseline |
| Female | 35+ | 0 | #NUM! | 0 | Baseline |
| Female | 22 to 35 | 0 | #NUM! | 0 | Baseline |
| Male | 35+ | 0 | #NUM! | 0 | Baseline |
| Female | 22 to 35 | 0 | #NUM! | 0 | Baseline |
| Female | 35+ | 0 | #NUM! | 0 | Baseline |
| Female | 35+ | 0 | #NUM! | 0 | Baseline |
| Female | 22 to 35 | 0 | #NUM! | 0 | Baseline |
| Female | 35+ | 0 | #NUM! | 0 | Baseline |
| Female | 35+ | 0 | #NUM! | 0 | Baseline |
| Female | 35+ | 0 | #NUM! | 0 | Baseline |
| Female | 35+ | 0 | #NUM! | 0 | Baseline |
| Female | 22 to 35 | 0 | #NUM! | 0 | Baseline |
| Female | 22 to 35 | 0 | #NUM! | 0 | Baseline |
| Male | 35+ | 0 | #NUM! | 0 | Baseline |
| Female | 22 to 35 | 0 | #NUM! | 0 | Baseline |
| Female | 35+ | 0 | #NUM! | 0 | Baseline |
| Male | 1 to 4 | 0 | #NUM! | 0 | Baseline |
| Male | 1 to 4 | 0 | #NUM! | 0 | Baseline |
| Male | 1 to 4 | 0 | #NUM! | 0 | Baseline |
| Male | 1 to 4 | 0 | #NUM! | 0 | Baseline |
| Male | 1 to 4 | 0 | #NUM! | 0 | Baseline |
| Male | 1 to 4 | 0 | #NUM! | 0 | Baseline |
| Male | 1 to 4 | 0 | #NUM! | 0 | Baseline |
| Male | 1 to 4 | 0 | #NUM! | 0 | Baseline |
| Male | 1 to 4 | 0 | #NUM! | 0 | Baseline |
| Male | 1 to 4 | 0 | #NUM! | 0 | Baseline |

| Male | 1 to 4 | 0 | #NUM! | 0 | Baseline |
| --- | --- | --- | --- | --- | --- |
| Male | 1 to 4 | 0 | #NUM! | 0 | Baseline |
| Male | 1 to 4 | 0 | #NUM! | 0 | Baseline |
| Male | 1 to 4 | 0 | #NUM! | 0 | Baseline |
| Male | 1 to 4 | 0 | #NUM! | 0 | Baseline |
| Male | 1 to 4 | 0 | #NUM! | 0 | Baseline |
| Male | 1 to 4 | 0 | #NUM! | 0 | Baseline |
| Male | 1 to 4 | 0 | #NUM! | 0 | Baseline |
| Male | 1 to 4 | 0 | #NUM! | 0 | Baseline |
| Male | 1 to 4 | 0 | #NUM! | 0 | Baseline |
| Male | 1 to 4 | 0 | #NUM! | 0 | Baseline |
| Male | 1 to 4 | 0 | #NUM! | 0 | Baseline |
| Male | 1 to 4 | 0 | #NUM! | 0 | Baseline |
| Male | 1 to 4 | 0 | #NUM! | 0 | Baseline |
| Male | 1 to 4 | 0 | #NUM! | 0 | Baseline |
| Male | 1 to 4 | 0 | #NUM! | 0 | Baseline |
| Female | 35+ | 0 | #NUM! | 0 | Baseline |
| Male | 1 to 4 | 3 | 0 | 0 | Baseline |
| Male | 1 to 4 | 0 | #NUM! | 0 | Baseline |
| Male | 1 to 4 | 0 | #NUM! | 0 | Baseline |
| Male | 1 to 4 | 0 | #NUM! | 0 | Baseline |
| Male | 1 to 4 | 0 | #NUM! | 0 | Baseline |
| Male | 1 to 4 | 0 | #NUM! | 0 | Baseline |
| Male | 1 to 4 | 0 | #NUM! | 0 | Baseline |
| Male | 1 to 4 | 0 | #NUM! | 0 | Baseline |
| Female | 5 to 14 | 0 | #NUM! | 0 | Baseline |
| Male | 15 to 21 | 0 | #NUM! | 0 | Baseline |
| Male | 22 to 35 | 0 | #NUM! | 0 | Baseline |
| Male | 5 to 14 | 0 | #NUM! | 0 | Baseline |
| Female | 15 to 21 | 0 | #NUM! | 0 | Baseline |
| Male | 22 to 35 | 0 | #NUM! | 0 | Baseline |
| Male | 5 to 14 | 0 | #NUM! | 0 | Baseline |
| Male | 5 to 14 | 0 | #NUM! | 0 | Baseline |
| Male | 15 to 21 | 0 | #NUM! | 0 | Baseline |
| Female | 5 to 14 | 0 | #NUM! | 0 | Baseline |
| Male | 1 to 4 | 0 | #NUM! | 0 | Baseline |
| Male | 1 to 4 | 0 | #NUM! | 0 | Baseline |
| Male | 1 to 4 | 0 | #NUM! | 0 | Baseline |
| Male | 1 to 4 | 0 | #NUM! | 0 | Baseline |
| Male | 1 to 4 | 0 | #NUM! | 0 | Baseline |
| Male | 1 to 4 | 0 | #NUM! | 0 | Baseline |
| Male | 1 to 4 | 0 | #NUM! | 0 | Baseline |
| Male | 1 to 4 | 0 | #NUM! | 0 | Baseline |
| Male | 1 to 4 | 0 | #NUM! | 0 | Baseline |
| Male | 1 to 4 | 0 | #NUM! | 0 | Baseline |
| Male | 1 to 4 | 0 | #NUM! | 0 | Baseline |
| Male | 1 to 4 | 0 | #NUM! | 0 | Baseline |
| Male | 1 to 4 | 0 | #NUM! | 0 | Baseline |
| Male | 1 to 4 | 0 | #NUM! | 0 | Baseline |
| Male | 1 to 4 | 0 | #NUM! | 0 | Baseline |

| Male | 1 to 4 | 0 | #NUM! | 0 | Baseline |
| --- | --- | --- | --- | --- | --- |
| Male | 1 to 4 | 0 | #NUM! | 0 | Baseline |
| Male | 1 to 4 | 0 | #NUM! | 0 | Baseline |
| Male | 1 to 4 | 0 | #NUM! | 0 | Baseline |
| Male | 1 to 4 | 0 | #NUM! | 0 | Baseline |
| Male | 1 to 4 | 0 | #NUM! | 0 | Baseline |
| Male | 1 to 4 | 0 | #NUM! | 0 | Baseline |
| Male | 1 to 4 | 0 | #NUM! | 0 | Baseline |
| Male | 1 to 4 | 0 | #NUM! | 0 | Baseline |
| Male | 1 to 4 | 0 | #NUM! | 0 | Baseline |
| Male | 1 to 4 | 0 | #NUM! | 0 | Baseline |
| Male | 1 to 4 | 0 | #NUM! | 0 | Baseline |
| Male | 1 to 4 | 0 | #NUM! | 0 | Baseline |
| Male | 1 to 4 | 0 | #NUM! | 0 | Baseline |
| Male | 1 to 4 | 0 | #NUM! | 0 | Baseline |
| Male | 1 to 4 | 0 | #NUM! | 0 | Baseline |
| Male | 1 to 4 | 0 | #NUM! | 0 | Baseline |
| Female | 5 to 14 | 0 | #NUM! | 0 | Baseline |
| Male | 1 to 4 | 0 | #NUM! | 0 | Baseline |
| Male | 1 to 4 | 0 | #NUM! | 0 | Baseline |
| Male | 1 to 4 | 0 | #NUM! | 0 | Baseline |
| Male | 1 to 4 | 3 | #NUM! | 0 | Baseline |
| Male | 1 to 4 | 3 | #NUM! | 0 | Baseline |
| Male | 1 to 4 | 3 | #NUM! | 0 | Baseline |
| Male | 1 to 4 | 4 | #NUM! | 0 | Baseline |
| Male | 1 to 4 | 4 | #NUM! | 0 | Baseline |
| Male | 1 to 4 | 0 | #NUM! | 0 | Baseline |
| Male | 1 to 4 | 3 | #NUM! | 0 | Baseline |
| Male | 1 to 4 | 0 | #NUM! | 0 | Baseline |
| Male | 1 to 4 | 0 | #NUM! | 0 | Baseline |
| Male | 1 to 4 | 0 | #NUM! | 0 | Baseline |
| Male | 1 to 4 | 0 | #NUM! | 0 | Baseline |
| Male | 1 to 4 | 0 | #NUM! | 0 | Baseline |
| Male | 1 to 4 | 0 | #NUM! | 0 | Baseline |
| Male | 1 to 4 | 0 | #NUM! | 0 | Baseline |
| Male | 1 to 4 | 0 | #NUM! | 0 | Baseline |
| Male | 1 to 4 | 0 | #NUM! | 0 | Baseline |
| Male | 1 to 4 | 0 | #NUM! | 0 | Baseline |
| Male | 1 to 4 | 0 | #NUM! | 0 | Baseline |
| Male | 1 to 4 | 0 | #NUM! | 0 | Baseline |
| Male | 1 to 4 | 0 | #NUM! | 0 | Baseline |
| Male | 1 to 4 | 0 | #NUM! | 0 | Baseline |
| Male | 1 to 4 | 0 | #NUM! | 0 | Baseline |
| Male | 1 to 4 | 0 | #NUM! | 0 | Baseline |
| Male | 1 to 4 | 0 | #NUM! | 0 | Baseline |
| Male | 1 to 4 | 0 | #NUM! | 0 | Baseline |
| Male | 1 to 4 | 0 | #NUM! | 0 | Baseline |
| Male | 1 to 4 | 0 | #NUM! | 0 | Baseline |
| Male | 1 to 4 | 0 | #NUM! | 0 | Baseline |
| Male | 1 to 4 | 0 | #NUM! | 0 | Baseline |

| Male | 1 to 4 | 0 | #NUM! | 0 | Baseline |
| --- | --- | --- | --- | --- | --- |
| Male | 1 to 4 | 0 | #NUM! | 0 | Baseline |
| Male | 1 to 4 | 0 | #NUM! | 0 | Baseline |
| Male | 1 to 4 | 0 | #NUM! | 0 | Baseline |
| Male | 1 to 4 | 0 | #NUM! | 0 | Baseline |
| Male | 1 to 4 | 0 | #NUM! | 0 | Baseline |
| Male | 1 to 4 | 0 | #NUM! | 0 | Baseline |
| Male | 1 to 4 | 0 | #NUM! | 0 | Baseline |
| Male | 1 to 4 | 0 | #NUM! | 0 | Baseline |
| Male | 1 to 4 | 0 | #NUM! | 0 | Baseline |
| Male | 1 to 4 | 0 | #NUM! | 0 | Baseline |
| Male | 1 to 4 | 0 | #NUM! | 0 | Baseline |
| Male | 1 to 4 | 0 | #NUM! | 0 | Baseline |
| Male | 1 to 4 | 0 | #NUM! | 0 | Baseline |
| Male | 1 to 4 | 0 | #NUM! | 0 | Baseline |
| Male | 1 to 4 | 0 | #NUM! | 0 | Baseline |
| Male | 1 to 4 | 0 | #NUM! | 0 | Baseline |
| Male | 1 to 4 | 0 | #NUM! | 0 | Baseline |
| Male | 1 to 4 | 0 | #NUM! | 0 | Baseline |
| Male | 1 to 4 | 0 | #NUM! | 0 | Baseline |
| Male | 1 to 4 | 0 | #NUM! | 0 | Baseline |
| Male | 1 to 4 | 0 | #NUM! | 0 | Baseline |
| Male | 1 to 4 | 0 | #NUM! | 0 | Baseline |
| Male | 1 to 4 | 0 | #NUM! | 0 | Baseline |
| Male | 1 to 4 | 0 | #NUM! | 0 | Baseline |
| Male | 1 to 4 | 0 | #NUM! | 0 | Baseline |
| Male | 1 to 4 | 0 | #NUM! | 0 | Baseline |
| Male | 1 to 4 | 0 | #NUM! | 0 | Baseline |
| Male | 1 to 4 | 0 | #NUM! | 0 | Baseline |
| Male | 1 to 4 | 0 | #NUM! | 0 | Baseline |
| Male | 1 to 4 | 0 | #NUM! | 0 | Baseline |
| Male | 1 to 4 | 0 | #NUM! | 0 | Baseline |
| Male | 1 to 4 | 0 | #NUM! | 0 | Baseline |
| Male | 1 to 4 | 0 | #NUM! | 0 | Baseline |
| Male | 1 to 4 | 0 | #NUM! | 0 | Baseline |
| Male | 1 to 4 | 0 | #NUM! | 0 | Baseline |
| Male | 1 to 4 | 0 | #NUM! | 0 | Baseline |
| Male | 1 to 4 | 0 | #NUM! | 0 | Baseline |
| Male | 1 to 4 | 0 | #NUM! | 0 | Baseline |
| Male | 1 to 4 | 0 | #NUM! | 0 | Baseline |
| Male | 1 to 4 | 0 | #NUM! | 0 | Baseline |
| Male | 1 to 4 | 0 | #NUM! | 0 | Baseline |
| Male | 1 to 4 | 0 | #NUM! | 0 | Baseline |
| Male | 1 to 4 | 0 | #NUM! | 0 | Baseline |
| Male | 1 to 4 | 0 | #NUM! | 0 | Baseline |
| Male | 1 to 4 | 0 | #NUM! | 0 | Baseline |
| Male | 1 to 4 | 0 | #NUM! | 0 | Baseline |
| Male | 1 to 4 | 0 | #NUM! | 0 | Baseline |
| Male | 1 to 4 | 0 | #NUM! | 0 | Baseline |
| Male | 1 to 4 | 0 | #NUM! | 0 | Baseline |

| Male | 1 to 4 | 0 | #NUM! | 0 | Baseline |
| --- | --- | --- | --- | --- | --- |
| Male | 1 to 4 | 0 | #NUM! | 0 | Baseline |
| Male | 1 to 4 | 0 | #NUM! | 0 | Baseline |
| Male | 1 to 4 | 0 | #NUM! | 0 | Baseline |
| Male | 1 to 4 | 0 | #NUM! | 0 | Baseline |
| Male | 1 to 4 | 0 | #NUM! | 0 | Baseline |
| Male | 1 to 4 | 0 | #NUM! | 0 | Baseline |
| Male | 1 to 4 | 0 | #NUM! | 0 | Baseline |
| Male | 1 to 4 | 0 | #NUM! | 0 | Baseline |
| Male | 1 to 4 | 0 | #NUM! | 0 | Baseline |
| Male | 1 to 4 | 0 | #NUM! | 0 | Baseline |
| Male | 1 to 4 | 0 | #NUM! | 0 | Baseline |
| Male | 1 to 4 | 0 | #NUM! | 0 | Baseline |
| Male | 1 to 4 | 0 | #NUM! | 0 | Baseline |
| Male | 1 to 4 | 0 | #NUM! | 0 | Baseline |
| Male | 1 to 4 | 0 | #NUM! | 0 | Baseline |
| Male | 1 to 4 | 0 | #NUM! | 0 | Baseline |
| Male | 1 to 4 | 0 | #NUM! | 0 | Baseline |
| Male | 1 to 4 | 0 | #NUM! | 0 | Baseline |
| Male | 1 to 4 | 0 | #NUM! | 0 | Baseline |
| Male | 1 to 4 | 0 | #NUM! | 0 | Baseline |
| Male | 1 to 4 | 0 | #NUM! | 0 | Baseline |
| Male | 1 to 4 | 0 | #NUM! | 0 | Baseline |
| Male | 1 to 4 | 0 | #NUM! | 0 | Baseline |
| Male | 1 to 4 | 0 | #NUM! | 0 | Baseline |
| Male | 1 to 4 | 0 | #NUM! | 0 | Baseline |
| Male | 1 to 4 | 0 | #NUM! | 0 | Baseline |
| Male | 1 to 4 | 0 | #NUM! | 0 | Baseline |
| Male | 1 to 4 | 0 | #NUM! | 0 | Baseline |
| Male | 1 to 4 | 0 | #NUM! | 0 | Baseline |
| Male | 1 to 4 | 0 | #NUM! | 0 | Baseline |
| Male | 1 to 4 | 0 | #NUM! | 0 | Baseline |
| Male | 1 to 4 | 0 | #NUM! | 0 | Baseline |
| Male | 1 to 4 | 0 | #NUM! | 0 | Baseline |
| Male | 1 to 4 | 0 | #NUM! | 0 | Baseline |
| Male | 1 to 4 | 0 | #NUM! | 0 | Baseline |
| Male | 1 to 4 | 0 | #NUM! | 0 | Baseline |
| Male | 1 to 4 | 0 | #NUM! | 0 | Baseline |
| Male | 1 to 4 | 0 | #NUM! | 0 | Baseline |
| Male | 1 to 4 | 0 | #NUM! | 0 | Baseline |
| Male | 1 to 4 | 0 | #NUM! | 0 | Baseline |
| Male | 1 to 4 | 0 | #NUM! | 0 | Baseline |
| Male | 1 to 4 | 0 | #NUM! | 0 | Baseline |
| Male | 1 to 4 | 0 | #NUM! | 0 | Baseline |
| Male | 1 to 4 | 0 | #NUM! | 0 | Baseline |
| Male | 1 to 4 | 0 | #NUM! | 0 | Baseline |
| Male | 1 to 4 | 0 | #NUM! | 0 | Baseline |
| Male | 1 to 4 | 0 | #NUM! | 0 | Baseline |
| Male | 1 to 4 | 0 | #NUM! | 0 | Baseline |
| Male | 1 to 4 | 0 | #NUM! | 0 | Baseline |

| Male | 1 to 4 | 0 | #NUM! | 0 | Baseline |
| --- | --- | --- | --- | --- | --- |
| Male | 1 to 4 | 0 | #NUM! | 0 | Baseline |
| Male | 1 to 4 | 0 | #NUM! | 0 | Baseline |
| Male | 1 to 4 | 0 | #NUM! | 0 | Baseline |
| Male | 1 to 4 | 0 | #NUM! | 0 | Baseline |
| Male | 1 to 4 | 0 | #NUM! | 0 | Baseline |
| Male | 1 to 4 | 0 | #NUM! | 0 | Baseline |
| Male | 1 to 4 | 0 | #NUM! | 0 | Baseline |
| Male | 1 to 4 | 0 | #NUM! | 0 | Baseline |
| Male | 1 to 4 | 0 | #NUM! | 0 | Baseline |
| Male | 1 to 4 | 0 | #NUM! | 0 | Baseline |
| Male | 1 to 4 | 0 | #NUM! | 0 | Baseline |
| Male | 35+ | 0 | #NUM! | 0 | Baseline |
| Male | 22 to 35 | 0 | #NUM! | 0 | Baseline |
| Male | 1 to 4 | 0 | #NUM! | 0 | Baseline |
| Male | 1 to 4 | 0 | #NUM! | 0 | Baseline |
| Male | 1 to 4 | 0 | #NUM! | 0 | Baseline |
| Male | 1 to 4 | 0 | #NUM! | 0 | Baseline |
| Male | 1 to 4 | 0 | #NUM! | 0 | Baseline |
| Male | 1 to 4 | 0 | #NUM! | 0 | Baseline |
| Male | 1 to 4 | 0 | #NUM! | 0 | Baseline |
| Male | 1 to 4 | 0 | #NUM! | 0 | Baseline |
| Male | 1 to 4 | 0 | #NUM! | 0 | Baseline |
| Male | 1 to 4 | 0 | #NUM! | 0 | Baseline |
| Male | 1 to 4 | 0 | #NUM! | 0 | Baseline |
| Male | 1 to 4 | 0 | #NUM! | 0 | Baseline |
| Male | 1 to 4 | 0 | #NUM! | 0 | Baseline |
| Male | 1 to 4 | 0 | #NUM! | 0 | Baseline |
| Male | 1 to 4 | 0 | #NUM! | 0 | Baseline |
| Male | 1 to 4 | 0 | #NUM! | 0 | Baseline |
| Male | 1 to 4 | 0 | #NUM! | 0 | Baseline |
| Male | 1 to 4 | 0 | #NUM! | 0 | Baseline |
| Male | 1 to 4 | 0 | #NUM! | 0 | Baseline |
| Male | 1 to 4 | 0 | #NUM! | 0 | Baseline |
| Male | 1 to 4 | 0 | #NUM! | 0 | Baseline |
| Male | 1 to 4 | 0 | #NUM! | 0 | Baseline |
| Male | 1 to 4 | 0 | #NUM! | 0 | Baseline |
| Male | 1 to 4 | 0 | #NUM! | 0 | Baseline |
| Male | 1 to 4 | 0 | #NUM! | 0 | Baseline |
| Male | 1 to 4 | 0 | #NUM! | 0 | Baseline |
| Male | 1 to 4 | 0 | #NUM! | 0 | Baseline |
| Male | 1 to 4 | 0 | #NUM! | 0 | Baseline |
| Male | 1 to 4 | 0 | #NUM! | 0 | Baseline |
| Male | 1 to 4 | 0 | #NUM! | 0 | Baseline |
| Male | 1 to 4 | 0 | #NUM! | 0 | Baseline |
| Male | 1 to 4 | 0 | #NUM! | 0 | Baseline |
| Male | 1 to 4 | 0 | #NUM! | 0 | Baseline |
| Male | 1 to 4 | 0 | #NUM! | 0 | Baseline |
| Male | 1 to 4 | 0 | #NUM! | 0 | Baseline |
| Male | 1 to 4 | 0 | #NUM! | 0 | Baseline |

| Male | 1 to 4 | 0 | #NUM! | 0 | Baseline |
| --- | --- | --- | --- | --- | --- |
| Male | 1 to 4 | 0 | #NUM! | 0 | Baseline |
| Male | 1 to 4 | 0 | #NUM! | 0 | Baseline |
| Male | 1 to 4 | 0 | #NUM! | 0 | Baseline |
| Male | 1 to 4 | 0 | #NUM! | 0 | Baseline |
| Male | 1 to 4 | 0 | #NUM! | 0 | Baseline |
| Female | 22 to 35 | 1 | 0 | 0 | Baseline |
| Male | 22 to 35 | 1 | 0 | 0 | Baseline |
| Male | 1 to 4 | 1 | 0 | 0 | Baseline |
| Male | 35+ | 1 | 0 | 0 | Baseline |
| Male | 15 to 21 | 1 | 0 | 0 | Baseline |
| Female | 22 to 35 | 1 | 0 | 0 | Baseline |
| Female | 22 to 35 | 1 | 0 | 0 | Baseline |
| Female | 22 to 35 | 1 | 0 | 0 | Baseline |
| Male | 1 to 4 | 1 | 0 | 0 | Baseline |
| Male | 1 to 4 | 0 | #NUM! | 0 | Baseline |
| Male | 1 to 4 | 1 | 0 | 0 | Baseline |
| Male | 15 to 21 | 0 | #NUM! | 0 | Baseline |
| Male | 1 to 4 | 0 | #NUM! | 0 | Baseline |
| Male | 1 to 4 | 0 | #NUM! | 0 | Baseline |
| Male | 1 to 4 | 0 | #NUM! | 0 | Baseline |
| Male | 1 to 4 | 0 | #NUM! | 0 | Baseline |
| Male | 1 to 4 | 0 | #NUM! | 0 | Baseline |
| Male | 1 to 4 | 0 | #NUM! | 0 | Baseline |
| Male | 1 to 4 | 0 | #NUM! | 0 | Baseline |
| Male | 1 to 4 | 0 | #NUM! | 0 | Baseline |
| Male | 1 to 4 | 0 | #NUM! | 0 | Baseline |
| Male | 1 to 4 | 0 | #NUM! | 0 | Baseline |
| Male | 1 to 4 | 0 | #NUM! | 0 | Baseline |
| Male | 1 to 4 | 0 | #NUM! | 0 | Baseline |
| Male | 1 to 4 | 0 | #NUM! | 0 | Baseline |
| Male | 1 to 4 | 0 | #NUM! | 0 | Baseline |
| Male | 1 to 4 | 0 | #NUM! | 0 | Baseline |
| Male | 1 to 4 | 0 | #NUM! | 0 | Baseline |
| Male | 1 to 4 | 0 | #NUM! | 0 | Baseline |
| Male | 1 to 4 | 0 | #NUM! | 0 | Baseline |
| Male | 1 to 4 | 0 | #NUM! | 0 | Baseline |
| Male | 1 to 4 | 0 | #NUM! | 0 | Baseline |
| Male | 1 to 4 | 0 | #NUM! | 0 | Baseline |
| Male | 1 to 4 | 0 | #NUM! | 0 | Baseline |
| Male | 1 to 4 | 0 | #NUM! | 0 | Baseline |
| Male | 1 to 4 | 0 | #NUM! | 0 | Baseline |
| Male | 1 to 4 | 0 | #NUM! | 0 | Baseline |
| Male | 1 to 4 | 0 | #NUM! | 0 | Baseline |
| Male | 1 to 4 | 0 | #NUM! | 0 | Baseline |
| Male | 1 to 4 | 0 | #NUM! | 0 | Baseline |
| Male | 1 to 4 | 0 | #NUM! | 0 | Baseline |
| Male | 1 to 4 | 0 | #NUM! | 0 | Baseline |
| Male | 1 to 4 | 0 | #NUM! | 0 | Baseline |
| Male | 1 to 4 | 0 | #NUM! | 0 | Baseline |

| Male | 1 to 4 | 0 | #NUM! | 0 | Baseline |
| --- | --- | --- | --- | --- | --- |
| Male | 1 to 4 | 0 | #NUM! | 0 | Baseline |
| Male | 1 to 4 | 0 | #NUM! | 0 | Baseline |
| Male | 1 to 4 | 0 | #NUM! | 0 | Baseline |
| Male | 1 to 4 | 0 | #NUM! | 0 | Baseline |
| Male | 1 to 4 | 0 | #NUM! | 0 | Baseline |
| Male | 1 to 4 | 0 | #NUM! | 0 | Baseline |
| Male | 1 to 4 | 0 | #NUM! | 0 | Baseline |
| Male | 1 to 4 | 0 | #NUM! | 0 | Baseline |
| Male | 1 to 4 | 0 | #NUM! | 0 | Baseline |
| Male | 1 to 4 | 0 | #NUM! | 0 | Baseline |
| Male | 1 to 4 | 0 | #NUM! | 0 | Baseline |
| Male | 1 to 4 | 0 | #NUM! | 0 | Baseline |
| Male | 1 to 4 | 0 | #NUM! | 0 | Baseline |
| Male | 1 to 4 | 0 | #NUM! | 0 | Baseline |
| Male | 1 to 4 | 0 | #NUM! | 0 | Baseline |
| Male | 1 to 4 | 0 | #NUM! | 0 | Baseline |
| Male | 1 to 4 | 0 | #NUM! | 0 | Baseline |
| Male | 1 to 4 | 0 | #NUM! | 0 | Baseline |
| Male | 1 to 4 | 0 | #NUM! | 0 | Baseline |
| Male | 1 to 4 | 0 | #NUM! | 0 | Baseline |
| Male | 1 to 4 | 0 | #NUM! | 0 | Baseline |
| Male | 1 to 4 | 0 | #NUM! | 0 | Baseline |
| Male | 1 to 4 | 0 | #NUM! | 0 | Baseline |
| Male | 1 to 4 | 0 | #NUM! | 0 | Baseline |
| Male | 1 to 4 | 0 | #NUM! | 0 | Baseline |
| Male | 1 to 4 | 0 | #NUM! | 0 | Baseline |
| Male | 1 to 4 | 0 | #NUM! | 0 | Baseline |
| Male | 1 to 4 | 0 | #NUM! | 0 | Baseline |
| Male | 1 to 4 | 0 | #NUM! | 0 | Baseline |
| Male | 1 to 4 | 0 | #NUM! | 0 | Baseline |
| Male | 1 to 4 | 0 | #NUM! | 0 | Baseline |
| Male | 1 to 4 | 0 | #NUM! | 0 | Baseline |
| Male | 1 to 4 | 0 | #NUM! | 0 | Baseline |
| Male | 1 to 4 | 0 | #NUM! | 0 | Baseline |
| Male | 1 to 4 | 0 | #NUM! | 0 | Baseline |
| Male | 1 to 4 | 0 | #NUM! | 0 | Baseline |
| Male | 1 to 4 | 0 | #NUM! | 0 | Baseline |
| Male | 1 to 4 | 0 | #NUM! | 0 | Baseline |
| Male | 1 to 4 | 0 | #NUM! | 0 | Baseline |
| Male | 1 to 4 | 0 | #NUM! | 0 | Baseline |
| Male | 1 to 4 | 0 | #NUM! | 0 | Baseline |
| Male | 1 to 4 | 0 | #NUM! | 0 | Baseline |
| Male | 1 to 4 | 0 | #NUM! | 0 | Baseline |
| Male | 1 to 4 | 0 | #NUM! | 0 | Baseline |
| Male | 1 to 4 | 0 | #NUM! | 0 | Baseline |
| Male | 1 to 4 | 0 | #NUM! | 0 | Baseline |
| Male | 1 to 4 | 0 | #NUM! | 0 | Baseline |
| Male | 1 to 4 | 0 | #NUM! | 0 | Baseline |
| Male | 1 to 4 | 0 | #NUM! | 0 | Baseline |

| Male | 1 to 4 | 0 | #NUM! | 0 | Baseline |
| --- | --- | --- | --- | --- | --- |
| Male | 1 to 4 | 0 | #NUM! | 0 | Baseline |
| Male | 1 to 4 | 0 | #NUM! | 0 | Baseline |
| Male | 1 to 4 | 0 | #NUM! | 0 | Baseline |
| Male | 1 to 4 | 0 | #NUM! | 0 | Baseline |
| Male | 1 to 4 | 0 | #NUM! | 0 | Baseline |
| Female | 22 to 35 | 0 | #NUM! | 0 | Baseline |
| Male | 1 to 4 | 0 | #NUM! | 0 | Baseline |
| Male | 22 to 35 | 0 | #NUM! | 0 | Baseline |
| Male | 1 to 4 | 0 | #NUM! | 0 | Baseline |
| Female | 35+ | 0 | #NUM! | 0 | Baseline |
| Male | 1 to 4 | 0 | #NUM! | 0 | Baseline |
| Male | 22 to 35 | 0 | #NUM! | 0 | Baseline |
| Male | 35+ | 0 | #NUM! | 0 | Baseline |
| Female | 15 to 21 | 0 | #NUM! | 0 | Baseline |
| Male | 1 to 4 | 0 | #NUM! | 0 | Baseline |
| Male | 22 to 35 | 0 | #NUM! | 0 | Baseline |
| Male | 22 to 35 | 0 | #NUM! | 0 | Baseline |
| Female | 22 to 35 | 0 | #NUM! | 0 | Baseline |
| Female | 15 to 21 | 0 | #NUM! | 0 | Baseline |
| Female | 22 to 35 | 0 | #NUM! | 0 | Baseline |
| Male | 22 to 35 | 0 | #NUM! | 0 | Baseline |
| Male | 22 to 35 | 0 | #NUM! | 0 | Baseline |
| Female | 15 to 21 | 0 | #NUM! | 0 | Baseline |
| Male | 1 to 4 | 0 | #NUM! | 0 | Baseline |
| Female | 22 to 35 | 0 | #NUM! | 0 | Baseline |
| Female | 22 to 35 | 0 | #NUM! | 0 | Baseline |
| Male | 22 to 35 | 0 | #NUM! | 0 | Baseline |
| Male | 22 to 35 | 0 | #NUM! | 0 | Baseline |
| Male | 35+ | 0 | #NUM! | 0 | Baseline |
| Female | 15 to 21 | 0 | #NUM! | 0 | Baseline |
| Male | 15 to 21 | 0 | #NUM! | 0 | Baseline |
| Male | 15 to 21 | 0 | #NUM! | 0 | Baseline |
| Male | 1 to 4 | 0 | #NUM! | 0 | Baseline |
| Male | 1 to 4 | 0 | #NUM! | 0 | Baseline |
| Male | 1 to 4 | 0 | #NUM! | 0 | Baseline |
| Male | 1 to 4 | 0 | #NUM! | 0 | Baseline |
| Male | 1 to 4 | 0 | #NUM! | 0 | Baseline |
| Female | 22 to 35 | 0 | #NUM! | 0 | Baseline |
| Male | 1 to 4 | 0 | #NUM! | 0 | Baseline |
| Male | 1 to 4 | 0 | #NUM! | 0 | Baseline |
| Male | 1 to 4 | 0 | #NUM! | 0 | Baseline |
| Male | 1 to 4 | 0 | #NUM! | 0 | Baseline |
| Male | 1 to 4 | 0 | #NUM! | 0 | Baseline |
| Male | 1 to 4 | 0 | #NUM! | 0 | Baseline |
| Male | 1 to 4 | 0 | #NUM! | 0 | Baseline |
| Male | 1 to 4 | 0 | #NUM! | 0 | Baseline |
| Male | 1 to 4 | 0 | #NUM! | 0 | Baseline |
| Male | 1 to 4 | 0 | #NUM! | 0 | Baseline |
| Male | 1 to 4 | 0 | #NUM! | 0 | Baseline |

| Male | 1 to 4 | 0 | #NUM! | 0 | Baseline |
| --- | --- | --- | --- | --- | --- |
| Male | 1 to 4 | 0 | #NUM! | 0 | Baseline |
| Male | 1 to 4 | 0 | #NUM! | 0 | Baseline |
| Male | 1 to 4 | 0 | #NUM! | 0 | Baseline |
| Female | 15 to 21 | 0 | #NUM! | 0 | Baseline |
| Male | 1 to 4 | 0 | #NUM! | 0 | Baseline |
| Male | 1 to 4 | 0 | #NUM! | 0 | Baseline |
| Male | 1 to 4 | 0 | #NUM! | 0 | Baseline |
| Male | 1 to 4 | 0 | #NUM! | 0 | Baseline |
| Male | 1 to 4 | 0 | #NUM! | 0 | Baseline |
| Female | 22 to 35 | 0 | #NUM! | 0 | Baseline |
| Female | 22 to 35 | 0 | #NUM! | 0 | Baseline |
| Male | 35+ | 0 | #NUM! | 0 | Baseline |
| Male | 1 to 4 | 0 | #NUM! | 0 | Baseline |
| Male | 22 to 35 | 0 | #NUM! | 0 | Baseline |
| Male | 5 to 14 | 0 | #NUM! | 0 | Baseline |
| Male | 1 to 4 | 0 | #NUM! | 0 | Baseline |
| Male | 1 to 4 | 0 | #NUM! | 0 | Baseline |
| Male | 1 to 4 | 0 | #NUM! | 0 | Baseline |
| Male | 1 to 4 | 0 | #NUM! | 0 | Baseline |
| Male | 1 to 4 | 0 | #NUM! | 0 | Baseline |
| Male | 1 to 4 | 0 | #NUM! | 0 | Baseline |
| Male | 1 to 4 | 0 | #NUM! | 0 | Baseline |
| Male | 1 to 4 | 0 | #NUM! | 0 | Baseline |
| Male | 1 to 4 | 0 | #NUM! | 0 | Baseline |
| Male | 5 to 14 | 0 | #NUM! | 0 | Baseline |
| Male | 5 to 14 | 0 | #NUM! | 0 | Baseline |
| Male | 5 to 14 | 0 | #NUM! | 0 | Baseline |
| Male | 1 to 4 | 0 | #NUM! | 0 | Baseline |
| Male | 5 to 14 | 0 | #NUM! | 0 | Baseline |
| Female | 5 to 14 | 0 | #NUM! | 0 | Baseline |
| Female | 1 to 4 | 0 | #NUM! | 0 | Baseline |
| Female | 5 to 14 | 0 | #NUM! | 0 | Baseline |
| Female | 5 to 14 | 0 | #NUM! | 0 | Baseline |
| Female | 1 to 4 | 0 | #NUM! | 0 | Baseline |
| Female | 5 to 14 | 0 | #NUM! | 0 | Baseline |
| Male | 15 to 21 | 0 | #NUM! | 0 | Baseline |
| Male | 15 to 21 | 0 | #NUM! | 0 | Baseline |
| Male | 15 to 21 | 0 | #NUM! | 0 | Baseline |
| Male | 15 to 21 | 0 | #NUM! | 0 | Baseline |
| Male | 15 to 21 | 0 | #NUM! | 0 | Baseline |
| Male | 15 to 21 | 0 | #NUM! | 0 | Baseline |
| Male | 15 to 21 | 0 | #NUM! | 0 | Baseline |
| Male | 15 to 21 | 0 | #NUM! | 0 | Baseline |
| Male | 5 to 14 | 0 | #NUM! | 0 | Baseline |
| Male | 5 to 14 | 0 | #NUM! | 0 | Baseline |
| Male | 5 to 14 | 0 | #NUM! | 0 | Baseline |
| Female | 15 to 21 | 0 | #NUM! | 0 | Baseline |
| Female | 15 to 21 | 0 | #NUM! | 0 | Baseline |
| Female | 15 to 21 | 0 | #NUM! | 0 | Baseline |

| Female | 15 to 21 | 0 | #NUM! | 0 | Baseline |
| --- | --- | --- | --- | --- | --- |
| Female | 15 to 21 | 0 | #NUM! | 0 | Baseline |
| Female | 5 to 14 | 0 | #NUM! | 0 | Baseline |
| Female | 5 to 14 | 0 | #NUM! | 0 | Baseline |
| Male | 1 to 4 | 0 | #NUM! | 0 | Baseline |
| Female | 15 to 21 | 0 | #NUM! | 0 | Baseline |
| Female | 15 to 21 | 0 | #NUM! | 0 | Baseline |
| Female | 5 to 14 | 0 | #NUM! | 0 | Baseline |
| Female | 5 to 14 | 0 | #NUM! | 0 | Baseline |
| Female | 5 to 14 | 0 | #NUM! | 0 | Baseline |
| Female | 5 to 14 | 0 | #NUM! | 0 | Baseline |
| Female | 5 to 14 | 0 | #NUM! | 0 | Baseline |
| Female | 5 to 14 | 0 | #NUM! | 0 | Baseline |
| Male | 5 to 14 | 0 | #NUM! | 0 | Baseline |
| Male | 5 to 14 | 0 | #NUM! | 0 | Baseline |
| Male | 15 to 21 | 0 | #NUM! | 0 | Baseline |
| Male | 15 to 21 | 0 | #NUM! | 0 | Baseline |
| Female | 5 to 14 | 0 | #NUM! | 0 | Baseline |
| Female | 22 to 35 | 0 | #NUM! | 0 | Baseline |
| Female | 22 to 35 | 0 | #NUM! | 0 | Baseline |
| Female | 22 to 35 | 0 | #NUM! | 0 | Baseline |
| Female | 22 to 35 | 0 | #NUM! | 0 | Baseline |
| Male | 22 to 35 | 0 | #NUM! | 0 | Baseline |
| Male | 22 to 35 | 0 | #NUM! | 0 | Baseline |
| Male | 22 to 35 | 0 | #NUM! | 0 | Baseline |
| Female | 22 to 35 | 0 | #NUM! | 0 | Baseline |
| Male | 22 to 35 | 0 | #NUM! | 0 | Baseline |
| Female | 22 to 35 | 0 | #NUM! | 0 | Baseline |
| Female | 22 to 35 | 0 | #NUM! | 0 | Baseline |
| Male | 35+ | 0 | #NUM! | 0 | Baseline |
| Female | 22 to 35 | 0 | #NUM! | 0 | Baseline |
| Male | 22 to 35 | 0 | #NUM! | 0 | Baseline |
| Male | 22 to 35 | 0 | #NUM! | 0 | Baseline |
| Male | 22 to 35 | 0 | #NUM! | 0 | Baseline |
| Male | 22 to 35 | 0 | #NUM! | 0 | Baseline |
| Female | 22 to 35 | 0 | #NUM! | 0 | Baseline |
| Female | 22 to 35 | 0 | #NUM! | 0 | Baseline |
| Male | 35+ | 0 | #NUM! | 0 | Baseline |
| Female | 35+ | 0 | #NUM! | 0 | Baseline |
| Male | 35+ | 0 | #NUM! | 0 | Baseline |
| Male | 35+ | 0 | #NUM! | 0 | Baseline |
| Female | 35+ | 0 | #NUM! | 0 | Baseline |
| Female | 35+ | 0 | #NUM! | 0 | Baseline |
| Female | 35+ | 0 | #NUM! | 0 | Baseline |
| Female | 35+ | 0 | #NUM! | 0 | Baseline |
| Male | 35+ | 0 | #NUM! | 0 | Baseline |
| Female | 35+ | 0 | #NUM! | 0 | Baseline |
| Female | 35+ | 0 | #NUM! | 0 | Baseline |
| Male | 35+ | 0 | #NUM! | 0 | Baseline |
| Female | 35+ | 0 | #NUM! | 0 | Baseline |

| Male | 35+ | 0 | #NUM! | 0 | Baseline |
| --- | --- | --- | --- | --- | --- |
| Male | 35+ | 0 | #NUM! | 0 | Baseline |
| Male | 35+ | 0 | #NUM! | 0 | Baseline |
| Male | 35+ | 0 | #NUM! | 0 | Baseline |
| Male | 35+ | 0 | #NUM! | 0 | Baseline |
| Male | 35+ | 0 | #NUM! | 0 | Baseline |
| Female | 35+ | 0 | #NUM! | 0 | Baseline |
| Female | 22 to 35 | 0 | #NUM! | 0 | Baseline |
| Female | 22 to 35 | 0 | #NUM! | 0 | Baseline |
| Male | 22 to 35 | 0 | #NUM! | 0 | Baseline |
| Female | 35+ | 0 | #NUM! | 0 | Baseline |
| Female | 22 to 35 | 0 | #NUM! | 0 | Baseline |
| Female | 22 to 35 | 0 | #NUM! | 0 | Baseline |
| Male | 1 to 4 | 0 | #NUM! | 0 | Baseline |
| Female | 35+ | 0 | #NUM! | 0 | Baseline |
| Female | 22 to 35 | 0 | #NUM! | 0 | Baseline |
| Female | 22 to 35 | 0 | #NUM! | 0 | Baseline |
| Female | 15 to 21 | 0 | #NUM! | 0 | Baseline |
| Male | 15 to 21 | 0 | #NUM! | 0 | Baseline |
| Female | 15 to 21 | 0 | #NUM! | 0 | Baseline |
| Female | 15 to 21 | 0 | #NUM! | 0 | Baseline |
| Female | 5 to 14 | 0 | #NUM! | 0 | Baseline |
| Female | 5 to 14 | 0 | #NUM! | 0 | Baseline |
| Female | 15 to 21 | 0 | #NUM! | 0 | Baseline |
| Female | 5 to 14 | 0 | #NUM! | 0 | Baseline |
| Female | 15 to 21 | 0 | #NUM! | 0 | Baseline |
| Male | 5 to 14 | 0 | #NUM! | 0 | Baseline |
| Male | 5 to 14 | 0 | #NUM! | 0 | Baseline |
| Female | 5 to 14 | 0 | #NUM! | 0 | Baseline |
| Female | 15 to 21 | 0 | #NUM! | 0 | Baseline |
| Female | 15 to 21 | 0 | #NUM! | 0 | Baseline |
| Female | 15 to 21 | 0 | #NUM! | 0 | Baseline |
| Male | 15 to 21 | 0 | #NUM! | 0 | Baseline |
| Male | 15 to 21 | 0 | #NUM! | 0 | Baseline |
| Male | 5 to 14 | 0 | #NUM! | 0 | Baseline |
| Male | 5 to 14 | 0 | #NUM! | 0 | Baseline |
| Male | 5 to 14 | 0 | #NUM! | 0 | Baseline |
| Male | 15 to 21 | 0 | #NUM! | 0 | Baseline |
| Female | 5 to 14 | 0 | #NUM! | 0 | Baseline |
| Male | 15 to 21 | 0 | #NUM! | 0 | Baseline |
| Male | 15 to 21 | 0 | #NUM! | 0 | Baseline |
| Male | 5 to 14 | 0 | #NUM! | 0 | Baseline |
| Male | 5 to 14 | 0 | #NUM! | 0 | Baseline |
| Male | 5 to 14 | 0 | #NUM! | 0 | Baseline |
| Male | 5 to 14 | 0 | #NUM! | 0 | Baseline |
| Male | 5 to 14 | 0 | #NUM! | 0 | Baseline |
| Male | 5 to 14 | 0 | #NUM! | 0 | Baseline |
| Male | 5 to 14 | 0 | #NUM! | 0 | Baseline |
| Male | 5 to 14 | 0 | #NUM! | 0 | Baseline |
| Female | 5 to 14 | 0 | #NUM! | 0 | Baseline |

| Female | 5 to 14 | 0 | #NUM! | 0 | Baseline |
| --- | --- | --- | --- | --- | --- |
| Female | 5 to 14 | 0 | #NUM! | 0 | Baseline |
| Female | 5 to 14 | 0 | #NUM! | 0 | Baseline |
| Female | 5 to 14 | 0 | #NUM! | 0 | Baseline |
| Female | 1 to 4 | 0 | #NUM! | 0 | Baseline |
| Female | 5 to 14 | 0 | #NUM! | 0 | Baseline |
| Female | 5 to 14 | 0 | #NUM! | 0 | Baseline |
| Female | 5 to 14 | 0 | #NUM! | 0 | Baseline |
| Male | 5 to 14 | 0 | #NUM! | 0 | Baseline |
| Male | 5 to 14 | 0 | #NUM! | 0 | Baseline |
| Male | 5 to 14 | 0 | #NUM! | 0 | Baseline |
| Female | 5 to 14 | 0 | #NUM! | 0 | Baseline |
| Male | 5 to 14 | 0 | #NUM! | 0 | Baseline |
| Male | 5 to 14 | 0 | #NUM! | 0 | Baseline |
| Male | 5 to 14 | 0 | #NUM! | 0 | Baseline |
| Male | 5 to 14 | 0 | #NUM! | 0 | Baseline |
| Female | 5 to 14 | 0 | #NUM! | 0 | Baseline |
| Female | 5 to 14 | 0 | #NUM! | 0 | Baseline |
| Female | 5 to 14 | 0 | #NUM! | 0 | Baseline |
| Female | 5 to 14 | 0 | #NUM! | 0 | Baseline |
| Female | 5 to 14 | 0 | #NUM! | 0 | Baseline |
| Male | 5 to 14 | 0 | #NUM! | 0 | Baseline |
| Male | 1 to 4 | 0 | #NUM! | 0 | Baseline |
| Female | 22 to 35 | 0 | #NUM! | 0 | Baseline |
| Female | 22 to 35 | 0 | #NUM! | 0 | Baseline |
| Female | 35+ | 0 | #NUM! | 0 | Baseline |
| Male | 22 to 35 | 0 | #NUM! | 0 | Baseline |
| Male | 35+ | 0 | #NUM! | 0 | Baseline |
| Male | 22 to 35 | 0 | #NUM! | 0 | Baseline |
| Female | 22 to 35 | 0 | #NUM! | 0 | Baseline |
| Male | 35+ | 0 | #NUM! | 0 | Baseline |
| Male | 22 to 35 | 0 | #NUM! | 0 | Baseline |
| Female | 35+ | 0 | #NUM! | 0 | Baseline |
| Male | 35+ | 0 | #NUM! | 0 | Baseline |
| Male | 35+ | 0 | #NUM! | 0 | Baseline |
| Male | 35+ | 0 | #NUM! | 0 | Baseline |
| Male | 22 to 35 | 0 | #NUM! | 0 | Baseline |
| Male | 35+ | 0 | #NUM! | 0 | Baseline |
| Male | 35+ | 0 | #NUM! | 0 | Baseline |
| Male | 35+ | 0 | #NUM! | 0 | Baseline |
| Male | 35+ | 0 | #NUM! | 0 | Baseline |
| Male | 22 to 35 | 0 | #NUM! | 0 | Baseline |
| Male | 35+ | 0 | #NUM! | 0 | Baseline |
| Female | 35+ | 0 | #NUM! | 0 | Baseline |
| Female | 35+ | 0 | #NUM! | 0 | Baseline |
| Female | 22 to 35 | 0 | #NUM! | 0 | Baseline |
| Male | 35+ | 0 | #NUM! | 0 | Baseline |
| Male | 35+ | 0 | #NUM! | 0 | Baseline |
| Male | 35+ | 0 | #NUM! | 0 | Baseline |
| Male | 22 to 35 | 0 | #NUM! | 0 | Baseline |

| Male | 22 to 35 | 0 | #NUM! | 0 | Baseline |
| --- | --- | --- | --- | --- | --- |
| Male | 22 to 35 | 0 | #NUM! | 0 | Baseline |
| Male | 5 to 14 | 0 | #NUM! | 0 | Baseline |
| Male | 5 to 14 | 0 | #NUM! | 0 | Baseline |
| Male | 5 to 14 | 0 | #NUM! | 0 | Baseline |
| Male | 5 to 14 | 0 | #NUM! | 0 | Baseline |
| Male | 5 to 14 | 0 | #NUM! | 0 | Baseline |
| Male | 5 to 14 | 0 | #NUM! | 0 | Baseline |
| Male | 5 to 14 | 0 | #NUM! | 0 | Baseline |
| Male | 5 to 14 | 0 | #NUM! | 0 | Baseline |
| Male | 5 to 14 | 0 | #NUM! | 0 | Baseline |
| Male | 5 to 14 | 0 | #NUM! | 0 | Baseline |
| Male | 5 to 14 | 0 | #NUM! | 0 | Baseline |
| Male | 5 to 14 | 0 | #NUM! | 0 | Baseline |
| Female | 5 to 14 | 0 | #NUM! | 0 | Baseline |
| Male | 5 to 14 | 0 | #NUM! | 0 | Baseline |
| Male | 15 to 21 | 0 | #NUM! | 0 | Baseline |
| Male | 5 to 14 | 0 | #NUM! | 0 | Baseline |
| Male | 5 to 14 | 0 | #NUM! | 0 | Baseline |
| Male | 5 to 14 | 0 | #NUM! | 0 | Baseline |
| Male | 5 to 14 | 0 | #NUM! | 0 | Baseline |
| Male | 5 to 14 | 0 | #NUM! | 0 | Baseline |
| Male | 5 to 14 |  | #NUM! | 0 | Baseline |
| Male | 5 to 14 | 0 | #NUM! | 0 | Baseline |
| Male | 15 to 21 | 0 | #NUM! | 0 | Baseline |
| Male | 15 to 21 | 0 | #NUM! | 0 | Baseline |
| Male | 15 to 21 | 0 | #NUM! | 0 | Baseline |
| Female | 15 to 21 | 0 | #NUM! | 0 | Baseline |
| Female | 15 to 21 | 0 | #NUM! | 0 | Baseline |
| Female | 15 to 21 | 0 | #NUM! | 0 | Baseline |
| Female | 15 to 21 | 0 | #NUM! | 0 | Baseline |
| Female | 15 to 21 | 0 | #NUM! | 0 | Baseline |
| Female | 15 to 21 | 0 | #NUM! | 0 | Baseline |
| Female | 15 to 21 | 0 | #NUM! | 0 | Baseline |
| Female | 15 to 21 | 0 | #NUM! | 0 | Baseline |
| Female | 15 to 21 | 0 | #NUM! | 0 | Baseline |
| Female | 15 to 21 | 0 | #NUM! | 0 | Baseline |
| Female | 5 to 14 | 0 | #NUM! | 0 | Baseline |
| Female | 5 to 14 | 0 | #NUM! | 0 | Baseline |
| Female | 5 to 14 | 0 | #NUM! | 0 | Baseline |
| Female | 5 to 14 | 0 | #NUM! | 0 | Baseline |
| Female | 5 to 14 | 0 | #NUM! | 0 | Baseline |
| Female | 5 to 14 | 0 | #NUM! | 0 | Baseline |
| Female | 5 to 14 | 0 | #NUM! | 0 | Baseline |
| Female | 5 to 14 | 0 | #NUM! | 0 | Baseline |
| Female | 5 to 14 | 0 | #NUM! | 0 | Baseline |
| Female | 5 to 14 | 0 | #NUM! | 0 | Baseline |
| Female | 5 to 14 | 0 | #NUM! | 0 | Baseline |
| Female | 5 to 14 | 0 | #NUM! | 0 | Baseline |
| Female | 15 to 21 | 0 | #NUM! | 0 | Baseline |

| Female | 5 to 14 | 0 | #NUM! | 0 | Baseline |
| --- | --- | --- | --- | --- | --- |
| Female | 5 to 14 | 0 | #NUM! | 0 | Baseline |
| Male | 1 to 4 | 0 | #NUM! | 0 | Baseline |
| Male | 15 to 21 | 0 | #NUM! | 0 | Baseline |
| Male | 15 to 21 | 0 | #NUM! | 0 | Baseline |
| Male | 15 to 21 | 0 | #NUM! | 0 | Baseline |
| Male | 15 to 21 | 0 | #NUM! | 0 | Baseline |
| Male | 15 to 21 | 0 | #NUM! | 0 | Baseline |
| Male | 15 to 21 | 0 | #NUM! | 0 | Baseline |
| Female | 5 to 14 | 0 | #NUM! | 0 | Baseline |
| Female | 5 to 14 | 0 | #NUM! | 0 | Baseline |
| Female | 5 to 14 | 0 | #NUM! | 0 | Baseline |
| Male | 35+ | 0 | #NUM! | 0 | Baseline |
| Male | 22 to 35 | 0 | #NUM! | 0 | Baseline |
| Female | 22 to 35 | 0 | #NUM! | 0 | Baseline |
| Male | 35+ | 0 | #NUM! | 0 | Baseline |
| Male | 35+ | 0 | #NUM! | 0 | Baseline |
| Male | 22 to 35 | 0 | #NUM! | 0 | Baseline |
| Male | 35+ | 0 | #NUM! | 0 | Baseline |
| Female | 22 to 35 | 0 | #NUM! | 0 | Baseline |
| Female | 22 to 35 | 0 | #NUM! | 0 | Baseline |
| Female | 35+ | 0 | #NUM! | 0 | Baseline |
| Female | 35+ | 0 | #NUM! | 0 | Baseline |
| Male | 35+ | 0 | #NUM! | 0 | Baseline |
| Female | 22 to 35 | 0 | #NUM! | 0 | Baseline |
| Female | 22 to 35 | 0 | #NUM! | 0 | Baseline |
| Female | 22 to 35 | 0 | #NUM! | 0 | Baseline |
| Female | 22 to 35 | 0 | #NUM! | 0 | Baseline |
| Male | 1 to 4 | 0 | #NUM! | 0 | Baseline |
| Female | 22 to 35 | 0 | #NUM! | 0 | Baseline |
| Female | 22 to 35 | 0 | #NUM! | 0 | Baseline |
| Female | 35+ | 0 | #NUM! | 0 | Baseline |
| Female | 35+ | 0 | #NUM! | 0 | Baseline |
| Female | 35+ | 0 | #NUM! | 0 | Baseline |
| Female | 35+ | 0 | #NUM! | 0 | Baseline |
| Male | 22 to 35 | 0 | #NUM! | 0 | Baseline |
| Male | 22 to 35 | 0 | #NUM! | 0 | Baseline |
| Male | 22 to 35 | 0 | #NUM! | 0 | Baseline |
| Male | 22 to 35 | 0 | #NUM! | 0 | Baseline |
| Male | 35+ | 0 | #NUM! | 0 | Baseline |
| Male | 22 to 35 | 0 | #NUM! | 0 | Baseline |
| Female | 35+ | 0 | #NUM! | 0 | Baseline |
| Male | 22 to 35 | 0 | #NUM! | 0 | Baseline |
| Male | 35+ | 0 | #NUM! | 0 | Baseline |
| Female | 35+ | 0 | #NUM! | 0 | Baseline |
| Female | 35+ | 0 | #NUM! | 0 | Baseline |
| Female | 35+ | 0 | #NUM! | 0 | Baseline |
| Female | 35+ | 0 | #NUM! | 0 | Baseline |
| Male | 35+ | 0 | #NUM! | 0 | Baseline |
| Male | 22 to 35 | 0 | #NUM! | 0 | Baseline |

| Male | 22 to 35 | 0 | #NUM! | 0 | Baseline |
| --- | --- | --- | --- | --- | --- |
| Male | 35+ | 0 | #NUM! | 0 | Baseline |
| Female | 35+ | 0 | #NUM! | 0 | Baseline |
| Female | 35+ | 0 | #NUM! | 0 | Baseline |
| Male | 35+ | 0 | #NUM! | 0 | Baseline |
| Male | 35+ | 0 | #NUM! | 0 | Baseline |
| Male | 1 to 4 | 0 | #NUM! | 0 | Baseline |
| Male | 22 to 35 | 0 | #NUM! | 0 | Baseline |
| Female | 22 to 35 | 0 | #NUM! | 0 | Baseline |
| Female | 5 to 14 | 0 | #NUM! | 0 | Baseline |
| Male | 5 to 14 | 0 | #NUM! | 0 | Baseline |
| Female | 35+ | 0 | #NUM! | 0 | Baseline |
| Male | 35+ | 0 | #NUM! | 0 | Baseline |
| Female | 35+ | 0 | #NUM! | 0 | Baseline |
| Male | 5 to 14 | 0 | #NUM! | 0 | Baseline |
| Male | 35+ | 0 | #NUM! | 0 | Baseline |
| Male | 1 to 4 | 0 | #NUM! | 0 | Baseline |
| Female | 35+ | 0 | #NUM! | 0 | Baseline |
| Male | 1 to 4 | 0 | #NUM! | 0 | Baseline |
| Female | 35+ | 0 | #NUM! | 0 | Baseline |
| Male | 22 to 35 | 0 | #NUM! | 0 | Baseline |
| Female | 35+ | 0 | #NUM! | 0 | Baseline |
| Female | 35+ | 0 | #NUM! | 0 | Baseline |
| Female | 35+ | 0 | #NUM! | 0 | Baseline |
| Female | 22 to 35 | 0 | #NUM! | 0 | Baseline |
| Male | 5 to 14 | 0 | #NUM! | 0 | Baseline |
| Female | 22 to 35 | 0 | #NUM! | 0 | Baseline |
| Male | 5 to 14 | 0 | #NUM! | 0 | Baseline |
| Male | 5 to 14 | 0 | #NUM! | 0 | Baseline |
| Male | 1 to 4 | 0 | #NUM! | 0 | Baseline |
| Female | 1 to 4 | 0 | #NUM! | 0 | Baseline |
| Male | 1 to 4 | 0 | #NUM! | 0 | Baseline |
| Female | 5 to 14 | 0 | #NUM! | 0 | Baseline |
| Female | 5 to 14 | 0 | #NUM! | 0 | Baseline |
| Female | 5 to 14 | 0 | #NUM! | 0 | Baseline |
| Female | 1 to 4 | 0 | #NUM! | 0 | Baseline |
| Male | 35+ | 0 | #NUM! | 0 | Baseline |
| Male | 1 to 4 | 0 | #NUM! | 0 | Baseline |
| Male | 22 to 35 | 0 | #NUM! | 0 | Baseline |
| Male | 5 to 14 | 0 | #NUM! | 0 | Baseline |
| Female | 5 to 14 | 0 | #NUM! | 0 | Baseline |
| Male | 35+ | 0 | #NUM! | 0 | Baseline |
| Male | 22 to 35 | 0 | #NUM! | 0 | Baseline |
| Female | 5 to 14 | 0 | #NUM! | 0 | Baseline |
| Female | 5 to 14 | 0 | #NUM! | 0 | Baseline |
| Male | 5 to 14 | 0 | #NUM! | 0 | Baseline |
| Male | 35+ | 0 | #NUM! | 0 | Baseline |
| Female | 5 to 14 | 0 | #NUM! | 0 | Baseline |
| Female | 5 to 14 | 0 | #NUM! | 0 | Baseline |
| Male | 5 to 14 | 0 | #NUM! | 0 | Baseline |

| Male | 5 to 14 | 0 | #NUM! | 0 | Baseline |
| --- | --- | --- | --- | --- | --- |
| Male | 5 to 14 | 0 | #NUM! | 0 | Baseline |
| Male | 5 to 14 | 0 | #NUM! | 0 | Baseline |
| Male | 5 to 14 | 0 | #NUM! | 0 | Baseline |
| Male | 5 to 14 | 0 | #NUM! | 0 | Baseline |
| Female | 5 to 14 | 0 | #NUM! | 0 | Baseline |
| Female | 5 to 14 | 0 | #NUM! | 0 | Baseline |
| Female | 5 to 14 | 0 | #NUM! | 0 | Baseline |
| Male | 5 to 14 | 0 | #NUM! | 0 | Baseline |
| Male | 5 to 14 | 0 | #NUM! | 0 | Baseline |
| Male | 5 to 14 | 0 | #NUM! | 0 | Baseline |
| Male | 5 to 14 | 0 | #NUM! | 0 | Baseline |
| Female | 15 to 21 | 0 | #NUM! | 0 | Baseline |
| Female | 15 to 21 | 0 | #NUM! | 0 | Baseline |
| Female | 15 to 21 | 0 | #NUM! | 0 | Baseline |
| Female | 15 to 21 | 0 | #NUM! | 0 | Baseline |
| Female | 15 to 21 | 0 | #NUM! | 0 | Baseline |
| Female | 15 to 21 | 0 | #NUM! | 0 | Baseline |
| Female | 5 to 14 | 0 | #NUM! | 0 | Baseline |
| Male | 1 to 4 | 0 | #NUM! | 0 | Baseline |
| Female | 5 to 14 | 0 | #NUM! | 0 | Baseline |
| Male | 1 to 4 | 0 | #NUM! | 0 | Baseline |
| Female | 5 to 14 | 0 | #NUM! | 0 | Baseline |
| Male | 15 to 21 | 0 | #NUM! | 0 | Baseline |
| Male | 15 to 21 | 0 | #NUM! | 0 | Baseline |
| Male | 5 to 14 | 0 | #NUM! | 0 | Baseline |
| Male | 22 to 35 | 0 | #NUM! | 0 | Baseline |
| Male | 5 to 14 | 0 | #NUM! | 0 | Baseline |
| Male | 5 to 14 | 0 | #NUM! | 0 | Baseline |
| Male | 5 to 14 | 0 | #NUM! | 0 | Baseline |
| Male | 1 to 4 | 0 | #NUM! | 0 | Baseline |
| Male | 1 to 4 | 0 | #NUM! | 0 | Baseline |
| Male | 22 to 35 | 0 | #NUM! | 0 | Baseline |
| Female | 1 to 4 | 0 | #NUM! | 0 | Baseline |
| Female | 15 to 21 | 0 | #NUM! | 0 | Baseline |
| Male | 1 to 4 | 0 | #NUM! | 0 | Baseline |
| Female | 5 to 14 | 0 | #NUM! | 0 | Baseline |
| Female | 5 to 14 | 0 | #NUM! | 0 | Baseline |
| Female | 1 to 4 | 0 | #NUM! | 0 | Baseline |
| Female | 1 to 4 | 0 | #NUM! | 0 | Baseline |
| Female | 5 to 14 | 0 | #NUM! | 0 | Baseline |
| Male | 5 to 14 | 0 | #NUM! | 0 | Baseline |
| Male | 5 to 14 | 0 | #NUM! | 0 | Baseline |
| Male | 5 to 14 | 0 | #NUM! | 0 | Baseline |
| Male | 5 to 14 | 0 | #NUM! | 0 | Baseline |
| Male | 1 to 4 | 0 | #NUM! | 0 | Baseline |
| Male | 5 to 14 | 0 | #NUM! | 0 | Baseline |
| Male | 1 to 4 | 0 | #NUM! | 0 | Baseline |
| Female | 5 to 14 | 0 | #NUM! | 0 | Baseline |
| Female | 35+ | 0 | #NUM! | 0 | Baseline |

| Male | 35+ | 0 | #NUM! | 0 | Baseline |
| --- | --- | --- | --- | --- | --- |
| Male | 35+ | 0 | #NUM! | 0 | Baseline |
| Male | 22 to 35 | 0 | #NUM! | 0 | Baseline |
| Male | 35+ | 0 | #NUM! | 0 | Baseline |
| Male | 22 to 35 | 0 | #NUM! | 0 | Baseline |
| Female | 35+ | 0 | #NUM! | 0 | Baseline |
| Female | 35+ | 0 | #NUM! | 0 | Baseline |
| Female | 22 to 35 | 0 | #NUM! | 0 | Baseline |
| Male | 35+ | 0 | #NUM! | 0 | Baseline |
| Female | 22 to 35 | 0 | #NUM! | 0 | Baseline |
| Female | 35+ | 0 | #NUM! | 0 | Baseline |
| Female | 35+ | 0 | #NUM! | 0 | Baseline |
| Male | 22 to 35 | 0 | #NUM! | 0 | Baseline |
| Male | 22 to 35 | 0 | #NUM! | 0 | Baseline |
| Male | 35+ | 0 | #NUM! | 0 | Baseline |
| Female | 22 to 35 | 0 | #NUM! | 0 | Baseline |
| Male | 35+ | 0 | #NUM! | 0 | Baseline |
| Male | 22 to 35 | 0 | #NUM! | 0 | Baseline |
| Female | 35+ | 0 | #NUM! | 0 | Baseline |
| Female | 5 to 14 | 0 | #NUM! | 0 | Baseline |
| Male | 5 to 14 | 0 | #NUM! | 0 | Baseline |
| Male | 5 to 14 | 0 | #NUM! | 0 | Baseline |
| Female | 15 to 21 | 0 | #NUM! | 0 | Baseline |
| Male | 15 to 21 | 0 | #NUM! | 0 | Baseline |
| Male | 15 to 21 | 0 | #NUM! | 0 | Baseline |
| Male | 15 to 21 | 0 | #NUM! | 0 | Baseline |
| Female | 15 to 21 | 0 | #NUM! | 0 | Baseline |
| Female | 15 to 21 | 0 | #NUM! | 0 | Baseline |
| Male | 5 to 14 | 0 | #NUM! | 0 | Baseline |
| Male | 5 to 14 | 0 | #NUM! | 0 | Baseline |
| Female | 15 to 21 | 0 | #NUM! | 0 | Baseline |
| Male | 1 to 4 | 0 | #NUM! | 0 | Baseline |
| Female | 15 to 21 | 0 | #NUM! | 0 | Baseline |
| Female | 15 to 21 | 0 | #NUM! | 0 | Baseline |
| Male | 15 to 21 | 0 | #NUM! | 0 | Baseline |
| Male | 15 to 21 | 0 | #NUM! | 0 | Baseline |
| Male | 5 to 14 | 0 | #NUM! | 0 | Baseline |
| Male | 5 to 14 | 0 | #NUM! | 0 | Baseline |
| Female | 15 to 21 | 0 | #NUM! | 0 | Baseline |
| Female | 5 to 14 | 0 | #NUM! | 0 | Baseline |
| Female | 5 to 14 | 0 | #NUM! | 0 | Baseline |
| Male | 15 to 21 | 0 | #NUM! | 0 | Baseline |
| Male | 1 to 4 | 0 | #NUM! | 0 | Baseline |
| Female | 5 to 14 | 0 | #NUM! | 0 | Baseline |
| Female | 5 to 14 | 0 | #NUM! | 0 | Baseline |
| Female | 5 to 14 | 0 | #NUM! | 0 | Baseline |
| Male | 5 to 14 | 0 | #NUM! | 0 | Baseline |
| Male | 5 to 14 | 0 | #NUM! | 0 | Baseline |
| Male | 5 to 14 | 0 | #NUM! | 0 | Baseline |
| Female | 5 to 14 | 0 | #NUM! | 0 | Baseline |

| Male | 5 to 14 | 0 | #NUM! | 0 | Baseline |
| --- | --- | --- | --- | --- | --- |
| Male | 5 to 14 | 0 | #NUM! | 0 | Baseline |
| Male | 1 to 4 | 0 | #NUM! | 0 | Baseline |
| Female | 5 to 14 | 0 | #NUM! | 0 | Baseline |
| Female | 22 to 35 | 0 | #NUM! | 0 | Baseline |
| Female | 22 to 35 | 0 | #NUM! | 0 | Baseline |
| Female | 15 to 21 | 0 | #NUM! | 0 | Baseline |
| Male | 15 to 21 | 0 | #NUM! | 0 | Baseline |
| Male | 5 to 14 | 0 | #NUM! | 0 | Baseline |
| Female | 5 to 14 | 0 | #NUM! | 0 | Baseline |
| Female | 5 to 14 | 0 | #NUM! | 0 | Baseline |
| Female | 15 to 21 | 0 | #NUM! | 0 | Baseline |
| Female | 15 to 21 | 0 | #NUM! | 0 | Baseline |
| Female | 5 to 14 | 0 | #NUM! | 0 | Baseline |
| Male | 35+ | 0 | #NUM! | 0 | Baseline |
| Male | 15 to 21 | 0 | #NUM! | 0 | Baseline |
| Female | 5 to 14 | 0 | #NUM! | 0 | Baseline |
| Female | 15 to 21 | 0 | #NUM! | 0 | Baseline |
| Male | 15 to 21 | 0 | #NUM! | 0 | Baseline |
| Male | 15 to 21 | 0 | #NUM! | 0 | Baseline |
| Male | 15 to 21 | 0 | #NUM! | 0 | Baseline |
| Female | 15 to 21 | 0 | #NUM! | 0 | Baseline |
| Male | 5 to 14 | 0 | #NUM! | 0 | Baseline |
| Female | 5 to 14 | 0 | #NUM! | 0 | Baseline |
| Male | 5 to 14 | 0 | #NUM! | 0 | Baseline |
| Female | 15 to 21 | 0 | #NUM! | 0 | Baseline |
| Female | 15 to 21 | 0 | #NUM! | 0 | Baseline |
| Male | 15 to 21 | 0 | #NUM! | 0 | Baseline |
| Female | 5 to 14 | 0 | #NUM! | 0 | Baseline |
| Male | 1 to 4 | 0 | #NUM! | 0 | Baseline |
| Female | 35+ | 0 | #NUM! | 0 | Baseline |
| Male | 35+ | 0 | #NUM! | 0 | Baseline |
| Female | 35+ | 0 | #NUM! | 0 | Baseline |
| Male | 35+ | 0 | #NUM! | 0 | Baseline |
| Male | 35+ | 0 | #NUM! | 0 | Baseline |
| Female | 35+ | 0 | #NUM! | 0 | Baseline |
| Female | 35+ | 0 | #NUM! | 0 | Baseline |
| Male | 35+ | 0 | #NUM! | 0 | Baseline |
| Male | 35+ | 0 | #NUM! | 0 | Baseline |
| Female | 35+ | 0 | #NUM! | 0 | Baseline |
| Male | 35+ | 0 | #NUM! | 0 | Baseline |
| Female | 35+ | 0 | #NUM! | 0 | Baseline |
| Female | 35+ | 0 | #NUM! | 0 | Baseline |
| Male | 22 to 35 | 0 | #NUM! | 0 | Baseline |
| Female | 35+ | 0 | #NUM! | 0 | Baseline |
| Male | 22 to 35 | 0 | #NUM! | 0 | Baseline |
| Female | 35+ | 0 | #NUM! | 0 | Baseline |
| Male | 22 to 35 | 0 | #NUM! | 0 | Baseline |
| Male | 22 to 35 | 0 | #NUM! | 0 | Baseline |
| Female | 22 to 35 | 0 | #NUM! | 0 | Baseline |

| Female | 35+ | 0 | #NUM! | 0 | Baseline |
| --- | --- | --- | --- | --- | --- |
| Female | 22 to 35 | 0 | #NUM! | 0 | Baseline |
| Female | 22 to 35 | 0 | #NUM! | 0 | Baseline |
| Female | 22 to 35 | 0 | #NUM! | 0 | Baseline |
| Male | 22 to 35 | 0 | #NUM! | 0 | Baseline |
| Male | 22 to 35 | 0 | #NUM! | 0 | Baseline |
| Female | 22 to 35 | 0 | #NUM! | 0 | Baseline |
| Male | 1 to 4 | 0 | #NUM! | 0 | Baseline |
| Male | 22 to 35 | 0 | #NUM! | 0 | Baseline |
| Female | 22 to 35 | 0 | #NUM! | 0 | Baseline |
| Female | 22 to 35 | 0 | #NUM! | 0 | Baseline |
| Female | 22 to 35 | 0 | #NUM! | 0 | Baseline |
| Female | 22 to 35 | 0 | #NUM! | 0 | Baseline |
| Female | 22 to 35 | 0 | #NUM! | 0 | Baseline |
| Male | 15 to 21 | 0 | #NUM! | 0 | Baseline |
| Female | 5 to 14 | 0 | #NUM! | 0 | Baseline |
| Male | 15 to 21 | 0 | #NUM! | 0 | Baseline |
| Female | 15 to 21 | 0 | #NUM! | 0 | Baseline |
| Male | 15 to 21 | 0 | #NUM! | 0 | Baseline |
| Female | 5 to 14 | 0 | #NUM! | 0 | Baseline |
| Female | 15 to 21 | 0 | #NUM! | 0 | Baseline |
| Female | 15 to 21 | 0 | #NUM! | 0 | Baseline |
| Female | 15 to 21 | 0 | #NUM! | 0 | Baseline |
| Male | 5 to 14 | 0 | #NUM! | 0 | Baseline |
| Male | 5 to 14 | 0 | #NUM! | 0 | Baseline |
| Male | 5 to 14 | 0 | #NUM! | 0 | Baseline |
| Male | 1 to 4 | 0 | #NUM! | 0 | Baseline |
| Male | 1 to 4 | 0 | #NUM! | 0 | Baseline |
| Male | 1 to 4 | 0 | #NUM! | 0 | Baseline |
| Male | 5 to 14 | 0 | #NUM! | 0 | Baseline |
| Female | 5 to 14 | 0 | #NUM! | 0 | Baseline |
| Female | 5 to 14 | 0 | #NUM! | 0 | Baseline |
| Female | 5 to 14 | 0 | #NUM! | 0 | Baseline |
| Female | 35+ | 0 | #NUM! | 0 | Baseline |
| Male | 35+ | 0 | #NUM! | 0 | Baseline |
| Female | 22 to 35 | 0 | #NUM! | 0 | Baseline |
| Female | 1 to 4 | 0 | #NUM! | 0 | Baseline |
| Female | 5 to 14 | 0 | #NUM! | 0 | Baseline |
| Male | 22 to 35 | 0 | #NUM! | 0 | Baseline |
| Male | 22 to 35 | 0 | #NUM! | 0 | Baseline |
| Male | 22 to 35 | 0 | #NUM! | 0 | Baseline |
| Male | 22 to 35 | 0 | #NUM! | 0 | Baseline |
| Male | 22 to 35 | 0 | #NUM! | 0 | Baseline |
| Male | 22 to 35 | 0 | #NUM! | 0 | Baseline |
| Male | 22 to 35 | 0 | #NUM! | 0 | Baseline |
| Male | 22 to 35 | 0 | #NUM! | 0 | Baseline |
| Male | 22 to 35 | 0 | #NUM! | 0 | Baseline |
| Male | 22 to 35 | 0 | #NUM! | 0 | Baseline |
| Male | 1 to 4 | 0 | #NUM! | 0 | Baseline |
| Male | 35+ | 0 | #NUM! | 0 | Baseline |

| Male | 35+ | 0 | #NUM! | 0 | Baseline |
| --- | --- | --- | --- | --- | --- |
| Male | 35+ | 0 | #NUM! | 0 | Baseline |
| Male | 35+ | 0 | #NUM! | 0 | Baseline |
| Male | 35+ | 0 | #NUM! | 0 | Baseline |
| Male | 35+ | 0 | #NUM! | 0 | Baseline |
| Male | 35+ | 0 | #NUM! | 0 | Baseline |
| Male | 35+ | 0 | #NUM! | 0 | Baseline |
| Male | 35+ | 0 | #NUM! | 0 | Baseline |
| Male | 35+ | 0 | #NUM! | 0 | Baseline |
| Female | 22 to 35 | 0 | #NUM! | 0 | Baseline |
| Female | 22 to 35 | 0 | #NUM! | 0 | Baseline |
| Female | 22 to 35 | 0 | #NUM! | 0 | Baseline |
| Male | 22 to 35 | 0 | #NUM! | 0 | Baseline |
| Female | 22 to 35 | 0 | #NUM! | 0 | Baseline |
| Female | 22 to 35 | 0 | #NUM! | 0 | Baseline |
| Female | 22 to 35 | 0 | #NUM! | 0 | Baseline |
| Female | 22 to 35 | 0 | #NUM! | 0 | Baseline |
| Female | 22 to 35 | 0 | #NUM! | 0 | Baseline |
| Female | 1 to 4 | 0 | #NUM! | 0 | Baseline |
| Female | 35+ | 0 | #NUM! | 0 | Baseline |
| Female | 35+ | 0 | #NUM! | 0 | Baseline |
| Female | 35+ | 0 | #NUM! | 0 | Baseline |
| Female | 35+ | 0 | #NUM! | 0 | Baseline |
| Male | 5 to 14 | 0 | #NUM! | 0 | Baseline |
| Female | 35+ | 0 | #NUM! | 0 | Baseline |
| Female | 35+ | 0 | #NUM! | 0 | Baseline |
| Female | 35+ | 0 | #NUM! | 0 | Baseline |
| Female | 35+ | 0 | #NUM! | 0 | Baseline |
| Male | 1 to 4 | 0 | #NUM! | 0 | Baseline |
| Male | 22 to 35 | 0 | #NUM! | 0 | Baseline |
| Female | 35+ | 0 | #NUM! | 0 | Baseline |
| Male | 1 to 4 | 0 | #NUM! | 0 | Baseline |
| Male | 1 to 4 | 0 | #NUM! | 0 | Baseline |
| Male | 1 to 4 | 0 | #NUM! | 0 | Baseline |
| Male | 1 to 4 | 0 | #NUM! | 0 | Baseline |
| Male | 1 to 4 | 0 | #NUM! | 0 | Baseline |
| Male | 5 to 14 | 0 | #NUM! | 0 | Baseline |
| Male | 5 to 14 | 0 | #NUM! | 0 | Baseline |
| Male | 5 to 14 | 0 | #NUM! | 0 | Baseline |
| Male | 5 to 14 | 0 | #NUM! | 0 | Baseline |
| Female | 1 to 4 | 0 | #NUM! | 0 | Baseline |
| Female | 5 to 14 | 0 | #NUM! | 0 | Baseline |
| Female | 1 to 4 | 0 | #NUM! | 0 | Baseline |
| Female | 1 to 4 | 0 | #NUM! | 0 | Baseline |
| Female | 5 to 14 | 0 | #NUM! | 0 | Baseline |
| Female | 5 to 14 | 0 | #NUM! | 0 | Baseline |
| Female | 5 to 14 | 0 | #NUM! | 0 | Baseline |
| Female | 5 to 14 | 0 | #NUM! | 0 | Baseline |
| Female | 5 to 14 | 0 | #NUM! | 0 | Baseline |
| Female | 1 to 4 | 0 | #NUM! | 0 | Baseline |

| Male | 1 to 4 | 0 | #NUM! | 0 | Baseline |
| --- | --- | --- | --- | --- | --- |
| Male | 22 to 35 | 0 | #NUM! | 0 | Baseline |
| Female | 35+ | 0 | #NUM! | 0 | Baseline |
| Male | 35+ | 0 | #NUM! | 0 | Baseline |
| Female | 5 to 14 | 0 | #NUM! | 0 | Baseline |
| Male | 35+ | 0 | #NUM! | 0 | Baseline |
| Male | 22 to 35 | 0 | #NUM! | 0 | Baseline |
| Male | 35+ | 0 | #NUM! | 0 | Baseline |
| Male | 1 to 4 | 0 | #NUM! | 0 | Baseline |
| Female | 1 to 4 | 0 | #NUM! | 0 | Baseline |
| Female | 5 to 14 | 0 | #NUM! | 0 | Baseline |
| Female | 22 to 35 | 0 | #NUM! | 0 | Baseline |
| Male | 35+ | 0 | #NUM! | 0 | Baseline |
| Male | 15 to 21 | 0 | #NUM! | 0 | Baseline |
| Male | 1 to 4 | 0 | #NUM! | 0 | Baseline |
| Male | 15 to 21 | 0 | #NUM! | 0 | Baseline |
| Female | 22 to 35 | 0 | #NUM! | 0 | Baseline |
| Female | 22 to 35 | 0 | #NUM! | 0 | Baseline |
| Male | 15 to 21 | 0 | #NUM! | 0 | Baseline |
| Male | 15 to 21 | 0 | #NUM! | 0 | Baseline |
| Male | 22 to 35 | 0 | #NUM! | 0 | Baseline |
| Female | 15 to 21 | 0 | #NUM! | 0 | Baseline |
| Female | 22 to 35 | 0 | #NUM! | 0 | Baseline |
| Male | 1 to 4 | 0 | #NUM! | 0 | Baseline |
| Female | 5 to 14 | 0 | #NUM! | 0 | Baseline |
| Male | 5 to 14 | 0 | #NUM! | 0 | Baseline |
| Female | 22 to 35 | 0 | #NUM! | 0 | Baseline |
| Female | 1 to 4 | 0 | #NUM! | 0 | Baseline |
| Male | 1 to 4 | 0 | #NUM! | 0 | Baseline |
| Male | 1 to 4 | 0 | #NUM! | 0 | Baseline |
| Male | 35+ | 0 | #NUM! | 0 | Baseline |
| Male | 1 to 4 | 0 | #NUM! | 0 | Baseline |
| Female | 22 to 35 | 0 | #NUM! | 0 | Baseline |
| Male | 22 to 35 | 0 | #NUM! | 0 | Baseline |
| Male | 1 to 4 | 0 | #NUM! | 0 | Baseline |
| Male | 22 to 35 | 0 | #NUM! | 0 | Baseline |
| Female | 22 to 35 | 0 | #NUM! | 0 | Baseline |
| Female | 35+ | 0 | #NUM! | 0 | Baseline |
| Female | 22 to 35 | 0 | #NUM! | 0 | Baseline |
| Male | 1 to 4 | 0 | #NUM! | 0 | Baseline |
| Female | 35+ | 0 | #NUM! | 0 | Baseline |
| Female | 22 to 35 | 0 | #NUM! | 0 | Baseline |
| Male | 1 to 4 | 0 | #NUM! | 0 | Baseline |
| Male | 1 to 4 | 0 | #NUM! | 0 | Baseline |
| Male | 1 to 4 | 0 | #NUM! | 0 | Baseline |
| Male | 1 to 4 | 0 | #NUM! | 0 | Baseline |
| Male | 1 to 4 | 0 | #NUM! | 0 | Baseline |
| Female | 22 to 35 | 0 | #NUM! | 0 | Baseline |
| Male | 15 to 21 | 0 | #NUM! | 0 | Baseline |
| Male | 15 to 21 | 0 | #NUM! | 0 | Baseline |

| Male | 1 to 4 | 0 | #NUM! | 0 | Baseline |
| --- | --- | --- | --- | --- | --- |
| Male | 15 to 21 | 0 | #NUM! | 0 | Baseline |
| Male | 15 to 21 | 0 | #NUM! | 0 | Baseline |
| Male | 1 to 4 | 0 | #NUM! | 0 | Baseline |
| Male | 1 to 4 | 0 | #NUM! | 0 | Baseline |
| Female | 5 to 14 | 0 | #NUM! | 0 | Baseline |
| Male | 22 to 35 | 0 | #NUM! | 0 | Baseline |
| Male | 1 to 4 | 0 | #NUM! | 0 | Baseline |
| Female | 15 to 21 | 0 | #NUM! | 0 | Baseline |
| Male | 35+ | 0 | #NUM! | 0 | Baseline |
| Female | 22 to 35 | 0 | #NUM! | 0 | Baseline |
| Male | 1 to 4 | 0 | #NUM! | 0 | Baseline |
| Female | 1 to 4 | 0 | #NUM! | 0 | Baseline |
| Female | 22 to 35 | 0 | #NUM! | 0 | Baseline |
| Female | 5 to 14 | 0 | #NUM! | 0 | Baseline |
| Male | 1 to 4 | 0 | #NUM! | 0 | Baseline |
| Male | 1 to 4 | 0 | #NUM! | 0 | Baseline |
| Female | 35+ | 0 | #NUM! | 0 | Baseline |
| Female | 5 to 14 | 0 | #NUM! | 0 | Baseline |
| Male | 35+ | 0 | #NUM! | 0 | Baseline |
| Male | 22 to 35 | 0 | #NUM! | 0 | Baseline |
| Male | 22 to 35 | 0 | #NUM! | 0 | Baseline |
| Female | 15 to 21 | 0 | #NUM! | 0 | Baseline |
| Female | 35+ | 0 | #NUM! | 0 | Baseline |
| Female | 15 to 21 | 0 | #NUM! | 0 | Baseline |
| Male | 15 to 21 | 0 | #NUM! | 0 | Baseline |
| Male | 35+ | 0 | #NUM! | 0 | Baseline |
| Male | 35+ | 0 | #NUM! | 0 | Baseline |
| Female | 35+ | 0 | #NUM! | 0 | Baseline |
| Female | 22 to 35 | 0 | #NUM! | 0 | Baseline |
| Male | 22 to 35 | 0 | #NUM! | 0 | Baseline |
| Male | 22 to 35 | 0 | #NUM! | 0 | Baseline |
| Female | 35+ | 0 | #NUM! | 0 | Baseline |
| Male | 35+ | 0 | #NUM! | 0 | Baseline |
| Male | 22 to 35 | 0 | #NUM! | 0 | Baseline |
| Female | 22 to 35 | 0 | #NUM! | 0 | Baseline |
| Male | 22 to 35 | 0 | #NUM! | 0 | Baseline |
| Female | 35+ | 0 | #NUM! | 0 | Baseline |
| Male | 5 to 14 | 0 | #NUM! | 0 | Baseline |
| Female | 35+ | 0 | #NUM! | 0 | Baseline |
| Female | 5 to 14 | 0 | #NUM! | 0 | Baseline |
| Male | 1 to 4 | 0 | #NUM! | 0 | Baseline |
| Female | 5 to 14 | 0 | #NUM! | 0 | Baseline |
| Female | 22 to 35 | 0 | #NUM! | 0 | Baseline |
| Male | 22 to 35 | 0 | #NUM! | 0 | Baseline |
| Male | 1 to 4 | 0 | #NUM! | 0 | Baseline |
| Female | 22 to 35 | 0 | #NUM! | 0 | Baseline |
| Female | 5 to 14 | 0 | #NUM! | 0 | Baseline |
| Male | 22 to 35 | 0 | #NUM! | 0 | Baseline |
| Female | 5 to 14 | 0 | #NUM! | 0 | Baseline |

| Female | 22 to 35 | 0 | #NUM! | 0 | Baseline |
| --- | --- | --- | --- | --- | --- |
| Male | 1 to 4 | 0 | #NUM! | 0 | Baseline |
| Male | 5 to 14 | 0 | #NUM! | 0 | Baseline |
| Female | 35+ | 0 | #NUM! | 0 | Baseline |
| Female | 15 to 21 | 0 | #NUM! | 0 | Baseline |
| Female | 15 to 21 | 0 | #NUM! | 0 | Baseline |
| Female | 15 to 21 | 0 | #NUM! | 0 | Baseline |
| Male | 15 to 21 | 0 | #NUM! | 0 | Baseline |
| Female | 1 to 4 | 0 | #NUM! | 0 | Baseline |
| Female | 5 to 14 | 0 | #NUM! | 0 | Baseline |
| Female | 22 to 35 | 0 | #NUM! | 0 | Baseline |
| Female | 1 to 4 | 0 | #NUM! | 0 | Baseline |
| Male | 5 to 14 | 0 | #NUM! | 0 | Baseline |
| Female | 5 to 14 | 0 | #NUM! | 0 | Baseline |
| Male | 1 to 4 | 0 | #NUM! | 0 | Baseline |
| Male | 1 to 4 | 0 | #NUM! | 0 | Baseline |
| Male | 5 to 14 | 0 | #NUM! | 0 | Baseline |
| Female | 1 to 4 | 0 | #NUM! | 0 | Baseline |
| Female | 5 to 14 | 0 | #NUM! | 0 | Baseline |
| Male | 5 to 14 | 0 | #NUM! | 0 | Baseline |
| Female | 5 to 14 | 0 | #NUM! | 0 | Baseline |
| Male | 35+ | 0 | #NUM! | 0 | Baseline |
| Female | 5 to 14 | 0 | #NUM! | 0 | Baseline |
| Male | 15 to 21 | 0 | #NUM! | 0 | Baseline |
| Male | 35+ | 0 | #NUM! | 0 | Baseline |
| Female | 5 to 14 | 0 | #NUM! | 0 | Baseline |
| Male | 15 to 21 | 0 | #NUM! | 0 | Baseline |
| Male | 22 to 35 | 0 | #NUM! | 0 | Baseline |
| Male | 15 to 21 | 0 | #NUM! | 0 | Baseline |
| Male | 15 to 21 | 0 | #NUM! | 0 | Baseline |
| Male | 15 to 21 | 0 | #NUM! | 0 | Baseline |
| Male | 1 to 4 | 0 | #NUM! | 0 | Baseline |
| Male | 35+ | 0 | #NUM! | 0 | Baseline |
| Female | 35+ | 0 | #NUM! | 0 | Baseline |
| Male | 35+ | 0 | #NUM! | 0 | Baseline |
| Female | 35+ | 0 | #NUM! | 0 | Baseline |
| Female | 35+ | 0 | #NUM! | 0 | Baseline |
| Female | 35+ | 0 | #NUM! | 0 | Baseline |
| Male | 22 to 35 | 0 | #NUM! | 0 | Baseline |
| Male | 22 to 35 | 0 | #NUM! | 0 | Baseline |
| Male | 22 to 35 | 0 | #NUM! | 0 | Baseline |
| Female | 22 to 35 | 0 | #NUM! | 0 | Baseline |
| Male | 22 to 35 | 0 | #NUM! | 0 | Baseline |
| Female | 22 to 35 | 0 | #NUM! | 0 | Baseline |
| Female | 22 to 35 | 0 | #NUM! | 0 | Baseline |
| Female | 22 to 35 | 0 | #NUM! | 0 | Baseline |
| Male | 22 to 35 | 0 | #NUM! | 0 | Baseline |
| Male | 22 to 35 | 0 | #NUM! | 0 | Baseline |
| Female | 22 to 35 | 0 | #NUM! | 0 | Baseline |
| Male | 22 to 35 | 0 | #NUM! | 0 | Baseline |

| Female | 1 to 4 | 0 | #NUM! | 0 | Baseline |
| --- | --- | --- | --- | --- | --- |
| Male | 1 to 4 | 0 | #NUM! | 0 | Baseline |
| Male | 1 to 4 | 0 | #NUM! | 0 | Baseline |
| Male | 1 to 4 | 0 | #NUM! | 0 | Baseline |
| Female | 22 to 35 | 0 | #NUM! | 0 | Baseline |
| Male | 22 to 35 | 0 | #NUM! | 0 | Baseline |
| Female | 15 to 21 | 0 | #NUM! | 0 | Baseline |
| Female | 22 to 35 | 0 | #NUM! | 0 | Baseline |
| Male | 35+ | 0 | #NUM! | 0 | Baseline |
| Female | 15 to 21 | 0 | #NUM! | 0 | Baseline |
| Male | 5 to 14 | 0 | #NUM! | 0 | Baseline |
| Male | 5 to 14 | 0 | #NUM! | 0 | Baseline |
| Male | 5 to 14 | 0 | #NUM! | 0 | Baseline |
| Female | 22 to 35 | 0 | #NUM! | 0 | Baseline |
| Female | 35+ | 0 | #NUM! | 0 | Baseline |
| Female | 35+ | 0 | #NUM! | 0 | Baseline |
| Male | 5 to 14 | 0 | #NUM! | 0 | Baseline |
| Female | 35+ | 0 | #NUM! | 0 | Baseline |
| Male | 35+ | 0 | #NUM! | 0 | Baseline |
| Male | 15 to 21 | 0 | #NUM! | 0 | Baseline |
| Male | 15 to 21 | 0 | #NUM! | 0 | Baseline |
| Female | 35+ | 0 | #NUM! | 0 | Baseline |
| Female | 22 to 35 | 0 | #NUM! | 0 | Baseline |
| Female | 15 to 21 | 0 | #NUM! | 0 | Baseline |
| Male | 5 to 14 | 0 | #NUM! | 0 | Baseline |
| Male | 15 to 21 | 0 | #NUM! | 0 | Baseline |
| Female | 15 to 21 | 0 | #NUM! | 0 | Baseline |
| Male | 5 to 14 | 1 | 0 | 0 | Baseline |
| Male | 1 to 4 | 0 | #NUM! | 0 | Baseline |
| Female | 15 to 21 | 0 | #NUM! | 0 | Baseline |
| Female | 15 to 21 | 0 | #NUM! | 0 | Baseline |
| Male | 15 to 21 | 0 | #NUM! | 0 | Baseline |
| Male | 15 to 21 | 0 | #NUM! | 0 | Baseline |
| Female | 22 to 35 | 1 | 0 | 0 | Baseline |
| Male | 1 to 4 | 1 | 0 | 0 | Baseline |
| Female | 15 to 21 | 0 | #NUM! | 0 | Baseline |
| Male | 15 to 21 | 0 | #NUM! | 0 | Baseline |
| Male | 22 to 35 | 0 | #NUM! | 0 | Baseline |
| Male | 5 to 14 | 0 | #NUM! | 0 | Baseline |
| Female | 22 to 35 | 0 | #NUM! | 0 | Baseline |
| Female | 5 to 14 | 0 | #NUM! | 0 | Baseline |
| Male | 15 to 21 | 0 | #NUM! | 0 | Baseline |
| Female | 15 to 21 | 0 | #NUM! | 0 | Baseline |
| Female | 22 to 35 | 0 | #NUM! | 0 | Baseline |
| Female | 15 to 21 | 0 | #NUM! | 0 | Baseline |
| Female | 15 to 21 | 0 | #NUM! | 0 | Baseline |
| Male | 15 to 21 | 0 | #NUM! | 0 | Baseline |
| Female | 15 to 21 | 0 | #NUM! | 0 | Baseline |
| Female | 15 to 21 | 0 | #NUM! | 0 | Baseline |
| Male | 5 to 14 | 0 | #NUM! | 0 | Baseline |

| Male | 22 to 35 | 0 | #NUM! | 0 | Baseline |
| --- | --- | --- | --- | --- | --- |
| Male | 22 to 35 | 0 | #NUM! | 0 | Baseline |
| Male | 35+ | 0 | #NUM! | 0 | Baseline |
| Female | 35+ | 0 | #NUM! | 0 | Baseline |
| Male | 15 to 21 | 0 | #NUM! | 0 | Baseline |
| Male | 22 to 35 | 0 | #NUM! | 0 | Baseline |
| Male | 15 to 21 | 0 | #NUM! | 0 | Baseline |
| Female | 15 to 21 | 0 | #NUM! | 0 | Baseline |
| Female | 5 to 14 | 0 | #NUM! | 0 | Baseline |
| Female | 22 to 35 | 0 | #NUM! | 0 | Baseline |
| Female | 15 to 21 | 0 | #NUM! | 0 | Baseline |
| Male | 15 to 21 | 0 | #NUM! | 0 | Baseline |
| Female | 5 to 14 | 0 | #NUM! | 0 | Baseline |
| Male | 15 to 21 | 0 | #NUM! | 0 | Baseline |
| Female | 15 to 21 | 0 | #NUM! | 0 | Baseline |
| Male | 15 to 21 | 0 | #NUM! | 0 | Baseline |
| Male | 5 to 14 | 0 | #NUM! | 0 | Baseline |
| Male | 5 to 14 | 0 | #NUM! | 0 | Baseline |
| Male | 5 to 14 | 0 | #NUM! | 0 | Baseline |
| Male | 5 to 14 | 0 | #NUM! | 0 | Baseline |
| Female | 15 to 21 | 0 | #NUM! | 0 | Baseline |
| Male | 5 to 14 | 0 | #NUM! | 0 | Baseline |
| Male | 5 to 14 | 0 | #NUM! | 0 | Baseline |
| Female | 22 to 35 | 0 | #NUM! | 0 | Baseline |
| Male | 15 to 21 | 0 | #NUM! | 0 | Baseline |
| Male | 15 to 21 | 1 | 0 | 0 | Baseline |
| Male | 15 to 21 | 0 | #NUM! | 0 | Baseline |
| Male | 15 to 21 | 0 | #NUM! | 0 | Baseline |
| Male | 22 to 35 | 0 | #NUM! | 0 | Baseline |
| Female | 5 to 14 | 0 | #NUM! | 0 | Baseline |
| Female | 15 to 21 | 0 | #NUM! | 0 | Baseline |
| Female | 15 to 21 | 0 | #NUM! | 0 | Baseline |
| Male | 5 to 14 | 0 | #NUM! | 0 | Baseline |
| Female | 22 to 35 | 0 | #NUM! | 0 | Baseline |
| Female | 5 to 14 | 0 | #NUM! | 0 | Baseline |
| Female | 15 to 21 | 0 | #NUM! | 0 | Baseline |
| Female | 15 to 21 | 0 | #NUM! | 0 | Baseline |
| Male | 15 to 21 | 0 | #NUM! | 0 | Baseline |
| Male | 15 to 21 | 0 | #NUM! | 0 | Baseline |
| Male | 15 to 21 | 0 | #NUM! | 0 | Baseline |
| Male | 5 to 14 | 0 | #NUM! | 0 | Baseline |
| Male | 5 to 14 | 0 | #NUM! | 0 | Baseline |
| Male | 15 to 21 | 1 | 0 | 0 | Baseline |
| Male | 22 to 35 | 0 | #NUM! | 0 | Baseline |
| Female | 15 to 21 | 0 | #NUM! | 0 | Baseline |
| Male | 15 to 21 | 0 | #NUM! | 0 | Baseline |
| Female | 15 to 21 | 0 | #NUM! | 0 | Baseline |
| Female | 15 to 21 | 0 | #NUM! | 0 | Baseline |
| Female | 15 to 21 | 0 | #NUM! | 0 | Baseline |
| Female | 5 to 14 | 0 | #NUM! | 0 | Baseline |

| Female | 22 to 35 | 0 | #NUM! | 0 | Baseline |
| --- | --- | --- | --- | --- | --- |
| Male | 22 to 35 | 0 | #NUM! | 0 | Baseline |
| Male | 22 to 35 | 0 | #NUM! | 0 | Baseline |
| Female | 22 to 35 | 0 | #NUM! | 0 | Baseline |
| Female | 22 to 35 | 0 | #NUM! | 0 | Baseline |
| Female | 22 to 35 | 0 | #NUM! | 0 | Baseline |
| Male | 22 to 35 | 0 | #NUM! | 0 | Baseline |
| Male | 35+ | 0 | #NUM! | 0 | Baseline |
| Male | 35+ | 0 | #NUM! | 0 | Baseline |
| Male | 35+ | 0 | #NUM! | 0 | Baseline |
| Male | 22 to 35 | 0 | #NUM! | 0 | Baseline |
| Female | 5 to 14 | 0 | #NUM! | 0 | Baseline |
| Male | 35+ | 0 | #NUM! | 0 | Baseline |
| Male | 5 to 14 | 0 | #NUM! | 0 | Baseline |
| Male | 35+ | 0 | #NUM! | 0 | Baseline |
| Male | 1 to 4 | 0 | #NUM! | 0 | Baseline |
| Male | 22 to 35 | 0 | #NUM! | 0 | Baseline |
| Female | 22 to 35 | 0 | #NUM! | 0 | Baseline |
| Male | 5 to 14 | 0 | #NUM! | 0 | Baseline |
| Female | 5 to 14 | 0 | #NUM! | 0 | Baseline |
| Male | 1 to 4 | 0 | #NUM! | 0 | Baseline |
| Male | 35+ | 0 | #NUM! | 0 | Baseline |
| Male | 35+ | 0 | #NUM! | 0 | Baseline |
| Female | 22 to 35 | 0 | #NUM! | 0 | Baseline |
| Male | 35+ | 0 | #NUM! | 0 | Baseline |
| Male | 35+ | 0 | #NUM! | 0 | Baseline |
| Female | 22 to 35 | 0 | #NUM! | 0 | Baseline |
| Male | 5 to 14 | 0 | #NUM! | 0 | Baseline |
| Male | 1 to 4 | 0 | #NUM! | 0 | Baseline |
| Male | 5 to 14 | 0 | #NUM! | 0 | Baseline |
| Female | 35+ | 0 | #NUM! | 0 | Baseline |
| Female | 22 to 35 | 0 | #NUM! | 0 | Baseline |
| Male | 1 to 4 | 0 | #NUM! | 0 | Baseline |
| Male | 35+ | 0 | #NUM! | 0 | Baseline |
| Female | 22 to 35 | 0 | #NUM! | 0 | Baseline |
| Male | 5 to 14 | 0 | #NUM! | 0 | Baseline |
| Female | 35+ | 0 | #NUM! | 0 | Baseline |
| Male | 35+ | 0 | #NUM! | 0 | Baseline |
| Male | 35+ | 0 | #NUM! | 0 | Baseline |
| Female | 35+ | 0 | #NUM! | 0 | Baseline |
| Female | 22 to 35 | 0 | #NUM! | 0 | Baseline |
| Male | 35+ | 0 | #NUM! | 0 | Baseline |
| Male | 5 to 14 | 0 | #NUM! | 0 | Baseline |
| Female | 15 to 21 | 0 | #NUM! | 0 | Baseline |
| Female | 35+ | 0 | #NUM! | 0 | Baseline |
| Male | 5 to 14 | 0 | #NUM! | 0 | Baseline |
| Male | 1 to 4 | 0 | #NUM! | 0 | Baseline |
| Female | 1 to 4 | 0 | #NUM! | 0 | Baseline |
| Female | 35+ | 0 | #NUM! | 0 | Baseline |
| Female | 35+ | 0 | #NUM! | 0 | Baseline |

| Female | 22 to 35 | 0 | #NUM! | 0 | Baseline |
| --- | --- | --- | --- | --- | --- |
| Male | 1 to 4 | 0 | #NUM! | 0 | Baseline |
| Female | 22 to 35 | 0 | #NUM! | 0 | Baseline |
| Male | 5 to 14 | 0 | #NUM! | 0 | Baseline |
| Female | 22 to 35 | 0 | #NUM! | 0 | Baseline |
| Male | 15 to 21 | 0 | #NUM! | 0 | Baseline |
| Female | 5 to 14 | 0 | #NUM! | 0 | Baseline |
| Male | 15 to 21 | 0 | #NUM! | 0 | Baseline |
| Male | 15 to 21 | 0 | #NUM! | 0 | Baseline |
| Male | 15 to 21 | 0 | #NUM! | 0 | Baseline |
| Male | 15 to 21 | 0 | #NUM! | 0 | Baseline |
| Female | 15 to 21 | 0 | #NUM! | 0 | Baseline |
| Male | 15 to 21 | 0 | #NUM! | 0 | Baseline |
| Female | 5 to 14 | 0 | #NUM! | 0 | Baseline |
| Male | 15 to 21 | 0 | #NUM! | 0 | Baseline |
| Male | 15 to 21 | 0 | #NUM! | 0 | Baseline |
| Female | 15 to 21 | 0 | #NUM! | 0 | Baseline |
| Female | 15 to 21 | 0 | #NUM! | 0 | Baseline |
| Female | 15 to 21 | 0 | #NUM! | 0 | Baseline |
| Male | 5 to 14 | 0 | #NUM! | 0 | Baseline |
| Male | 15 to 21 | 0 | #NUM! | 0 | Baseline |
| Male | 22 to 35 | 0 | #NUM! | 0 | Baseline |
| Female | 15 to 21 | 0 | #NUM! | 0 | Baseline |
| Male | 15 to 21 | 0 | #NUM! | 0 | Baseline |
| Male | 15 to 21 | 0 | #NUM! | 0 | Baseline |
| Female | 5 to 14 | 0 | #NUM! | 0 | Baseline |
| Female | 5 to 14 | 0 | #NUM! | 0 | Baseline |
| Female | 15 to 21 | 0 | #NUM! | 0 | Baseline |
| Male | 5 to 14 | 0 | #NUM! | 0 | Baseline |
| Male | 35+ | 0 | #NUM! | 0 | Baseline |
| Female | 15 to 21 | 0 | #NUM! | 0 | Baseline |
| Male | 5 to 14 | 0 | #NUM! | 0 | Baseline |
| Male | 35+ | 0 | #NUM! | 0 | Baseline |
| Male | 15 to 21 | 0 | #NUM! | 0 | Baseline |
| Female | 35+ | 0 | #NUM! | 0 | Baseline |
| Female | 22 to 35 | 0 | #NUM! | 0 | Baseline |
| Male | 22 to 35 | 0 | #NUM! | 0 | Baseline |
| Female | 15 to 21 | 0 | #NUM! | 0 | Baseline |
| Male | 15 to 21 | 0 | #NUM! | 0 | Baseline |
| Female | 5 to 14 | 0 | #NUM! | 0 | Baseline |
| Male | 5 to 14 | 0 | #NUM! | 0 | Baseline |
| Male | 15 to 21 | 0 | #NUM! | 0 | Baseline |
| Male | 1 to 4 | 0 | #NUM! | 0 | Baseline |
| Male | 1 to 4 | 0 | #NUM! | 0 | Baseline |
| Male | 5 to 14 | 0 | #NUM! | 0 | Baseline |
| Male | 5 to 14 | 0 | #NUM! | 0 | Baseline |
| Female | 5 to 14 | 0 | #NUM! | 0 | Baseline |
| Female | 35+ | 0 | #NUM! | 0 | Baseline |
| Male | 15 to 21 | 0 | #NUM! | 0 | Baseline |
| Male | 35+ | 0 | #NUM! | 0 | Baseline |

| Male | 35+ | 0 | #NUM! | 0 | Baseline |
| --- | --- | --- | --- | --- | --- |
| Male | 5 to 14 | 0 | #NUM! | 0 | Baseline |
| Female | 22 to 35 | 0 | #NUM! | 0 | Baseline |
| Female | 22 to 35 | 0 | #NUM! | 0 | Baseline |
| Male | 35+ | 0 | #NUM! | 0 | Baseline |
| Female | 35+ | 0 | #NUM! | 0 | Baseline |
| Male | 22 to 35 | 0 | #NUM! | 0 | Baseline |
| Female | 22 to 35 | 0 | #NUM! | 0 | Baseline |
| Male | 5 to 14 | 0 | #NUM! | 0 | Baseline |
| Female | 35+ | 0 | #NUM! | 0 | Baseline |
| Male | 1 to 4 | 0 | #NUM! | 0 | Baseline |
| Male | 22 to 35 | 0 | #NUM! | 0 | Baseline |
| Female | 35+ | 0 | #NUM! | 0 | Baseline |
| Male | 5 to 14 | 0 | #NUM! | 0 | Baseline |
| Female | 22 to 35 | 0 | #NUM! | 0 | Baseline |
| Female | 35+ | 0 | #NUM! | 0 | Baseline |
| Male | 5 to 14 | 0 | #NUM! | 0 | Baseline |
| Female | 15 to 21 | 0 | #NUM! | 0 | Baseline |
| Female | 15 to 21 | 0 | #NUM! | 0 | Baseline |
| Male | 22 to 35 | 0 | #NUM! | 0 | Baseline |
| Male | 5 to 14 | 0 | #NUM! | 0 | Baseline |
| Male | 15 to 21 | 0 | #NUM! | 0 | Baseline |
| Female | 5 to 14 | 0 | #NUM! | 0 | Baseline |
| Female | 15 to 21 | 0 | #NUM! | 0 | Baseline |
| Male | 35+ | 0 | #NUM! | 0 | Baseline |
| Female | 5 to 14 | 0 | #NUM! | 0 | Baseline |
| Female | 15 to 21 | 0 | #NUM! | 0 | Baseline |
| Male | 5 to 14 | 0 | #NUM! | 0 | Baseline |
| Female | 15 to 21 | 0 | #NUM! | 0 | Baseline |
| Male | 5 to 14 | 0 | #NUM! | 0 | Baseline |
| Female | 35+ | 0 | #NUM! | 0 | Baseline |
| Female | 15 to 21 | 0 | #NUM! | 0 | Baseline |
| Male | 5 to 14 | 0 | #NUM! | 0 | Baseline |
| Female | 22 to 35 | 0 | #NUM! | 0 | Baseline |
| Female | 35+ | 0 | #NUM! | 0 | Baseline |
| Female | 35+ | 0 | #NUM! | 0 | Baseline |
| Male | 1 to 4 | 0 | #NUM! | 0 | Baseline |
| Male | 1 to 4 | 0 | #NUM! | 0 | Baseline |
| Female | 35+ | 0 | #NUM! | 0 | Baseline |
| Male | 15 to 21 | 0 | #NUM! | 0 | Baseline |
| Female | 15 to 21 | 0 | #NUM! | 0 | Baseline |
| Female | 5 to 14 | 0 | #NUM! | 0 | Baseline |
| Male | 5 to 14 | 0 | #NUM! | 0 | Baseline |
| Male | 15 to 21 | 1 | 0 | 0 | Baseline |
| Male | 15 to 21 | 0 | #NUM! | 0 | Baseline |
| Female | 15 to 21 | 0 | #NUM! | 0 | Baseline |
| Male | 15 to 21 | 0 | #NUM! | 0 | Baseline |
| Male | 22 to 35 | 0 | #NUM! | 0 | Baseline |
| Female | 22 to 35 | 0 | #NUM! | 0 | Baseline |
| Male | 22 to 35 | 0 | #NUM! | 0 | Baseline |

| Female | 5 to 14 | 0 | #NUM! | 0 | Baseline |
| --- | --- | --- | --- | --- | --- |
| Male | 22 to 35 | 0 | #NUM! | 0 | Baseline |
| Male | 15 to 21 | 0 | #NUM! | 0 | Baseline |
| Male | 22 to 35 | 0 | #NUM! | 0 | Baseline |
| Female | 22 to 35 | 0 | #NUM! | 0 | Baseline |
| Female | 15 to 21 | 0 | #NUM! | 0 | Baseline |
| Male | 22 to 35 | 0 | #NUM! | 0 | Baseline |
| Male | 15 to 21 | 0 | #NUM! | 0 | Baseline |
| Male | 22 to 35 | 0 | #NUM! | 0 | Baseline |
| Male | 5 to 14 | 0 | #NUM! | 0 | Baseline |
| Male | 22 to 35 | 0 | #NUM! | 0 | Baseline |
| Male | 35+ | 0 | #NUM! | 0 | Baseline |
| Male | 22 to 35 | 0 | #NUM! | 0 | Baseline |
| Female | 5 to 14 | 0 | #NUM! | 0 | Baseline |
| Female | 15 to 21 | 0 | #NUM! | 0 | Baseline |
| Male | 5 to 14 | 0 | #NUM! | 0 | Baseline |
| Female | 15 to 21 | 0 | #NUM! | 0 | Baseline |
| Female | 15 to 21 | 0 | #NUM! | 0 | Baseline |
| Female | 5 to 14 | 0 | #NUM! | 0 | Baseline |
| Male | 35+ | 0 | #NUM! | 0 | Baseline |
| Female | 15 to 21 | 0 | #NUM! | 0 | Baseline |
| Male | 22 to 35 | 0 | #NUM! | 0 | Baseline |
| Female | 22 to 35 | 0 | #NUM! | 0 | Baseline |
| Female | 35+ | 0 | #NUM! | 0 | Baseline |
| Female | 22 to 35 | 0 | #NUM! | 0 | Baseline |
| Male | 15 to 21 | 0 | #NUM! | 0 | Baseline |
| Male | 35+ | 0 | #NUM! | 0 | Baseline |
| Male | 22 to 35 | 0 | #NUM! | 0 | Baseline |
| Male | 15 to 21 | 0 | #NUM! | 0 | Baseline |
| Female | 5 to 14 | 0 | #NUM! | 0 | Baseline |
| Male | 5 to 14 | 0 | #NUM! | 0 | Baseline |
| Female | 15 to 21 | 0 | #NUM! | 0 | Baseline |
| Female | 22 to 35 | 0 | #NUM! | 0 | Baseline |
| Female | 15 to 21 | 0 | #NUM! | 0 | Baseline |
| Male | 15 to 21 | 0 | #NUM! | 0 | Baseline |
| Female | 22 to 35 | 0 | #NUM! | 0 | Baseline |
| Female | 15 to 21 | 0 | #NUM! | 0 | Baseline |
| Female | 35+ | 0 | #NUM! | 0 | Baseline |
| Male | 15 to 21 | 0 | #NUM! | 0 | Baseline |
| Female | 35+ | 0 | #NUM! | 0 | Baseline |
| Female | 15 to 21 | 0 | #NUM! | 0 | Baseline |
| Male | 22 to 35 | 0 | #NUM! | 0 | Baseline |
| Male | 15 to 21 | 0 | #NUM! | 0 | Baseline |
| Male | 15 to 21 | 0 | #NUM! | 0 | Baseline |
| Male | 22 to 35 | 0 | #NUM! | 0 | Baseline |
| Female | 22 to 35 | 0 | #NUM! | 0 | Baseline |
| Male | 15 to 21 | 0 | #NUM! | 0 | Baseline |
| Female | 15 to 21 | 0 | #NUM! | 0 | Baseline |
| Male | 15 to 21 | 0 | #NUM! | 0 | Baseline |
| Male | 22 to 35 | 0 | #NUM! | 0 | Baseline |

| Female | 15 to 21 | 0 | #NUM! | 0 | Baseline |
| --- | --- | --- | --- | --- | --- |
| Female | 15 to 21 | 0 | #NUM! | 0 | Baseline |
| Male | 15 to 21 | 0 | #NUM! | 0 | Baseline |
| Male | 15 to 21 | 0 | #NUM! | 0 | Baseline |
| Male | 15 to 21 | 0 | #NUM! | 0 | Baseline |
| Male | 15 to 21 | 0 | #NUM! | 0 | Baseline |
| Male | 5 to 14 | 0 | #NUM! | 0 | Baseline |
| Male | 15 to 21 | 0 | #NUM! | 0 | Baseline |
| Male | 22 to 35 | 0 | #NUM! | 0 | Baseline |
| Male | 5 to 14 | 0 | #NUM! | 0 | Baseline |
| Female | 5 to 14 | 0 | #NUM! | 0 | Baseline |
| Male | 15 to 21 | 0 | #NUM! | 0 | Baseline |
| Female | 5 to 14 | 0 | #NUM! | 0 | Baseline |
| Male | 5 to 14 | 0 | #NUM! | 0 | Baseline |
| Male | 5 to 14 | 0 | #NUM! | 0 | Baseline |
| Female | 5 to 14 | 0 | #NUM! | 0 | Baseline |
| Female | 15 to 21 | 0 | #NUM! | 0 | Baseline |
| Male | 5 to 14 | 0 | #NUM! | 0 | Baseline |
| Female | 5 to 14 | 0 | #NUM! | 0 | Baseline |
| Male | 5 to 14 | 0 | #NUM! | 0 | Baseline |
| Male | 15 to 21 | 0 | #NUM! | 0 | Baseline |
| Male | 22 to 35 | 0 | #NUM! | 0 | Baseline |
| Female | 5 to 14 | 0 | #NUM! | 0 | Baseline |
| Female | 22 to 35 | 0 | #NUM! | 0 | Baseline |
| Female | 22 to 35 | 0 | #NUM! | 0 | Baseline |
| Male | 5 to 14 | 0 | #NUM! | 0 | Baseline |
| Female | 22 to 35 | 0 | #NUM! | 0 | Baseline |
| Male | 1 to 4 | 0 | #NUM! | 0 | Baseline |
| Male | 22 to 35 | 0 | #NUM! | 0 | Baseline |
| Male | 5 to 14 | 0 | #NUM! | 0 | Baseline |
| Female | 15 to 21 | 0 | #NUM! | 0 | Baseline |
| Male | 35+ | 0 | #NUM! | 0 | Baseline |
| Female | 5 to 14 | 0 | #NUM! | 0 | Baseline |
| Male | 22 to 35 | 0 | #NUM! | 0 | Baseline |
| Female | 22 to 35 | 0 | #NUM! | 0 | Baseline |
| Male | 5 to 14 | 0 | #NUM! | 0 | Baseline |
| Female | 22 to 35 | 0 | #NUM! | 0 | Baseline |
| Female | 22 to 35 | 0 | #NUM! | 0 | Baseline |
| Male | 15 to 21 | 0 | #NUM! | 0 | Baseline |
| Female | 15 to 21 | 0 | #NUM! | 0 | Baseline |
| Female | 5 to 14 | 0 | #NUM! | 0 | Baseline |
| Female | 5 to 14 | 0 | #NUM! | 0 | Baseline |
| Male | 5 to 14 | 0 | #NUM! | 0 | Baseline |
| Male | 22 to 35 | 0 | #NUM! | 0 | Baseline |
| Male | 15 to 21 | 0 | #NUM! | 0 | Baseline |
| Male | 22 to 35 | 0 | #NUM! | 0 | Baseline |
| Male | 5 to 14 | 0 | #NUM! | 0 | Baseline |
| Male | 5 to 14 | 0 | #NUM! | 0 | Baseline |
| Male | 15 to 21 | 0 | #NUM! | 0 | Baseline |
| Male | 5 to 14 | 0 | #NUM! | 0 | Baseline |

| Male | 15 to 21 | 0 | #NUM! | 0 | Baseline |
| --- | --- | --- | --- | --- | --- |
| Male | 15 to 21 | 0 | #NUM! | 0 | Baseline |
| Male | 5 to 14 | 0 | #NUM! | 0 | Baseline |
| Female | 5 to 14 | 0 | #NUM! | 0 | Baseline |
| Male | 1 to 4 | 0 | #NUM! | 0 | Baseline |
| Male | 15 to 21 | 0 | #NUM! | 0 | Baseline |
| Male | 5 to 14 | 0 | #NUM! | 0 | Baseline |
| Male | 5 to 14 | 0 | #NUM! | 0 | Baseline |
| Female | 22 to 35 | 0 | #NUM! | 0 | Baseline |
| Male | 22 to 35 | 0 | #NUM! | 0 | Baseline |
| Female | 15 to 21 | 0 | #NUM! | 0 | Baseline |
| Male | 15 to 21 | 0 | #NUM! | 0 | Baseline |
| Female | 22 to 35 | 0 | #NUM! | 0 | Baseline |
| Male | 5 to 14 | 0 | #NUM! | 0 | Baseline |
| Male | 5 to 14 | 0 | #NUM! | 0 | Baseline |
| Male | 35+ | 0 | #NUM! | 0 | Baseline |
| Male | 15 to 21 | 0 | #NUM! | 0 | Baseline |
| Male | 5 to 14 | 0 | #NUM! | 0 | Baseline |
| Male | 5 to 14 | 0 | #NUM! | 0 | Baseline |
| Female | 15 to 21 | 0 | #NUM! | 0 | Baseline |
| Female | 15 to 21 | 0 | #NUM! | 0 | Baseline |
| Female | 5 to 14 | 0 | #NUM! | 0 | Baseline |
| Male | 5 to 14 | 0 | #NUM! | 0 | Baseline |
| Male | 5 to 14 | 0 | #NUM! | 0 | Baseline |
| Female | 15 to 21 | 0 | #NUM! | 0 | Baseline |
| Female | 15 to 21 | 0 | #NUM! | 0 | Baseline |
| Female | 5 to 14 | 0 | #NUM! | 0 | Baseline |
| Female | 5 to 14 | 0 | #NUM! | 0 | Baseline |
| Male | 15 to 21 | 0 | #NUM! | 0 | Baseline |
| Male | 15 to 21 | 0 | #NUM! | 0 | Baseline |
| Male | 15 to 21 | 0 | #NUM! | 0 | Baseline |
| Female | 5 to 14 | 0 | #NUM! | 0 | Baseline |
| Male | 5 to 14 | 0 | #NUM! | 0 | Baseline |
| Female | 15 to 21 | 0 | #NUM! | 0 | Baseline |
| Female | 35+ | 0 | #NUM! | 0 | Baseline |
| Male | 22 to 35 | 0 | #NUM! | 0 | Baseline |
| Male | 15 to 21 | 0 | #NUM! | 0 | Baseline |
| Male | 22 to 35 | 0 | #NUM! | 0 | Baseline |
| Male | 15 to 21 | 0 | #NUM! | 0 | Baseline |
| Male | 1 to 4 | 0 | #NUM! | 0 | Baseline |
| Male | 1 to 4 | 0 | #NUM! | 0 | Baseline |
| Female | 22 to 35 | 0 | #NUM! | 0 | Baseline |
| Male | 1 to 4 | 0 | #NUM! | 0 | Baseline |
| Male | 1 to 4 | 0 | #NUM! | 0 | Baseline |
| Male | 1 to 4 | 0 | #NUM! | 0 | Baseline |
| Male | 1 to 4 | 0 | #NUM! | 0 | Baseline |
| Male | 1 to 4 | 0 | #NUM! | 0 | Baseline |
| Male | 1 to 4 | 0 | #NUM! | 0 | Baseline |
| Male | 1 to 4 | 0 | #NUM! | 0 | Baseline |
| Male | 1 to 4 | 0 | #NUM! | 0 | Baseline |

| Male | 1 to 4 | 0 | #NUM! | 0 | Baseline |
| --- | --- | --- | --- | --- | --- |
| Male | 1 to 4 | 0 | #NUM! | 0 | Baseline |
| Male | 1 to 4 | 0 | #NUM! | 0 | Baseline |
| Male | 1 to 4 | 0 | #NUM! | 0 | Baseline |
| Male | 1 to 4 | 0 | #NUM! | 0 | Baseline |
| Female | 15 to 21 | 0 | #NUM! | 0 | Baseline |
| Male | 22 to 35 | 0 | #NUM! | 0 | Baseline |
| Male | 35+ | 0 | #NUM! | 0 | Baseline |
| Female | 22 to 35 | 0 | #NUM! | 0 | Baseline |
| Male | 22 to 35 | 0 | #NUM! | 0 | Baseline |
| Male | 1 to 4 | 0 | #NUM! | 0 | Baseline |
| Female | 15 to 21 | 0 | #NUM! | 0 | Baseline |
| Male | 1 to 4 | 0 | #NUM! | 0 | Baseline |
| Female | 22 to 35 | 0 | #NUM! | 0 | Baseline |
| Male | 1 to 4 | 0 | #NUM! | 0 | Baseline |
| Female | 22 to 35 | 0 | #NUM! | 0 | Baseline |
| Male | 15 to 21 | 0 | #NUM! | 0 | Baseline |
| Male | 5 to 14 | 0 | #NUM! | 0 | Baseline |
| Female | 35+ | 0 | #NUM! | 0 | Baseline |
| Female | 22 to 35 | 0 | #NUM! | 0 | Baseline |
| Male | 35+ | 0 | #NUM! | 0 | Baseline |
| Male | 22 to 35 | 0 | #NUM! | 0 | Baseline |
| Male | 22 to 35 | 0 | #NUM! | 0 | Baseline |
| Male | 5 to 14 | 0 | #NUM! | 0 | Baseline |
| Male | 5 to 14 | 0 | #NUM! | 0 | Baseline |
| Male | 15 to 21 | 0 | #NUM! | 0 | Baseline |
| Female | 15 to 21 | 0 | #NUM! | 0 | Baseline |
| Male | 22 to 35 | 0 | #NUM! | 0 | Baseline |
| Male | 5 to 14 | 0 | #NUM! | 0 | Baseline |
| Female | 5 to 14 | 0 | #NUM! | 0 | Baseline |
| Female | 22 to 35 | 0 | #NUM! | 0 | Baseline |
| Male | 15 to 21 | 0 | #NUM! | 0 | Baseline |
| Male | 5 to 14 | 0 | #NUM! | 0 | Baseline |
| Female | 22 to 35 | 0 | #NUM! | 0 | Baseline |
| Male | 22 to 35 | 0 | #NUM! | 0 | Baseline |
| Female | 22 to 35 | 0 | #NUM! | 0 | Baseline |
| Male | 5 to 14 | 0 | #NUM! | 0 | Baseline |
| Male | 22 to 35 | 0 | #NUM! | 0 | Baseline |
| Male | 15 to 21 | 0 | #NUM! | 0 | Baseline |
| Male | 15 to 21 | 0 | #NUM! | 0 | Baseline |
| Male | 15 to 21 | 0 | #NUM! | 0 | Baseline |
| Male | 15 to 21 | 0 | #NUM! | 0 | Baseline |
| Male | 1 to 4 | 0 | #NUM! | 0 | Baseline |
| Female | 15 to 21 | 0 | #NUM! | 0 | Baseline |
| Male | 15 to 21 | 0 | #NUM! | 0 | Baseline |
| Male | 22 to 35 | 0 | #NUM! | 0 | Baseline |
| Female | 5 to 14 | 0 | #NUM! | 0 | Baseline |
| Male | 15 to 21 | 0 | #NUM! | 0 | Baseline |
| Female | 15 to 21 | 0 | #NUM! | 0 | Baseline |
| Male | 15 to 21 | 0 | #NUM! | 0 | Baseline |

| Male | 15 to 21 | 0 | #NUM! | 0 | Baseline |
| --- | --- | --- | --- | --- | --- |
| Male | 15 to 21 | 0 | #NUM! | 0 | Baseline |
| Male | 1 to 4 | 0 | #NUM! | 0 | Baseline |
| Male | 5 to 14 | 0 | #NUM! | 0 | Baseline |
| Male | 15 to 21 | 0 | #NUM! | 0 | Baseline |
| Male | 5 to 14 | 0 | #NUM! | 0 | Baseline |
| Male | 35+ | 0 | #NUM! | 0 | Baseline |
| Male | 5 to 14 | 0 | #NUM! | 0 | Baseline |
| Male | 15 to 21 | 0 | #NUM! | 0 | Baseline |
| Male | 22 to 35 | 0 | #NUM! | 0 | Baseline |
| Male | 5 to 14 | 0 | #NUM! | 0 | Baseline |
| Male | 15 to 21 | 0 | #NUM! | 0 | Baseline |
| Male | 1 to 4 | 0 | #NUM! | 0 | Baseline |
| Male | 1 to 4 | 0 | #NUM! | 0 | Baseline |
| Male | 1 to 4 | 0 | #NUM! | 0 | Baseline |
| Male | 1 to 4 | 0 | #NUM! | 0 | Baseline |
| Male | 1 to 4 | 0 | #NUM! | 0 | Baseline |
| Female | 22 to 35 | 0 | #NUM! | 0 | Baseline |
| Male | 1 to 4 | 0 | #NUM! | 0 | Baseline |
| Male | 5 to 14 | 0 | #NUM! | 0 | Baseline |
| Female | 22 to 35 | 0 | #NUM! | 0 | Baseline |
| Male | 15 to 21 | 0 | #NUM! | 0 | Baseline |
| Male | 1 to 4 | 0 | #NUM! | 0 | Baseline |
| Male | 15 to 21 | 0 | #NUM! | 0 | Baseline |
| Male | 1 to 4 | 0 | #NUM! | 0 | Baseline |
| Female | 5 to 14 | 0 | #NUM! | 0 | Baseline |
| Male | 1 to 4 | 0 | #NUM! | 0 | Baseline |
| Male | 1 to 4 | 0 | #NUM! | 0 | Baseline |
| Female | 15 to 21 | 0 | #NUM! | 0 | Baseline |
| Female | 15 to 21 | 0 | #NUM! | 0 | Baseline |
| Male | 5 to 14 | 0 | #NUM! | 0 | Baseline |
| Female | 22 to 35 | 0 | #NUM! | 0 | Baseline |
| Female | 5 to 14 | 0 | #NUM! | 0 | Baseline |
| Male | 1 to 4 | 0 | #NUM! | 0 | Baseline |
| Male | 1 to 4 | 0 | #NUM! | 0 | Baseline |
| Female | 22 to 35 | 0 | #NUM! | 0 | Baseline |
| Male | 1 to 4 | 0 | #NUM! | 0 | Baseline |
| Male | 1 to 4 | 0 | #NUM! | 0 | Baseline |
| Male | 35+ | 0 | #NUM! | 0 | Baseline |
| Male | 1 to 4 | 0 | #NUM! | 0 | Baseline |
| Male | 1 to 4 | 0 | #NUM! | 0 | Baseline |
| Male | 22 to 35 | 0 | #NUM! | 0 | Baseline |
| Male | 1 to 4 | 0 | #NUM! | 0 | Baseline |
| Female | 5 to 14 | 0 | #NUM! | 0 | Baseline |
| Male | 1 to 4 | 0 | #NUM! | 0 | Baseline |
| Male | 1 to 4 | 0 | #NUM! | 0 | Baseline |
| Male | 1 to 4 | 0 | #NUM! | 0 | Baseline |
| Male | 1 to 4 | 0 | #NUM! | 0 | Baseline |
| Male | 1 to 4 | 0 | #NUM! | 0 | Baseline |
| Male | 1 to 4 | 0 | #NUM! | 0 | Baseline |

| Male | 1 to 4 | 0 | #NUM! | 0 | Baseline |
| --- | --- | --- | --- | --- | --- |
| Male | 1 to 4 | 0 | #NUM! | 0 | Baseline |
| Male | 1 to 4 | 0 | #NUM! | 0 | Baseline |
| Male | 1 to 4 | 0 | #NUM! | 0 | Baseline |
| Male | 1 to 4 | 0 | #NUM! | 0 | Baseline |
| Male | 1 to 4 | 0 | #NUM! | 0 | Baseline |
| Male | 1 to 4 | 0 | #NUM! | 0 | Baseline |
| Male | 1 to 4 | 0 | #NUM! | 0 | Baseline |
| Male | 1 to 4 | 0 | #NUM! | 0 | Baseline |
| Male | 22 to 35 | 0 | #NUM! | 0 | Baseline |
| Male | 15 to 21 | 0 | #NUM! | 0 | Baseline |
| Female | 5 to 14 | 0 | #NUM! | 0 | Baseline |
| Male | 15 to 21 | 0 | #NUM! | 0 | Baseline |
| Male | 5 to 14 | 0 | #NUM! | 0 | Baseline |
| Male | 1 to 4 | 0 | #NUM! | 0 | Baseline |
| Male | 1 to 4 | 0 | #NUM! | 0 | Baseline |
| Male | 15 to 21 | 0 | #NUM! | 0 | Baseline |
| Male | 15 to 21 | 0 | #NUM! | 0 | Baseline |
| Male | 15 to 21 | 0 | #NUM! | 0 | Baseline |
| Female | 15 to 21 | 0 | #NUM! | 0 | Baseline |
| Male | 22 to 35 | 0 | #NUM! | 0 | Baseline |
| Male | 15 to 21 | 0 | #NUM! | 0 | Baseline |
| Female | 22 to 35 | 0 | #NUM! | 0 | Baseline |
| Female | 15 to 21 | 0 | #NUM! | 0 | Baseline |
| Male | 15 to 21 | 0 | #NUM! | 0 | Baseline |
| Male | 15 to 21 | 0 | #NUM! | 0 | Baseline |
| Male | 35+ | 0 | #NUM! | 0 | Baseline |
| Male | 15 to 21 | 0 | #NUM! | 0 | Baseline |
| Male | 15 to 21 | 0 | #NUM! | 0 | Baseline |
| Female | 15 to 21 | 0 | #NUM! | 0 | Baseline |
| Male | 22 to 35 | 0 | #NUM! | 0 | Baseline |
| Male | 35+ | 0 | #NUM! | 0 | Baseline |
| Female | 22 to 35 | 0 | #NUM! | 0 | Baseline |
| Female | 22 to 35 | 0 | #NUM! | 0 | Baseline |
| Male | 22 to 35 | 0 | #NUM! | 0 | Baseline |
| Male | 15 to 21 | 0 | #NUM! | 0 | Baseline |
| Male | 22 to 35 | 0 | #NUM! | 0 | Baseline |
| Female | 22 to 35 | 0 | #NUM! | 0 | Baseline |
| Male | 22 to 35 | 0 | #NUM! | 0 | Baseline |
| Male | 15 to 21 | 0 | #NUM! | 0 | Baseline |
| Male | 22 to 35 | 0 | #NUM! | 0 | Baseline |
| Female | 22 to 35 | 0 | #NUM! | 0 | Baseline |
| Female | 22 to 35 | 0 | #NUM! | 0 | Baseline |
| Male | 15 to 21 | 0 | #NUM! | 0 | Baseline |
| Female | 22 to 35 | 0 | #NUM! | 0 | Baseline |
| Male | 15 to 21 | 0 | #NUM! | 0 | Baseline |
| Male | 22 to 35 | 0 | #NUM! | 0 | Baseline |
| Female | 22 to 35 | 0 | #NUM! | 0 | Baseline |
| Female | 35+ | 0 | #NUM! | 0 | Baseline |
| Female | 22 to 35 | 0 | #NUM! | 0 | Baseline |

| Female | 15 to 21 | 0 | #NUM! | 0 | Baseline |
| --- | --- | --- | --- | --- | --- |
| Male | 15 to 21 | 0 | #NUM! | 0 | Baseline |
| Female | 22 to 35 | 0 | #NUM! | 0 | Baseline |
| Male | 22 to 35 | 0 | #NUM! | 0 | Baseline |
| Female | 15 to 21 | 0 | #NUM! | 0 | Baseline |
| Male | 35+ | 0 | #NUM! | 0 | Baseline |
| Male | 15 to 21 | 0 | #NUM! | 0 | Baseline |
| Male | 15 to 21 | 0 | #NUM! | 0 | Baseline |
| Female | 15 to 21 | 0 | #NUM! | 0 | Baseline |
| Female | 22 to 35 | 0 | #NUM! | 0 | Baseline |
| Male | 5 to 14 | 0 | #NUM! | 0 | Baseline |
| Male | 15 to 21 | 0 | #NUM! | 0 | Baseline |
| Female | 35+ | 0 | #NUM! | 0 | Baseline |
| Male | 15 to 21 | 0 | #NUM! | 0 | Baseline |
| Male | 22 to 35 | 0 | #NUM! | 0 | Baseline |
| Male | 15 to 21 | 0 | #NUM! | 0 | Baseline |
| Male | 15 to 21 | 0 | #NUM! | 0 | Baseline |
| Male | 1 to 4 | 0 | #NUM! | 0 | Baseline |
| Male | 22 to 35 | 0 | #NUM! | 0 | Baseline |
| Male | 22 to 35 | 0 | #NUM! | 0 | Baseline |
| Female | 15 to 21 | 0 | #NUM! | 0 | Baseline |
| Male | 22 to 35 | 0 | #NUM! | 0 | Baseline |
| Male | 1 to 4 | 0 | #NUM! | 0 | Baseline |
| Male | 15 to 21 | 0 | #NUM! | 0 | Baseline |
| Male | 15 to 21 | 0 | #NUM! | 0 | Baseline |
| Male | 1 to 4 | 0 | #NUM! | 0 | Baseline |
| Male | 15 to 21 | 0 | #NUM! | 0 | Baseline |
| Male | 1 to 4 | 0 | #NUM! | 0 | Baseline |
| Male | 1 to 4 | 0 | #NUM! | 0 | Baseline |
| Male | 15 to 21 | 0 | #NUM! | 0 | Baseline |
| Male | 15 to 21 | 0 | #NUM! | 0 | Baseline |
| Male | 1 to 4 | 0 | #NUM! | 0 | Baseline |
| Male | 22 to 35 | 0 | #NUM! | 0 | Baseline |
| Male | 22 to 35 | 0 | #NUM! | 0 | Baseline |
| Male | 1 to 4 | 0 | #NUM! | 0 | Baseline |
| Male | 15 to 21 | 0 | #NUM! | 0 | Baseline |
| Male | 1 to 4 | 0 | #NUM! | 0 | Baseline |
| Male | 15 to 21 | 0 | #NUM! | 0 | Baseline |
| Male | 1 to 4 | 0 | #NUM! | 0 | Baseline |
| Male | 15 to 21 | 0 | #NUM! | 0 | Baseline |
| Male | 5 to 14 | 0 | #NUM! | 0 | Baseline |
| Female | 35+ | 0 | #NUM! | 0 | Baseline |
| Male | 5 to 14 | 0 | #NUM! | 0 | Baseline |
| Male | 22 to 35 | 0 | #NUM! | 0 | Baseline |
| Female | 15 to 21 | 0 | #NUM! | 0 | Baseline |
| Male | 15 to 21 | 0 | #NUM! | 0 | Baseline |
| Female | 15 to 21 | 0 | #NUM! | 0 | Baseline |
| Male | 5 to 14 | 0 | #NUM! | 0 | Baseline |
| Male | 1 to 4 | 0 | #NUM! | 0 | Baseline |
| Male | 1 to 4 | 0 | #NUM! | 0 | Baseline |

| Male | 15 to 21 | 0 | #NUM! | 0 | Baseline |
| --- | --- | --- | --- | --- | --- |
| Female | 15 to 21 | 0 | #NUM! | 0 | Baseline |
| Male | 35+ | 0 | #NUM! | 0 | Baseline |
| Male | 15 to 21 | 0 | #NUM! | 0 | Baseline |
| Male | 22 to 35 | 0 | #NUM! | 0 | Baseline |
| Female | 22 to 35 | 0 | #NUM! | 0 | Baseline |
| Male | 15 to 21 | 0 | #NUM! | 0 | Baseline |
| Female | 15 to 21 | 0 | #NUM! | 0 | Baseline |
| Male | 15 to 21 | 0 | #NUM! | 0 | Baseline |
| Male | 22 to 35 | 0 | #NUM! | 0 | Baseline |
| Male | 1 to 4 | 0 | #NUM! | 0 | Baseline |
| Male | 22 to 35 | 0 | #NUM! | 0 | Baseline |
| Male | 22 to 35 | 0 | #NUM! | 0 | Baseline |
| Male | 22 to 35 | 0 | #NUM! | 0 | Baseline |
| Male | 22 to 35 | 0 | #NUM! | 0 | Baseline |
| Female | 22 to 35 | 0 | #NUM! | 0 | Baseline |
| Female | 22 to 35 | 0 | #NUM! | 0 | Baseline |
| Female | 35+ | 0 | #NUM! | 0 | Baseline |
| Female | 35+ | 0 | #NUM! | 0 | Baseline |
| Female | 35+ | 0 | #NUM! | 0 | Baseline |
| Female | 35+ | 0 | #NUM! | 0 | Baseline |
| Female | 35+ | 0 | #NUM! | 0 | Baseline |
| Male | 5 to 14 | 0 | #NUM! | 0 | Baseline |
| Female | 5 to 14 | 0 | #NUM! | 0 | Baseline |
| Male | 5 to 14 | 0 | #NUM! | 0 | Baseline |
| Female | 15 to 21 | 0 | #NUM! | 0 | Baseline |
| Male | 5 to 14 | 0 | #NUM! | 0 | Baseline |
| Male | 5 to 14 | 0 | #NUM! | 0 | Baseline |
| Female | 5 to 14 | 0 | #NUM! | 0 | Baseline |
| Male | 5 to 14 | 0 | #NUM! | 0 | Baseline |
| Male | 5 to 14 | 0 | #NUM! | 0 | Baseline |
| Male | 5 to 14 | 0 | #NUM! | 0 | Baseline |
| Female | 35+ | 0 | #NUM! | 0 | Baseline |
| Male | 5 to 14 | 0 | #NUM! | 0 | Baseline |
| Female | 35+ | 0 | #NUM! | 0 | Baseline |
| Male | 5 to 14 | 0 | #NUM! | 0 | Baseline |
| Female | 5 to 14 | 0 | #NUM! | 0 | Baseline |
| Female | 35+ | 0 | #NUM! | 0 | Baseline |
| Female | 22 to 35 | 0 | #NUM! | 0 | Baseline |
| Female | 5 to 14 | 0 | #NUM! | 0 | Baseline |
| Female | 5 to 14 | 0 | #NUM! | 0 | Baseline |
| Male | 5 to 14 | 0 | #NUM! | 0 | Baseline |
| Female | 22 to 35 | 0 | #NUM! | 0 | Baseline |
| Female | 22 to 35 | 0 | #NUM! | 0 | Baseline |
| Male | 5 to 14 | 0 | #NUM! | 0 | Baseline |
| Female | 5 to 14 | 0 | #NUM! | 0 | Baseline |
| Female | 5 to 14 | 0 | #NUM! | 0 | Baseline |
| Female | 22 to 35 | 0 | #NUM! | 0 | Baseline |
| Female | 35+ | 0 | #NUM! | 0 | Baseline |
| Female | 5 to 14 | 0 | #NUM! | 0 | Baseline |

| Female | 5 to 14 | 0 | #NUM! | 0 | Baseline |
| --- | --- | --- | --- | --- | --- |
| Female | 15 to 21 | 0 | #NUM! | 0 | Baseline |
| Male | 5 to 14 | 0 | #NUM! | 0 | Baseline |
| Male | 5 to 14 | 0 | #NUM! | 0 | Baseline |
| Female | 5 to 14 | 0 | #NUM! | 0 | Baseline |
| Male | 5 to 14 | 0 | #NUM! | 0 | Baseline |
| Female | 22 to 35 | 0 | #NUM! | 0 | Baseline |
| Female | 5 to 14 | 0 | #NUM! | 0 | Baseline |
| Female | 5 to 14 | 0 | #NUM! | 0 | Baseline |
| Female | 5 to 14 | 0 | #NUM! | 0 | Baseline |
| Female | 5 to 14 | 0 | #NUM! | 0 | Baseline |
| Male | 5 to 14 | 0 | #NUM! | 0 | Baseline |
| Female | 22 to 35 | 0 | #NUM! | 0 | Baseline |
| Male | 5 to 14 | 0 | #NUM! | 0 | Baseline |
| Female | 5 to 14 | 0 | #NUM! | 0 | Baseline |
| Female | 15 to 21 | 0 | #NUM! | 0 | Baseline |
| Female | 5 to 14 | 0 | #NUM! | 0 | Baseline |
| Male | 22 to 35 | 0 | #NUM! | 0 | Baseline |
| Female | 35+ | 0 | #NUM! | 0 | Baseline |
| Female | 35+ | 0 | #NUM! | 0 | Baseline |
| Male | 35+ | 0 | #NUM! | 0 | Baseline |
| Male | 35+ | 0 | #NUM! | 0 | Baseline |
| Male | 22 to 35 | 0 | #NUM! | 0 | Baseline |
| Female | 5 to 14 | 0 | #NUM! | 0 | Baseline |
| Male | 5 to 14 | 0 | #NUM! | 0 | Baseline |
| Male | 5 to 14 | 0 | #NUM! | 0 | Baseline |
| Female | 22 to 35 | 0 | #NUM! | 0 | Baseline |
| Male | 5 to 14 | 0 | #NUM! | 0 | Baseline |
| Female | 5 to 14 | 0 | #NUM! | 0 | Baseline |
| Male | 35+ | 0 | #NUM! | 0 | Baseline |
| Male | 35+ | 0 | #NUM! | 0 | Baseline |
| Female | 35+ | 0 | #NUM! | 0 | Baseline |
| Male | 5 to 14 | 0 | #NUM! | 0 | Baseline |
| Female | 35+ | 0 | #NUM! | 0 | Baseline |
| Female | 22 to 35 | 0 | #NUM! | 0 | Baseline |
| Female | 5 to 14 | 0 | #NUM! | 0 | Baseline |
| Female | 5 to 14 | 0 | #NUM! | 0 | Baseline |
| Female | 15 to 21 | 0 | #NUM! | 0 | Baseline |
| Female | 5 to 14 | 0 | #NUM! | 0 | Baseline |
| Female | 35+ | 0 | #NUM! | 0 | Baseline |
| Female | 35+ | 0 | #NUM! | 0 | Baseline |
| Female | 22 to 35 | 0 | #NUM! | 0 | Baseline |
| Female | 5 to 14 | 0 | #NUM! | 0 | Baseline |
| Male | 5 to 14 | 0 | #NUM! | 0 | Baseline |
| Female | 22 to 35 | 0 | #NUM! | 0 | Baseline |
| Female | 35+ | 0 | #NUM! | 0 | Baseline |
| Male | 5 to 14 | 0 | #NUM! | 0 | Baseline |
| Female | 5 to 14 | 0 | #NUM! | 0 | Baseline |
| Female | 5 to 14 | 0 | #NUM! | 0 | Baseline |
| Female | 22 to 35 | 0 | #NUM! | 0 | Baseline |

| Male | 5 to 14 | 0 | #NUM! | 0 | Baseline |
| --- | --- | --- | --- | --- | --- |
| Female | 35+ | 0 | #NUM! | 0 | Baseline |
| Female | 35+ | 0 | #NUM! | 0 | Baseline |
| Female | 35+ | 0 | #NUM! | 0 | Baseline |
| Female | 22 to 35 | 0 | #NUM! | 0 | Baseline |
| Female | 5 to 14 | 0 | #NUM! | 0 | Baseline |
| Female | 5 to 14 | 0 | #NUM! | 0 | Baseline |
| Female | 35+ | 0 | #NUM! | 0 | Baseline |
| Female | 5 to 14 | 0 | #NUM! | 0 | Baseline |
| Female | 15 to 21 | 0 | #NUM! | 0 | Baseline |
| Male | 15 to 21 | 0 | #NUM! | 0 | Baseline |
| Male | 15 to 21 | 0 | #NUM! | 0 | Baseline |
| Male | 15 to 21 | 0 | #NUM! | 0 | Baseline |
| Female | 5 to 14 | 0 | #NUM! | 0 | Baseline |
| Female | 5 to 14 | 0 | #NUM! | 0 | Baseline |
| Female | 15 to 21 | 0 | #NUM! | 0 | Baseline |
| Male | 5 to 14 | 0 | #NUM! | 0 | Baseline |
| Male | 15 to 21 | 0 | #NUM! | 0 | Baseline |
| Female | 5 to 14 | 0 | #NUM! | 0 | Baseline |
| Male | 5 to 14 | 0 | #NUM! | 0 | Baseline |
| Male | 5 to 14 | 0 | #NUM! | 0 | Baseline |
| Female | 5 to 14 | 0 | #NUM! | 0 | Baseline |
| Female | 15 to 21 | 0 | #NUM! | 0 | Baseline |
| Male | 15 to 21 | 0 | #NUM! | 0 | Baseline |
| Male | 15 to 21 | 0 | #NUM! | 0 | Baseline |
| Female | 15 to 21 | 0 | #NUM! | 0 | Baseline |
| Male | 15 to 21 | 0 | #NUM! | 0 | Baseline |
| Male | 15 to 21 | 0 | #NUM! | 0 | Baseline |
| Female | 15 to 21 | 0 | #NUM! | 0 | Baseline |
| Male | 5 to 14 | 0 | #NUM! | 0 | Baseline |
| Male | 15 to 21 | 0 | #NUM! | 0 | Baseline |
| Male | 5 to 14 | 0 | #NUM! | 0 | Baseline |
| Female | 5 to 14 | 0 | #NUM! | 0 | Baseline |
| Female | 15 to 21 | 0 | #NUM! | 0 | Baseline |
| Male | 5 to 14 | 0 | #NUM! | 0 | Baseline |
| Male | 15 to 21 | 0 | #NUM! | 0 | Baseline |
| Female | 5 to 14 | 0 | #NUM! | 0 | Baseline |
| Male | 5 to 14 | 0 | #NUM! | 0 | Baseline |
| Female | 5 to 14 | 0 | #NUM! | 0 | Baseline |
| Female | 35+ | 0 | #NUM! | 0 | Baseline |
| Female | 22 to 35 | 0 | #NUM! | 0 | Baseline |
| Male | 5 to 14 | 0 | #NUM! | 0 | Baseline |
| Female | 15 to 21 | 0 | #NUM! | 0 | Baseline |
| Female | 22 to 35 | 0 | #NUM! | 0 | Baseline |
| Female | 35+ | 0 | #NUM! | 0 | Baseline |
| Female | 35+ | 0 | #NUM! | 0 | Baseline |
| Female | 35+ | 0 | #NUM! | 0 | Baseline |
| Female | 35+ | 0 | #NUM! | 0 | Baseline |
| Female | 35+ | 0 | #NUM! | 0 | Baseline |
| Female | 35+ | 0 | #NUM! | 0 | Baseline |

| Female | 35+ | 0 | #NUM! | 0 | Baseline |
| --- | --- | --- | --- | --- | --- |
| Female | 35+ | 0 | #NUM! | 0 | Baseline |
| Female | 35+ | 0 | #NUM! | 0 | Baseline |
| Female | 35+ | 0 | #NUM! | 0 | Baseline |
| Female | 35+ | 0 | #NUM! | 0 | Baseline |
| Female | 15 to 21 | 0 | #NUM! | 0 | Baseline |
| Female | 5 to 14 | 0 | #NUM! | 0 | Baseline |
| Female | 35+ | 0 | #NUM! | 0 | Baseline |
| Female | 35+ | 0 | #NUM! | 0 | Baseline |
| Male | 35+ | 0 | #NUM! | 0 | Baseline |
| Male | 5 to 14 | 0 | #NUM! | 0 | Baseline |
| Female | 22 to 35 | 0 | #NUM! | 0 | Baseline |
| Male | 35+ | 0 | #NUM! | 0 | Baseline |
| Male | 35+ | 0 | #NUM! | 0 | Baseline |
| Female | 5 to 14 | 0 | #NUM! | 0 | Baseline |
| Female | 35+ | 0 | #NUM! | 0 | Baseline |
| Male | 1 to 4 | 0 | #NUM! | 0 | Baseline |
| Female | 5 to 14 | 0 | #NUM! | 0 | Baseline |
| Male | 1 to 4 | 0 | #NUM! | 0 | Baseline |
| Male | 5 to 14 | 0 | #NUM! | 0 | Baseline |
| Female | 5 to 14 | 0 | #NUM! | 0 | Baseline |
| Male | 5 to 14 | 0 | #NUM! | 0 | Baseline |
| Female | 5 to 14 | 0 | #NUM! | 0 | Baseline |
| Female | 5 to 14 | 0 | #NUM! | 0 | Baseline |
| Female | 5 to 14 | 0 | #NUM! | 0 | Baseline |
| Male | 5 to 14 | 0 | #NUM! | 0 | Baseline |
| Male | 35+ | 0 | #NUM! | 0 | Baseline |
| Male | 5 to 14 | 0 | #NUM! | 0 | Baseline |
| Male | 35+ | 0 | #NUM! | 0 | Baseline |
| Female | 35+ | 0 | #NUM! | 0 | Baseline |
| Female | 22 to 35 | 0 | #NUM! | 0 | Baseline |
| Male | 5 to 14 | 0 | #NUM! | 0 | Baseline |
| Female | 5 to 14 | 0 | #NUM! | 0 | Baseline |
| Male | 1 to 4 | 0 | #NUM! | 0 | Baseline |
| Male | 5 to 14 | 0 | #NUM! | 0 | Baseline |
| Female | 5 to 14 | 0 | #NUM! | 0 | Baseline |
| Male | 5 to 14 | 0 | #NUM! | 0 | Baseline |
| Female | 5 to 14 | 0 | #NUM! | 0 | Baseline |
| Female | 1 to 4 | 0 | #NUM! | 0 | Baseline |
| Female | 5 to 14 | 0 | #NUM! | 0 | Baseline |
| Female | 22 to 35 | 0 | #NUM! | 0 | Baseline |
| Male | 5 to 14 | 0 | #NUM! | 0 | Baseline |
| Male | 35+ | 0 | #NUM! | 0 | Baseline |
| Female | 22 to 35 | 0 | #NUM! | 0 | Baseline |
| Female | 35+ | 0 | #NUM! | 0 | Baseline |
| Female | 35+ | 0 | #NUM! | 0 | Baseline |
| Female | 5 to 14 | 0 | #NUM! | 0 | Baseline |
| Male | 1 to 4 | 0 | #NUM! | 0 | Baseline |
| Female | 35+ | 0 | #NUM! | 0 | Baseline |
| Female | 5 to 14 | 0 | #NUM! | 0 | Baseline |

| Female | 15 to 21 | 0 | #NUM! | 0 | Baseline |
| --- | --- | --- | --- | --- | --- |
| Female | 15 to 21 | 0 | #NUM! | 0 | Baseline |
| Female | 5 to 14 | 0 | #NUM! | 0 | Baseline |
| Female | 22 to 35 | 0 | #NUM! | 0 | Baseline |
| Male | 1 to 4 | 0 | #NUM! | 0 | Baseline |
| Male | 1 to 4 | 0 | #NUM! | 0 | Baseline |
| Female | 15 to 21 | 0 | #NUM! | 0 | Baseline |
| Male | 1 to 4 | 0 | #NUM! | 0 | Baseline |
| Male | 15 to 21 | 0 | #NUM! | 0 | Baseline |
| Female | 15 to 21 | 0 | #NUM! | 0 | Baseline |
| Female | 15 to 21 | 0 | #NUM! | 0 | Baseline |
| Male | 15 to 21 | 0 | #NUM! | 0 | Baseline |
| Male | 1 to 4 | 0 | #NUM! | 0 | Baseline |
| Male | 15 to 21 | 0 | #NUM! | 0 | Baseline |
| Male | 1 to 4 | 0 | #NUM! | 0 | Baseline |
| Female | 15 to 21 | 0 | #NUM! | 0 | Baseline |
| Male | 1 to 4 | 0 | #NUM! | 0 | Baseline |
| Female | 15 to 21 | 0 | #NUM! | 0 | Baseline |
| Male | 1 to 4 | 0 | #NUM! | 0 | Baseline |
| Female | 15 to 21 | 0 | #NUM! | 0 | Baseline |
| Male | 1 to 4 | 0 | #NUM! | 0 | Baseline |
| Male | 15 to 21 | 0 | #NUM! | 0 | Baseline |
| Male | 1 to 4 | 0 | #NUM! | 0 | Baseline |
| Male | 1 to 4 | 0 | #NUM! | 0 | Baseline |
| Male | 1 to 4 | 0 | #NUM! | 0 | Baseline |
| Male | 1 to 4 | 0 | #NUM! | 0 | Baseline |
| Male | 15 to 21 | 0 | #NUM! | 0 | Baseline |
| Male | 15 to 21 | 0 | #NUM! | 0 | Baseline |
| Male | 5 to 14 | 0 | #NUM! | 0 | Baseline |
| Male | 15 to 21 | 0 | #NUM! | 0 | Baseline |
| Male | 5 to 14 | 0 | #NUM! | 0 | Baseline |
| Female | 5 to 14 | 0 | #NUM! | 0 | Baseline |
| Male | 5 to 14 | 0 | #NUM! | 0 | Baseline |
| Male | 15 to 21 | 0 | #NUM! | 0 | Baseline |
| Male | 15 to 21 | 0 | #NUM! | 0 | Baseline |
| Female | 15 to 21 | 0 | #NUM! | 0 | Baseline |
| Male | 5 to 14 | 0 | #NUM! | 0 | Baseline |
| Male | 15 to 21 | 0 | #NUM! | 0 | Baseline |
| Female | 15 to 21 | 0 | #NUM! | 0 | Baseline |
| Female | 22 to 35 | 0 | #NUM! | 0 | Baseline |
| Male | 5 to 14 | 0 | #NUM! | 0 | Baseline |
| Female | 35+ | 0 | #NUM! | 0 | Baseline |
| Female | 22 to 35 | 0 | #NUM! | 0 | Baseline |
| Male | 5 to 14 | 0 | #NUM! | 0 | Baseline |
| Female | 1 to 4 | 0 | #NUM! | 0 | Baseline |
| Female | 5 to 14 | 0 | #NUM! | 0 | Baseline |
| Male | 1 to 4 | 0 | #NUM! | 0 | Baseline |
| Female | 22 to 35 | 0 | #NUM! | 0 | Baseline |
| Male | 5 to 14 | 0 | #NUM! | 0 | Baseline |
| Female | 5 to 14 | 0 | #NUM! | 0 | Baseline |

| Male | 5 to 14 | 0 | #NUM! | 0 | Baseline |
| --- | --- | --- | --- | --- | --- |
| Male | 35+ | 0 | #NUM! | 0 | Baseline |
| Male | 35+ | 0 | #NUM! | 0 | Baseline |
| Female | 35+ | 0 | #NUM! | 0 | Baseline |
| Female | 1 to 4 | 0 | #NUM! | 0 | Baseline |
| Male | 35+ | 0 | #NUM! | 0 | Baseline |
| Female | 5 to 14 | 0 | #NUM! | 0 | Baseline |
| Male | 35+ | 0 | #NUM! | 0 | Baseline |
| Male | 5 to 14 | 0 | #NUM! | 0 | Baseline |
| Female | 5 to 14 | 0 | #NUM! | 0 | Baseline |
| Male | 35+ | 0 | #NUM! | 0 | Baseline |
| Male | 35+ | 0 | #NUM! | 0 | Baseline |
| Female | 35+ | 0 | #NUM! | 0 | Baseline |
| Female | 15 to 21 | 0 | #NUM! | 0 | Baseline |
| Male | 1 to 4 | 0 | #NUM! | 0 | Baseline |
| Male | 22 to 35 | 0 | #NUM! | 0 | Baseline |
| Female | 22 to 35 | 0 | #NUM! | 0 | Baseline |
| Female | 15 to 21 | 0 | #NUM! | 0 | Baseline |
| Male | 15 to 21 | 0 | #NUM! | 0 | Baseline |
| Female | 35+ | 0 | #NUM! | 0 | Baseline |
| Female | 1 to 4 | 0 | #NUM! | 0 | Baseline |
| Male | 22 to 35 | 0 | #NUM! | 0 | Baseline |
| Male | 1 to 4 | 0 | #NUM! | 0 | Baseline |
| Male | 1 to 4 | 0 | #NUM! | 0 | Baseline |
| Male | 15 to 21 | 0 | #NUM! | 0 | Baseline |
| Male | 1 to 4 | 0 | #NUM! | 0 | Baseline |
| Male | 1 to 4 | 0 | #NUM! | 0 | Baseline |
| Male | 5 to 14 | 0 | #NUM! | 0 | Baseline |
| Female | 35+ | 0 | #NUM! | 0 | Baseline |
| Male | 1 to 4 | 0 | #NUM! | 0 | Baseline |
| Male | 35+ | 0 | #NUM! | 0 | Baseline |
| Male | 1 to 4 | 0 | #NUM! | 0 | Baseline |
| Male | 35+ | 0 | #NUM! | 0 | Baseline |
| Male | 5 to 14 | 0 | #NUM! | 0 | Baseline |
| Male | 5 to 14 | 0 | #NUM! | 0 | Baseline |
| Male | 35+ | 0 | #NUM! | 0 | Baseline |
| Female | 35+ | 0 | #NUM! | 0 | Baseline |
| Female | 22 to 35 | 0 | #NUM! | 0 | Baseline |
| Female | 35+ | 0 | #NUM! | 0 | Baseline |
| Female | 35+ | 0 | #NUM! | 0 | Baseline |
| Male | 1 to 4 | 0 | #NUM! | 0 | Baseline |
| Male | 35+ | 0 | #NUM! | 0 | Baseline |
| Female | 35+ | 0 | #NUM! | 0 | Baseline |
| Male | 1 to 4 | 0 | #NUM! | 0 | Baseline |
| Male | 35+ | 0 | #NUM! | 0 | Baseline |
| Female | 22 to 35 | 0 | #NUM! | 0 | Baseline |
| Male | 35+ | 0 | #NUM! | 0 | Baseline |
| Male | 5 to 14 | 0 | #NUM! | 0 | Baseline |
| Male | 5 to 14 | 0 | #NUM! | 0 | Baseline |
| Male | 35+ | 0 | #NUM! | 0 | Baseline |

| Male | 5 to 14 | 0 | #NUM! | 0 | Baseline |
| --- | --- | --- | --- | --- | --- |
| Female | 35+ | 0 | #NUM! | 0 | Baseline |
| Male | 5 to 14 | 0 | #NUM! | 0 | Baseline |
| Male | 15 to 21 | 0 | #NUM! | 0 | Baseline |
| Male | 5 to 14 | 0 | #NUM! | 0 | Baseline |
| Female | 5 to 14 | 0 | #NUM! | 0 | Baseline |
| Male | 1 to 4 | 0 | #NUM! | 0 | Baseline |
| Male | 22 to 35 | 0 | #NUM! | 0 | Baseline |
| Female | 5 to 14 | 0 | #NUM! | 0 | Baseline |
| Male | 5 to 14 | 0 | #NUM! | 0 | Baseline |
| Male | 5 to 14 | 0 | #NUM! | 0 | Baseline |
| Male | 1 to 4 | 0 | #NUM! | 0 | Baseline |
| Male | 1 to 4 | 0 | #NUM! | 0 | Baseline |
| Female | 5 to 14 | 0 | #NUM! | 0 | Baseline |
| Male | 1 to 4 | 0 | #NUM! | 0 | Baseline |
| Male | 1 to 4 | 0 | #NUM! | 0 | Baseline |
| Male | 1 to 4 | 0 | #NUM! | 0 | Baseline |
| Female | 5 to 14 | 0 | #NUM! | 0 | Baseline |
| Male | 5 to 14 | 0 | #NUM! | 0 | Baseline |
| Female | 5 to 14 | 0 | #NUM! | 0 | Baseline |
| Male | 5 to 14 | 0 | #NUM! | 0 | Baseline |
| Female | 15 to 21 | 0 | #NUM! | 0 | Baseline |
| Male | 15 to 21 | 0 | #NUM! | 0 | Baseline |
| Male | 5 to 14 | 0 | #NUM! | 0 | Baseline |
| Male | 5 to 14 | 0 | #NUM! | 0 | Baseline |
| Male | 5 to 14 | 0 | #NUM! | 0 | Baseline |
| Male | 5 to 14 | 0 | #NUM! | 0 | Baseline |
| Male | 15 to 21 | 0 | #NUM! | 0 | Baseline |
| Male | 5 to 14 | 0 | #NUM! | 0 | Baseline |
| Male | 5 to 14 | 0 | #NUM! | 0 | Baseline |
| Male | 5 to 14 | 0 | #NUM! | 0 | Baseline |
| Male | 5 to 14 | 0 | #NUM! | 0 | Baseline |
| Male | 5 to 14 | 0 | #NUM! | 0 | Baseline |
| Male | 22 to 35 | 0 | #NUM! | 0 | Baseline |
| Male | 5 to 14 | 0 | #NUM! | 0 | Baseline |
| Female | 5 to 14 | 0 | #NUM! | 0 | Baseline |
| Male | 22 to 35 | 0 | #NUM! | 0 | Baseline |
| Female | 5 to 14 | 0 | #NUM! | 0 | Baseline |
| Female | 5 to 14 | 0 | #NUM! | 0 | Baseline |
| Male | 5 to 14 | 0 | #NUM! | 0 | Baseline |
| Female | 15 to 21 | 0 | #NUM! | 0 | Baseline |
| Female | 5 to 14 | 0 | #NUM! | 0 | Baseline |
| Female | 5 to 14 | 0 | #NUM! | 0 | Baseline |
| Male | 5 to 14 | 0 | #NUM! | 0 | Baseline |
| Female | 5 to 14 | 0 | #NUM! | 0 | Baseline |
| Female | 15 to 21 | 0 | #NUM! | 0 | Baseline |
| Male | 15 to 21 | 0 | #NUM! | 0 | Baseline |
| Male | 15 to 21 | 0 | #NUM! | 0 | Baseline |
| Male | 5 to 14 | 0 | #NUM! | 0 | Baseline |
| Male | 5 to 14 | 0 | #NUM! | 0 | Baseline |

| Female | 5 to 14 | 0 | #NUM! | 0 | Baseline |
| --- | --- | --- | --- | --- | --- |
| Male | 5 to 14 | 0 | #NUM! | 0 | Baseline |
| Female | 5 to 14 | 0 | #NUM! | 0 | Baseline |
| Female | 35+ | 0 | #NUM! | 0 | Baseline |
| Female | 22 to 35 | 0 | #NUM! | 0 | Baseline |
| Female | 35+ | 0 | #NUM! | 0 | Baseline |
| Male | 1 to 4 | 0 | #NUM! | 0 | Baseline |
| Male | 1 to 4 | 0 | #NUM! | 0 | Baseline |
| Male | 1 to 4 | 0 | #NUM! | 0 | Baseline |
| Male | 1 to 4 | 0 | #NUM! | 0 | Baseline |
| Male | 5 to 14 | 0 | #NUM! | 0 | Baseline |
| Male | 5 to 14 | 0 | #NUM! | 0 | Baseline |
| Male | 1 to 4 | 0 | #NUM! | 0 | Baseline |
| Male | 5 to 14 | 0 | #NUM! | 0 | Baseline |
| Female | 1 to 4 | 0 | #NUM! | 0 | Baseline |
| Male | 1 to 4 | 0 | #NUM! | 0 | Baseline |
| Male | 1 to 4 | 0 | #NUM! | 0 | Baseline |
| Male | 1 to 4 | 0 | #NUM! | 0 | Baseline |
| Male | 1 to 4 | 0 | #NUM! | 0 | Baseline |
| Male | 5 to 14 | 0 | #NUM! | 0 | Baseline |
| Male | 1 to 4 | 0 | #NUM! | 0 | Baseline |
| Female | 5 to 14 | 0 | #NUM! | 0 | Baseline |
| Male | 1 to 4 | 0 | #NUM! | 0 | Baseline |
| Male | 1 to 4 | 0 | #NUM! | 0 | Baseline |
| Male | 1 to 4 | 0 | #NUM! | 0 | Baseline |
| Male | 1 to 4 | 0 | #NUM! | 0 | Baseline |
| Male | 1 to 4 | 0 | #NUM! | 0 | Baseline |
| Female | 5 to 14 | 0 | #NUM! | 0 | Baseline |
| Male | 1 to 4 | 0 | #NUM! | 0 | Baseline |
| Male | 1 to 4 | 0 | #NUM! | 0 | Baseline |
| Female | 35+ | 0 | #NUM! | 0 | Baseline |
| Female | 5 to 14 | 0 | #NUM! | 0 | Baseline |
| Male | 1 to 4 | 0 | #NUM! | 0 | Baseline |
| Female | 35+ | 0 | #NUM! | 0 | Baseline |
| Male | 1 to 4 | 0 | #NUM! | 0 | Baseline |
| Male | 5 to 14 | 0 | #NUM! | 0 | Baseline |
| Male | 1 to 4 | 0 | #NUM! | 0 | Baseline |
| Male | 5 to 14 | 0 | #NUM! | 0 | Baseline |
| Male | 1 to 4 | 0 | #NUM! | 0 | Baseline |
| Female | 35+ | 0 | #NUM! | 0 | Baseline |
| Female | 5 to 14 | 0 | #NUM! | 0 | Baseline |
| Male | 1 to 4 | 0 | #NUM! | 0 | Baseline |
| Male | 1 to 4 | 0 | #NUM! | 0 | Baseline |
| Male | 1 to 4 | 0 | #NUM! | 0 | Baseline |
| Male | 1 to 4 | 0 | #NUM! | 0 | Baseline |
| Male | 1 to 4 | 0 | #NUM! | 0 | Baseline |
| Male | 5 to 14 | 0 | #NUM! | 0 | Baseline |
| Female | 22 to 35 | 1 | 0 | 0 | Baseline |
| Male | 5 to 14 | 0 | #NUM! | 0 | Baseline |
| Male | 5 to 14 | 0 | #NUM! | 0 | Baseline |

| Female | 22 to 35 | 2 | 0 | 0 | Baseline |
| --- | --- | --- | --- | --- | --- |
| Male | 5 to 14 | 0 | #NUM! | 0 | Baseline |
| Female | 22 to 35 | 5 | #NUM! | 0 | Baseline |
| Female | 22 to 35 | 0 | #NUM! | 0 | Baseline |
| Male | 5 to 14 | 0 | #NUM! | 0 | Baseline |
| Female | 5 to 14 | 0 | #NUM! | 0 | Baseline |
| Female | 22 to 35 | 0 | #NUM! | 0 | Baseline |
| Female | 22 to 35 | 0 | #NUM! | 0 | Baseline |
| Female | 22 to 35 | 0 | #NUM! | 0 | Baseline |
| Male | 5 to 14 | 0 | #NUM! | 0 | Baseline |
| Female | 35+ | 0 | #NUM! | 0 | Baseline |
| Female | 22 to 35 | 0 | #NUM! | 0 | Baseline |
| Male | 15 to 21 | 0 | #NUM! | 0 | Baseline |
| Female | 15 to 21 | 5 | #NUM! | 0 | Baseline |
| Male | 15 to 21 | 0 | #NUM! | 0 | Baseline |
| Male | 15 to 21 | 0 | #NUM! | 0 | Baseline |
| Male | 15 to 21 | 0 | #NUM! | 0 | Baseline |
| Male | 15 to 21 | 0 | #NUM! | 0 | Baseline |
| Female | 15 to 21 | 0 | #NUM! | 0 | Baseline |
| Male | 15 to 21 | 0 | #NUM! | 0 | Baseline |
| Male | 15 to 21 | 0 | #NUM! | 0 | Baseline |
| Female | 15 to 21 | 0 | #NUM! | 0 | Baseline |
| Male | 15 to 21 | 0 | #NUM! | 0 | Baseline |
| Male | 15 to 21 | 0 | #NUM! | 0 | Baseline |
| Female | 15 to 21 | 0 | #NUM! | 0 | Baseline |
| Female | 15 to 21 | 0 | #NUM! | 0 | Baseline |
| Male | 15 to 21 | 0 | #NUM! | 0 | Baseline |
| Female | 15 to 21 | 0 | #NUM! | 0 | Baseline |
| Female | 5 to 14 | 0 | #NUM! | 0 | Baseline |
| Female | 5 to 14 | 0 | #NUM! | 0 | Baseline |
| Male | 5 to 14 | 0 | #NUM! | 0 | Baseline |
| Male | 5 to 14 | 0 | #NUM! | 0 | Baseline |
| Female | 5 to 14 | 0 | #NUM! | 0 | Baseline |
| Female | 5 to 14 | 0 | #NUM! | 0 | Baseline |
| Female | 5 to 14 | 0 | #NUM! | 0 | Baseline |
| Male | 1 to 4 | 0 | #NUM! | 0 | Baseline |
| Male | 15 to 21 | 0 | #NUM! | 0 | Baseline |
| Female | 15 to 21 | 0 | #NUM! | 0 | Baseline |
| Male | 15 to 21 | 0 | #NUM! | 0 | Baseline |
| Male | 15 to 21 | 0 | #NUM! | 0 | Baseline |
| Female | 22 to 35 | 0 | #NUM! | 0 | Baseline |
| Female | 15 to 21 | 0 | #NUM! | 0 | Baseline |
| Female | 35+ | 0 | #NUM! | 0 | Baseline |
| Male | 5 to 14 | 0 | #NUM! | 0 | Baseline |
| Female | 5 to 14 | 0 | #NUM! | 0 | Baseline |
| Female | 35+ | 0 | #NUM! | 0 | Baseline |
| Female | 22 to 35 | 0 | #NUM! | 0 | Baseline |
| Female | 22 to 35 | 0 | #NUM! | 0 | Baseline |
| Male | 15 to 21 | 0 | #NUM! | 0 | Baseline |
| Female | 5 to 14 | 0 | #NUM! | 0 | Baseline |

| Male | 22 to 35 | 0 | #NUM! | 0 | Baseline |
| --- | --- | --- | --- | --- | --- |
| Female | 35+ | 0 | #NUM! | 0 | Baseline |
| Female | 22 to 35 | 0 | #NUM! | 0 | Baseline |
| Male | 5 to 14 | 0 | #NUM! | 0 | Baseline |
| Female | 22 to 35 | 0 | #NUM! | 0 | Baseline |
| Male | 5 to 14 | 0 | #NUM! | 0 | Baseline |
| Female | 5 to 14 | 0 | #NUM! | 0 | Baseline |
| Male | 1 to 4 | 0 | #NUM! | 0 | Baseline |
| Female | 22 to 35 | 0 | #NUM! | 0 | Baseline |
| Female | 1 to 4 | 0 | #NUM! | 0 | Baseline |
| Female | 22 to 35 | 0 | #NUM! | 0 | Baseline |
| Female | 5 to 14 | 0 | #NUM! | 0 | Baseline |
| Male | 22 to 35 | 0 | #NUM! | 0 | Baseline |
| Female | 15 to 21 | 0 | #NUM! | 0 | Baseline |
| Female | 15 to 21 | 0 | #NUM! | 0 | Baseline |
| Male | 15 to 21 | 0 | #NUM! | 0 | Baseline |
| Male | 15 to 21 | 0 | #NUM! | 0 | Baseline |
| Male | 15 to 21 | 0 | #NUM! | 0 | Baseline |
| Male | 5 to 14 | 0 | #NUM! | 0 | Baseline |
| Female | 15 to 21 | 0 | #NUM! | 0 | Baseline |
| Male | 5 to 14 | 0 | #NUM! | 0 | Baseline |
| Female | 15 to 21 | 0 | #NUM! | 0 | Baseline |
| Male | 5 to 14 | 0 | #NUM! | 0 | Baseline |
| Female | 5 to 14 | 0 | #NUM! | 0 | Baseline |
| Female | 15 to 21 | 0 | #NUM! | 0 | Baseline |
| Female | 15 to 21 | 0 | #NUM! | 0 | Baseline |
| Male | 15 to 21 | 0 | #NUM! | 0 | Baseline |
| Female | 5 to 14 | 0 | #NUM! | 0 | Baseline |
| Male | 5 to 14 | 0 | #NUM! | 0 | Baseline |
| Female | 15 to 21 | 0 | #NUM! | 0 | Baseline |
| Male | 5 to 14 | 0 | #NUM! | 0 | Baseline |
| Female | 15 to 21 | 0 | #NUM! | 0 | Baseline |
| Female | 5 to 14 | 0 | #NUM! | 0 | Baseline |
| Female | 5 to 14 | 0 | #NUM! | 0 | Baseline |
| Female | 15 to 21 | 5 | #NUM! | 0 | Baseline |
| Female | 5 to 14 | 0 | #NUM! | 0 | Baseline |
| Female | 5 to 14 | 0 | #NUM! | 0 | Baseline |
| Female | 5 to 14 | 0 | #NUM! | 0 | Baseline |
| Male | 5 to 14 | 0 | #NUM! | 0 | Baseline |
| Male | 5 to 14 | 0 | #NUM! | 0 | Baseline |
| Female | 15 to 21 | 0 | #NUM! | 0 | Baseline |
| Male | 15 to 21 | 0 | #NUM! | 0 | Baseline |
| Female | 5 to 14 | 0 | #NUM! | 0 | Baseline |
| Male | 1 to 4 | 0 | #NUM! | 0 | Baseline |
| Male | 5 to 14 | 0 | #NUM! | 0 | Baseline |
| Female | 5 to 14 | 0 | #NUM! | 0 | Baseline |
| Male | 5 to 14 | 0 | #NUM! | 0 | Baseline |
| Male | 5 to 14 | 0 | #NUM! | 0 | Baseline |
| Female | 22 to 35 | 0 | #NUM! | 0 | Baseline |
| Female | 22 to 35 | 0 | #NUM! | 0 | Baseline |

| Male | 5 to 14 | 0 | #NUM! | 0 | Baseline |
| --- | --- | --- | --- | --- | --- |
| Male | 15 to 21 | 0 | #NUM! | 0 | Baseline |
| Male | 5 to 14 | 0 | #NUM! | 0 | Baseline |
| Female | 35+ | 0 | #NUM! | 0 | Baseline |
| Male | 5 to 14 | 0 | #NUM! | 0 | Baseline |
| Female | 35+ | 0 | #NUM! | 0 | Baseline |
| Female | 22 to 35 | 0 | #NUM! | 0 | Baseline |
| Male | 35+ | 0 | #NUM! | 0 | Baseline |
| Male | 22 to 35 | 0 | #NUM! | 0 | Baseline |
| Male | 15 to 21 | 0 | #NUM! | 0 | Baseline |
| Female | 22 to 35 | 2 | #NUM! | 0 | Baseline |
| Female | 1 to 4 | 0 | #NUM! | 0 | Baseline |
| Female | 15 to 21 | 0 | #NUM! | 0 | Baseline |
| Female | 35+ | 0 | #NUM! | 0 | Baseline |
| Female | 5 to 14 | 0 | #NUM! | 0 | Baseline |
| Female | 1 to 4 | 0 | #NUM! | 0 | Baseline |
| Female | 15 to 21 | 0 | #NUM! | 0 | Baseline |
| Female | 15 to 21 | 0 | #NUM! | 0 | Baseline |
| Female | 35+ | 0 | #NUM! | 0 | Baseline |
| Male | 5 to 14 | 0 | #NUM! | 0 | Baseline |
| Female | 35+ | 0 | #NUM! | 0 | Baseline |
| Female | 22 to 35 | 0 | #NUM! | 0 | Baseline |
| Male | 22 to 35 | 0 | #NUM! | 0 | Baseline |
| Female | 35+ | 0 | #NUM! | 0 | Baseline |
| Female | 35+ | 0 | #NUM! | 0 | Baseline |
| Female | 22 to 35 | 0 | #NUM! | 0 | Baseline |
| Male | 1 to 4 | 0 | #NUM! | 0 | Baseline |
| Male | 15 to 21 | 0 | #NUM! | 0 | Baseline |
| Female | 15 to 21 | 0 | #NUM! | 0 | Baseline |
| Male | 15 to 21 | 0 | #NUM! | 0 | Baseline |
| Female | 35+ | 0 | #NUM! | 0 | Baseline |
| Female | 5 to 14 | 0 | #NUM! | 0 | Baseline |
| Male | 5 to 14 | 0 | #NUM! | 0 | Baseline |
| Female | 15 to 21 | 0 | #NUM! | 0 | Baseline |
| Male | 5 to 14 | 0 | #NUM! | 0 | Baseline |
| Female | 5 to 14 | 0 | #NUM! | 0 | Baseline |
| Female | 35+ | 0 | #NUM! | 0 | Baseline |
| Male | 35+ | 0 | #NUM! | 0 | Baseline |
| Male | 5 to 14 | 0 | #NUM! | 0 | Baseline |
| Female | 35+ | 0 | #NUM! | 0 | Baseline |
| Male | 5 to 14 | 0 | #NUM! | 0 | Baseline |
| Female | 35+ | 0 | #NUM! | 0 | Baseline |
| Female | 22 to 35 | 0 | #NUM! | 0 | Baseline |
| Male | 5 to 14 | 0 | #NUM! | 0 | Baseline |
| Female | 5 to 14 | 0 | #NUM! | 0 | Baseline |
| Female | 15 to 21 | 0 | #NUM! | 0 | Baseline |
| Female | 35+ | 0 | #NUM! | 0 | Baseline |
| Male | 35+ | 0 | #NUM! | 0 | Baseline |
| Female | 35+ | 0 | #NUM! | 0 | Baseline |
| Female | 22 to 35 | 0 | #NUM! | 0 | Baseline |

| Male | 5 to 14 | 0 | #NUM! | 0 | Baseline |
| --- | --- | --- | --- | --- | --- |
| Female | 22 to 35 | 0 | #NUM! | 0 | Baseline |
| Female | 5 to 14 | 0 | #NUM! | 0 | Baseline |
| Male | 1 to 4 | 0 | #NUM! | 0 | Baseline |
| Female | 5 to 14 | 0 | #NUM! | 0 | Baseline |
| Female | 5 to 14 | 0 | #NUM! | 0 | Baseline |
| Male | 5 to 14 | 0 | #NUM! | 0 | Baseline |
| Male | 5 to 14 | 0 | #NUM! | 0 | Baseline |
| Male | 5 to 14 | 0 | #NUM! | 0 | Baseline |
| Male | 5 to 14 | 0 | #NUM! | 0 | Baseline |
| Male | 5 to 14 | 0 | #NUM! | 0 | Baseline |
| Male | 1 to 4 | 0 | #NUM! | 0 | Baseline |
| Male | 5 to 14 | 0 | #NUM! | 0 | Baseline |
| Male | 5 to 14 | 0 | #NUM! | 0 | Baseline |
| Male | 5 to 14 | 0 | #NUM! | 0 | Baseline |
| Male | 5 to 14 | 0 | #NUM! | 0 | Baseline |
| Female | 35+ | 0 | #NUM! | 0 | Baseline |
| Male | 35+ | 0 | #NUM! | 0 | Baseline |
| Female | 35+ | 0 | #NUM! | 0 | Baseline |
| Male | 1 to 4 | 0 | #NUM! | 0 | Baseline |
| Male | 5 to 14 | 0 | #NUM! | 0 | Baseline |
| Male | 35+ | 0 | #NUM! | 0 | Baseline |
| Female | 35+ | 0 | #NUM! | 0 | Baseline |
| Male | 5 to 14 | 0 | #NUM! | 0 | Baseline |
| Female | 35+ | 0 | #NUM! | 0 | Baseline |
| Female | 35+ | 0 | #NUM! | 0 | Baseline |
| Male | 15 to 21 | 0 | #NUM! | 0 | Baseline |
| Male | 22 to 35 | 0 | #NUM! | 0 | Baseline |
| Female | 15 to 21 | 0 | #NUM! | 0 | Baseline |
| Female | 5 to 14 | 0 | #NUM! | 0 | Baseline |
| Female | 15 to 21 | 0 | #NUM! | 0 | Baseline |
| Male | 1 to 4 | 0 | #NUM! | 0 | Baseline |
| Male | 5 to 14 | 0 | #NUM! | 0 | Baseline |
| Female | 5 to 14 | 0 | #NUM! | 0 | Baseline |
| Male | 5 to 14 | 0 | #NUM! | 0 | Baseline |
| Female | 35+ | 0 | #NUM! | 0 | Baseline |
| Female | 5 to 14 | 0 | #NUM! | 0 | Baseline |
| Female | 22 to 35 | 0 | #NUM! | 0 | Baseline |
| Male | 5 to 14 | 0 | #NUM! | 0 | Baseline |
| Female | 15 to 21 | 0 | #NUM! | 0 | Baseline |
| Female | 5 to 14 | 0 | #NUM! | 0 | Baseline |
| Male | 5 to 14 | 0 | #NUM! | 0 | Baseline |
| Female | 5 to 14 | 0 | #NUM! | 0 | Baseline |
| Female | 15 to 21 | 0 | #NUM! | 0 | Baseline |
| Female | 15 to 21 | 0 | #NUM! | 0 | Baseline |
| Female | 22 to 35 | 0 | #NUM! | 0 | Baseline |
| Female | 15 to 21 | 0 | #NUM! | 0 | Baseline |
| Female | 15 to 21 | 0 | #NUM! | 0 | Baseline |
| Male | 5 to 14 | 0 | #NUM! | 0 | Baseline |
| Female | 15 to 21 | 0 | #NUM! | 0 | Baseline |

| Female | 22 to 35 | 0 | #NUM! | 0 | Baseline |
| --- | --- | --- | --- | --- | --- |
| Female | 5 to 14 | 0 | #NUM! | 0 | Baseline |
| Female | 5 to 14 | 0 | #NUM! | 0 | Baseline |
| Male | 15 to 21 | 0 | #NUM! | 0 | Baseline |
| Male | 15 to 21 | 0 | #NUM! | 0 | Baseline |
| Female | 22 to 35 | 0 | #NUM! | 0 | Baseline |
| Male | 5 to 14 | 0 | #NUM! | 0 | Baseline |
| Male | 35+ | 0 | #NUM! | 0 | Baseline |
| Female | 35+ | 0 | #NUM! | 0 | Baseline |
| Male | 5 to 14 | 0 | #NUM! | 0 | Baseline |
| Female | 35+ | 0 | #NUM! | 0 | Baseline |
| Female | 5 to 14 | 0 | #NUM! | 0 | Baseline |
| Male | 5 to 14 | 0 | #NUM! | 0 | Baseline |
| Female | 5 to 14 | 0 | #NUM! | 0 | Baseline |
| Male | 1 to 4 | 0 | #NUM! | 0 | Baseline |
| Male | 5 to 14 | 0 | #NUM! | 0 | Baseline |
| Male | 5 to 14 | 0 | #NUM! | 0 | Baseline |
| Female | 22 to 35 | 0 | #NUM! | 0 | Baseline |
| Female | 22 to 35 | 0 | #NUM! | 0 | Baseline |
| Female | 35+ | 0 | #NUM! | 0 | Baseline |
| Male | 35+ | 0 | #NUM! | 0 | Baseline |
| Female | 35+ | 0 | #NUM! | 0 | Baseline |
| Male | 5 to 14 | 0 | #NUM! | 0 | Baseline |
| Male | 1 to 4 | 0 | #NUM! | 0 | Baseline |
| Male | 35+ | 0 | #NUM! | 0 | Baseline |
| Female | 5 to 14 | 0 | #NUM! | 0 | Baseline |
| Male | 5 to 14 | 0 | #NUM! | 0 | Baseline |
| Male | 5 to 14 | 0 | #NUM! | 0 | Baseline |
| Female | 5 to 14 | 0 | #NUM! | 0 | Baseline |
| Male | 15 to 21 | 0 | #NUM! | 0 | Baseline |
| Female | 5 to 14 | 0 | #NUM! | 0 | Baseline |
| Male | 5 to 14 | 0 | #NUM! | 0 | Baseline |
| Female | 5 to 14 | 0 | #NUM! | 0 | Baseline |
| Female | 22 to 35 | 0 | #NUM! | 0 | Baseline |
| Male | 15 to 21 | 0 | #NUM! | 0 | Baseline |
| Female | 5 to 14 | 0 | #NUM! | 0 | Baseline |
| Male | 5 to 14 | 0 | #NUM! | 0 | Baseline |
| Male | 1 to 4 | 0 | #NUM! | 0 | Baseline |
| Male | 1 to 4 | 0 | #NUM! | 0 | Baseline |
| Male | 5 to 14 | 0 | #NUM! | 0 | Baseline |
| Male | 1 to 4 | 0 | #NUM! | 0 | Baseline |
| Female | 5 to 14 | 0 | #NUM! | 0 | Baseline |
| Male | 15 to 21 | 0 | #NUM! | 0 | Baseline |
| Female | 5 to 14 | 0 | #NUM! | 0 | Baseline |
| Male | 22 to 35 | 0 | #NUM! | 0 | Baseline |
| Female | 5 to 14 | 0 | #NUM! | 0 | Baseline |
| Male | 5 to 14 | 0 | #NUM! | 0 | Baseline |
| Male | 1 to 4 | 0 | #NUM! | 0 | Baseline |
| Male | 15 to 21 | 0 | #NUM! | 0 | Baseline |
| Male | 1 to 4 | 0 | #NUM! | 0 | Baseline |

| Female | 5 to 14 | 0 | #NUM! | 0 | Baseline |
| --- | --- | --- | --- | --- | --- |
| Male | 5 to 14 | 0 | #NUM! | 0 | Baseline |
| Male | 1 to 4 | 0 | #NUM! | 0 | Baseline |
| Male | 5 to 14 | 0 | #NUM! | 0 | Baseline |
| Female | 15 to 21 | 0 | #NUM! | 0 | Baseline |
| Male | 1 to 4 | 0 | #NUM! | 0 | Baseline |
| Male | 1 to 4 | 0 | #NUM! | 0 | Baseline |
| Male | 15 to 21 | 0 | #NUM! | 0 | Baseline |
| Male | 1 to 4 | 0 | #NUM! | 0 | Baseline |
| Female | 15 to 21 | 0 | #NUM! | 0 | Baseline |
| Male | 1 to 4 | 0 | #NUM! | 0 | Baseline |
| Female | 15 to 21 | 0 | #NUM! | 0 | Baseline |
| Male | 1 to 4 | 0 | #NUM! | 0 | Baseline |
| Female | 22 to 35 | 0 | #NUM! | 0 | Baseline |
| Female | 35+ | 0 | #NUM! | 0 | Baseline |
| Female | 22 to 35 | 0 | #NUM! | 0 | Baseline |
| Male | 15 to 21 | 0 | #NUM! | 0 | Baseline |
| Male | 5 to 14 | 0 | #NUM! | 0 | Baseline |
| Female | 22 to 35 | 0 | #NUM! | 0 | Baseline |
| Male | 1 to 4 | 0 | #NUM! | 0 | Baseline |
| Male | 1 to 4 | 0 | #NUM! | 0 | Baseline |
| Male | 1 to 4 | 0 | #NUM! | 0 | Baseline |
| Male | 5 to 14 | 0 | #NUM! | 0 | Baseline |
| Male | 5 to 14 | 0 | #NUM! | 0 | Baseline |
| Male | 5 to 14 | 0 | #NUM! | 0 | Baseline |
| Female | 22 to 35 | 0 | #NUM! | 0 | Baseline |
| Female | 5 to 14 | 0 | #NUM! | 0 | Baseline |
| Female | 35+ | 0 | #NUM! | 0 | Baseline |
| Female | 35+ | 0 | #NUM! | 0 | Baseline |
| Female | 5 to 14 | 0 | #NUM! | 0 | Baseline |
| Female | 15 to 21 | 0 | #NUM! | 0 | Baseline |
| Female | 35+ | 0 | #NUM! | 0 | Baseline |
| Male | 1 to 4 | 0 | #NUM! | 0 | Baseline |
| Male | 5 to 14 | 0 | #NUM! | 0 | Baseline |
| Male | 5 to 14 | 0 | #NUM! | 0 | Baseline |
| Female | 22 to 35 | 0 | #NUM! | 0 | Baseline |
| Male | 15 to 21 | 0 | #NUM! | 0 | Baseline |
| Female | 35+ | 0 | #NUM! | 0 | Baseline |
| Male | 5 to 14 | 0 | #NUM! | 0 | Baseline |
| Male | 15 to 21 | 0 | #NUM! | 0 | Baseline |
| Female | 22 to 35 | 0 | #NUM! | 0 | Baseline |
| Female | 5 to 14 | 0 | #NUM! | 0 | Baseline |
| Female | 5 to 14 | 0 | #NUM! | 0 | Baseline |
| Male | 1 to 4 | 0 | #NUM! | 0 | Baseline |
| Male | 22 to 35 | 0 | #NUM! | 0 | Baseline |
| Male | 22 to 35 | 0 | #NUM! | 0 | Baseline |
| Male | 1 to 4 | 0 | #NUM! | 0 | Baseline |
| Female | 5 to 14 | 0 | #NUM! | 0 | Baseline |
| Male | 1 to 4 | 0 | #NUM! | 0 | Baseline |
| Male | 22 to 35 | 0 | #NUM! | 0 | Baseline |

| Male | 1 to 4 | 0 | #NUM! | 0 | Baseline |
| --- | --- | --- | --- | --- | --- |
| Male | 1 to 4 | 0 | #NUM! | 0 | Baseline |
| Male | 1 to 4 | 0 | #NUM! | 0 | Baseline |
| Male | 5 to 14 | 0 | #NUM! | 0 | Baseline |
| Male | 1 to 4 | 0 | #NUM! | 0 | Baseline |
| Female | 5 to 14 | 0 | #NUM! | 0 | Baseline |
| Female | 5 to 14 | 0 | #NUM! | 0 | Baseline |
| Male | 5 to 14 | 0 | #NUM! | 0 | Baseline |
| Male | 5 to 14 | 0 | #NUM! | 0 | Baseline |
| Male | 1 to 4 | 0 | #NUM! | 0 | Baseline |
| Female | 22 to 35 | 0 | #NUM! | 0 | Baseline |
| Male | 1 to 4 | 0 | #NUM! | 0 | Baseline |
| Male | 1 to 4 | 0 | #NUM! | 0 | Baseline |
| Male | 35+ | 0 | #NUM! | 0 | Baseline |
| Male | 22 to 35 | 0 | #NUM! | 0 | Baseline |
| Male | 1 to 4 | 0 | #NUM! | 0 | Baseline |
| Male | 35+ | 0 | #NUM! | 0 | Baseline |
| Female | 35+ | 0 | #NUM! | 0 | Baseline |
| Female | 35+ | 0 | #NUM! | 0 | Baseline |
| Female | 22 to 35 | 0 | #NUM! | 0 | Baseline |
| Female | 15 to 21 | 0 | #NUM! | 0 | Baseline |
| Male | 22 to 35 | 0 | #NUM! | 0 | Baseline |
| Female | 35+ | 0 | #NUM! | 0 | Baseline |
| Male | 5 to 14 | 0 | #NUM! | 0 | Baseline |
| Female | 35+ | 0 | #NUM! | 0 | Baseline |
| Female | 22 to 35 | 5 | #NUM! | 0 | Baseline |
| Male | 5 to 14 | 0 | #NUM! | 0 | Baseline |
| Female | 15 to 21 | 0 | #NUM! | 0 | Baseline |
| Female | 22 to 35 | 5 | #NUM! | 0 | Baseline |
| Female | 22 to 35 | 0 | #NUM! | 0 | Baseline |
| Male | 15 to 21 | 0 | #NUM! | 0 | Baseline |
| Male | 35+ | 0 | #NUM! | 0 | Baseline |
| Female | 35+ | 3 | #NUM! | 0 | Baseline |
| Female | 5 to 14 | 0 | #NUM! | 0 | Baseline |
| Female | 22 to 35 | 0 | #NUM! | 0 | Baseline |
| Female | 22 to 35 | 0 | #NUM! | 0 | Baseline |
| Female | 5 to 14 | 0 | #NUM! | 0 | Baseline |
| Male | 15 to 21 | 0 | #NUM! | 0 | Baseline |
| Female | 5 to 14 | 0 | #NUM! | 0 | Baseline |
| Male | 35+ | 0 | #NUM! | 0 | Baseline |
| Male | 15 to 21 | 0 | #NUM! | 0 | Baseline |
| Male | 15 to 21 | 0 | #NUM! | 0 | Baseline |
| Male | 15 to 21 | 0 | #NUM! | 0 | Baseline |
| Male | 15 to 21 | 0 | #NUM! | 0 | Baseline |
| Female | 15 to 21 | 0 | #NUM! | 0 | Baseline |
| Male | 15 to 21 | 0 | #NUM! | 0 | Baseline |
| Male | 15 to 21 | 0 | #NUM! | 0 | Baseline |
| Female | 5 to 14 | 0 | #NUM! | 0 | Baseline |
| Female | 5 to 14 | 0 | #NUM! | 0 | Baseline |
| Female | 15 to 21 | 0 | #NUM! | 0 | Baseline |

| Female | 22 to 35 | 0 | #NUM! | 0 | Baseline |
| --- | --- | --- | --- | --- | --- |
| Female | 5 to 14 | 0 | #NUM! | 0 | Baseline |
| Male | 22 to 35 | 0 | #NUM! | 0 | Baseline |
| Male | 22 to 35 | 0 | #NUM! | 0 | Baseline |
| Male | 15 to 21 | 0 | #NUM! | 0 | Baseline |
| Male | 5 to 14 | 0 | #NUM! | 0 | Baseline |
| Female | 5 to 14 | 0 | #NUM! | 0 | Baseline |
| Female | 22 to 35 | 0 | #NUM! | 0 | Baseline |
| Female | 35+ | 0 | #NUM! | 0 | Baseline |
| Female | 15 to 21 | 0 | #NUM! | 0 | Baseline |
| Male | 5 to 14 | 0 | #NUM! | 0 | Baseline |
| Male | 35+ | 0 | #NUM! | 0 | Baseline |
| Female | 15 to 21 | 0 | #NUM! | 0 | Baseline |
| Male | 5 to 14 | 0 | #NUM! | 0 | Baseline |
| Female | 15 to 21 | 0 | #NUM! | 0 | Baseline |
| Female | 15 to 21 | 0 | #NUM! | 0 | Baseline |
| Female | 15 to 21 | 0 | #NUM! | 0 | Baseline |
| Male | 22 to 35 | 0 | #NUM! | 0 | Baseline |
| Female | 22 to 35 | 0 | #NUM! | 0 | Baseline |
| Female | 15 to 21 | 0 | #NUM! | 0 | Baseline |
| Male | 22 to 35 | 0 | #NUM! | 0 | Baseline |
| Female | 15 to 21 | 0 | #NUM! | 0 | Baseline |
| Male | 1 to 4 | 0 | #NUM! | 0 | Baseline |
| Male | 22 to 35 | 0 | #NUM! | 0 | Baseline |
| Female | 22 to 35 | 0 | #NUM! | 0 | Baseline |
| Male | 35+ | 0 | #NUM! | 0 | Baseline |
| Female | 22 to 35 | 0 | #NUM! | 0 | Baseline |
| Female | 22 to 35 | 0 | #NUM! | 0 | Baseline |
| Male | 5 to 14 | 0 | #NUM! | 0 | Baseline |
| Male | 5 to 14 | 0 | #NUM! | 0 | Baseline |
| Male | 22 to 35 | 0 | #NUM! | 0 | Baseline |
| Female | 22 to 35 | 0 | #NUM! | 0 | Baseline |
| Male | 1 to 4 | 0 | #NUM! | 0 | Baseline |
| Male | 5 to 14 | 0 | #NUM! | 0 | Baseline |
| Male | 22 to 35 | 0 | #NUM! | 0 | Baseline |
| Male | 15 to 21 | 0 | #NUM! | 0 | Baseline |
| Female | 15 to 21 | 0 | #NUM! | 0 | Baseline |
| Male | 1 to 4 | 0 | #NUM! | 0 | Baseline |
| Female | 22 to 35 | 0 | #NUM! | 0 | Baseline |
| Male | 15 to 21 | 0 | #NUM! | 0 | Baseline |
| Female | 22 to 35 | 0 | #NUM! | 0 | Baseline |
| Female | 22 to 35 | 0 | #NUM! | 0 | Baseline |
| Male | 15 to 21 | 0 | #NUM! | 0 | Baseline |
| Female | 22 to 35 | 0 | #NUM! | 0 | Baseline |
| Male | 1 to 4 | 0 | #NUM! | 0 | Baseline |
| Male | 1 to 4 | 0 | #NUM! | 0 | Baseline |
| Female | 5 to 14 | 0 | #NUM! | 0 | Baseline |
| Male | 1 to 4 | 0 | #NUM! | 0 | Baseline |
| Male | 1 to 4 | 0 | #NUM! | 0 | Baseline |
| Female | 22 to 35 | 0 | #NUM! | 0 | Baseline |

| Female | 15 to 21 | 0 | #NUM! | 0 | Baseline |
| --- | --- | --- | --- | --- | --- |
| Female | 22 to 35 | 0 | #NUM! | 0 | Baseline |
| Female | 15 to 21 | 0 | #NUM! | 0 | Baseline |
| Male | 1 to 4 | 0 | #NUM! | 0 | Baseline |
| Female | 22 to 35 | 0 | #NUM! | 0 | Baseline |
| Female | 22 to 35 | 0 | #NUM! | 0 | Baseline |
| Male | 1 to 4 | 0 | #NUM! | 0 | Baseline |
| Male | 22 to 35 | 0 | #NUM! | 0 | Baseline |
| Female | 15 to 21 | 0 | #NUM! | 0 | Baseline |
| Male | 35+ | 0 | #NUM! | 0 | Baseline |
| Female | 22 to 35 | 0 | #NUM! | 0 | Baseline |
| Male | 1 to 4 | 0 | #NUM! | 0 | Baseline |
| Male | 5 to 14 | 0 | #NUM! | 0 | Baseline |
| Female | 35+ | 0 | #NUM! | 0 | Baseline |
| Female | 5 to 14 | 1 | #NUM! | 0 | Baseline |
| Female | 5 to 14 | 0 | #NUM! | 0 | Baseline |
| Male | 5 to 14 | 0 | #NUM! | 0 | Baseline |
| Male | 5 to 14 | 1 | #NUM! | 0 | Baseline |
| Male | 5 to 14 | 0 | #NUM! | 0 | Baseline |
| Male | 1 to 4 | 0 | #NUM! | 0 | Baseline |
| Male | 1 to 4 | 0 | #NUM! | 0 | Baseline |
| Female | 15 to 21 | 0 | #NUM! | 0 | Baseline |
| Male | 1 to 4 | 0 | #NUM! | 0 | Baseline |
| Female | 15 to 21 | 0 | #NUM! | 0 | Baseline |
| Male | 15 to 21 | 0 | #NUM! | 0 | Baseline |
| Male | 15 to 21 | 0 | #NUM! | 0 | Baseline |
| Male | 15 to 21 | 0 | #NUM! | 0 | Baseline |
| Male | 15 to 21 | 0 | #NUM! | 0 | Baseline |
| Female | 5 to 14 | 0 | #NUM! | 0 | Baseline |
| Male | 1 to 4 | 0 | #NUM! | 0 | Baseline |
| Male | 1 to 4 | 0 | #NUM! | 0 | Baseline |
| Female | 5 to 14 | 0 | #NUM! | 0 | Baseline |
| Female | 5 to 14 | 0 | #NUM! | 0 | Baseline |
| Female | 5 to 14 | 0 | #NUM! | 0 | Baseline |
| Female | 5 to 14 | 0 | #NUM! | 0 | Baseline |
| Male | 5 to 14 | 0 | #NUM! | 0 | Baseline |
| Female | 5 to 14 | 0 | #NUM! | 0 | Baseline |
| Male | 5 to 14 | 0 | #NUM! | 0 | Baseline |
| Male | 5 to 14 | 0 | #NUM! | 0 | Baseline |
| Male | 1 to 4 | 0 | #NUM! | 0 | Baseline |
| Male | 5 to 14 | 0 | #NUM! | 0 | Baseline |
| Female | 1 to 4 | 0 | #NUM! | 0 | Baseline |
| Female | 1 to 4 | 0 | #NUM! | 0 | Baseline |
| Female | 15 to 21 | 0 | #NUM! | 0 | Baseline |
| Male | 1 to 4 | 0 | #NUM! | 0 | Baseline |
| Male | 1 to 4 | 0 | #NUM! | 0 | Baseline |
| Male | 1 to 4 | 0 | #NUM! | 0 | Baseline |
| Male | 1 to 4 | 0 | #NUM! | 0 | Baseline |
| Male | 1 to 4 | 0 | #NUM! | 0 | Baseline |
| Male | 22 to 35 | 0 | #NUM! | 0 | Baseline |

| Female | 35+ | 0 | #NUM! | 0 | Baseline |
| --- | --- | --- | --- | --- | --- |
| Male | 1 to 4 | 0 | #NUM! | 0 | Baseline |
| Male | 35+ | 0 | #NUM! | 0 | Baseline |
| Female | 22 to 35 | 0 | #NUM! | 0 | Baseline |
| Female | 35+ | 0 | #NUM! | 0 | Baseline |
| Female | 22 to 35 | 0 | #NUM! | 0 | Baseline |
| Male | 22 to 35 | 0 | #NUM! | 0 | Baseline |
| Female | 22 to 35 | 0 | #NUM! | 0 | Baseline |
| Male | 22 to 35 | 0 | #NUM! | 0 | Baseline |
| Male | 1 to 4 | 0 | #NUM! | 0 | Baseline |
| Female | 35+ | 0 | #NUM! | 0 | Baseline |
| Female | 22 to 35 | 0 | #NUM! | 0 | Baseline |
| Female | 35+ | 0 | #NUM! | 0 | Baseline |
| Female | 35+ | 0 | #NUM! | 0 | Baseline |
| Female | 22 to 35 | 0 | #NUM! | 0 | Baseline |
| Female | 22 to 35 | 0 | #NUM! | 0 | Baseline |
| Female | 22 to 35 | 0 | #NUM! | 0 | Baseline |
| Male | 35+ | 0 | #NUM! | 0 | Baseline |
| Male | 35+ | 0 | #NUM! | 0 | Baseline |
| Male | 22 to 35 | 0 | #NUM! | 0 | Baseline |
| Male | 22 to 35 | 0 | #NUM! | 0 | Baseline |
| Male | 35+ | 0 | #NUM! | 0 | Baseline |
| Male | 22 to 35 | 0 | #NUM! | 0 | Baseline |
| Male | 22 to 35 | 0 | #NUM! | 0 | Baseline |
| Male | 22 to 35 | 0 | #NUM! | 0 | Baseline |
| Male | 1 to 4 | 0 | #NUM! | 0 | Baseline |
| Male | 22 to 35 | 0 | #NUM! | 0 | Baseline |
| Male | 35+ | 0 | #NUM! | 0 | Baseline |
| Female | 15 to 21 | 0 | #NUM! | 0 | Baseline |
| Female | 15 to 21 | 0 | #NUM! | 0 | Baseline |
| Male | 15 to 21 | 0 | #NUM! | 0 | Baseline |
| Female | 1 to 4 | 0 | #NUM! | 0 | Baseline |
| Male | 1 to 4 | 0 | #NUM! | 0 | Baseline |
| Male | 1 to 4 | 0 | #NUM! | 0 | Baseline |
| Male | 35+ | 0 | #NUM! | 0 | Baseline |
| Female | 35+ | 0 | #NUM! | 0 | Baseline |
| Male | 1 to 4 | 0 | #NUM! | 0 | Baseline |
| Male | 15 to 21 | 0 | #NUM! | 0 | Baseline |
| Female | 35+ | 0 | #NUM! | 0 | Baseline |
| Male | 35+ | 0 | #NUM! | 0 | Baseline |
| Female | 22 to 35 | 0 | #NUM! | 0 | Baseline |
| Male | 35+ | 0 | #NUM! | 0 | Baseline |
| Female | 35+ | 0 | #NUM! | 0 | Baseline |
| Female | 22 to 35 | 0 | #NUM! | 0 | Baseline |
| Female | 35+ | 0 | #NUM! | 0 | Baseline |
| Male | 22 to 35 | 0 | #NUM! | 0 | Baseline |
| Female | 22 to 35 | 0 | #NUM! | 0 | Baseline |
| Female | 22 to 35 | 0 | #NUM! | 0 | Baseline |
| Male | 22 to 35 | 0 | #NUM! | 0 | Baseline |
| Male | 35+ | 0 | #NUM! | 0 | Baseline |

| Male | 35+ | 0 | #NUM! | 0 | Baseline |
| --- | --- | --- | --- | --- | --- |
| Female | 35+ | 0 | #NUM! | 0 | Baseline |
| Female | 22 to 35 | 0 | #NUM! | 0 | Baseline |
| Male | 22 to 35 | 0 | #NUM! | 0 | Baseline |
| Male | 22 to 35 | 0 | #NUM! | 0 | Baseline |
| Male | 35+ | 0 | #NUM! | 0 | Baseline |
| Male | 35+ | 0 | #NUM! | 0 | Baseline |
| Female | 22 to 35 | 0 | #NUM! | 0 | Baseline |
| Male | 1 to 4 | 0 | #NUM! | 0 | Baseline |
| Male | 1 to 4 | 0 | #NUM! | 0 | Baseline |
| Female | 35+ | 0 | #NUM! | 0 | Baseline |
| Male | 5 to 14 | 0 | #NUM! | 0 | Baseline |
| Female | 5 to 14 | 0 | #NUM! | 0 | Baseline |
| Female | 5 to 14 | 0 | #NUM! | 0 | Baseline |
| Female | 15 to 21 | 0 | #NUM! | 0 | Baseline |
| Female | 1 to 4 | 0 | #NUM! | 0 | Baseline |
| Female | 5 to 14 | 0 | #NUM! | 0 | Baseline |
| Male | 5 to 14 | 0 | #NUM! | 0 | Baseline |
| Male | 5 to 14 | 0 | #NUM! | 0 | Baseline |
| Male | 5 to 14 | 0 | #NUM! | 0 | Baseline |
| Female | 5 to 14 | 0 | #NUM! | 0 | Baseline |
| Female | 5 to 14 | 0 | #NUM! | 0 | Baseline |
| Male | 5 to 14 | 0 | #NUM! | 0 | Baseline |
| Male | 5 to 14 | 0 | #NUM! | 0 | Baseline |
| Male | 5 to 14 | 0 | #NUM! | 0 | Baseline |
| Male | 15 to 21 | 0 | #NUM! | 0 | Baseline |
| Male | 5 to 14 | 0 | #NUM! | 0 | Baseline |
| Male | 5 to 14 | 0 | #NUM! | 0 | Baseline |
| Male | 5 to 14 | 0 | #NUM! | 0 | Baseline |
| Male | 15 to 21 | 0 | #NUM! | 0 | Baseline |
| Male | 5 to 14 | 0 | #NUM! | 0 | Baseline |
| Female | 15 to 21 | 0 | #NUM! | 0 | Baseline |
| Male | 15 to 21 | 0 | #NUM! | 0 | Baseline |
| Male | 15 to 21 | 0 | #NUM! | 0 | Baseline |
| Male | 15 to 21 | 0 | #NUM! | 0 | Baseline |
| Male | 5 to 14 | 0 | #NUM! | 0 | Baseline |
| Female | 1 to 4 | 0 | #NUM! | 0 | Baseline |
| Female | 1 to 4 | 0 | #NUM! | 0 | Baseline |
| Female | 5 to 14 | 0 | #NUM! | 0 | Baseline |
| Female | 5 to 14 | 0 | #NUM! | 0 | Baseline |
| Female | 5 to 14 | 0 | #NUM! | 0 | Baseline |
| Female | 15 to 21 | 0 | #NUM! | 0 | Baseline |
| Female | 15 to 21 | 0 | #NUM! | 0 | Baseline |
| Female | 15 to 21 | 0 | #NUM! | 0 | Baseline |
| Female | 5 to 14 | 0 | #NUM! | 0 | Baseline |
| Female | 15 to 21 | 0 | #NUM! | 0 | Baseline |
| Male | 5 to 14 | 0 | #NUM! | 0 | Baseline |
| Male | 15 to 21 | 0 | #NUM! | 0 | Baseline |
| Female | 15 to 21 | 0 | #NUM! | 0 | Baseline |
| Female | 15 to 21 | 0 | #NUM! | 0 | Baseline |

| Female | 15 to 21 | 0 | #NUM! | 0 | Baseline |
| --- | --- | --- | --- | --- | --- |
| Female | 15 to 21 | 0 | #NUM! | 0 | Baseline |
| Female | 5 to 14 | 0 | #NUM! | 0 | Baseline |
| Female | 15 to 21 | 0 | #NUM! | 0 | Baseline |
| Male | 5 to 14 | 0 | #NUM! | 0 | Baseline |
| Male | 5 to 14 | 0 | #NUM! | 0 | Baseline |
| Male | 5 to 14 | 0 | #NUM! | 0 | Baseline |
| Male | 5 to 14 | 0 | #NUM! | 0 | Baseline |
| Male | 5 to 14 | 0 | #NUM! | 0 | Baseline |
| Male | 5 to 14 | 0 | #NUM! | 0 | Baseline |
| Male | 15 to 21 | 0 | #NUM! | 0 | Baseline |
| Male | 5 to 14 | 0 | #NUM! | 0 | Baseline |
| Male | 5 to 14 | 0 | #NUM! | 0 | Baseline |
| Male | 5 to 14 | 0 | #NUM! | 0 | Baseline |
| Female | 15 to 21 | 0 | #NUM! | 0 | Baseline |
| Female | 15 to 21 | 0 | #NUM! | 0 | Baseline |
| Female | 5 to 14 | 0 | #NUM! | 0 | Baseline |
| Female | 5 to 14 | 0 | #NUM! | 0 | Baseline |
| Female | 15 to 21 | 0 | #NUM! | 0 | Baseline |
| Female | 15 to 21 | 0 | #NUM! | 0 | Baseline |
| Female | 5 to 14 | 0 | #NUM! | 0 | Baseline |
| Female | 5 to 14 | 0 | #NUM! | 0 | Baseline |
| Female | 5 to 14 | 0 | #NUM! | 0 | Baseline |
| Female | 5 to 14 | 0 | #NUM! | 0 | Baseline |
| Female | 5 to 14 | 0 | #NUM! | 0 | Baseline |
| Female | 5 to 14 | 0 | #NUM! | 0 | Baseline |
| Female | 5 to 14 | 0 | #NUM! | 0 | Baseline |
| Female | 5 to 14 | 0 | #NUM! | 0 | Baseline |
| Male | 5 to 14 | 0 | #NUM! | 0 | Baseline |
| Male | 5 to 14 | 0 | #NUM! | 0 | Baseline |
| Male | 5 to 14 | 0 | #NUM! | 0 | Baseline |
| Male | 5 to 14 | 0 | #NUM! | 0 | Baseline |
| Female | 5 to 14 | 0 | #NUM! | 0 | Baseline |
| Male | 5 to 14 | 0 | #NUM! | 0 | Baseline |
| Male | 5 to 14 | 0 | #NUM! | 0 | Baseline |
| Male | 5 to 14 | 0 | #NUM! | 0 | Baseline |
| Male | 5 to 14 | 0 | #NUM! | 0 | Baseline |
| Male | 5 to 14 | 0 | #NUM! | 0 | Baseline |
| Female | 5 to 14 | 0 | #NUM! | 0 | Baseline |
| Male | 15 to 21 | 0 | #NUM! | 0 | Baseline |
| Female | 5 to 14 | 0 | #NUM! | 0 | Baseline |
| Male | 15 to 21 | 0 | #NUM! | 0 | Baseline |
| Female | 5 to 14 | 0 | #NUM! | 0 | Baseline |
| Male | 15 to 21 | 0 | #NUM! | 0 | Baseline |
| Male | 15 to 21 | 0 | #NUM! | 0 | Baseline |
| Male | 15 to 21 | 0 | #NUM! | 0 | Baseline |
| Female | 15 to 21 | 0 | #NUM! | 0 | Baseline |
| Female | 5 to 14 | 0 | #NUM! | 0 | Baseline |
| Female | 5 to 14 | 0 | #NUM! | 0 | Baseline |
| Female | 5 to 14 | 0 | #NUM! | 0 | Baseline |

| Female | 5 to 14 | 0 | #NUM! | 0 | Baseline |
| --- | --- | --- | --- | --- | --- |
| Male | 1 to 4 | 0 | #NUM! | 0 | Baseline |
| Male | 1 to 4 | 0 | #NUM! | 0 | Baseline |
| Male | 1 to 4 | 0 | #NUM! | 0 | Baseline |
| Male | 1 to 4 | 0 | #NUM! | 0 | Baseline |
| Male | 1 to 4 | 0 | #NUM! | 0 | Baseline |
| Male | 1 to 4 | 0 | #NUM! | 0 | Baseline |
| Male | 1 to 4 | 0 | #NUM! | 0 | Baseline |
| Male | 1 to 4 | 0 | #NUM! | 0 | Baseline |
| Male | 1 to 4 | 0 | #NUM! | 0 | Baseline |
| Male | 1 to 4 | 0 | #NUM! | 0 | Baseline |
| Male | 1 to 4 | 0 | #NUM! | 0 | Baseline |
| Male | 1 to 4 | 0 | #NUM! | 0 | Baseline |
| Male | 1 to 4 | 0 | #NUM! | 0 | Baseline |
| Male | 1 to 4 | 0 | #NUM! | 0 | Baseline |
| Male | 1 to 4 | 0 | #NUM! | 0 | Baseline |
| Male | 1 to 4 | 0 | #NUM! | 0 | Baseline |
| Male | 1 to 4 | 0 | #NUM! | 0 | Baseline |
| Female | 35+ | 0 | #NUM! | 0 | Baseline |
| Male | 35+ | 0 | #NUM! | 0 | Baseline |
| Male | 35+ | 0 | #NUM! | 0 | Baseline |
| Male | 35+ | 0 | #NUM! | 0 | Baseline |
| Male | 35+ | 0 | #NUM! | 0 | Baseline |
| Female | 22 to 35 | 0 | #NUM! | 0 | Baseline |
| Male | 35+ | 0 | #NUM! | 0 | Baseline |
| Male | 35+ | 0 | #NUM! | 0 | Baseline |
| Female | 22 to 35 | 0 | #NUM! | 0 | Baseline |
| Female | 22 to 35 | 0 | #NUM! | 0 | Baseline |
| Male | 22 to 35 | 0 | #NUM! | 0 | Baseline |
| Male | 35+ | 0 | #NUM! | 0 | Baseline |
| Male | 22 to 35 | 0 | #NUM! | 0 | Baseline |
| Female | 35+ | 0 | #NUM! | 0 | Baseline |
| Male | 22 to 35 | 0 | #NUM! | 0 | Baseline |
| Female | 22 to 35 | 0 | #NUM! | 0 | Baseline |
| Male | 1 to 4 | 0 | #NUM! | 0 | Baseline |
| Male | 1 to 4 | 0 | #NUM! | 0 | Baseline |
| Male | 1 to 4 | 0 | #NUM! | 0 | Baseline |
| Male | 1 to 4 | 0 | #NUM! | 0 | Baseline |
| Male | 1 to 4 | 0 | #NUM! | 0 | Baseline |
| Male | 1 to 4 | 0 | #NUM! | 0 | Baseline |
| Female | 35+ | 0 | #NUM! | 0 | Baseline |
| Female | 15 to 21 | 0 | #NUM! | 0 | Baseline |
| Female | 5 to 14 | 0 | #NUM! | 0 | Baseline |
| Male | 5 to 14 | 0 | #NUM! | 0 | Baseline |
| Male | 22 to 35 | 0 | #NUM! | 0 | Baseline |
| Female | 5 to 14 | 0 | #NUM! | 0 | Baseline |
| Female | 22 to 35 | 0 | #NUM! | 0 | Baseline |
| Male | 5 to 14 | 0 | #NUM! | 0 | Baseline |
| Female | 5 to 14 | 0 | #NUM! | 0 | Baseline |
| Male | 22 to 35 | 3 | 0 | 0 | Baseline |

| Male | 5 to 14 | 0 | #NUM! | 0 | Baseline |
| --- | --- | --- | --- | --- | --- |
| Male | 5 to 14 | 0 | #NUM! | 0 | Baseline |
| Female | 5 to 14 | 3 | 0 | 0 | Baseline |
| Male | 5 to 14 | 0 | #NUM! | 0 | Baseline |
| Male | 15 to 21 | 0 | #NUM! | 0 | Baseline |
| Female | 15 to 21 | 0 | #NUM! | 0 | Baseline |
| Female | 5 to 14 | 0 | #NUM! | 0 | Baseline |
| Male | 5 to 14 | 0 | #NUM! | 0 | Baseline |
| Male | 22 to 35 | 0 | #NUM! | 0 | Baseline |
| Male | 15 to 21 | 0 | #NUM! | 0 | Baseline |
| Female | 15 to 21 | 0 | #NUM! | 0 | Baseline |
| Male | 15 to 21 | 0 | #NUM! | 0 | Baseline |
| Female | 5 to 14 | 0 | #NUM! | 0 | Baseline |
| Male | 15 to 21 | 0 | #NUM! | 0 | Baseline |
| Male | 15 to 21 | 0 | #NUM! | 0 | Baseline |
| Male | 5 to 14 | 0 | #NUM! | 0 | Baseline |
| Female | 15 to 21 | 0 | #NUM! | 0 | Baseline |
| Male | 15 to 21 | 0 | #NUM! | 0 | Baseline |
| Male | 22 to 35 | 0 | #NUM! | 0 | Baseline |
| Male | 15 to 21 | 0 | #NUM! | 0 | Baseline |
| Male | 5 to 14 | 0 | #NUM! | 0 | Baseline |
| Male | 1 to 4 | 4 | 0 | 0 | Baseline |
| Male | 22 to 35 | 0 | #NUM! | 0 | Baseline |
| Male | 5 to 14 | 0 | #NUM! | 0 | Baseline |
| Male | 22 to 35 | 0 | #NUM! | 0 | Baseline |
| Female | 15 to 21 | 0 | #NUM! | 0 | Baseline |
| Male | 5 to 14 | 0 | #NUM! | 0 | Baseline |
| Male | 5 to 14 | 0 | #NUM! | 0 | Baseline |
| Male | 22 to 35 | 3 | 0 | 0 | Baseline |
| Male | 15 to 21 | 0 | #NUM! | 0 | Baseline |
| Female | 15 to 21 | 0 | #NUM! | 0 | Baseline |
| Male | 5 to 14 | 0 | #NUM! | 0 | Baseline |
| Female | 5 to 14 | 0 | #NUM! | 0 | Baseline |
| Female | 22 to 35 | 0 | #NUM! | 0 | Baseline |
| Male | 22 to 35 | 0 | #NUM! | 0 | Baseline |
| Female | 22 to 35 | 0 | #NUM! | 0 | Baseline |
| Female | 5 to 14 | 0 | #NUM! | 0 | Baseline |
| Male | 22 to 35 | 0 | #NUM! | 0 | Baseline |
| Female | 35+ | 0 | #NUM! | 0 | Baseline |
| Male | 15 to 21 | 0 | #NUM! | 0 | Baseline |
| Female | 22 to 35 | 0 | #NUM! | 0 | Baseline |
| Female | 22 to 35 | 0 | #NUM! | 0 | Baseline |
| Female | 22 to 35 | 3 | 0 | 0 | Baseline |
| Female | 5 to 14 | 0 | #NUM! | 0 | Baseline |
| Female | 22 to 35 | 0 | #NUM! | 0 | Baseline |
| Male | 22 to 35 | 0 | #NUM! | 0 | Baseline |
| Male | 5 to 14 | 0 | #NUM! | 0 | Baseline |
| Female | 15 to 21 | 5 | 0 | 0 | Baseline |
| Male | 15 to 21 | 0 | #NUM! | 0 | Baseline |
| Male | 5 to 14 | 0 | #NUM! | 0 | Baseline |

| Female | 15 to 21 | 0 | #NUM! | 0 | Baseline |
| --- | --- | --- | --- | --- | --- |
| Male | 5 to 14 | 0 | #NUM! | 0 | Baseline |
| Male | 5 to 14 | 0 | #NUM! | 0 | Baseline |
| Female | 15 to 21 | 4 | 0 | 0 | Baseline |
| Male | 5 to 14 | 0 | #NUM! | 0 | Baseline |
| Female | 22 to 35 | 0 | #NUM! | 0 | Baseline |
| Male | 35+ | 0 | #NUM! | 0 | Baseline |
| Male | 22 to 35 | 0 | #NUM! | 0 | Baseline |
| Female | 35+ | 0 | #NUM! | 0 | Baseline |
| Female | 15 to 21 | 0 | #NUM! | 0 | Baseline |
| Male | 5 to 14 | 3 | 0 | 0 | Baseline |
| Female | 35+ | 0 | #NUM! | 0 | Baseline |
| Male | 15 to 21 | 0 | #NUM! | 0 | Baseline |
| Female | 5 to 14 | 0 | #NUM! | 0 | Baseline |
| Female | 22 to 35 | 4 | 0 | 0 | Baseline |
| Female | 22 to 35 | 0 | #NUM! | 0 | Baseline |
| Female | 5 to 14 | 3 | 0 | 0 | Baseline |
| Female | 22 to 35 | 0 | #NUM! | 0 | Baseline |
| Male | 35+ | 0 | #NUM! | 0 | Baseline |
| Female | 35+ | 0 | #NUM! | 0 | Baseline |
| Male | 22 to 35 | 0 | #NUM! | 0 | Baseline |
| Male | 5 to 14 | 0 | #NUM! | 0 | Baseline |
| Female | 22 to 35 | 0 | #NUM! | 0 | Baseline |
| Female | 5 to 14 | 0 | #NUM! | 0 | Baseline |
| Male | 35+ | 0 | #NUM! | 0 | Baseline |
| Female | 15 to 21 | 3 | 0 | 0 | Baseline |
| Male | 35+ | 0 | #NUM! | 0 | Baseline |
| Female | 5 to 14 | 0 | #NUM! | 0 | Baseline |
| Male | 22 to 35 | 0 | #NUM! | 0 | Baseline |
| Male | 15 to 21 | 0 | #NUM! | 0 | Baseline |
| Female | 35+ | 0 | #NUM! | 0 | Baseline |
| Male | 5 to 14 | 0 | #NUM! | 0 | Baseline |
| Male | 1 to 4 | 0 | #NUM! | 0 | Baseline |
| Female | 15 to 21 | 0 | #NUM! | 0 | Baseline |
| Male | 1 to 4 | 0 | #NUM! | 0 | Baseline |
| Male | 5 to 14 | 0 | #NUM! | 0 | Baseline |
| Male | 15 to 21 | 0 | #NUM! | 0 | Baseline |
| Male | 15 to 21 | 0 | #NUM! | 0 | Baseline |
| Male | 15 to 21 | 0 | #NUM! | 0 | Baseline |
| Male | 1 to 4 | 0 | #NUM! | 0 | Baseline |
| Male | 1 to 4 | 0 | #NUM! | 0 | Baseline |
| Male | 1 to 4 | 0 | #NUM! | 0 | Baseline |
| Male | 1 to 4 | 5 | 0 | 0 | Baseline |
| Female | 5 to 14 | 0 | #NUM! | 0 | Baseline |
| Female | 5 to 14 | 0 | #NUM! | 0 | Baseline |
| Female | 5 to 14 | 0 | #NUM! | 0 | Baseline |
| Male | 5 to 14 | 0 | #NUM! | 0 | Baseline |
| Female | 5 to 14 | 0 | #NUM! | 0 | Baseline |
| Male | 15 to 21 | 0 | #NUM! | 0 | Baseline |
| Male | 1 to 4 | 4 | 0 | 0 | Baseline |

| Male | 15 to 21 | 0 | #NUM! | 0 | Baseline |
| --- | --- | --- | --- | --- | --- |
| Female | 15 to 21 | 0 | #NUM! | 0 | Baseline |
| Male | 15 to 21 | 4 | 0 | 0 | Baseline |
| Male | 1 to 4 | 0 | #NUM! | 0 | Baseline |
| Male | 1 to 4 | 0 | #NUM! | 0 | Baseline |
| Male | 1 to 4 | 4 | 0 | 0 | Baseline |
| Male | 1 to 4 | 4 | 0 | 0 | Baseline |
| Female | 5 to 14 | 0 | #NUM! | 0 | Baseline |
| Male | 1 to 4 | 0 | #NUM! | 0 | Baseline |
| Male | 5 to 14 | 0 | #NUM! | 0 | Baseline |
| Male | 1 to 4 | 0 | #NUM! | 0 | Baseline |
| Female | 15 to 21 | 0 | #NUM! | 0 | Baseline |
| Male | 1 to 4 | 0 | #NUM! | 0 | Baseline |
| Male | 1 to 4 | 0 | #NUM! | 0 | Baseline |
| Female | 15 to 21 | 0 | #NUM! | 0 | Baseline |
| Male | 1 to 4 | 0 | #NUM! | 0 | Baseline |
| Male | 1 to 4 | 0 | #NUM! | 0 | Baseline |
| Male | 1 to 4 | 0 | #NUM! | 0 | Baseline |
| Male | 1 to 4 | 0 | #NUM! | 0 | Baseline |
| Male | 1 to 4 | 0 | #NUM! | 0 | Baseline |
| Male | 1 to 4 | 0 | #NUM! | 0 | Baseline |
| Male | 1 to 4 | 0 | #NUM! | 0 | Baseline |
| Male | 1 to 4 | 0 | #NUM! | 0 | Baseline |
| Male | 1 to 4 | 0 | #NUM! | 0 | Baseline |
| Male | 1 to 4 | 0 | #NUM! | 0 | Baseline |
| Male | 1 to 4 | 0 | #NUM! | 0 | Baseline |
| Male | 1 to 4 | 0 | #NUM! | 0 | Baseline |
| Male | 1 to 4 | 0 | #NUM! | 0 | Baseline |
| Male | 1 to 4 | 0 | #NUM! | 0 | Baseline |
| Male | 1 to 4 | 0 | #NUM! | 0 | Baseline |
| Male | 1 to 4 | 0 | #NUM! | 0 | Baseline |
| Male | 1 to 4 | 0 | #NUM! | 0 | Baseline |
| Male | 1 to 4 | 0 | #NUM! | 0 | Baseline |
| Male | 1 to 4 | 0 | #NUM! | 0 | Baseline |
| Male | 1 to 4 | 0 | #NUM! | 0 | Baseline |
| Male | 1 to 4 | 0 | #NUM! | 0 | Baseline |
| Male | 1 to 4 | 0 | #NUM! | 0 | Baseline |
| Male | 1 to 4 | 0 | #NUM! | 0 | Baseline |
| Male | 1 to 4 | 0 | #NUM! | 0 | Baseline |
| Male | 1 to 4 | 0 | #NUM! | 0 | Baseline |
| Male | 1 to 4 | 0 | #NUM! | 0 | Baseline |
| Male | 1 to 4 | 0 | #NUM! | 0 | Baseline |
| Male | 1 to 4 | 0 | #NUM! | 0 | Baseline |
| Male | 1 to 4 | 0 | #NUM! | 0 | Baseline |
| Male | 1 to 4 | 0 | #NUM! | 0 | Baseline |
| Male | 1 to 4 | 0 | #NUM! | 0 | Baseline |
| Male | 1 to 4 | 0 | #NUM! | 0 | Baseline |
| Male | 1 to 4 | 0 | #NUM! | 0 | Baseline |
| Male | 1 to 4 | 0 | #NUM! | 0 | Baseline |
| Male | 1 to 4 | 0 | #NUM! | 0 | Baseline |

| Male | 1 to 4 | 0 | #NUM! | 0 | Baseline |
| --- | --- | --- | --- | --- | --- |
| Male | 1 to 4 | 0 | #NUM! | 0 | Baseline |
| Male | 1 to 4 | 0 | #NUM! | 0 | Baseline |
| Male | 1 to 4 | 0 | #NUM! | 0 | Baseline |
| Male | 1 to 4 | 0 | #NUM! | 0 | Baseline |
| Male | 1 to 4 | 0 | #NUM! | 0 | Baseline |
| Male | 1 to 4 | 0 | #NUM! | 0 | Baseline |
| Male | 1 to 4 | 0 | #NUM! | 0 | Baseline |
| Male | 1 to 4 | 0 | #NUM! | 0 | Baseline |
| Male | 1 to 4 | 0 | #NUM! | 0 | Baseline |
| Male | 1 to 4 | 0 | #NUM! | 0 | Baseline |
| Male | 1 to 4 | 0 | #NUM! | 0 | Baseline |
| Male | 1 to 4 | 0 | #NUM! | 0 | Baseline |
| Male | 1 to 4 | 0 | #NUM! | 0 | Baseline |
| Male | 1 to 4 | 0 | #NUM! | 0 | Baseline |
| Male | 1 to 4 | 0 | #NUM! | 0 | Baseline |
| Male | 1 to 4 | 0 | #NUM! | 0 | Baseline |
| Male | 1 to 4 | 0 | #NUM! | 0 | Baseline |
| Male | 1 to 4 | 0 | #NUM! | 0 | Baseline |
| Male | 22 to 35 | 0 | #NUM! | 0 | Baseline |
| Male | 22 to 35 | 0 | #NUM! | 0 | Baseline |
| Female | 15 to 21 | 0 | #NUM! | 0 | Baseline |
| Male | 22 to 35 | 0 | #NUM! | 0 | Baseline |
| Female | 15 to 21 | 0 | #NUM! | 0 | Baseline |
| Male | 1 to 4 | 0 | #NUM! | 0 | Baseline |
| Male | 1 to 4 | 0 | #NUM! | 0 | Baseline |
| Male | 1 to 4 | 0 | #NUM! | 0 | Baseline |
| Male | 1 to 4 | 0 | #NUM! | 0 | Baseline |
| Male | 1 to 4 | 0 | #NUM! | 0 | Baseline |
| Male | 1 to 4 | 0 | #NUM! | 0 | Baseline |
| Male | 1 to 4 | 0 | #NUM! | 0 | Baseline |
| Male | 1 to 4 | 0 | #NUM! | 0 | Baseline |
| Male | 1 to 4 | 0 | #NUM! | 0 | Baseline |
| Male | 1 to 4 | 0 | #NUM! | 0 | Baseline |
| Male | 1 to 4 | 0 | #NUM! | 0 | Baseline |
| Male | 1 to 4 | 0 | #NUM! | 0 | Baseline |
| Male | 1 to 4 | 0 | #NUM! | 0 | Baseline |
| Female | 22 to 35 | 0 | #NUM! | 0 | Baseline |
| Male | 1 to 4 | 0 | #NUM! | 0 | Baseline |
| Male | 1 to 4 | 0 | #NUM! | 0 | Baseline |
| Male | 1 to 4 | 0 | #NUM! | 0 | Baseline |
| Male | 1 to 4 | 0 | #NUM! | 0 | Baseline |
| Male | 1 to 4 | 0 | #NUM! | 0 | Baseline |
| Male | 1 to 4 | 0 | #NUM! | 0 | Baseline |
| Male | 1 to 4 | 0 | #NUM! | 0 | Baseline |
| Male | 1 to 4 | 0 | #NUM! | 0 | Baseline |
| Male | 1 to 4 | 0 | #NUM! | 0 | Baseline |
| Male | 1 to 4 | 0 | #NUM! | 0 | Baseline |
| Male | 1 to 4 | 0 | #NUM! | 0 | Baseline |
| Male | 1 to 4 | 0 | #NUM! | 0 | Baseline |

| Male | 1 to 4 | 0 | #NUM! | 0 | Baseline |
| --- | --- | --- | --- | --- | --- |
| Male | 1 to 4 | 0 | #NUM! | 0 | Baseline |
| Male | 1 to 4 | 0 | #NUM! | 0 | Baseline |
| Male | 1 to 4 | 0 | #NUM! | 0 | Baseline |
| Male | 1 to 4 | 0 | #NUM! | 0 | Baseline |
| Male | 1 to 4 | 0 | #NUM! | 0 | Baseline |
| Male | 1 to 4 | 0 | #NUM! | 0 | Baseline |
| Male | 1 to 4 | 0 | #NUM! | 0 | Baseline |
| Male | 1 to 4 | 0 | #NUM! | 0 | Baseline |
| Male | 1 to 4 | 0 | #NUM! | 0 | Baseline |
| Male | 1 to 4 | 0 | #NUM! | 0 | Baseline |
| Male | 1 to 4 | 0 | #NUM! | 0 | Baseline |
| Male | 1 to 4 | 0 | #NUM! | 0 | Baseline |
| Male | 1 to 4 | 0 | #NUM! | 0 | Baseline |
| Male | 1 to 4 | 0 | #NUM! | 0 | Baseline |
| Male | 1 to 4 | 0 | #NUM! | 0 | Baseline |
| Male | 1 to 4 | 0 | #NUM! | 0 | Baseline |
| Male | 1 to 4 | 0 | #NUM! | 0 | Baseline |
| Male | 1 to 4 | 0 | #NUM! | 0 | Baseline |
| Male | 1 to 4 | 0 | #NUM! | 0 | Baseline |
| Male | 1 to 4 | 0 | #NUM! | 0 | Baseline |
| Male | 1 to 4 | 0 | #NUM! | 0 | Baseline |
| Female | 22 to 35 | 0 | #NUM! | 0 | Baseline |
| Male | 1 to 4 | 0 | #NUM! | 0 | Baseline |
| Male | 1 to 4 | 0 | #NUM! | 0 | Baseline |
| Male | 1 to 4 | 0 | #NUM! | 0 | Baseline |
| Male | 1 to 4 | 0 | #NUM! | 0 | Baseline |
| Female | 5 to 14 | 0 | #NUM! | 0 | Baseline |
| Male | 5 to 14 | 0 | #NUM! | 0 | Baseline |
| Male | 1 to 4 | 0 | #NUM! | 0 | Baseline |
| Male | 5 to 14 | 0 | #NUM! | 0 | Baseline |
| Male | 5 to 14 | 0 | #NUM! | 0 | Baseline |
| Male | 5 to 14 | 0 | #NUM! | 0 | Baseline |
| Male | 5 to 14 | 0 | #NUM! | 0 | Baseline |
| Male | 5 to 14 | 0 | #NUM! | 0 | Baseline |
| Male | 5 to 14 | 0 | #NUM! | 0 | Baseline |
| Female | 5 to 14 | 0 | #NUM! | 0 | Baseline |
| Female | 5 to 14 | 0 | #NUM! | 0 | Baseline |
| Female | 5 to 14 | 0 | #NUM! | 0 | Baseline |
| Male | 15 to 21 | 0 | #NUM! | 0 | Baseline |
| Male | 1 to 4 | 0 | #NUM! | 0 | Baseline |
| Male | 22 to 35 | 0 | #NUM! | 0 | Baseline |
| Male | 15 to 21 | 0 | #NUM! | 0 | Baseline |
| Male | 5 to 14 | 0 | #NUM! | 0 | Baseline |
| Male | 15 to 21 | 0 | #NUM! | 0 | Baseline |
| Male | 5 to 14 | 0 | #NUM! | 0 | Baseline |
| Female | 5 to 14 | 0 | #NUM! | 0 | Baseline |
| Female | 15 to 21 | 0 | #NUM! | 0 | Baseline |
| Female | 5 to 14 | 0 | #NUM! | 0 | Baseline |
| Female | 5 to 14 | 0 | #NUM! | 0 | Baseline |

| Male | 35+ | 0 | #NUM! | 0 | Baseline |
| --- | --- | --- | --- | --- | --- |
| Male | 1 to 4 | 0 | #NUM! | 0 | Baseline |
| Male | 35+ | 0 | #NUM! | 0 | Baseline |
| Female | 35+ | 0 | #NUM! | 0 | Baseline |
| Male | 35+ | 0 | #NUM! | 0 | Baseline |
| Female | 35+ | 0 | #NUM! | 0 | Baseline |
| Female | 35+ | 0 | #NUM! | 0 | Baseline |
| Male | 35+ | 0 | #NUM! | 0 | Baseline |
| Male | 35+ | 0 | #NUM! | 0 | Baseline |
| Male | 1 to 4 | 0 | #NUM! | 0 | Baseline |
| Female | 35+ | 0 | #NUM! | 0 | Baseline |
| Female | 1 to 4 | 0 | #NUM! | 0 | Baseline |
| Female | 1 to 4 | 0 | #NUM! | 0 | Baseline |
| Female | 5 to 14 | 0 | #NUM! | 0 | Baseline |
| Male | 5 to 14 | 0 | #NUM! | 0 | Baseline |
| Female | 1 to 4 | 0 | #NUM! | 0 | Baseline |
| Female | 1 to 4 | 0 | #NUM! | 0 | Baseline |
| Male | 22 to 35 | 0 | #NUM! | 0 | Baseline |
| Male | 35+ | 0 | #NUM! | 0 | Baseline |
| Female | 1 to 4 | 0 | #NUM! | 0 | Baseline |
| Male | 35+ | 0 | #NUM! | 0 | Baseline |
| Male | 35+ | 0 | #NUM! | 0 | Baseline |
| Female | 22 to 35 | 0 | #NUM! | 0 | Baseline |
| Female | 35+ | 0 | #NUM! | 0 | Baseline |
| Male | 1 to 4 | 0 | #NUM! | 0 | Baseline |
| Male | 15 to 21 | 0 | #NUM! | 0 | Baseline |
| Female | 5 to 14 | 0 | #NUM! | 0 | Baseline |
| Female | 5 to 14 | 0 | #NUM! | 0 | Baseline |
| Female | 15 to 21 | 0 | #NUM! | 0 | Baseline |
| Male | 15 to 21 | 0 | #NUM! | 0 | Baseline |
| Female | 5 to 14 | 0 | #NUM! | 0 | Baseline |
| Female | 5 to 14 | 0 | #NUM! | 0 | Baseline |
| Male | 22 to 35 | 0 | #NUM! | 0 | Baseline |
| Male | 22 to 35 | 0 | #NUM! | 0 | Baseline |
| Female | 22 to 35 | 0 | #NUM! | 0 | Baseline |
| Male | 22 to 35 | 0 | #NUM! | 0 | Baseline |
| Female | 22 to 35 | 0 | #NUM! | 0 | Baseline |
| Female | 22 to 35 | 0 | #NUM! | 0 | Baseline |
| Male | 22 to 35 | 0 | #NUM! | 0 | Baseline |
| Male | 22 to 35 | 0 | #NUM! | 0 | Baseline |
| Male | 22 to 35 | 0 | #NUM! | 0 | Baseline |
| Male | 22 to 35 | 0 | #NUM! | 0 | Baseline |
| Female | 1 to 4 | 0 | #NUM! | 0 | Baseline |
| Female | 1 to 4 | 0 | #NUM! | 0 | Baseline |
| Female | 35+ | 0 | #NUM! | 0 | Baseline |
| Male | 1 to 4 | 0 | #NUM! | 0 | Baseline |
| Female | 1 to 4 | 0 | #NUM! | 0 | Baseline |
| Female | 35+ | 0 | #NUM! | 0 | Baseline |
| Female | 5 to 14 | 0 | #NUM! | 0 | Baseline |
| Female | 35+ | 0 | #NUM! | 0 | Baseline |

| Male | 5 to 14 | 0 | #NUM! | 0 | Baseline |
| --- | --- | --- | --- | --- | --- |
| Female | 35+ | 0 | #NUM! | 0 | Baseline |
| Male | 35+ | 0 | #NUM! | 0 | Baseline |
| Male | 5 to 14 | 0 | #NUM! | 0 | Baseline |
| Female | 1 to 4 | 0 | #NUM! | 0 | Baseline |
| Female | 35+ | 0 | #NUM! | 0 | Baseline |
| Female | 5 to 14 | 0 | #NUM! | 0 | Baseline |
| Male | 15 to 21 | 0 | #NUM! | 0 | Baseline |
| Male | 5 to 14 | 0 | #NUM! | 0 | Baseline |
| Male | 22 to 35 | 0 | #NUM! | 0 | Baseline |
| Male | 5 to 14 | 0 | #NUM! | 0 | Baseline |
| Female | 5 to 14 | 0 | #NUM! | 0 | Baseline |
| Male | 5 to 14 | 0 | #NUM! | 0 | Baseline |
| Male | 5 to 14 | 0 | #NUM! | 0 | Baseline |
| Male | 5 to 14 | 0 | #NUM! | 0 | Baseline |
| Female | 15 to 21 | 0 | #NUM! | 0 | Baseline |
| Female | 5 to 14 | 0 | #NUM! | 0 | Baseline |
| Male | 35+ | 0 | #NUM! | 0 | Baseline |
| Male | 35+ | 0 | #NUM! | 0 | Baseline |
| Male | 22 to 35 | 0 | #NUM! | 0 | Baseline |
| Female | 22 to 35 | 0 | #NUM! | 0 | Baseline |
| Female | 22 to 35 | 0 | #NUM! | 0 | Baseline |
| Male | 22 to 35 | 0 | #NUM! | 0 | Baseline |
| Male | 22 to 35 | 0 | #NUM! | 0 | Baseline |
| Male | 22 to 35 | 0 | #NUM! | 0 | Baseline |
| Female | 22 to 35 | 0 | #NUM! | 0 | Baseline |
| Male | 22 to 35 | 0 | #NUM! | 0 | Baseline |
| Female | 15 to 21 | 0 | #NUM! | 0 | Baseline |
| Male | 15 to 21 | 0 | #NUM! | 0 | Baseline |
| Male | 15 to 21 | 0 | #NUM! | 0 | Baseline |
| Female | 5 to 14 | 0 | #NUM! | 0 | Baseline |
| Female | 15 to 21 | 0 | #NUM! | 0 | Baseline |
| Male | 15 to 21 | 0 | #NUM! | 0 | Baseline |
| Female | 5 to 14 | 0 | #NUM! | 0 | Baseline |
| Female | 5 to 14 | 0 | #NUM! | 0 | Baseline |
| Female | 15 to 21 | 0 | #NUM! | 0 | Baseline |
| Male | 15 to 21 | 0 | #NUM! | 0 | Baseline |
| Female | 35+ | 0 | #NUM! | 0 | Baseline |
| Male | 22 to 35 | 0 | #NUM! | 0 | Baseline |
| Female | 5 to 14 | 0 | #NUM! | 0 | Baseline |
| Male | 35+ | 0 | #NUM! | 0 | Baseline |
| Male | 35+ | 0 | #NUM! | 0 | Baseline |
| Male | 22 to 35 | 0 | #NUM! | 0 | Baseline |
| Male | 1 to 4 | 0 | #NUM! | 0 | Baseline |
| Female | 22 to 35 | 0 | #NUM! | 0 | Baseline |
| Female | 35+ | 0 | #NUM! | 0 | Baseline |
| Female | 35+ | 0 | #NUM! | 0 | Baseline |
| Male | 5 to 14 | 0 | #NUM! | 0 | Baseline |
| Male | 22 to 35 | 0 | #NUM! | 0 | Baseline |
| Male | 22 to 35 | 0 | #NUM! | 0 | Baseline |

| Female | 15 to 21 | 0 | #NUM! | 0 | Baseline |
| --- | --- | --- | --- | --- | --- |
| Female | 5 to 14 | 0 | #NUM! | 0 | Baseline |
| Male | 5 to 14 | 0 | #NUM! | 0 | Baseline |
| Female | 5 to 14 | 0 | #NUM! | 0 | Baseline |
| Female | 5 to 14 | 0 | #NUM! | 0 | Baseline |
| Female | 15 to 21 | 0 | #NUM! | 0 | Baseline |
| Male | 22 to 35 | 0 | #NUM! | 0 | Baseline |
| Male | 15 to 21 | 0 | #NUM! | 0 | Baseline |
| Female | 22 to 35 | 0 | #NUM! | 0 | Baseline |
| Female | 15 to 21 | 0 | #NUM! | 0 | Baseline |
| Female | 22 to 35 | 0 | #NUM! | 0 | Baseline |
| Female | 15 to 21 | 0 | #NUM! | 0 | Baseline |
| Male | 35+ | 0 | #NUM! | 0 | Baseline |
| Male | 22 to 35 | 0 | #NUM! | 0 | Baseline |
| Male | 35+ | 0 | #NUM! | 0 | Baseline |
| Male | 35+ | 0 | #NUM! | 0 | Baseline |
| Male | 1 to 4 | 0 | #NUM! | 0 | Baseline |
| Male | 35+ | 0 | #NUM! | 0 | Baseline |
| Female | 22 to 35 | 0 | #NUM! | 0 | Baseline |
| Female | 35+ | 0 | #NUM! | 0 | Baseline |
| Female | 22 to 35 | 0 | #NUM! | 0 | Baseline |
| Female | 5 to 14 | 0 | #NUM! | 0 | Baseline |
| Female | 1 to 4 | 0 | #NUM! | 0 | Baseline |
| Female | 1 to 4 | 0 | #NUM! | 0 | Baseline |
| Female | 1 to 4 | 0 | #NUM! | 0 | Baseline |
| Female | 22 to 35 | 0 | #NUM! | 0 | Baseline |
| Male | 5 to 14 | 0 | #NUM! | 0 | Baseline |
| Female | 35+ | 0 | #NUM! | 0 | Baseline |
| Female | 35+ | 0 | #NUM! | 0 | Baseline |
| Male | 35+ | 0 | #NUM! | 0 | Baseline |
| Male | 22 to 35 | 0 | #NUM! | 0 | Baseline |
| Female | 5 to 14 | 0 | #NUM! | 0 | Baseline |
| Female | 5 to 14 | 0 | #NUM! | 0 | Baseline |
| Female | 15 to 21 | 0 | #NUM! | 0 | Baseline |
| Female | 5 to 14 | 0 | #NUM! | 0 | Baseline |
| Male | 1 to 4 | 0 | #NUM! | 0 | Baseline |
| Female | 15 to 21 | 0 | #NUM! | 0 | Baseline |
| Female | 5 to 14 | 0 | #NUM! | 0 | Baseline |
| Female | 35+ | 0 | #NUM! | 0 | Baseline |
| Male | 35+ | 0 | #NUM! | 0 | Baseline |
| Female | 35+ | 0 | #NUM! | 0 | Baseline |
| Male | 5 to 14 | 0 | #NUM! | 0 | Baseline |
| Male | 5 to 14 | 0 | #NUM! | 0 | Baseline |
| Female | 15 to 21 | 0 | #NUM! | 0 | Baseline |
| Female | 15 to 21 | 0 | #NUM! | 0 | Baseline |
| Male | 15 to 21 | 0 | #NUM! | 0 | Baseline |
| Female | 5 to 14 | 0 | #NUM! | 0 | Baseline |
| Female | 15 to 21 | 0 | #NUM! | 0 | Baseline |
| Male | 22 to 35 | 0 | #NUM! | 0 | Baseline |
| Female | 35+ | 0 | #NUM! | 0 | Baseline |

| Female | 35+ | 0 | #NUM! | 0 | Baseline |
| --- | --- | --- | --- | --- | --- |
| Female | 35+ | 0 | #NUM! | 0 | Baseline |
| Female | 35+ | 0 | #NUM! | 0 | Baseline |
| Male | 22 to 35 | 0 | #NUM! | 0 | Baseline |
| Female | 22 to 35 | 0 | #NUM! | 0 | Baseline |
| Female | 22 to 35 | 0 | #NUM! | 0 | Baseline |
| Male | 22 to 35 | 0 | #NUM! | 0 | Baseline |
| Female | 22 to 35 | 0 | #NUM! | 0 | Baseline |
| Female | 22 to 35 | 0 | #NUM! | 0 | Baseline |
| Female | 22 to 35 | 0 | #NUM! | 0 | Baseline |
| Female | 1 to 4 | 0 | #NUM! | 0 | Baseline |
| Female | 22 to 35 | 0 | #NUM! | 0 | Baseline |
| Female | 22 to 35 | 0 | #NUM! | 0 | Baseline |
| Female | 22 to 35 | 0 | #NUM! | 0 | Baseline |
| Male | 1 to 4 | 0 | #NUM! | 0 | Baseline |
| Male | 1 to 4 | 0 | #NUM! | 0 | Baseline |
| Male | 15 to 21 | 0 | #NUM! | 0 | Baseline |
| Female | 1 to 4 | 0 | #NUM! | 0 | Baseline |
| Female | 22 to 35 | 0 | #NUM! | 0 | Baseline |
| Male | 1 to 4 | 0 | #NUM! | 0 | Baseline |
| Female | 35+ | 0 | #NUM! | 0 | Baseline |
| Female | 35+ | 0 | #NUM! | 0 | Baseline |
| Male | 1 to 4 | 0 | #NUM! | 0 | Baseline |
| Male | 1 to 4 | 0 | #NUM! | 0 | Baseline |
| Female | 35+ | 0 | #NUM! | 0 | Baseline |
| Male | 1 to 4 | 0 | #NUM! | 0 | Baseline |
| Female | 5 to 14 | 0 | #NUM! | 0 | Baseline |
| Female | 35+ | 0 | #NUM! | 0 | Baseline |
| Female | 35+ | 0 | #NUM! | 0 | Baseline |
| Male | 35+ | 0 | #NUM! | 0 | Baseline |
| Female | 22 to 35 | 0 | #NUM! | 0 | Baseline |
| Male | 35+ | 0 | #NUM! | 0 | Baseline |
| Female | 22 to 35 | 0 | #NUM! | 0 | Baseline |
| Female | 35+ | 0 | #NUM! | 0 | Baseline |
| Female | 1 to 4 | 0 | #NUM! | 0 | Baseline |
| Male | 1 to 4 | 0 | #NUM! | 0 | Baseline |
| Female | 35+ | 0 | #NUM! | 0 | Baseline |
| Female | 22 to 35 | 0 | #NUM! | 0 | Baseline |
| Male | 35+ | 0 | #NUM! | 0 | Baseline |
| Female | 35+ | 0 | #NUM! | 0 | Baseline |
| Female | 22 to 35 | 0 | #NUM! | 0 | Baseline |
| Female | 22 to 35 | 0 | #NUM! | 0 | Baseline |
| Female | 1 to 4 | 0 | #NUM! | 0 | Baseline |
| Female | 1 to 4 | 0 | #NUM! | 0 | Baseline |
| Male | 35+ | 0 | #NUM! | 0 | Baseline |
| Male | 35+ | 0 | #NUM! | 0 | Baseline |
| Female | 22 to 35 | 0 | #NUM! | 0 | Baseline |
| Female | 35+ | 0 | #NUM! | 0 | Baseline |
| Male | 22 to 35 | 0 | #NUM! | 0 | Baseline |
| Female | 22 to 35 | 0 | #NUM! | 0 | Baseline |

| Female | 15 to 21 | 0 | #NUM! | 0 | Baseline |
| --- | --- | --- | --- | --- | --- |
| Female | 1 to 4 | 0 | #NUM! | 0 | Baseline |
| Female | 35+ | 0 | #NUM! | 0 | Baseline |
| Male | 22 to 35 | 0 | #NUM! | 0 | Baseline |
| Male | 35+ | 0 | #NUM! | 0 | Baseline |
| Female | 22 to 35 | 0 | #NUM! | 0 | Baseline |
| Female | 22 to 35 | 0 | #NUM! | 0 | Baseline |
| Female | 22 to 35 | 0 | #NUM! | 0 | Baseline |
| Male | 5 to 14 | 0 | #NUM! | 0 | Baseline |
| Female | 22 to 35 | 0 | #NUM! | 0 | Baseline |
| Male | 22 to 35 | 0 | #NUM! | 0 | Baseline |
| Male | 22 to 35 | 0 | #NUM! | 0 | Baseline |
| Male | 35+ | 0 | #NUM! | 0 | Baseline |
| Female | 22 to 35 | 0 | #NUM! | 0 | Baseline |
| Male | 35+ | 0 | #NUM! | 0 | Baseline |
| Female | 35+ | 0 | #NUM! | 0 | Baseline |
| Female | 22 to 35 | 0 | #NUM! | 0 | Baseline |
| Female | 1 to 4 | 0 | #NUM! | 0 | Baseline |
| Female | 22 to 35 | 0 | #NUM! | 0 | Baseline |
| Female | 22 to 35 | 0 | #NUM! | 0 | Baseline |
| Male | 35+ | 0 | #NUM! | 0 | Baseline |
| Female | 22 to 35 | 0 | #NUM! | 0 | Baseline |
| Male | 35+ | 0 | #NUM! | 0 | Baseline |
| Female | 5 to 14 | 0 | #NUM! | 0 | Baseline |
| Male | 35+ | 0 | #NUM! | 0 | Baseline |
| Female | 22 to 35 | 0 | #NUM! | 0 | Baseline |
| Female | 15 to 21 | 0 | #NUM! | 0 | Baseline |
| Female | 1 to 4 | 0 | #NUM! | 0 | Baseline |
| Female | 35+ | 0 | #NUM! | 0 | Baseline |
| Male | 35+ | 0 | #NUM! | 0 | Baseline |
| Male | 35+ | 0 | #NUM! | 0 | Baseline |
| Female | 35+ | 5 | 0 | 0 | Baseline |
| Female | 35+ | 0 | #NUM! | 0 | Baseline |
| Female | 35+ | 0 | #NUM! | 0 | Baseline |
| Male | 35+ | 0 | #NUM! | 0 | Baseline |
| Male | 22 to 35 | 0 | #NUM! | 0 | Baseline |
| Male | 35+ | 0 | #NUM! | 0 | Baseline |
| Female | 22 to 35 | 0 | #NUM! | 0 | Baseline |
| Female | 35+ | 0 | #NUM! | 0 | Baseline |
| Female | 22 to 35 | 0 | #NUM! | 0 | Baseline |
| Female | 22 to 35 | 0 | #NUM! | 0 | Baseline |
| Male | 1 to 4 | 0 | #NUM! | 0 | Baseline |
| Male | 1 to 4 | 0 | #NUM! | 0 | Baseline |
| Female | 1 to 4 | 0 | #NUM! | 0 | Baseline |
| Male | 35+ | 0 | #NUM! | 0 | Baseline |
| Female | 35+ | 0 | #NUM! | 0 | Baseline |
| Male | 35+ | 0 | #NUM! | 0 | Baseline |
| Female | 1 to 4 | 0 | #NUM! | 0 | Baseline |
| Female | 1 to 4 | 0 | #NUM! | 0 | Baseline |
| Female | 1 to 4 | 0 | #NUM! | 0 | Baseline |

| Male | 22 to 35 | 0 | #NUM! | 0 | Baseline |
| --- | --- | --- | --- | --- | --- |
| Female | 1 to 4 | 0 | #NUM! | 0 | Baseline |
| Male | 35+ | 0 | #NUM! | 0 | Baseline |
| Female | 35+ | 0 | #NUM! | 0 | Baseline |
| Female | 1 to 4 | 0 | #NUM! | 0 | Baseline |
| Male | 1 to 4 | 0 | #NUM! | 0 | Baseline |
| Male | 1 to 4 | 0 | #NUM! | 0 | Baseline |
| Female | 1 to 4 | 0 | #NUM! | 0 | Baseline |
| Male | 1 to 4 | 0 | #NUM! | 0 | Baseline |
| Female | 22 to 35 | 0 | #NUM! | 0 | Baseline |
| Male | 35+ | 0 | #NUM! | 0 | Baseline |
| Female | 22 to 35 | 0 | #NUM! | 0 | Baseline |
| Female | 5 to 14 | 0 | #NUM! | 0 | Baseline |
| Female | 15 to 21 | 4 | 0 | 0 | Baseline |
| Male | 15 to 21 | 0 | #NUM! | 0 | Baseline |
| Male | 5 to 14 | 0 | #NUM! | 0 | Baseline |
| Male | 15 to 21 | 0 | #NUM! | 0 | Baseline |
| Male | 35+ | 0 | #NUM! | 0 | Baseline |
| Male | 35+ | 0 | #NUM! | 0 | Baseline |
| Male | 1 to 4 | 0 | #NUM! | 0 | Baseline |
| Female | 35+ | 0 | #NUM! | 0 | Baseline |
| Male | 35+ | 0 | #NUM! | 0 | Baseline |
| Male | 5 to 14 | 0 | #NUM! | 0 | Baseline |
| Male | 15 to 21 | 0 | #NUM! | 0 | Baseline |
| Female | 15 to 21 | 0 | #NUM! | 0 | Baseline |
| Female | 15 to 21 | 0 | #NUM! | 0 | Baseline |
| Male | 5 to 14 | 0 | #NUM! | 0 | Baseline |
| Male | 1 to 4 | 0 | #NUM! | 0 | Baseline |
| Male | 22 to 35 | 0 | #NUM! | 0 | Baseline |
| Male | 1 to 4 | 0 | #NUM! | 0 | Baseline |
| Male | 1 to 4 | 0 | #NUM! | 0 | Baseline |
| Female | 1 to 4 | 0 | #NUM! | 0 | Baseline |
| Male | 1 to 4 | 0 | #NUM! | 0 | Baseline |
| Female | 1 to 4 | 0 | #NUM! | 0 | Baseline |
| Female | 1 to 4 | 0 | #NUM! | 0 | Baseline |
| Female | 1 to 4 | 0 | #NUM! | 0 | Baseline |
| Male | 1 to 4 | 0 | #NUM! | 0 | Baseline |
| Female | 1 to 4 | 0 | #NUM! | 0 | Baseline |
| Female | 1 to 4 | 0 | #NUM! | 0 | Baseline |
| Female | 35+ | 0 | #NUM! | 0 | Baseline |
| Female | 35+ | 0 | #NUM! | 0 | Baseline |
| Male | 15 to 21 | 0 | #NUM! | 0 | Baseline |
| Male | 15 to 21 | 0 | #NUM! | 0 | Baseline |
| Male | 15 to 21 | 0 | #NUM! | 0 | Baseline |
| Female | 15 to 21 | 0 | #NUM! | 0 | Baseline |
| Female | 15 to 21 | 0 | #NUM! | 0 | Baseline |
| Female | 5 to 14 | 0 | #NUM! | 0 | Baseline |
| Female | 15 to 21 | 0 | #NUM! | 0 | Baseline |
| Male | 5 to 14 | 0 | #NUM! | 0 | Baseline |
| Male | 15 to 21 | 0 | #NUM! | 0 | Baseline |

| Female | 15 to 21 | 0 | #NUM! | 0 | Baseline |
| --- | --- | --- | --- | --- | --- |
| Male | 22 to 35 | 0 | #NUM! | 0 | Baseline |
| Female | 15 to 21 | 0 | #NUM! | 0 | Baseline |
| Female | 5 to 14 | 0 | #NUM! | 0 | Baseline |
| Male | 15 to 21 | 0 | #NUM! | 0 | Baseline |
| Male | 35+ | 1 | 0 | 0 | Baseline |
| Male | 35+ | 0 | #NUM! | 0 | Baseline |
| Female | 1 to 4 | 0 | #NUM! | 0 | Baseline |
| Male | 15 to 21 | 0 | #NUM! | 0 | Baseline |
| Male | 1 to 4 | 0 | #NUM! | 0 | Baseline |
| Male | 22 to 35 | 0 | #NUM! | 0 | Baseline |
| Male | 1 to 4 | 0 | #NUM! | 0 | Baseline |
| Female | 22 to 35 | 0 | #NUM! | 0 | Baseline |
| Male | 35+ | 0 | #NUM! | 0 | Baseline |
| Male | 35+ | 0 | #NUM! | 0 | Baseline |
| Male | 35+ | 0 | #NUM! | 0 | Baseline |
| Female | 15 to 21 | 0 | #NUM! | 0 | Baseline |
| Male | 15 to 21 | 0 | #NUM! | 0 | Baseline |
| Male | 15 to 21 | 0 | #NUM! | 0 | Baseline |
| Male | 1 to 4 | 0 | #NUM! | 0 | Baseline |
| Male | 1 to 4 | 0 | #NUM! | 0 | Baseline |
| Male | 5 to 14 | 0 | #NUM! | 0 | Baseline |
| Female | 15 to 21 | 0 | #NUM! | 0 | Baseline |
| Female | 22 to 35 | 0 | #NUM! | 0 | Baseline |
| Female | 22 to 35 | 0 | #NUM! | 0 | Baseline |
| Male | 22 to 35 | 0 | #NUM! | 0 | Baseline |
| Female | 15 to 21 | 0 | #NUM! | 0 | Baseline |
| Female | 35+ | 0 | #NUM! | 0 | Baseline |
| Male | 15 to 21 | 0 | #NUM! | 0 | Baseline |
| Male | 15 to 21 | 0 | #NUM! | 0 | Baseline |
| Male | 1 to 4 | 0 | #NUM! | 0 | Baseline |
| Female | 1 to 4 | 0 | #NUM! | 0 | Baseline |
| Female | 1 to 4 | 0 | #NUM! | 0 | Baseline |
| Female | 1 to 4 | 0 | #NUM! | 0 | Baseline |
| Female | 1 to 4 | 0 | #NUM! | 0 | Baseline |
| Male | 35+ | 0 | #NUM! | 0 | Baseline |
| Male | 22 to 35 | 0 | #NUM! | 0 | Baseline |
| Female | 5 to 14 | 0 | #NUM! | 0 | Baseline |
| Male | 5 to 14 | 0 | #NUM! | 0 | Baseline |
| Female | 5 to 14 | 0 | #NUM! | 0 | Baseline |
| Male | 5 to 14 | 0 | #NUM! | 0 | Baseline |
| Male | 5 to 14 | 0 | #NUM! | 0 | Baseline |
| Male | 5 to 14 | 0 | #NUM! | 0 | Baseline |
| Female | 5 to 14 | 0 | #NUM! | 0 | Baseline |
| Female | 5 to 14 | 3 | 0 | 0 | Baseline |
| Male | 5 to 14 | 0 | #NUM! | 0 | Baseline |
| Female | 5 to 14 | 0 | #NUM! | 0 | Baseline |
| Male | 5 to 14 | 0 | #NUM! | 0 | Baseline |
| Female | 5 to 14 | 0 | #NUM! | 0 | Baseline |
| Male | 5 to 14 | 0 | #NUM! | 0 | Baseline |

| Female | 5 to 14 | 0 | #NUM! | 0 | Baseline |
| --- | --- | --- | --- | --- | --- |
| Female | 35+ | 0 | #NUM! | 0 | Baseline |
| Male | 22 to 35 | 0 | #NUM! | 0 | Baseline |
| Male | 15 to 21 | 0 | #NUM! | 0 | Baseline |
| Male | 1 to 4 | 0 | #NUM! | 0 | Baseline |
| Female | 1 to 4 | 0 | #NUM! | 0 | Baseline |
| Male | 1 to 4 | 0 | #NUM! | 0 | Baseline |
| Male | 1 to 4 | 0 | #NUM! | 0 | Baseline |
| Male | 1 to 4 | 0 | #NUM! | 0 | Baseline |
| Female | 1 to 4 | 0 | #NUM! | 0 | Baseline |
| Male | 1 to 4 | 0 | #NUM! | 0 | Baseline |
| Female | 1 to 4 | 0 | #NUM! | 0 | Baseline |
| Male | 1 to 4 | 0 | #NUM! | 0 | Baseline |
| Male | 1 to 4 | 0 | #NUM! | 0 | Baseline |
| Male | 35+ | 0 | #NUM! | 0 | Baseline |
| Male | 22 to 35 | 0 | #NUM! | 0 | Baseline |
| Female | 22 to 35 | 0 | #NUM! | 0 | Baseline |
| Male | 35+ | 0 | #NUM! | 0 | Baseline |
| Female | 35+ | 0 | #NUM! | 0 | Baseline |
| Female | 22 to 35 | 0 | #NUM! | 0 | Baseline |
| Male | 35+ | 0 | #NUM! | 0 | Baseline |
| Female | 35+ | 0 | #NUM! | 0 | Baseline |
| Male | 22 to 35 | 0 | #NUM! | 0 | Baseline |
| Female | 35+ | 0 | #NUM! | 0 | Baseline |
| Male | 35+ | 0 | #NUM! | 0 | Baseline |
| Female | 22 to 35 | 0 | #NUM! | 0 | Baseline |
| Female | 15 to 21 | 0 | #NUM! | 0 | Baseline |
| Female | 15 to 21 | 0 | #NUM! | 0 | Baseline |
| Female | 22 to 35 | 0 | #NUM! | 0 | Baseline |
| Male | 15 to 21 | 0 | #NUM! | 0 | Baseline |
| Female | 22 to 35 | 1 | 0 | 0 | Baseline |
| Female | 22 to 35 | 0 | #NUM! | 0 | Baseline |
| Male | 1 to 4 | 0 | #NUM! | 0 | Baseline |
| Female | 1 to 4 | 0 | #NUM! | 0 | Baseline |
| Male | 1 to 4 | 0 | #NUM! | 0 | Baseline |
| Female | 1 to 4 | 0 | #NUM! | 0 | Baseline |
| Female | 5 to 14 | 0 | #NUM! | 0 | Baseline |
| Male | 1 to 4 | 0 | #NUM! | 0 | Baseline |
| Male | 15 to 21 | 0 | #NUM! | 0 | Baseline |
| Female | 1 to 4 | 0 | #NUM! | 0 | Baseline |
| Female | 5 to 14 | 0 | #NUM! | 0 | Baseline |
| Female | 35+ | 0 | #NUM! | 0 | Baseline |
| Male | 22 to 35 | 0 | #NUM! | 0 | Baseline |
| Male | 5 to 14 | 0 | #NUM! | 0 | Baseline |
| Female | 1 to 4 | 0 | #NUM! | 0 | Baseline |
| Male | 22 to 35 | 0 | #NUM! | 0 | Baseline |
| Male | 1 to 4 | 0 | #NUM! | 0 | Baseline |
| Female | 22 to 35 | 0 | #NUM! | 0 | Baseline |
| Female | 5 to 14 | 0 | #NUM! | 0 | Baseline |
| Female | 35+ | 0 | #NUM! | 0 | Baseline |

| Female | 1 to 4 | 0 | #NUM! | 0 | Baseline |
| --- | --- | --- | --- | --- | --- |
| Male | 15 to 21 | 0 | #NUM! | 0 | Baseline |
| Male | 1 to 4 | 0 | #NUM! | 0 | Baseline |
| Male | 1 to 4 | 0 | #NUM! | 0 | Baseline |
| Male | 22 to 35 | 0 | #NUM! | 0 | Baseline |
| Female | 22 to 35 | 0 | #NUM! | 0 | Baseline |
| Male | 35+ | 0 | #NUM! | 0 | Baseline |
| Female | 1 to 4 | 0 | #NUM! | 0 | Baseline |
| Female | 22 to 35 | 0 | #NUM! | 0 | Baseline |
| Female | 22 to 35 | 0 | #NUM! | 0 | Baseline |
| Female | 15 to 21 | 0 | #NUM! | 0 | Baseline |
| Male | 1 to 4 | 0 | #NUM! | 0 | Baseline |
| Male | 1 to 4 | 0 | #NUM! | 0 | Baseline |
| Female | 22 to 35 | 0 | #NUM! | 0 | Baseline |
| Male | 22 to 35 | 0 | #NUM! | 0 | Baseline |
| Male | 5 to 14 | 0 | #NUM! | 0 | Baseline |
| Male | 15 to 21 | 0 | #NUM! | 0 | Baseline |
| Male | 22 to 35 | 0 | #NUM! | 0 | Baseline |
| Female | 1 to 4 | 0 | #NUM! | 0 | Baseline |
| Male | 5 to 14 | 0 | #NUM! | 0 | Baseline |
| Male | 1 to 4 | 0 | #NUM! | 0 | Baseline |
| Male | 1 to 4 | 0 | #NUM! | 0 | Baseline |
| Male | 22 to 35 | 0 | #NUM! | 0 | Baseline |
| Male | 1 to 4 | 0 | #NUM! | 0 | Baseline |
| Female | 22 to 35 | 0 | #NUM! | 0 | Baseline |
| Male | 22 to 35 | 0 | #NUM! | 0 | Baseline |
| Female | 15 to 21 | 0 | #NUM! | 0 | Baseline |
| Female | 22 to 35 | 0 | #NUM! | 0 | Baseline |
| Male | 15 to 21 | 0 | #NUM! | 0 | Baseline |
| Female | 22 to 35 | 0 | #NUM! | 0 | Baseline |
| Male | 1 to 4 | 0 | #NUM! | 0 | Baseline |
| Female | 15 to 21 | 0 | #NUM! | 0 | Baseline |
| Male | 22 to 35 | 0 | #NUM! | 0 | Baseline |
| Female | 22 to 35 | 0 | #NUM! | 0 | Baseline |
| Female | 15 to 21 | 0 | #NUM! | 0 | Baseline |
| Male | 22 to 35 | 0 | #NUM! | 0 | Baseline |
| Male | 1 to 4 | 1 | 0 | 0 | Baseline |
| Male | 5 to 14 | 0 | #NUM! | 0 | Baseline |
| Female | 5 to 14 | 0 | #NUM! | 0 | Baseline |
| Female | 1 to 4 | 1 | 0 | 0 | Baseline |
| Male | 15 to 21 | 0 | #NUM! | 0 | Baseline |
| Female | 22 to 35 | 0 | #NUM! | 0 | Baseline |
| Male | 5 to 14 | 0 | #NUM! | 0 | Baseline |
| Male | 22 to 35 | 0 | #NUM! | 0 | Baseline |
| Male | 35+ | 0 | #NUM! | 0 | Baseline |
| Female | 35+ | 0 | #NUM! | 0 | Baseline |
| Female | 35+ | 1 | 0 | 0 | Baseline |
| Female | 35+ | 0 | #NUM! | 0 | Baseline |
| Female | 35+ | 0 | #NUM! | 0 | Baseline |
| Female | 35+ | 0 | #NUM! | 0 | Baseline |

| Male | 35+ | 0 | #NUM! | 0 | Baseline |
| --- | --- | --- | --- | --- | --- |
| Male | 35+ | 0 | #NUM! | 0 | Baseline |
| Female | 35+ | 0 | #NUM! | 0 | Baseline |
| Male | 35+ | 0 | #NUM! | 0 | Baseline |
| Male | 35+ | 0 | #NUM! | 0 | Baseline |
| Male | 22 to 35 | 0 | #NUM! | 0 | Baseline |
| Female | 1 to 4 | 0 | #NUM! | 0 | Baseline |
| Female | 5 to 14 | 0 | #NUM! | 0 | Baseline |
| Male | 5 to 14 | 0 | #NUM! | 0 | Baseline |
| Male | 5 to 14 | 0 | #NUM! | 0 | Baseline |
| Male | 5 to 14 | 0 | #NUM! | 0 | Baseline |
| Female | 5 to 14 | 0 | #NUM! | 0 | Baseline |
| Male | 5 to 14 | 0 | #NUM! | 0 | Baseline |
| Male | 1 to 4 | 0 | #NUM! | 0 | Baseline |
| Female | 5 to 14 | 0 | #NUM! | 0 | Baseline |
| Male | 5 to 14 | 0 | #NUM! | 0 | Baseline |
| Male | 5 to 14 | 0 | #NUM! | 0 | Baseline |
| Male | 5 to 14 | 0 | #NUM! | 0 | Baseline |
| Female | 5 to 14 | 0 | #NUM! | 0 | Baseline |
| Female | 5 to 14 | 0 | #NUM! | 0 | Baseline |
| Female | 5 to 14 | 0 | #NUM! | 0 | Baseline |
| Female | 22 to 35 | 0 | #NUM! | 0 | Baseline |
| Male | 15 to 21 | 0 | #NUM! | 0 | Baseline |
| Male | 5 to 14 | 0 | #NUM! | 0 | Baseline |
| Male | 5 to 14 | 0 | #NUM! | 0 | Baseline |
| Female | 5 to 14 | 0 | #NUM! | 0 | Baseline |
| Male | 1 to 4 | 0 | #NUM! | 0 | Baseline |
| Female | 5 to 14 | 0 | #NUM! | 0 | Baseline |
| Male | 15 to 21 | 0 | #NUM! | 0 | Baseline |
| Female | 15 to 21 | 0 | #NUM! | 0 | Baseline |
| Female | 15 to 21 | 0 | #NUM! | 0 | Baseline |
| Male | 15 to 21 | 0 | #NUM! | 0 | Baseline |
| Female | 15 to 21 | 0 | #NUM! | 0 | Baseline |
| Male | 15 to 21 | 0 | #NUM! | 0 | Baseline |
| Male | 1 to 4 | 0 | #NUM! | 0 | Baseline |
| Female | 1 to 4 | 0 | #NUM! | 0 | Baseline |
| Female | 1 to 4 | 0 | #NUM! | 0 | Baseline |
| Male | 1 to 4 | 0 | #NUM! | 0 | Baseline |
| Female | 1 to 4 | 0 | #NUM! | 0 | Baseline |
| Female | 15 to 21 | 0 | #NUM! | 0 | Baseline |
| Female | 22 to 35 | 0 | #NUM! | 0 | Baseline |
| Female | 22 to 35 | 0 | #NUM! | 0 | Baseline |
| Female | 15 to 21 | 0 | #NUM! | 0 | Baseline |
| Female | 22 to 35 | 0 | #NUM! | 0 | Baseline |
| Female | 35+ | 0 | #NUM! | 0 | Baseline |
| Male | 35+ | 0 | #NUM! | 0 | Baseline |
| Male | 1 to 4 | 0 | #NUM! | 0 | Baseline |
| Male | 1 to 4 | 0 | #NUM! | 0 | Baseline |
| Female | 35+ | 0 | #NUM! | 0 | Baseline |
| Male | 22 to 35 | 0 | #NUM! | 0 | Baseline |

| Male | 35+ | 0 | #NUM! | 0 | Baseline |
| --- | --- | --- | --- | --- | --- |
| Female | 35+ | 0 | #NUM! | 0 | Baseline |
| Female | 35+ | 0 | #NUM! | 0 | Baseline |
| Male | 35+ | 0 | #NUM! | 0 | Baseline |
| Male | 1 to 4 | 0 | #NUM! | 0 | Baseline |
| Male | 35+ | 0 | #NUM! | 0 | Baseline |
| Male | 22 to 35 | 0 | #NUM! | 0 | Baseline |
| Male | 15 to 21 | 0 | #NUM! | 0 | Baseline |
| Female | 35+ | 0 | #NUM! | 0 | Baseline |
| Male | 35+ | 0 | #NUM! | 0 | Baseline |
| Female | 35+ | 0 | #NUM! | 0 | Baseline |
| Male | 5 to 14 | 0 | #NUM! | 0 | Baseline |
| Male | 35+ | 0 | #NUM! | 0 | Baseline |
| Female | 1 to 4 | 0 | #NUM! | 0 | Baseline |
| Female | 1 to 4 | 0 | #NUM! | 0 | Baseline |
| Male | 1 to 4 | 0 | #NUM! | 0 | Baseline |
| Male | 15 to 21 | 0 | #NUM! | 0 | Baseline |
| Female | 22 to 35 | 0 | #NUM! | 0 | Baseline |
| Female | 1 to 4 | 0 | #NUM! | 0 | Baseline |
| Female | 15 to 21 | 0 | #NUM! | 0 | Baseline |
| Male | 1 to 4 | 0 | #NUM! | 0 | Baseline |
| Female | 22 to 35 | 0 | #NUM! | 0 | Baseline |
| Female | 22 to 35 | 0 | #NUM! | 0 | Baseline |
| Male | 22 to 35 | 0 | #NUM! | 0 | Baseline |
| Male | 15 to 21 | 0 | #NUM! | 0 | Baseline |
| Male | 15 to 21 | 0 | #NUM! | 0 | Baseline |
| Male | 22 to 35 | 0 | #NUM! | 0 | Baseline |
| Female | 22 to 35 | 0 | #NUM! | 0 | Baseline |
| Male | 35+ | 0 | #NUM! | 0 | Baseline |
| Male | 35+ | 0 | #NUM! | 0 | Baseline |
| Female | 35+ | 0 | #NUM! | 0 | Baseline |
| Female | 1 to 4 | 0 | #NUM! | 0 | Baseline |
| Female | 22 to 35 | 0 | #NUM! | 0 | Baseline |
| Female | 15 to 21 | 0 | #NUM! | 0 | Baseline |
| Female | 22 to 35 | 0 | #NUM! | 0 | Baseline |
| Female | 22 to 35 | 0 | #NUM! | 0 | Baseline |
| Male | 22 to 35 | 0 | #NUM! | 0 | Baseline |
| Male | 15 to 21 | 0 | #NUM! | 0 | Baseline |
| Female | 22 to 35 | 0 | #NUM! | 0 | Baseline |
| Male | 22 to 35 | 0 | #NUM! | 0 | Baseline |
| Female | 35+ | 0 | #NUM! | 0 | Baseline |
| Male | 15 to 21 | 0 | #NUM! | 0 | Baseline |
| Male | 15 to 21 | 0 | #NUM! | 0 | Baseline |
| Male | 15 to 21 | 0 | #NUM! | 0 | Baseline |
| Female | 15 to 21 | 0 | #NUM! | 0 | Baseline |
| Male | 5 to 14 | 0 | #NUM! | 0 | Baseline |
| Male | 1 to 4 | 0 | #NUM! | 0 | Baseline |
| Female | 35+ | 0 | #NUM! | 0 | Baseline |
| Male | 5 to 14 | 0 | #NUM! | 0 | Baseline |
| Male | 22 to 35 | 0 | #NUM! | 0 | Baseline |

| Male | 35+ | 0 | #NUM! | 0 | Baseline |
| --- | --- | --- | --- | --- | --- |
| Male | 35+ | 0 | #NUM! | 0 | Baseline |
| Female | 35+ | 0 | #NUM! | 0 | Baseline |
| Female | 22 to 35 | 0 | #NUM! | 0 | Baseline |
| Male | 5 to 14 | 0 | #NUM! | 0 | Baseline |
| Female | 5 to 14 | 0 | #NUM! | 0 | Baseline |
| Male | 15 to 21 | 0 | #NUM! | 0 | Baseline |
| Male | 5 to 14 | 0 | #NUM! | 0 | Baseline |
| Female | 5 to 14 | 0 | #NUM! | 0 | Baseline |
| Male | 15 to 21 | 0 | #NUM! | 0 | Baseline |
| Male | 5 to 14 | 0 | #NUM! | 0 | Baseline |
| Male | 5 to 14 | 0 | #NUM! | 0 | Baseline |
| Male | 15 to 21 | 0 | #NUM! | 0 | Baseline |
| Male | 5 to 14 | 0 | #NUM! | 0 | Baseline |
| Female | 1 to 4 | 0 | #NUM! | 0 | Baseline |
| Female | 35+ | 0 | #NUM! | 0 | Baseline |
| Male | 15 to 21 | 0 | #NUM! | 0 | Baseline |
| Female | 5 to 14 | 0 | #NUM! | 0 | Baseline |
| Female | 5 to 14 | 0 | #NUM! | 0 | Baseline |
| Female | 22 to 35 | 0 | #NUM! | 0 | Baseline |
| Female | 35+ | 0 | #NUM! | 0 | Baseline |
| Male | 22 to 35 | 0 | #NUM! | 0 | Baseline |
| Male | 22 to 35 | 0 | #NUM! | 0 | Baseline |
| Male | 22 to 35 | 0 | #NUM! | 0 | Baseline |
| Male | 22 to 35 | 0 | #NUM! | 0 | Baseline |
| Male | 35+ | 0 | #NUM! | 0 | Baseline |
| Female | 35+ | 0 | #NUM! | 0 | Baseline |
| Female | 22 to 35 | 0 | #NUM! | 0 | Baseline |
| Male | 15 to 21 | 0 | #NUM! | 0 | Baseline |
| Female | 22 to 35 | 0 | #NUM! | 0 | Baseline |
| Male | 35+ | 0 | #NUM! | 0 | Baseline |
| Female | 22 to 35 | 0 | #NUM! | 0 | Baseline |
| Male | 22 to 35 | 0 | #NUM! | 0 | Baseline |
| Female | 35+ | 0 | #NUM! | 0 | Baseline |
| Female | 35+ | 0 | #NUM! | 0 | Baseline |
| Male | 15 to 21 | 0 | #NUM! | 0 | Baseline |
| Female | 15 to 21 | 0 | #NUM! | 0 | Baseline |
| Male | 15 to 21 | 0 | #NUM! | 0 | Baseline |
| Male | 15 to 21 | 0 | #NUM! | 0 | Baseline |
| Female | 15 to 21 | 0 | #NUM! | 0 | Baseline |
| Female | 5 to 14 | 0 | #NUM! | 0 | Baseline |
| Female | 22 to 35 | 0 | #NUM! | 0 | Baseline |
| Female | 5 to 14 | 0 | #NUM! | 0 | Baseline |
| Female | 1 to 4 | 0 | #NUM! | 0 | Baseline |
| Female | 1 to 4 | 0 | #NUM! | 0 | Baseline |
| Female | 1 to 4 | 0 | #NUM! | 0 | Baseline |
| Male | 5 to 14 | 0 | #NUM! | 0 | Baseline |
| Male | 5 to 14 | 0 | #NUM! | 0 | Baseline |
| Male | 1 to 4 | 0 | #NUM! | 0 | Baseline |
| Male | 5 to 14 | 0 | #NUM! | 0 | Baseline |

| Male | 1 to 4 | 0 | #NUM! | 0 | Baseline |
| --- | --- | --- | --- | --- | --- |
| Male | 5 to 14 | 0 | #NUM! | 0 | Baseline |
| Female | 5 to 14 | 0 | #NUM! | 0 | Baseline |
| Female | 5 to 14 | 0 | #NUM! | 0 | Baseline |
| Male | 35+ | 0 | #NUM! | 0 | Baseline |
| Male | 22 to 35 | 0 | #NUM! | 0 | Baseline |
| Male | 35+ | 0 | #NUM! | 0 | Baseline |
| Male | 35+ | 0 | #NUM! | 0 | Baseline |
| Female | 15 to 21 | 0 | #NUM! | 0 | Baseline |
| Female | 15 to 21 | 0 | #NUM! | 0 | Baseline |
| Male | 15 to 21 | 0 | #NUM! | 0 | Baseline |
| Female | 15 to 21 | 0 | #NUM! | 0 | Baseline |
| Male | 15 to 21 | 0 | #NUM! | 0 | Baseline |
| Male | 15 to 21 | 0 | #NUM! | 0 | Baseline |
| Male | 5 to 14 | 0 | #NUM! | 0 | Baseline |
| Male | 15 to 21 | 0 | #NUM! | 0 | Baseline |
| Male | 5 to 14 | 0 | #NUM! | 0 | Baseline |
| Female | 22 to 35 | 0 | #NUM! | 0 | Baseline |
| Female | 15 to 21 | 0 | #NUM! | 0 | Baseline |
| Male | 5 to 14 | 0 | #NUM! | 0 | Baseline |
| Female | 5 to 14 | 0 | #NUM! | 0 | Baseline |
| Male | 22 to 35 | 0 | #NUM! | 0 | Baseline |
| Male | 1 to 4 | 0 | #NUM! | 0 | Baseline |
| Female | 15 to 21 | 0 | #NUM! | 0 | Baseline |
| Female | 5 to 14 | 0 | #NUM! | 0 | Baseline |
| Female | 35+ | 0 | #NUM! | 0 | Baseline |
| Female | 1 to 4 | 0 | #NUM! | 0 | Baseline |
| Male | 1 to 4 | 0 | #NUM! | 0 | Baseline |
| Female | 22 to 35 | 0 | #NUM! | 0 | Baseline |
| Male | 5 to 14 | 0 | #NUM! | 0 | Baseline |
| Female | 35+ | 0 | #NUM! | 0 | Baseline |
| Male | 22 to 35 | 0 | #NUM! | 0 | Baseline |
| Male | 15 to 21 | 0 | #NUM! | 0 | Baseline |
| Female | 22 to 35 | 0 | #NUM! | 0 | Baseline |
| Male | 22 to 35 | 0 | #NUM! | 0 | Baseline |
| Female | 15 to 21 | 0 | #NUM! | 0 | Baseline |
| Female | 1 to 4 | 0 | #NUM! | 0 | Baseline |
| Male | 1 to 4 | 0 | #NUM! | 0 | Baseline |
| Male | 1 to 4 | 0 | #NUM! | 0 | Baseline |
| Female | 1 to 4 | 0 | #NUM! | 0 | Baseline |
| Male | 1 to 4 | 0 | #NUM! | 0 | Baseline |
| Male | 1 to 4 | 0 | #NUM! | 0 | Baseline |
| Female | 15 to 21 | 0 | #NUM! | 0 | Baseline |
| Female | 15 to 21 | 0 | #NUM! | 0 | Baseline |
| Male | 15 to 21 | 0 | #NUM! | 0 | Baseline |
| Male | 15 to 21 | 3 | 0 | 0 | Baseline |
| Male | 1 to 4 | 0 | #NUM! | 0 | Baseline |
| Female | 15 to 21 | 0 | #NUM! | 0 | Baseline |
| Female | 15 to 21 | 0 | #NUM! | 0 | Baseline |
| Female | 5 to 14 | 0 | #NUM! | 0 | Baseline |

| Male | 15 to 21 | 0 | #NUM! | 0 | Baseline |
| --- | --- | --- | --- | --- | --- |
| Male | 5 to 14 | 0 | #NUM! | 0 | Baseline |
| Female | 1 to 4 | 0 | #NUM! | 0 | Baseline |
| Male | 1 to 4 | 0 | #NUM! | 0 | Baseline |
| Male | 1 to 4 | 0 | #NUM! | 0 | Baseline |
| Female | 1 to 4 | 0 | #NUM! | 0 | Baseline |
| Male | 1 to 4 | 0 | #NUM! | 0 | Baseline |
| Female | 1 to 4 | 0 | #NUM! | 0 | Baseline |
| Male | 5 to 14 | 0 | #NUM! | 0 | Baseline |
| Female | 5 to 14 | 0 | #NUM! | 0 | Baseline |
| Male | 5 to 14 | 0 | #NUM! | 0 | Baseline |
| Male | 1 to 4 | 0 | #NUM! | 0 | Baseline |
| Male | 1 to 4 | 0 | #NUM! | 0 | Baseline |
| Male | 5 to 14 | 0 | #NUM! | 0 | Baseline |
| Male | 5 to 14 | 0 | #NUM! | 0 | Baseline |
| Male | 15 to 21 | 0 | #NUM! | 0 | Baseline |
| Male | 15 to 21 | 0 | #NUM! | 0 | Baseline |
| Female | 1 to 4 | 0 | #NUM! | 0 | Baseline |
| Female | 1 to 4 | 0 | #NUM! | 0 | Baseline |
| Female | 1 to 4 | 0 | #NUM! | 0 | Baseline |
| Male | 1 to 4 | 0 | #NUM! | 0 | Baseline |
| Male | 5 to 14 | 0 | #NUM! | 0 | Baseline |
| Female | 22 to 35 | 0 | #NUM! | 0 | Baseline |
| Male | 1 to 4 | 0 | #NUM! | 0 | Baseline |
| Male | 5 to 14 | 0 | #NUM! | 0 | Baseline |
| Male | 1 to 4 | 0 | #NUM! | 0 | Baseline |
| Male | 1 to 4 | 0 | #NUM! | 0 | Baseline |
| Female | 1 to 4 | 0 | #NUM! | 0 | Baseline |
| Male | 22 to 35 | 0 | #NUM! | 0 | Baseline |
| Male | 22 to 35 | 0 | #NUM! | 0 | Baseline |
| Male | 35+ | 0 | #NUM! | 0 | Baseline |
| Female | 35+ | 0 | #NUM! | 0 | Baseline |
| Male | 35+ | 0 | #NUM! | 0 | Baseline |
| Female | 15 to 21 | 0 | #NUM! | 0 | Baseline |
| Female | 22 to 35 | 0 | #NUM! | 0 | Baseline |
| Male | 22 to 35 | 0 | #NUM! | 0 | Baseline |
| Female | 22 to 35 | 0 | #NUM! | 0 | Baseline |
| Female | 22 to 35 | 0 | #NUM! | 0 | Baseline |
| Male | 35+ | 0 | #NUM! | 0 | Baseline |
| Male | 22 to 35 | 0 | #NUM! | 0 | Baseline |
| Male | 35+ | 0 | #NUM! | 0 | Baseline |
| Female | 22 to 35 | 0 | #NUM! | 0 | Baseline |
| Female | 22 to 35 | 0 | #NUM! | 0 | Baseline |
| Female | 22 to 35 | 0 | #NUM! | 0 | Baseline |
| Female | 22 to 35 | 0 | #NUM! | 0 | Baseline |
| Male | 35+ | 0 | #NUM! | 0 | Baseline |
| Male | 35+ | 0 | #NUM! | 0 | Baseline |
| Female | 35+ | 0 | #NUM! | 0 | Baseline |
| Female | 1 to 4 | 0 | #NUM! | 0 | Baseline |
| Male | 22 to 35 | 0 | #NUM! | 0 | Baseline |

| Male | 35+ | 5 | 0 | 0 | Baseline |
| --- | --- | --- | --- | --- | --- |
| Male | 35+ | 0 | #NUM! | 0 | Baseline |
| Female | 15 to 21 | 0 | #NUM! | 0 | Baseline |
| Male | 35+ | 0 | #NUM! | 0 | Baseline |
| Female | 35+ | 0 | #NUM! | 0 | Baseline |
| Male | 22 to 35 | 0 | #NUM! | 0 | Baseline |
| Male | 22 to 35 | 0 | #NUM! | 0 | Baseline |
| Female | 22 to 35 | 0 | #NUM! | 0 | Baseline |
| Female | 22 to 35 | 0 | #NUM! | 0 | Baseline |
| Male | 1 to 4 | 0 | #NUM! | 0 | Baseline |
| Male | 15 to 21 | 0 | #NUM! | 0 | Baseline |
| Female | 35+ | 0 | #NUM! | 0 | Baseline |
| Male | 1 to 4 | 0 | #NUM! | 0 | Baseline |
| Male | 22 to 35 | 0 | #NUM! | 0 | Baseline |
| Male | 1 to 4 | 0 | #NUM! | 0 | Baseline |
| Female | 1 to 4 | 0 | #NUM! | 0 | Baseline |
| Female | 22 to 35 | 0 | #NUM! | 0 | Baseline |
| Male | 5 to 14 | 0 | #NUM! | 0 | Baseline |
| Male | 1 to 4 | 0 | #NUM! | 0 | Baseline |
| Male | 22 to 35 | 0 | #NUM! | 0 | Baseline |
| Male | 22 to 35 | 0 | #NUM! | 0 | Baseline |
| Female | 15 to 21 | 0 | #NUM! | 0 | Baseline |
| Male |  | 0 | #NUM! | 0 | Baseline |
| Female | 1 to 4 | 0 | #NUM! | 0 | Baseline |
| Male | 22 to 35 | 0 | #NUM! | 0 | Baseline |
| Male | 1 to 4 | 0 | #NUM! | 0 | Baseline |
| Female | 1 to 4 | 0 | #NUM! | 0 | Baseline |
| Male | 1 to 4 | 3 | 0 | 0 | Baseline |
| Male | 1 to 4 | 0 | #NUM! | 0 | Baseline |
| Male | 1 to 4 | 0 | #NUM! | 0 | Baseline |
| Male | 35+ | 0 | #NUM! | 0 | Baseline |
| Male | 35+ | 0 | #NUM! | 0 | Baseline |
| Male | 22 to 35 | 0 | #NUM! | 0 | Baseline |
| Female | 22 to 35 | 0 | #NUM! | 0 | Baseline |
| Male | 1 to 4 | 0 | #NUM! | 0 | Baseline |
| Female | 1 to 4 | 0 | #NUM! | 0 | Baseline |
| Female | 1 to 4 | 0 | #NUM! | 0 | Baseline |
| Male | 1 to 4 | 0 | #NUM! | 0 | Baseline |
| Male | 1 to 4 | 0 | #NUM! | 0 | Baseline |
| Female | 1 to 4 | 0 | #NUM! | 0 | Baseline |
| Female | 1 to 4 | 0 | #NUM! | 0 | Baseline |
| Female | 35+ | 0 | #NUM! | 0 | Baseline |
| Male | 35+ | 0 | #NUM! | 0 | Baseline |
| Male | 22 to 35 | 0 | #NUM! | 0 | Baseline |
| Female | 22 to 35 | 0 | #NUM! | 0 | Baseline |
| Male | 35+ | 0 | #NUM! | 0 | Baseline |
| Female | 35+ | 0 | #NUM! | 0 | Baseline |
| Female | 22 to 35 | 0 | #NUM! | 0 | Baseline |
| Female | 22 to 35 | 0 | #NUM! | 0 | Baseline |
| Female | 22 to 35 | 0 | #NUM! | 0 | Baseline |

| Female | 5 to 14 | 0 | #NUM! | 0 | Baseline |
| --- | --- | --- | --- | --- | --- |
| Male | 35+ | 0 | #NUM! | 0 | Baseline |
| Female | 22 to 35 | 0 | #NUM! | 0 | Baseline |
| Female | 35+ | 0 | #NUM! | 0 | Baseline |
| Female | 22 to 35 | 1 | 0 | 0 | Baseline |
| Male | 22 to 35 | 0 | #NUM! | 0 | Baseline |
| Male | 1 to 4 | 0 | #NUM! | 0 | Baseline |
| Female | 22 to 35 | 0 | #NUM! | 0 | Baseline |
| Male | 22 to 35 | 0 | #NUM! | 0 | Baseline |
| Male | 1 to 4 | 0 | #NUM! | 0 | Baseline |
| Male | 22 to 35 | 0 | #NUM! | 0 | Baseline |
| Female | 22 to 35 | 0 | #NUM! | 0 | Baseline |
| Male | 35+ | 0 | #NUM! | 0 | Baseline |
| Male | 35+ | 0 | #NUM! | 0 | Baseline |
| Male | 35+ | 0 | #NUM! | 0 | Baseline |
| Male | 15 to 21 | 0 | #NUM! | 0 | Baseline |
| Male | 35+ | 0 | #NUM! | 0 | Baseline |
| Male | 5 to 14 | 0 | #NUM! | 0 | Baseline |
| Female | 35+ | 0 | #NUM! | 0 | Baseline |
| Female | 35+ | 0 | #NUM! | 0 | Baseline |
| Female | 15 to 21 | 0 | #NUM! | 0 | Baseline |
| Female | 15 to 21 | 0 | #NUM! | 0 | Baseline |
| Male | 22 to 35 | 0 | #NUM! | 0 | Baseline |
| Female | 22 to 35 | 0 | #NUM! | 0 | Baseline |
| Male | 22 to 35 | 0 | #NUM! | 0 | Baseline |
| Male | 35+ | 0 | #NUM! | 0 | Baseline |
| Male | 22 to 35 | 0 | #NUM! | 0 | Baseline |
| Male | 15 to 21 | 0 | #NUM! | 0 | Baseline |
| Female | 35+ | 0 | #NUM! | 0 | Baseline |
| Female | 15 to 21 | 0 | #NUM! | 0 | Baseline |
| Male | 15 to 21 | 0 | #NUM! | 0 | Baseline |
| Female | 5 to 14 | 0 | #NUM! | 0 | Baseline |
| Male | 35+ | 0 | #NUM! | 0 | Baseline |
| Female | 15 to 21 | 0 | #NUM! | 0 | Baseline |
| Female | 35+ | 0 | #NUM! | 0 | Baseline |
| Female | 22 to 35 | 0 | #NUM! | 0 | Baseline |
| Female | 15 to 21 | 0 | #NUM! | 0 | Baseline |
| Female | 22 to 35 | 0 | #NUM! | 0 | Baseline |
| Female | 15 to 21 | 0 | #NUM! | 0 | Baseline |
| Male | 15 to 21 | 0 | #NUM! | 0 | Baseline |
| Male | 15 to 21 | 0 | #NUM! | 0 | Baseline |
| Male | 5 to 14 | 0 | #NUM! | 0 | Baseline |
| Female | 5 to 14 | 0 | #NUM! | 0 | Baseline |
| Male | 22 to 35 | 0 | #NUM! | 0 | Baseline |
| Male | 5 to 14 | 0 | #NUM! | 0 | Baseline |
| Female | 5 to 14 | 0 | #NUM! | 0 | Baseline |
| Female | 5 to 14 | 0 | #NUM! | 0 | Baseline |
| Male | 22 to 35 | 0 | #NUM! | 0 | Baseline |
| Male | 5 to 14 | 0 | #NUM! | 0 | Baseline |
| Male | 5 to 14 | 0 | #NUM! | 0 | Baseline |

| Male | 35+ | 0 | #NUM! | 0 | Baseline |
| --- | --- | --- | --- | --- | --- |
| Male | 5 to 14 | 0 | #NUM! | 0 | Baseline |
| Male | 5 to 14 | 0 | #NUM! | 0 | Baseline |
| Male | 5 to 14 | 0 | #NUM! | 0 | Baseline |
| Female | 22 to 35 | 0 | #NUM! | 0 | Baseline |
| Male | 22 to 35 | 0 | #NUM! | 0 | Baseline |
| Male | 22 to 35 | 0 | #NUM! | 0 | Baseline |
| Male | 22 to 35 | 0 | #NUM! | 0 | Baseline |
| Female | 5 to 14 | 0 | #NUM! | 0 | Baseline |
| Male | 5 to 14 | 0 | #NUM! | 0 | Baseline |
| Male | 22 to 35 | 0 | #NUM! | 0 | Baseline |
| Male | 5 to 14 | 0 | #NUM! | 0 | Baseline |
| Male | 15 to 21 | 0 | #NUM! | 0 | Baseline |
| Male | 5 to 14 | 0 | #NUM! | 0 | Baseline |
| Male | 5 to 14 | 0 | #NUM! | 0 | Baseline |
| Male | 5 to 14 | 0 | #NUM! | 0 | Baseline |
| Male | 15 to 21 | 0 | #NUM! | 0 | Baseline |
| Male | 35+ | 0 | #NUM! | 0 | Baseline |
| Female | 5 to 14 | 0 | #NUM! | 0 | Baseline |
| Female | 5 to 14 | 0 | #NUM! | 0 | Baseline |
| Male | 22 to 35 | 0 | #NUM! | 0 | Baseline |
| Male | 35+ | 0 | #NUM! | 0 | Baseline |
| Male | 15 to 21 | 0 | #NUM! | 0 | Baseline |
| Male | 35+ | 0 | #NUM! | 0 | Baseline |
| Male | 22 to 35 | 0 | #NUM! | 0 | Baseline |
| Male | 22 to 35 | 0 | #NUM! | 0 | Baseline |
| Male | 22 to 35 | 0 | #NUM! | 0 | Baseline |
| Male | 35+ | 0 | #NUM! | 0 | Baseline |
| Male | 35+ | 0 | #NUM! | 0 | Baseline |
| Female | 22 to 35 | 0 | #NUM! | 0 | Baseline |
| Female | 22 to 35 | 0 | #NUM! | 0 | Baseline |
| Female | 35+ | 0 | #NUM! | 0 | Baseline |
| Female | 35+ | 0 | #NUM! | 0 | Baseline |
| Female | 35+ | 0 | #NUM! | 0 | Baseline |
| Female | 22 to 35 | 0 | #NUM! | 0 | Baseline |
| Female | 22 to 35 | 0 | #NUM! | 0 | Baseline |
| Female | 35+ | 0 | #NUM! | 0 | Baseline |
| Female | 22 to 35 | 0 | #NUM! | 0 | Baseline |
| Female | 22 to 35 | 0 | #NUM! | 0 | Baseline |
| Male | 5 to 14 | 0 | #NUM! | 0 | Baseline |
| Male | 5 to 14 | 0 | #NUM! | 0 | Baseline |
| Male | 5 to 14 | 0 | #NUM! | 0 | Baseline |
| Male | 5 to 14 | 0 | #NUM! | 0 | Baseline |
| Male | 5 to 14 | 0 | #NUM! | 0 | Baseline |
| Male | 5 to 14 | 0 | #NUM! | 0 | Baseline |
| Female | 5 to 14 | 0 | #NUM! | 0 | Baseline |
| Female | 5 to 14 | 0 | #NUM! | 0 | Baseline |
| Female | 15 to 21 | 0 | #NUM! | 0 | Baseline |
| Female | 15 to 21 | 0 | #NUM! | 0 | Baseline |
| Female | 5 to 14 | 0 | #NUM! | 0 | Baseline |

| Female | 5 to 14 | 0 | #NUM! | 0 | Baseline |
| --- | --- | --- | --- | --- | --- |
| Female | 5 to 14 | 0 | #NUM! | 0 | Baseline |
| Female | 5 to 14 | 0 | #NUM! | 0 | Baseline |
| Female | 5 to 14 | 0 | #NUM! | 0 | Baseline |
| Female | 5 to 14 | 0 | #NUM! | 0 | Baseline |
| Female | 15 to 21 | 0 | #NUM! | 0 | Baseline |
| Female | 5 to 14 | 0 | #NUM! | 0 | Baseline |
| Female | 5 to 14 | 0 | #NUM! | 0 | Baseline |
| Female | 5 to 14 | 0 | #NUM! | 0 | Baseline |
| Male | 5 to 14 | 0 | #NUM! | 0 | Baseline |
| Female | 35+ | 0 | #NUM! | 0 | Baseline |
| Male | 22 to 35 | 0 | #NUM! | 0 | Baseline |
| Male | 35+ | 0 | #NUM! | 0 | Baseline |
| Female | 22 to 35 | 0 | #NUM! | 0 | Baseline |
| Female | 35+ | 0 | #NUM! | 0 | Baseline |
| Female | 22 to 35 | 0 | #NUM! | 0 | Baseline |
| Female | 15 to 21 | 0 | #NUM! | 0 | Baseline |
| Male | 22 to 35 | 0 | #NUM! | 0 | Baseline |
| Male | 22 to 35 | 0 | #NUM! | 0 | Baseline |
| Male | 22 to 35 | 0 | #NUM! | 0 | Baseline |
| Male | 5 to 14 | 0 | #NUM! | 0 | Baseline |
| Male | 35+ | 0 | #NUM! | 0 | Baseline |
| Male | 22 to 35 | 0 | #NUM! | 0 | Baseline |
| Male | 35+ | 0 | #NUM! | 0 | Baseline |
| Male | 1 to 4 | 0 | #NUM! | 0 | Baseline |
| Female | 22 to 35 | 0 | #NUM! | 0 | Baseline |
| Male | 35+ | 0 | #NUM! | 0 | Baseline |
| Male | 22 to 35 | 0 | #NUM! | 0 | Baseline |
| Male | 15 to 21 | 0 | #NUM! | 0 | Baseline |
| Female | 22 to 35 | 0 | #NUM! | 0 | Baseline |
| Female | 35+ | 0 | #NUM! | 0 | Baseline |
| Male | 1 to 4 | 0 | #NUM! | 0 | Baseline |
| Male | 5 to 14 | 0 | #NUM! | 0 | Baseline |
| Male | 1 to 4 | 0 | #NUM! | 0 | Baseline |
| Male | 35+ | 0 | #NUM! | 0 | Baseline |
| Female | 15 to 21 | 0 | #NUM! | 0 | Baseline |
| Male | 1 to 4 | 0 | #NUM! | 0 | Baseline |
| Female | 35+ | 0 | #NUM! | 0 | Baseline |
| Male | 1 to 4 | 0 | #NUM! | 0 | Baseline |
| Male | 1 to 4 | 0 | #NUM! | 0 | Baseline |
| Male | 1 to 4 | 0 | #NUM! | 0 | Baseline |
| Female | 22 to 35 | 0 | #NUM! | 0 | Baseline |
| Male | 1 to 4 | 0 | #NUM! | 0 | Baseline |
| Male | 22 to 35 | 0 | #NUM! | 0 | Baseline |
| Male | 1 to 4 | 0 | #NUM! | 0 | Baseline |
| Male | 1 to 4 | 0 | #NUM! | 0 | Baseline |
| Male | 35+ | 0 | #NUM! | 0 | Baseline |
| Female | 15 to 21 | 0 | #NUM! | 0 | Baseline |
| Female | 5 to 14 | 0 | #NUM! | 0 | Baseline |
| Male | 1 to 4 | 0 | #NUM! | 0 | Baseline |

| Male | 1 to 4 | 0 | #NUM! | 0 | Baseline |
| --- | --- | --- | --- | --- | --- |
| Female | 22 to 35 | 0 | #NUM! | 0 | Baseline |
| Male | 35+ | 0 | #NUM! | 0 | Baseline |
| Female | 1 to 4 | 0 | #NUM! | 0 | Baseline |
| Female | 22 to 35 | 0 | #NUM! | 0 | Baseline |
| Female | 5 to 14 | 0 | #NUM! | 0 | Baseline |
| Male | 5 to 14 | 0 | #NUM! | 0 | Baseline |
| Male | 22 to 35 | 0 | #NUM! | 0 | Baseline |
| Female | 5 to 14 | 0 | #NUM! | 0 | Baseline |
| Male | 1 to 4 | 0 | #NUM! | 0 | Baseline |
| Female | 35+ | 0 | #NUM! | 0 | Baseline |
| Male | 35+ | 0 | #NUM! | 0 | Baseline |
| Male | 35+ | 0 | #NUM! | 0 | Baseline |
| Male | 1 to 4 | 0 | #NUM! | 0 | Baseline |
| Male | 1 to 4 | 0 | #NUM! | 0 | Baseline |
| Male | 1 to 4 | 0 | #NUM! | 0 | Baseline |
| Female | 15 to 21 | 0 | #NUM! | 0 | Baseline |
| Female | 1 to 4 | 0 | #NUM! | 0 | Baseline |
| Female | 22 to 35 | 0 | #NUM! | 0 | Baseline |
| Female | 22 to 35 | 0 | #NUM! | 0 | Baseline |
| Female | 22 to 35 | 0 | #NUM! | 0 | Baseline |
| Male | 15 to 21 | 0 | #NUM! | 0 | Baseline |
| Male | 1 to 4 | 0 | #NUM! | 0 | Baseline |
| Female | 22 to 35 | 0 | #NUM! | 0 | Baseline |
| Female | 15 to 21 | 0 | #NUM! | 0 | Baseline |
| Male | 1 to 4 | 0 | #NUM! | 0 | Baseline |
| Male | 22 to 35 | 0 | #NUM! | 0 | Baseline |
| Male | 15 to 21 | 0 | #NUM! | 0 | Baseline |
| Male | 1 to 4 | 0 | #NUM! | 0 | Baseline |
| Female | 1 to 4 | 0 | #NUM! | 0 | Baseline |
| Male | 1 to 4 | 0 | #NUM! | 0 | Baseline |
| Male | 22 to 35 | 0 | #NUM! | 0 | Baseline |
| Female | 15 to 21 | 0 | #NUM! | 0 | Baseline |
| Male | 1 to 4 | 0 | #NUM! | 0 | Baseline |
| Male | 1 to 4 | 0 | #NUM! | 0 | Baseline |
| Male | 1 to 4 | 0 | #NUM! | 0 | Baseline |
| Male | 1 to 4 | 0 | #NUM! | 0 | Baseline |
| Male | 1 to 4 | 0 | #NUM! | 0 | Baseline |
| Male | 1 to 4 | 0 | #NUM! | 0 | Baseline |
| Male | 1 to 4 | 0 | #NUM! | 0 | Baseline |
| Male | 1 to 4 | 0 | #NUM! | 0 | Baseline |
| Male | 1 to 4 | 0 | #NUM! | 0 | Baseline |
| Male | 1 to 4 | 0 | #NUM! | 0 | Baseline |
| Male | 1 to 4 | 0 | #NUM! | 0 | Baseline |
| Male | 1 to 4 | 0 | #NUM! | 0 | Baseline |
| Male | 35+ | 0 | #NUM! | 0 | Baseline |
| Male | 1 to 4 | 0 | #NUM! | 0 | Baseline |
| Male | 1 to 4 | 0 | #NUM! | 0 | Baseline |
| Male | 1 to 4 | 0 | #NUM! | 0 | Baseline |
| Female | 35+ | 0 | #NUM! | 0 | Baseline |

| Male | 5 to 14 | 0 | #NUM! | 0 | Baseline |
| --- | --- | --- | --- | --- | --- |
| Female | 1 to 4 | 0 | #NUM! | 0 | Baseline |
| Male | 5 to 14 | 0 | #NUM! | 0 | Baseline |
| Male | 22 to 35 | 0 | #NUM! | 0 | Baseline |
| Female | 22 to 35 | 0 | #NUM! | 0 | Baseline |
| Male | 22 to 35 | 0 | #NUM! | 0 | Baseline |
| Female | 35+ | 0 | #NUM! | 0 | Baseline |
| Male | 22 to 35 | 0 | #NUM! | 0 | Baseline |
| Female | 22 to 35 | 0 | #NUM! | 0 | Baseline |
| Female | 15 to 21 | 0 | #NUM! | 0 | Baseline |
| Male | 35+ | 0 | #NUM! | 0 | Baseline |
| Female | 35+ | 0 | #NUM! | 0 | Baseline |
| Female | 22 to 35 | 0 | #NUM! | 0 | Baseline |
| Male | 35+ | 0 | #NUM! | 0 | Baseline |
| Female | 22 to 35 | 0 | #NUM! | 0 | Baseline |
| Male | 22 to 35 | 0 | #NUM! | 0 | Baseline |
| Male | 22 to 35 | 0 | #NUM! | 0 | Baseline |
| Female | 22 to 35 | 0 | #NUM! | 0 | Baseline |
| Male | 15 to 21 | 0 | #NUM! | 0 | Baseline |
| Male | 15 to 21 | 0 | #NUM! | 0 | Baseline |
| Male | 15 to 21 | 0 | #NUM! | 0 | Baseline |
| Female | 15 to 21 | 0 | #NUM! | 0 | Baseline |
| Female | 35+ | 0 | #NUM! | 0 | Baseline |
| Female | 35+ | 0 | #NUM! | 0 | Baseline |
| Male | 15 to 21 | 0 | #NUM! | 0 | Baseline |
| Female | 15 to 21 | 0 | #NUM! | 0 | Baseline |
| Female | 15 to 21 | 0 | #NUM! | 0 | Baseline |
| Male | 22 to 35 | 0 | #NUM! | 0 | Baseline |
| Female | 15 to 21 | 0 | #NUM! | 0 | Baseline |
| Male | 15 to 21 | 0 | #NUM! | 0 | Baseline |
| Male | 15 to 21 | 0 | #NUM! | 0 | Baseline |
| Male | 1 to 4 | 0 | #NUM! | 0 | Baseline |
| Male | 1 to 4 | 0 | #NUM! | 0 | Baseline |
| Female | 1 to 4 | 0 | #NUM! | 0 | Baseline |
| Female | 1 to 4 | 0 | #NUM! | 0 | Baseline |
| Female | 22 to 35 | 0 | #NUM! | 0 | Baseline |
| Female | 5 to 14 | 0 | #NUM! | 0 | Baseline |
| Female | 1 to 4 | 0 | #NUM! | 0 | Baseline |
| Male | 1 to 4 | 0 | #NUM! | 0 | Baseline |
| Female | 5 to 14 | 0 | #NUM! | 0 | Baseline |
| Male | 22 to 35 | 0 | #NUM! | 0 | Baseline |
| Male | 5 to 14 | 0 | #NUM! | 0 | Baseline |
| Male | 1 to 4 | 0 | #NUM! | 0 | Baseline |
| Female | 1 to 4 | 0 | #NUM! | 0 | Baseline |
| Female | 5 to 14 | 0 | #NUM! | 0 | Baseline |
| Male | 5 to 14 | 0 | #NUM! | 0 | Baseline |
| Male | 1 to 4 | 0 | #NUM! | 0 | Baseline |
| Male | 5 to 14 | 0 | #NUM! | 0 | Baseline |
| Male | 35+ | 0 | #NUM! | 0 | Baseline |
| Female | 5 to 14 | 0 | #NUM! | 0 | Baseline |

| Female | 5 to 14 | 0 | #NUM! | 0 | Baseline |
| --- | --- | --- | --- | --- | --- |
| Male | 22 to 35 | 0 | #NUM! | 0 | Baseline |
| Male | 15 to 21 | 0 | #NUM! | 0 | Baseline |
| Male | 22 to 35 | 0 | #NUM! | 0 | Baseline |
| Female | 22 to 35 | 0 | #NUM! | 0 | Baseline |
| Male | 35+ | 0 | #NUM! | 0 | Baseline |
| Female | 35+ | 0 | #NUM! | 0 | Baseline |
| Male | 22 to 35 | 0 | #NUM! | 0 | Baseline |
| Female | 5 to 14 | 0 | #NUM! | 0 | Baseline |
| Male | 15 to 21 | 0 | #NUM! | 0 | Baseline |
| Male | 5 to 14 | 0 | #NUM! | 0 | Baseline |
| Male | 5 to 14 | 0 | #NUM! | 0 | Baseline |
| Male | 15 to 21 | 0 | #NUM! | 0 | Baseline |
| Male | 5 to 14 | 0 | #NUM! | 0 | Baseline |
| Female | 22 to 35 | 0 | #NUM! | 0 | Baseline |
| Female | 22 to 35 | 0 | #NUM! | 0 | Baseline |
| Female | 22 to 35 | 0 | #NUM! | 0 | Baseline |
| Male | 35+ | 0 | #NUM! | 0 | Baseline |
| Male | 15 to 21 | 0 | #NUM! | 0 | Baseline |
| Male | 22 to 35 | 0 | #NUM! | 0 | Baseline |
| Male | 22 to 35 | 0 | #NUM! | 0 | Baseline |
| Female | 22 to 35 | 0 | #NUM! | 0 | Baseline |
| Male | 15 to 21 | 0 | #NUM! | 0 | Baseline |
| Male | 5 to 14 | 0 | #NUM! | 0 | Baseline |
| Male | 22 to 35 | 0 | #NUM! | 0 | Baseline |
| Male | 5 to 14 | 0 | #NUM! | 0 | Baseline |
| Male | 15 to 21 | 0 | #NUM! | 0 | Baseline |
| Male | 15 to 21 | 0 | #NUM! | 0 | Baseline |
| Male | 5 to 14 | 0 | #NUM! | 0 | Baseline |
| Male | 5 to 14 | 0 | #NUM! | 0 | Baseline |
| Male | 15 to 21 | 0 | #NUM! | 0 | Baseline |
| Male | 15 to 21 | 0 | #NUM! | 0 | Baseline |
| Female | 5 to 14 | 0 | #NUM! | 0 | Baseline |
| Female | 5 to 14 | 0 | #NUM! | 0 | Baseline |
| Male | 5 to 14 | 0 | #NUM! | 0 | Baseline |
| Male | 5 to 14 | 0 | #NUM! | 0 | Baseline |
| Female | 5 to 14 | 0 | #NUM! | 0 | Baseline |
| Female | 15 to 21 | 0 | #NUM! | 0 | Baseline |
| Female | 5 to 14 | 0 | #NUM! | 0 | Baseline |
| Female | 5 to 14 | 0 | #NUM! | 0 | Baseline |
| Female | 5 to 14 | 0 | #NUM! | 0 | Baseline |
| Female | 22 to 35 | 0 | #NUM! | 0 | Baseline |
| Male | 22 to 35 | 0 | #NUM! | 0 | Baseline |
| Female | 22 to 35 | 0 | #NUM! | 0 | Baseline |
| Male | 35+ | 0 | #NUM! | 0 | Baseline |
| Female | 35+ | 0 | #NUM! | 0 | Baseline |
| Female | 35+ | 0 | #NUM! | 0 | Baseline |
| Male | 22 to 35 | 0 | #NUM! | 0 | Baseline |
| Female | 35+ | 0 | #NUM! | 0 | Baseline |
| Female | 35+ | 0 | #NUM! | 0 | Baseline |

| Male | 35+ | 0 | #NUM! | 0 | Baseline |
| --- | --- | --- | --- | --- | --- |
| Female | 22 to 35 | 0 | #NUM! | 0 | Baseline |
| Male | 35+ | 0 | #NUM! | 0 | Baseline |
| Male | 35+ | 0 | #NUM! | 0 | Baseline |
| Male | 1 to 4 | 0 | #NUM! | 0 | Baseline |
| Female | 5 to 14 | 0 | #NUM! | 0 | Baseline |
| Female | 5 to 14 | 0 | #NUM! | 0 | Baseline |
| Female | 5 to 14 | 0 | #NUM! | 0 | Baseline |
| Female | 15 to 21 | 0 | #NUM! | 0 | Baseline |
| Female | 35+ | 0 | #NUM! | 0 | Baseline |
| Female | 15 to 21 | 0 | #NUM! | 0 | Baseline |
| Female | 15 to 21 | 0 | #NUM! | 0 | Baseline |
| Female | 15 to 21 | 0 | #NUM! | 0 | Baseline |
| Female | 15 to 21 | 0 | #NUM! | 0 | Baseline |
| Female | 15 to 21 | 0 | #NUM! | 0 | Baseline |
| Female | 1 to 4 | 0 | #NUM! | 0 | Baseline |
| Female | 5 to 14 | 0 | #NUM! | 0 | Baseline |
| Female | 22 to 35 | 0 | #NUM! | 0 | Baseline |
| Female | 35+ | 0 | #NUM! | 0 | Baseline |
| Male | 35+ | 0 | #NUM! | 0 | Baseline |
| Female | 35+ | 0 | #NUM! | 0 | Baseline |
| Male | 5 to 14 | 0 | #NUM! | 0 | Baseline |
| Female | 5 to 14 | 0 | #NUM! | 0 | Baseline |
| Male | 35+ | 0 | #NUM! | 0 | Baseline |
| Male | 22 to 35 | 0 | #NUM! | 0 | Baseline |
| Male | 1 to 4 | 0 | #NUM! | 0 | Baseline |
| Male | 15 to 21 | 0 | #NUM! | 0 | Baseline |
| Male | 15 to 21 | 0 | #NUM! | 0 | Baseline |
| Male | 15 to 21 | 0 | #NUM! | 0 | Baseline |
| Female | 5 to 14 | 0 | #NUM! | 0 | Baseline |
| Male | 15 to 21 | 0 | #NUM! | 0 | Baseline |
| Male | 15 to 21 | 0 | #NUM! | 0 | Baseline |
| Male | 15 to 21 | 0 | #NUM! | 0 | Baseline |
| Female | 35+ | 0 | #NUM! | 0 | Baseline |
| Female | 22 to 35 | 0 | #NUM! | 0 | Baseline |
| Female | 22 to 35 | 0 | #NUM! | 0 | Baseline |
| Female | 15 to 21 | 0 | #NUM! | 0 | Baseline |
| Female | 35+ | 0 | #NUM! | 0 | Baseline |
| Male | 22 to 35 | 0 | #NUM! | 0 | Baseline |
| Male | 5 to 14 | 0 | #NUM! | 0 | Baseline |
| Male | 5 to 14 | 0 | #NUM! | 0 | Baseline |
| Male | 22 to 35 | 0 | #NUM! | 0 | Baseline |
| Male | 35+ | 0 | #NUM! | 0 | Baseline |
| Female | 35+ | 0 | #NUM! | 0 | Baseline |
| Male | 15 to 21 | 0 | #NUM! | 0 | Baseline |
| Male | 35+ | 0 | #NUM! | 0 | Baseline |
| Female | 15 to 21 | 0 | #NUM! | 0 | Baseline |
| Female | 15 to 21 | 0 | #NUM! | 0 | Baseline |
| Male | 35+ | 0 | #NUM! | 0 | Baseline |
| Male | 35+ | 0 | #NUM! | 0 | Baseline |

| Male | 15 to 21 | 0 | #NUM! | 0 | Baseline |
| --- | --- | --- | --- | --- | --- |
| Female | 22 to 35 | 0 | #NUM! | 0 | Baseline |
| Male | 15 to 21 | 0 | #NUM! | 0 | Baseline |
| Female | 35+ | 0 | #NUM! | 0 | Baseline |
| Female | 15 to 21 | 0 | #NUM! | 0 | Baseline |
| Male | 35+ | 0 | #NUM! | 0 | Baseline |
| Female | 35+ | 0 | #NUM! | 0 | Baseline |
| Female | 22 to 35 | 0 | #NUM! | 0 | Baseline |
| Female | 22 to 35 | 0 | #NUM! | 0 | Baseline |
| Female | 22 to 35 | 0 | #NUM! | 0 | Baseline |
| Female | 35+ | 0 | #NUM! | 0 | Baseline |
| Male | 22 to 35 | 0 | #NUM! | 0 | Baseline |
| Male | 22 to 35 | 0 | #NUM! | 0 | Baseline |
| Female | 15 to 21 | 0 | #NUM! | 0 | Baseline |
| Male | 35+ | 0 | #NUM! | 0 | Baseline |
| Male | 35+ | 0 | #NUM! | 0 | Baseline |
| Male | 22 to 35 | 0 | #NUM! | 0 | Baseline |
| Male | 22 to 35 | 0 | #NUM! | 0 | Baseline |
| Male | 22 to 35 | 0 | #NUM! | 0 | Baseline |
| Female | 5 to 14 | 0 | #NUM! | 0 | Baseline |
| Male | 5 to 14 | 0 | #NUM! | 0 | Baseline |
| Male | 5 to 14 | 0 | #NUM! | 0 | Baseline |
| Female | 5 to 14 | 0 | #NUM! | 0 | Baseline |
| Female | 5 to 14 | 0 | #NUM! | 0 | Baseline |
| Female | 5 to 14 | 0 | #NUM! | 0 | Baseline |
| Female | 5 to 14 | 0 | #NUM! | 0 | Baseline |
| Female | 5 to 14 | 0 | #NUM! | 0 | Baseline |
| Female | 5 to 14 | 0 | #NUM! | 0 | Baseline |
| Female | 5 to 14 | 0 | #NUM! | 0 | Baseline |
| Female | 5 to 14 | 0 | #NUM! | 0 | Baseline |
| Female | 5 to 14 | 0 | #NUM! | 0 | Baseline |
| Male | 5 to 14 | 0 | #NUM! | 0 | Baseline |
| Male | 5 to 14 | 0 | #NUM! | 0 | Baseline |
| Male | 5 to 14 | 0 | #NUM! | 0 | Baseline |
| Female | 5 to 14 | 0 | #NUM! | 0 | Baseline |
| Female | 5 to 14 | 0 | #NUM! | 0 | Baseline |
| Male | 5 to 14 | 0 | #NUM! | 0 | Baseline |
| Male | 5 to 14 | 0 | #NUM! | 0 | Baseline |
| Male | 5 to 14 | 0 | #NUM! | 0 | Baseline |
| Male | 5 to 14 | 0 | #NUM! | 0 | Baseline |
| Male | 35+ | 0 | #NUM! | 0 | Baseline |
| Female | 22 to 35 | 0 | #NUM! | 0 | Baseline |
| Female | 22 to 35 | 0 | #NUM! | 0 | Baseline |
| Male | 35+ | 0 | #NUM! | 0 | Baseline |
| Male | 5 to 14 | 0 | #NUM! | 0 | Baseline |
| Female | 22 to 35 | 0 | #NUM! | 0 | Baseline |
| Female | 22 to 35 | 0 | #NUM! | 0 | Baseline |
| Male | 22 to 35 | 0 | #NUM! | 0 | Baseline |
| Female | 22 to 35 | 0 | #NUM! | 0 | Baseline |
| Female | 1 to 4 | 0 | #NUM! | 0 | Baseline |

| Male | 35+ | 0 | #NUM! | 0 | Baseline |
| --- | --- | --- | --- | --- | --- |
| Male | 35+ | 0 | #NUM! | 0 | Baseline |
| Male | 35+ | 0 | #NUM! | 0 | Baseline |
| Male | 22 to 35 | 0 | #NUM! | 0 | Baseline |
| Male | 35+ | 0 | #NUM! | 0 | Baseline |
| Male | 35+ | 0 | #NUM! | 0 | Baseline |
| Female | 35+ | 0 | #NUM! | 0 | Baseline |
| Female | 22 to 35 | 0 | #NUM! | 0 | Baseline |
| Female | 35+ | 0 | #NUM! | 0 | Baseline |
| Female | 35+ | 0 | #NUM! | 0 | Baseline |
| Female | 35+ | 0 | #NUM! | 0 | Baseline |
| Male | 35+ | 0 | #NUM! | 0 | Baseline |
| Female | 22 to 35 | 0 | #NUM! | 0 | Baseline |
| Female | 35+ | 0 | #NUM! | 0 | Baseline |
| Female | 5 to 14 | 0 | #NUM! | 0 | Baseline |
| Female | 22 to 35 | 0 | #NUM! | 0 | Baseline |
| Female | 22 to 35 | 0 | #NUM! | 0 | Baseline |
| Female | 22 to 35 | 0 | #NUM! | 0 | Baseline |
| Female | 35+ | 0 | #NUM! | 0 | Baseline |
| Female | 22 to 35 | 0 | #NUM! | 0 | Baseline |
| Female | 35+ | 0 | #NUM! | 0 | Baseline |
| Female | 35+ | 0 | #NUM! | 0 | Baseline |
| Female | 22 to 35 | 0 | #NUM! | 0 | Baseline |
| Female | 5 to 14 | 0 | #NUM! | 0 | Baseline |
| Male | 22 to 35 | 0 | #NUM! | 0 | Baseline |
| Male | 35+ | 0 | #NUM! | 0 | Baseline |
| Male | 5 to 14 | 0 | #NUM! | 0 | Baseline |
| Female | 5 to 14 | 0 | #NUM! | 0 | Baseline |
| Female | 5 to 14 | 0 | #NUM! | 0 | Baseline |
| Female | 5 to 14 | 0 | #NUM! | 0 | Baseline |
| Female | 5 to 14 | 0 | #NUM! | 0 | Baseline |
| Male | 5 to 14 | 0 | #NUM! | 0 | Baseline |
| Male | 1 to 4 | 0 | #NUM! | 0 | Baseline |
| Male | 1 to 4 | 0 | #NUM! | 0 | Baseline |
| Male | 5 to 14 | 0 | #NUM! | 0 | Baseline |
| Female | 5 to 14 | 0 | #NUM! | 0 | Baseline |
| Male | 5 to 14 | 0 | #NUM! | 0 | Baseline |
| Male | 15 to 21 | 0 | #NUM! | 0 | Baseline |
| Male | 5 to 14 | 0 | #NUM! | 0 | Baseline |
| Female | 15 to 21 | 0 | #NUM! | 0 | Baseline |
| Male | 15 to 21 | 0 | #NUM! | 0 | Baseline |
| Female | 15 to 21 | 0 | #NUM! | 0 | Baseline |
| Female | 15 to 21 | 0 | #NUM! | 0 | Baseline |
| Female | 15 to 21 | 0 | #NUM! | 0 | Baseline |
| Female | 5 to 14 | 0 | #NUM! | 0 | Baseline |
| Female | 5 to 14 | 0 | #NUM! | 0 | Baseline |
| Male | 1 to 4 | 0 | #NUM! | 0 | Baseline |
| Male | 5 to 14 | 0 | #NUM! | 0 | Baseline |
| Male | 5 to 14 | 0 | #NUM! | 0 | Baseline |
| Male | 15 to 21 | 0 | #NUM! | 0 | Baseline |

| Male | 5 to 14 | 0 | #NUM! | 0 | Baseline |
| --- | --- | --- | --- | --- | --- |
| Male | 1 to 4 | 0 | #NUM! | 0 | Baseline |
| Female | 5 to 14 | 0 | #NUM! | 0 | Baseline |
| Male | 15 to 21 | 0 | #NUM! | 0 | Baseline |
| Female | 15 to 21 | 0 | #NUM! | 0 | Baseline |
| Female | 5 to 14 | 0 | #NUM! | 0 | Baseline |
| Female | 15 to 21 | 0 | #NUM! | 0 | Baseline |
| Male | 5 to 14 | 0 | #NUM! | 0 | Baseline |
| Male | 15 to 21 | 0 | #NUM! | 0 | Baseline |
| Male | 15 to 21 | 0 | #NUM! | 0 | Baseline |
| Male | 15 to 21 | 0 | #NUM! | 0 | Baseline |
| Male | 15 to 21 | 0 | #NUM! | 0 | Baseline |
| Female | 15 to 21 | 0 | #NUM! | 0 | Baseline |
| Female | 15 to 21 | 0 | #NUM! | 0 | Baseline |
| Female | 5 to 14 | 0 | #NUM! | 0 | Baseline |
| Male | 5 to 14 | 0 | #NUM! | 0 | Baseline |
| Male | 5 to 14 | 0 | #NUM! | 0 | Baseline |
| Female | 5 to 14 | 0 | #NUM! | 0 | Baseline |
| Female | 5 to 14 | 0 | #NUM! | 0 | Baseline |
| Male | 5 to 14 | 0 | #NUM! | 0 | Baseline |
| Male | 22 to 35 | 0 | #NUM! | 0 | Baseline |
| Male | 22 to 35 | 0 | #NUM! | 0 | Baseline |
| Male | 22 to 35 | 0 | #NUM! | 0 | Baseline |
| Male | 22 to 35 | 0 | #NUM! | 0 | Baseline |
| Male | 15 to 21 | 0 | #NUM! | 0 | Baseline |
| Male | 15 to 21 | 0 | #NUM! | 0 | Baseline |
| Female | 5 to 14 | 0 | #NUM! | 0 | Baseline |
| Male | 15 to 21 | 0 | #NUM! | 0 | Baseline |
| Male | 15 to 21 | 0 | #NUM! | 0 | Baseline |
| Male | 15 to 21 | 0 | #NUM! | 0 | Baseline |
| Male | 15 to 21 | 0 | #NUM! | 0 | Baseline |
| Male | 5 to 14 | 0 | #NUM! | 0 | Baseline |
| Male | 5 to 14 | 0 | #NUM! | 0 | Baseline |
| Male | 5 to 14 | 0 | #NUM! | 0 | Baseline |
| Male | 15 to 21 | 0 | #NUM! | 0 | Baseline |
| Male | 15 to 21 | 0 | #NUM! | 0 | Baseline |
| Male | 5 to 14 | 0 | #NUM! | 0 | Baseline |
| Male | 5 to 14 | 0 | #NUM! | 0 | Baseline |
| Male | 15 to 21 | 0 | #NUM! | 0 | Baseline |
| Male | 5 to 14 | 0 | #NUM! | 0 | Baseline |
| Male | 15 to 21 | 0 | #NUM! | 0 | Baseline |
| Female | 5 to 14 | 0 | #NUM! | 0 | Baseline |
| Male | 15 to 21 | 0 | #NUM! | 0 | Baseline |
| Female | 15 to 21 | 0 | #NUM! | 0 | Baseline |
| Male | 5 to 14 | 0 | #NUM! | 0 | Baseline |
| Female | 15 to 21 | 0 | #NUM! | 0 | Baseline |
| Male | 5 to 14 | 3 | 0 | 0 | Baseline |
| Male | 35+ | 0 | #NUM! | 0 | Baseline |
| Male | 5 to 14 | 0 | #NUM! | 0 | Baseline |
| Female | 35+ | 0 | #NUM! | 0 | Baseline |

| Male | 5 to 14 | 0 | #NUM! | 0 | Baseline |
| --- | --- | --- | --- | --- | --- |
| Female | 15 to 21 | 0 | #NUM! | 0 | Baseline |
| Female | 15 to 21 | 0 | #NUM! | 0 | Baseline |
| Female | 15 to 21 | 0 | #NUM! | 0 | Baseline |
| Female | 15 to 21 | 0 | #NUM! | 0 | Baseline |
| Male | 22 to 35 | 0 | #NUM! | 0 | Baseline |
| Female | 35+ | 0 | #NUM! | 0 | Baseline |
| Female | 35+ | 0 | #NUM! | 0 | Baseline |
| Female | 35+ | 0 | #NUM! | 0 | Baseline |
| Female | 15 to 21 | 0 | #NUM! | 0 | Baseline |
| Female | 35+ | 0 | #NUM! | 0 | Baseline |
| Female | 5 to 14 | 0 | #NUM! | 0 | Baseline |
| Female | 35+ | 0 | #NUM! | 0 | Baseline |
| Female | 15 to 21 | 3 | 0 | 0 | Baseline |
| Female | 15 to 21 | 0 | #NUM! | 0 | Baseline |
| Female | 15 to 21 | 0 | #NUM! | 0 | Baseline |
| Male | 22 to 35 | 0 | #NUM! | 0 | Baseline |
| Male | 22 to 35 | 0 | #NUM! | 0 | Baseline |
| Female | 22 to 35 | 0 | #NUM! | 0 | Baseline |
| Female | 5 to 14 | 0 | #NUM! | 0 | Baseline |
| Female | 5 to 14 | 0 | #NUM! | 0 | Baseline |
| Male | 35+ | 0 | #NUM! | 0 | Baseline |
| Male | 5 to 14 | 0 | #NUM! | 0 | Baseline |
| Male | 35+ | 0 | #NUM! | 0 | Baseline |
| Male | 35+ | 0 | #NUM! | 0 | Baseline |
| Male | 15 to 21 | 0 | #NUM! | 0 | Baseline |
| Male | 35+ | 0 | #NUM! | 0 | Baseline |
| Male | 5 to 14 | 0 | #NUM! | 0 | Baseline |
| Male | 15 to 21 | 0 | #NUM! | 0 | Baseline |
| Male | 5 to 14 | 0 | #NUM! | 0 | Baseline |
| Female | 5 to 14 | 0 | #NUM! | 0 | Baseline |
| Male | 5 to 14 | 0 | #NUM! | 0 | Baseline |
| Male | 5 to 14 | 0 | #NUM! | 0 | Baseline |
| Male | 5 to 14 | 0 | #NUM! | 0 | Baseline |
| Female | 5 to 14 | 0 | #NUM! | 0 | Baseline |
| Female | 5 to 14 | 0 | #NUM! | 0 | Baseline |
| Female | 15 to 21 | 0 | #NUM! | 0 | Baseline |
| Male | 35+ | 0 | #NUM! | 0 | Baseline |
| Male | 5 to 14 | 0 | #NUM! | 0 | Baseline |
| Male | 5 to 14 | 3 | 0 | 0 | Baseline |
| Male | 5 to 14 | 0 | #NUM! | 0 | Baseline |
| Female | 5 to 14 | 0 | #NUM! | 0 | Baseline |
| Female | 5 to 14 | 0 | #NUM! | 0 | Baseline |
| Female | 5 to 14 | 0 | #NUM! | 0 | Baseline |
| Female | 5 to 14 | 0 | #NUM! | 0 | Baseline |
| Female | 5 to 14 | 0 | #NUM! | 0 | Baseline |
| Male | 5 to 14 | 0 | #NUM! | 0 | Baseline |
| Male | 35+ | 0 | #NUM! | 0 | Baseline |
| Male | 5 to 14 | 3 | 0 | 0 | Baseline |
| Female | 5 to 14 | 3 | 0 | 0 | Baseline |

| Female | 5 to 14 | 4 | 0 | 0 | Baseline |
| --- | --- | --- | --- | --- | --- |
| Female | 5 to 14 | 0 | #NUM! | 0 | Baseline |
| Female | 35+ | 0 | #NUM! | 0 | Baseline |
| Female | 15 to 21 | 4 | 0 | 0 | Baseline |
| Male | 5 to 14 | 0 | #NUM! | 0 | Baseline |
| Male | 15 to 21 | 0 | #NUM! | 0 | Baseline |
| Male | 22 to 35 | 0 | #NUM! | 0 | Baseline |
| Male | 5 to 14 | 0 | #NUM! | 0 | Baseline |
| Female | 15 to 21 | 0 | #NUM! | 0 | Baseline |
| Female | 5 to 14 | 0 | #NUM! | 0 | Baseline |
| Male | 22 to 35 | 0 | #NUM! | 0 | Baseline |
| Female | 5 to 14 | 0 | #NUM! | 0 | Baseline |
| Female | 5 to 14 | 0 | #NUM! | 0 | Baseline |
| Female | 5 to 14 | 0 | #NUM! | 0 | Baseline |
| Female | 5 to 14 | 0 | #NUM! | 0 | Baseline |
| Female | 22 to 35 | 0 | #NUM! | 0 | Baseline |
| Female | 15 to 21 | 0 | #NUM! | 0 | Baseline |
| Female | 35+ | 0 | #NUM! | 0 | Baseline |
| Female | 5 to 14 | 0 | #NUM! | 0 | Baseline |
| Female | 15 to 21 | 0 | #NUM! | 0 | Baseline |
| Female | 5 to 14 | 0 | #NUM! | 0 | Baseline |
| Female | 5 to 14 | 0 | #NUM! | 0 | Baseline |
| Female | 15 to 21 | 0 | #NUM! | 0 | Baseline |
| Female | 22 to 35 | 0 | #NUM! | 0 | Baseline |
| Female | 15 to 21 | 0 | #NUM! | 0 | Baseline |
| Female | 5 to 14 | 0 | #NUM! | 0 | Baseline |
| Male | 1 to 4 | 0 | #NUM! | 0 | Baseline |
| Female | 5 to 14 | 0 | #NUM! | 0 | Baseline |
| Male | 1 to 4 | 0 | #NUM! | 0 | Baseline |
| Female | 5 to 14 | 0 | #NUM! | 0 | Baseline |
| Female | 15 to 21 |  | #NUM! | 0 | Baseline |
| Female | 5 to 14 | 0 | #NUM! | 0 | Baseline |
| Male | 15 to 21 | 0 | #NUM! | 0 | Baseline |
| Male | 5 to 14 | 0 | #NUM! | 0 | Baseline |
| Male | 22 to 35 | 0 | #NUM! | 0 | Baseline |
| Male | 5 to 14 | 0 | #NUM! | 0 | Baseline |
| Female | 35+ | 0 | #NUM! | 0 | Baseline |
| Female | 5 to 14 | 0 | #NUM! | 0 | Baseline |
| Female | 22 to 35 | 2 | 0 | 0 | Baseline |
| Male | 5 to 14 | 0 | #NUM! | 0 | Baseline |
| Female | 22 to 35 | 0 | #NUM! | 0 | Baseline |
| Female | 15 to 21 | 0 | #NUM! | 0 | Baseline |
| Female | 5 to 14 | 0 | #NUM! | 0 | Baseline |
| Female | 22 to 35 | 0 | #NUM! | 0 | Baseline |
| Female | 22 to 35 | 0 | #NUM! | 0 | Baseline |
| Male | 5 to 14 | 0 | #NUM! | 0 | Baseline |
| Female | 22 to 35 | 0 | #NUM! | 0 | Baseline |
| Female | 5 to 14 | 0 | #NUM! | 0 | Baseline |
| Female | 5 to 14 | 0 | #NUM! | 0 | Baseline |
| Female | 1 to 4 | 0 | #NUM! | 0 | Baseline |

| Female | 35+ | 0 | #NUM! | 0 | Baseline |
| --- | --- | --- | --- | --- | --- |
| Male | 5 to 14 | 0 | #NUM! | 0 | Baseline |
| Female | 1 to 4 | 0 | #NUM! | 0 | Baseline |
| Female | 1 to 4 | 0 | #NUM! | 0 | Baseline |
| Male | 15 to 21 | 0 | #NUM! | 0 | Baseline |
| Female | 15 to 21 | 0 | #NUM! | 0 | Baseline |
| Female | 22 to 35 | 0 | #NUM! | 0 | Baseline |
| Female | 5 to 14 | 0 | #NUM! | 0 | Baseline |
| Female | 15 to 21 | 0 | #NUM! | 0 | Baseline |
| Female | 22 to 35 | 0 | #NUM! | 0 | Baseline |
| Female | 5 to 14 | 0 | #NUM! | 0 | Baseline |
| Female | 5 to 14 | 0 | #NUM! | 0 | Baseline |
| Female | 5 to 14 | 0 | #NUM! | 0 | Baseline |
| Female | 1 to 4 | 0 | #NUM! | 0 | Baseline |
| Male | 15 to 21 | 0 | #NUM! | 0 | Baseline |
| Female | 22 to 35 | 0 | #NUM! | 0 | Baseline |
| Male | 5 to 14 | 0 | #NUM! | 0 | Baseline |
| Male | 5 to 14 | 0 | #NUM! | 0 | Baseline |
| Female | 22 to 35 | 0 | #NUM! | 0 | Baseline |
| Male | 22 to 35 | 0 | #NUM! | 0 | Baseline |
| Male | 35+ | 0 | #NUM! | 0 | Baseline |
| Female | 5 to 14 | 0 | #NUM! | 0 | Baseline |
| Female | 22 to 35 | 0 | #NUM! | 0 | Baseline |
| Male | 1 to 4 | 0 | #NUM! | 0 | Baseline |
| Male | 22 to 35 | 0 | #NUM! | 0 | Baseline |
| Male | 5 to 14 | 0 | #NUM! | 0 | Baseline |
| Female | 35+ | 0 | #NUM! | 0 | Baseline |
| Female | 22 to 35 | 0 | #NUM! | 0 | Baseline |
| Female | 15 to 21 | 0 | #NUM! | 0 | Baseline |
| Female | 35+ | 0 | #NUM! | 0 | Baseline |
| Female | 22 to 35 | 0 | #NUM! | 0 | Baseline |
| Female | 15 to 21 | 0 | #NUM! | 0 | Baseline |
| Female | 22 to 35 | 0 | #NUM! | 0 | Baseline |
| Female | 22 to 35 | 0 | #NUM! | 0 | Baseline |
| Female | 35+ | 0 | #NUM! | 0 | Baseline |
| Female | 22 to 35 | 0 | #NUM! | 0 | Baseline |
| Male | 22 to 35 | 0 | #NUM! | 0 | Baseline |
| Female | 22 to 35 | 0 | #NUM! | 0 | Baseline |
| Female | 5 to 14 | 0 | #NUM! | 0 | Baseline |
| Female | 35+ | 0 | #NUM! | 0 | Baseline |
| Female | 5 to 14 | 0 | #NUM! | 0 | Baseline |
| Male | 15 to 21 | 0 | #NUM! | 0 | Baseline |
| Female | 15 to 21 | 0 | #NUM! | 0 | Baseline |
| Female | 5 to 14 | 0 | #NUM! | 0 | Baseline |
| Male | 5 to 14 | 0 | #NUM! | 0 | Baseline |
| Male | 22 to 35 | 0 | #NUM! | 0 | Baseline |
| Male | 5 to 14 | 0 | #NUM! | 0 | Baseline |
| Male | 35+ | 0 | #NUM! | 0 | Baseline |
| Female | 22 to 35 | 0 | #NUM! | 0 | Baseline |
| Male | 35+ | 0 | #NUM! | 0 | Baseline |

| Female | 22 to 35 | 0 | #NUM! | 0 | Baseline |
| --- | --- | --- | --- | --- | --- |
| Female | 35+ | 0 | #NUM! | 0 | Baseline |
| Female | 1 to 4 | 0 | #NUM! | 0 | Baseline |
| Male | 5 to 14 | 0 | #NUM! | 0 | Baseline |
| Female | 35+ | 0 | #NUM! | 0 | Baseline |
| Male | 1 to 4 | 3 | 0 | 0 | Baseline |
| Female | 35+ | 0 | #NUM! | 0 | Baseline |
| Female | 35+ | 0 | #NUM! | 0 | Baseline |
| Female | 35+ | 0 | #NUM! | 0 | Baseline |
| Male | 5 to 14 | 0 | #NUM! | 0 | Baseline |
| Female | 15 to 21 | 0 | #NUM! | 0 | Baseline |
| Female | 35+ | 0 | #NUM! | 0 | Baseline |
| Male | 5 to 14 | 0 | #NUM! | 0 | Baseline |
| Male | 5 to 14 | 0 | #NUM! | 0 | Baseline |
| Male | 5 to 14 | 0 | #NUM! | 0 | Baseline |
| Male | 5 to 14 | 0 | #NUM! | 0 | Baseline |
| Female | 5 to 14 | 0 | #NUM! | 0 | Baseline |
| Male | 5 to 14 | 0 | #NUM! | 0 | Baseline |
| Male | 5 to 14 | 0 | #NUM! | 0 | Baseline |
| Female | 5 to 14 | 0 | #NUM! | 0 | Baseline |
| Male | 5 to 14 | 0 | #NUM! | 0 | Baseline |
| Male | 15 to 21 | 0 | #NUM! | 0 | Baseline |
| Male | 1 to 4 | 0 | #NUM! | 0 | Baseline |
| Male | 5 to 14 | 0 | #NUM! | 0 | Baseline |
| Female | 5 to 14 | 0 | #NUM! | 0 | Baseline |
| Male | 5 to 14 | 0 | #NUM! | 0 | Baseline |
| Female | 15 to 21 | 0 | #NUM! | 0 | Baseline |
| Male | 1 to 4 | 0 | #NUM! | 0 | Baseline |
| Male | 5 to 14 | 0 | #NUM! | 0 | Baseline |
| Female | 5 to 14 | 0 | #NUM! | 0 | Baseline |
| Male | 5 to 14 | 0 | #NUM! | 0 | Baseline |
| Female | 5 to 14 | 0 | #NUM! | 0 | Baseline |
| Female | 5 to 14 | 1 | 0 | 0 | Baseline |
| Male | 5 to 14 | 0 | #NUM! | 0 | Baseline |
| Male | 5 to 14 | 0 | #NUM! | 0 | Baseline |
| Female | 15 to 21 | 0 | #NUM! | 0 | Baseline |
| Female | 15 to 21 | 0 | #NUM! | 0 | Baseline |
| Female | 5 to 14 | 0 | #NUM! | 0 | Baseline |
| Male | 15 to 21 | 0 | #NUM! | 0 | Baseline |
| Male | 35+ | 0 | #NUM! | 0 | Baseline |
| Female | 22 to 35 | 0 | #NUM! | 0 | Baseline |
| Male | 5 to 14 | 0 | #NUM! | 0 | Baseline |
| Male | 5 to 14 | 0 | #NUM! | 0 | Baseline |
| Female | 22 to 35 | 0 | #NUM! | 0 | Baseline |
| Female | 22 to 35 | 0 | #NUM! | 0 | Baseline |
| Female | 35+ | 0 | #NUM! | 0 | Baseline |
| Male | 5 to 14 | 0 | #NUM! | 0 | Baseline |
| Female | 35+ | 0 | #NUM! | 0 | Baseline |
| Male | 22 to 35 | 0 | #NUM! | 0 | Baseline |
| Female | 15 to 21 | 0 | #NUM! | 0 | Baseline |

| Male | 5 to 14 | 0 | #NUM! | 0 | Baseline |
| --- | --- | --- | --- | --- | --- |
| Female | 22 to 35 | 0 | #NUM! | 0 | Baseline |
| Male | 15 to 21 | 1 | 0 | 0 | Baseline |
| Female | 5 to 14 | 0 | #NUM! | 0 | Baseline |
| Female | 35+ | 0 | #NUM! | 0 | Baseline |
| Female | 22 to 35 | 0 | #NUM! | 0 | Baseline |
| Male | 35+ | 1 | 0 | 0 | Baseline |
| Female | 35+ | 0 | #NUM! | 0 | Baseline |
| Male | 5 to 14 | 0 | #NUM! | 0 | Baseline |
| Male | 5 to 14 | 0 | #NUM! | 0 | Baseline |
| Male | 15 to 21 | 0 | #NUM! | 0 | Baseline |
| Male | 15 to 21 | 0 | #NUM! | 0 | Baseline |
| Male | 15 to 21 | 0 | #NUM! | 0 | Baseline |
| Female | 5 to 14 | 0 | #NUM! | 0 | Baseline |
| Female | 15 to 21 | 0 | #NUM! | 0 | Baseline |
| Male | 5 to 14 | 0 | #NUM! | 0 | Baseline |
| Female | 15 to 21 | 0 | #NUM! | 0 | Baseline |
| Male | 15 to 21 | 0 | #NUM! | 0 | Baseline |
| Female | 15 to 21 | 0 | #NUM! | 0 | Baseline |
| Male | 5 to 14 | 0 | #NUM! | 0 | Baseline |
| Male | 15 to 21 | 0 | #NUM! | 0 | Baseline |
| Female | 15 to 21 | 0 | #NUM! | 0 | Baseline |
| Male | 15 to 21 | 0 | #NUM! | 0 | Baseline |
| Male | 22 to 35 | 0 | #NUM! | 0 | Baseline |
| Male | 15 to 21 | 0 | #NUM! | 0 | Baseline |
| Male | 35+ | 0 | #NUM! | 0 | Baseline |
| Male | 15 to 21 | 0 | #NUM! | 0 | Baseline |
| Male | 15 to 21 | 0 | #NUM! | 0 | Baseline |
| Female | 5 to 14 | 0 | #NUM! | 0 | Baseline |
| Female | 15 to 21 | 0 | #NUM! | 0 | Baseline |
| Female | 15 to 21 | 0 | #NUM! | 0 | Baseline |
| Male | 1 to 4 | 0 | #NUM! | 0 | Baseline |
| Female | 35+ | 0 | #NUM! | 0 | Baseline |
| Male | 5 to 14 | 0 | #NUM! | 0 | Baseline |
| Female | 1 to 4 | 0 | #NUM! | 0 | Baseline |
| Female | 35+ | 0 | #NUM! | 0 | Baseline |
| Female | 35+ | 0 | #NUM! | 0 | Baseline |
| Female | 35+ | 0 | #NUM! | 0 | Baseline |
| Female | 35+ | 0 | #NUM! | 0 | Baseline |
| Female | 22 to 35 | 0 | #NUM! | 0 | Baseline |
| Male | 5 to 14 | 0 | #NUM! | 0 | Baseline |
| Female | 35+ | 0 | #NUM! | 0 | Baseline |
| Male | 1 to 4 | 0 | #NUM! | 0 | Baseline |
| Male | 1 to 4 | 0 | #NUM! | 0 | Baseline |
| Male | 1 to 4 | 0 | #NUM! | 0 | Baseline |
| Female | 22 to 35 | 0 | #NUM! | 0 | Baseline |
| Male | 15 to 21 | 0 | #NUM! | 0 | Baseline |
| Male | 15 to 21 | 0 | #NUM! | 0 | Baseline |
| Male | 22 to 35 | 0 | #NUM! | 0 | Baseline |
| Female | 22 to 35 | 0 | #NUM! | 0 | Baseline |

| Male | 35+ | 0 | #NUM! | 0 | Baseline |
| --- | --- | --- | --- | --- | --- |
| Female | 35+ | 0 | #NUM! | 0 | Baseline |
| Female | 35+ | 0 | #NUM! | 0 | Baseline |
| Male | 22 to 35 | 0 | #NUM! | 0 | Baseline |
| Male | 5 to 14 | 0 | #NUM! | 0 | Baseline |
| Male | 5 to 14 | 0 | #NUM! | 0 | Baseline |
| Female | 5 to 14 | 0 | #NUM! | 0 | Baseline |
| Female | 5 to 14 | 0 | #NUM! | 0 | Baseline |
| Female | 5 to 14 | 0 | #NUM! | 0 | Baseline |
| Female | 5 to 14 | 0 | #NUM! | 0 | Baseline |
| Female | 5 to 14 | 0 | #NUM! | 0 | Baseline |
| Male | 5 to 14 | 0 | #NUM! | 0 | Baseline |
| Male | 5 to 14 | 0 | #NUM! | 0 | Baseline |
| Female | 5 to 14 | 0 | #NUM! | 0 | Baseline |
| Male | 15 to 21 | 0 | #NUM! | 0 | Baseline |
| Male | 15 to 21 | 0 | #NUM! | 0 | Baseline |
| Male | 1 to 4 | 0 | #NUM! | 0 | Baseline |
| Male | 5 to 14 | 0 | #NUM! | 0 | Baseline |
| Male | 5 to 14 | 0 | #NUM! | 0 | Baseline |
| Female | 5 to 14 | 0 | #NUM! | 0 | Baseline |
| Female | 5 to 14 | 0 | #NUM! | 0 | Baseline |
| Female | 1 to 4 | 0 | #NUM! | 0 | Baseline |
| Female | 5 to 14 | 0 | #NUM! | 0 | Baseline |
| Female | 35+ | 0 | #NUM! | 0 | Baseline |
| Female | 35+ | 0 | #NUM! | 0 | Baseline |
| Male | 22 to 35 | 0 | #NUM! | 0 | Baseline |
| Male | 35+ | 1 | 0 | 0 | Baseline |
| Female | 22 to 35 | 0 | #NUM! | 0 | Baseline |
| Male | 22 to 35 | 0 | #NUM! | 0 | Baseline |
| Male | 35+ | 0 | #NUM! | 0 | Baseline |
| Male | 22 to 35 | 0 | #NUM! | 0 | Baseline |
| Female | 22 to 35 | 0 | #NUM! | 0 | Baseline |
| Female | 22 to 35 | 0 | #NUM! | 0 | Baseline |
| Male | 35+ | 0 | #NUM! | 0 | Baseline |
| Male | 35+ | 0 | #NUM! | 0 | Baseline |
| Male | 35+ | 0 | #NUM! | 0 | Baseline |
| Male | 35+ | 0 | #NUM! | 0 | Baseline |
| Male | 15 to 21 | 0 | #NUM! | 0 | Baseline |
| Female | 5 to 14 | 0 | #NUM! | 0 | Baseline |
| Male | 5 to 14 | 0 | #NUM! | 0 | Baseline |
| Female | 5 to 14 | 0 | #NUM! | 0 | Baseline |
| Male | 5 to 14 | 0 | #NUM! | 0 | Baseline |
| Male | 5 to 14 | 0 | #NUM! | 0 | Baseline |
| Male | 5 to 14 | 0 | #NUM! | 0 | Baseline |
| Male | 15 to 21 | 0 | #NUM! | 0 | Baseline |
| Male | 5 to 14 | 0 | #NUM! | 0 | Baseline |
| Female | 15 to 21 | 0 | #NUM! | 0 | Baseline |
| Male | 15 to 21 | 0 | #NUM! | 0 | Baseline |
| Male | 15 to 21 | 0 | #NUM! | 0 | Baseline |
| Male | 1 to 4 | 0 | #NUM! | 0 | Baseline |

| Male | 1 to 4 | 0 | #NUM! | 0 | Baseline |
| --- | --- | --- | --- | --- | --- |
| Male | 5 to 14 | 0 | #NUM! | 0 | Baseline |
| Male | 5 to 14 | 0 | #NUM! | 0 | Baseline |
| Male | 5 to 14 | 0 | #NUM! | 0 | Baseline |
| Female | 1 to 4 | 0 | #NUM! | 0 | Baseline |
| Female | 5 to 14 | 0 | #NUM! | 0 | Baseline |
| Male | 5 to 14 | 0 | #NUM! | 0 | Baseline |
| Female | 5 to 14 | 0 | #NUM! | 0 | Baseline |
| Female | 5 to 14 | 0 | #NUM! | 0 | Baseline |
| Female | 22 to 35 | 0 | #NUM! | 0 | Baseline |
| Male | 35+ | 0 | #NUM! | 0 | Baseline |
| Female | 22 to 35 | 0 | #NUM! | 0 | Baseline |
| Female | 22 to 35 | 0 | #NUM! | 0 | Baseline |
| Female | 22 to 35 | 0 | #NUM! | 0 | Baseline |
| Female | 35+ | 0 | #NUM! | 0 | Baseline |
| Male | 35+ | 0 | #NUM! | 0 | Baseline |
| Male | 22 to 35 | 0 | #NUM! | 0 | Baseline |
| Female | 35+ | 0 | #NUM! | 0 | Baseline |
| Male | 35+ | 0 | #NUM! | 0 | Baseline |
| Male | 35+ | 0 | #NUM! | 0 | Baseline |
| Male | 35+ | 0 | #NUM! | 0 | Baseline |
| Female | 22 to 35 | 0 | #NUM! | 0 | Baseline |
| Male | 22 to 35 | 0 | #NUM! | 0 | Baseline |
| Male | 35+ | 0 | #NUM! | 0 | Baseline |
| Female | 22 to 35 | 0 | #NUM! | 0 | Baseline |
| Male | 35+ | 0 | #NUM! | 0 | Baseline |
| Male | 35+ | 0 | #NUM! | 0 | Baseline |
| Male | 1 to 4 | 0 | #NUM! | 0 | Baseline |
| Male | 1 to 4 | 0 | #NUM! | 0 | Baseline |
| Male | 1 to 4 | 0 | #NUM! | 0 | Baseline |
| Male | 1 to 4 | 0 | #NUM! | 0 | Baseline |
| Male | 1 to 4 | 0 | #NUM! | 0 | Baseline |
| Male | 1 to 4 | 0 | #NUM! | 0 | Baseline |
| Male | 1 to 4 | 0 | #NUM! | 0 | Baseline |
| Male | 1 to 4 | 0 | #NUM! | 0 | Baseline |
| Male | 1 to 4 | 0 | #NUM! | 0 | Baseline |
| Male | 1 to 4 | 0 | #NUM! | 0 | Baseline |
| Male | 1 to 4 | 0 | #NUM! | 0 | Baseline |
| Male | 1 to 4 | 0 | #NUM! | 0 | Baseline |
| Male | 1 to 4 | 0 | #NUM! | 0 | Baseline |
| Male | 1 to 4 | 0 | #NUM! | 0 | Baseline |
| Male | 1 to 4 | 0 | #NUM! | 0 | Baseline |
| Male | 1 to 4 | 0 | #NUM! | 0 | Baseline |
| Male | 1 to 4 | 0 | #NUM! | 0 | Baseline |
| Male | 1 to 4 | 0 | #NUM! | 0 | Baseline |
| Male | 1 to 4 | 0 | #NUM! | 0 | Baseline |
| Male | 1 to 4 | 0 | #NUM! | 0 | Baseline |
| Male | 1 to 4 | 0 | #NUM! | 0 | Baseline |
| Male | 1 to 4 | 0 | #NUM! | 0 | Baseline |
| Male | 1 to 4 | 0 | #NUM! | 0 | Baseline |

| Male | 1 to 4 | 0 | #NUM! | 0 | Baseline |
| --- | --- | --- | --- | --- | --- |
| Male | 1 to 4 | 0 | #NUM! | 0 | Baseline |
| Male | 1 to 4 | 0 | #NUM! | 0 | Baseline |
| Male | 1 to 4 | 0 | #NUM! | 0 | Baseline |
| Male | 1 to 4 | 0 | #NUM! | 0 | Baseline |
| Male | 1 to 4 | 0 | #NUM! | 0 | Baseline |
| Male | 1 to 4 | 0 | #NUM! | 0 | Baseline |
| Male | 1 to 4 | 0 | #NUM! | 0 | Baseline |
| Male | 1 to 4 | 0 | #NUM! | 0 | Baseline |
| Male | 1 to 4 | 0 | #NUM! | 0 | Baseline |
| Male | 1 to 4 | 0 | #NUM! | 0 | Baseline |
| Male | 1 to 4 | 0 | #NUM! | 0 | Baseline |
| Male | 1 to 4 | 0 | #NUM! | 0 | Baseline |
| Male | 1 to 4 | 0 | #NUM! | 0 | Baseline |
| Male | 1 to 4 | 0 | #NUM! | 0 | Baseline |
| Male | 1 to 4 | 0 | #NUM! | 0 | Baseline |
| Male | 1 to 4 | 0 | #NUM! | 0 | Baseline |
| Male | 1 to 4 | 0 | #NUM! | 0 | Baseline |
| Male | 1 to 4 | 0 | #NUM! | 0 | Baseline |
| Male | 1 to 4 | 0 | #NUM! | 0 | Baseline |
| Male | 1 to 4 | 0 | #NUM! | 0 | Baseline |
| Male | 1 to 4 | 0 | #NUM! | 0 | Baseline |
| Male | 1 to 4 | 0 | #NUM! | 0 | Baseline |
| Male | 1 to 4 | 0 | #NUM! | 0 | Baseline |
| Male | 1 to 4 | 0 | #NUM! | 0 | Baseline |
| Male | 1 to 4 | 0 | #NUM! | 0 | Baseline |
| Male | 1 to 4 | 0 | #NUM! | 0 | Baseline |
| Male | 1 to 4 | 0 | #NUM! | 0 | Baseline |
| Male | 1 to 4 | 0 | #NUM! | 0 | Baseline |
| Male | 1 to 4 | 0 | #NUM! | 0 | Baseline |
| Male | 1 to 4 | 0 | #NUM! | 0 | Baseline |
| Male | 1 to 4 | 0 | #NUM! | 0 | Baseline |
| Male | 1 to 4 | 0 | #NUM! | 0 | Baseline |
| Male | 1 to 4 | 0 | #NUM! | 0 | Baseline |
| Male | 1 to 4 | 0 | #NUM! | 0 | Baseline |
| Male | 1 to 4 | 0 | #NUM! | 0 | Baseline |
| Male | 1 to 4 | 0 | #NUM! | 0 | Baseline |
| Male | 1 to 4 | 0 | #NUM! | 0 | Baseline |
| Male | 1 to 4 | 0 | #NUM! | 0 | Baseline |
| Male | 1 to 4 | 0 | #NUM! | 0 | Baseline |
| Male | 1 to 4 | 0 | #NUM! | 0 | Baseline |
| Male | 1 to 4 | 0 | #NUM! | 0 | Baseline |
| Male | 1 to 4 | 0 | #NUM! | 0 | Baseline |
| Male | 1 to 4 | 0 | #NUM! | 0 | Baseline |
| Male | 1 to 4 | 0 | #NUM! | 0 | Baseline |
| Male | 1 to 4 | 0 | #NUM! | 0 | Baseline |
| Male | 1 to 4 | 0 | #NUM! | 0 | Baseline |
| Male | 1 to 4 | 0 | #NUM! | 0 | Baseline |
| Male | 1 to 4 | 0 | #NUM! | 0 | Baseline |
| Male | 1 to 4 | 0 | #NUM! | 0 | Baseline |

| Male | 1 to 4 | 0 | #NUM! | 0 | Baseline |
| --- | --- | --- | --- | --- | --- |
| Male | 1 to 4 | 0 | #NUM! | 0 | Baseline |
| Male | 1 to 4 | 0 | #NUM! | 0 | Baseline |
| Male | 1 to 4 | 0 | #NUM! | 0 | Baseline |
| Male | 1 to 4 | 0 | #NUM! | 0 | Baseline |
| Male | 1 to 4 | 0 | #NUM! | 0 | Baseline |
| Male | 1 to 4 | 0 | #NUM! | 0 | Baseline |
| Male | 1 to 4 | 0 | #NUM! | 0 | Baseline |
| Male | 1 to 4 | 0 | #NUM! | 0 | Baseline |
| Male | 1 to 4 | 0 | #NUM! | 0 | Baseline |
| Male | 1 to 4 | 0 | #NUM! | 0 | Baseline |
| Male | 1 to 4 | 0 | #NUM! | 0 | Baseline |
| Male | 1 to 4 | 0 | #NUM! | 0 | Baseline |
| Male | 1 to 4 | 0 | #NUM! | 0 | Baseline |
| Male | 1 to 4 | 0 | #NUM! | 0 | Baseline |
| Male | 1 to 4 | 0 | #NUM! | 0 | Baseline |
| Male | 1 to 4 | 0 | #NUM! | 0 | Baseline |
| Male | 1 to 4 | 0 | #NUM! | 0 | Baseline |
| Male | 1 to 4 | 0 | #NUM! | 0 | Baseline |
| Male | 1 to 4 | 0 | #NUM! | 0 | Baseline |
| Male | 1 to 4 | 0 | #NUM! | 0 | Baseline |
| Male | 1 to 4 | 0 | #NUM! | 0 | Baseline |
| Female | 5 to 14 | 0 | #NUM! | 0 | Baseline |
| Female | 22 to 35 | 0 | #NUM! | 0 | Baseline |
| Male | 5 to 14 | 0 | #NUM! | 0 | Baseline |
| Female | 15 to 21 | 0 | #NUM! | 0 | Baseline |
| Female | 15 to 21 | 0 | #NUM! | 0 | Baseline |
| Female | 5 to 14 | 0 | #NUM! | 0 | Baseline |
| Male | 5 to 14 | 0 | #NUM! | 0 | Baseline |
| Male | 15 to 21 | 0 | #NUM! | 0 | Baseline |
| Male | 5 to 14 | 0 | #NUM! | 0 | Baseline |
| Male | 15 to 21 | 0 | #NUM! | 0 | Baseline |
| Female | 22 to 35 | 0 | #NUM! | 0 | Baseline |
| Male | 15 to 21 | 0 | #NUM! | 0 | Baseline |
| Male | 15 to 21 | 0 | #NUM! | 0 | Baseline |
| Female | 5 to 14 | 0 | #NUM! | 0 | Baseline |
| Male | 5 to 14 | 0 | #NUM! | 0 | Baseline |
| Male | 5 to 14 | 0 | #NUM! | 0 | Baseline |
| Female | 22 to 35 | 0 | #NUM! | 0 | Baseline |
| Female | 15 to 21 | 0 | #NUM! | 0 | Baseline |
| Female | 22 to 35 | 0 | #NUM! | 0 | Baseline |
| Female | 22 to 35 | 0 | #NUM! | 0 | Baseline |
| Female | 22 to 35 | 0 | #NUM! | 0 | Baseline |
| Female | 22 to 35 | 0 | #NUM! | 0 | Baseline |
| Female | 22 to 35 | 0 | #NUM! | 0 | Baseline |
| Male | 5 to 14 | 0 | #NUM! | 0 | Baseline |
| Male | 5 to 14 | 0 | #NUM! | 0 | Baseline |
| Female | 15 to 21 | 0 | #NUM! | 0 | Baseline |
| Female | 15 to 21 | 0 | #NUM! | 0 | Baseline |
| Female | 15 to 21 | 0 | #NUM! | 0 | Baseline |

| Female | 15 to 21 | 0 | #NUM! | 0 | Baseline |
| --- | --- | --- | --- | --- | --- |
| Male | 1 to 4 | 0 | #NUM! | 0 | Baseline |
| Female | 5 to 14 | 0 | #NUM! | 0 | Baseline |
| Female | 5 to 14 | 0 | #NUM! | 0 | Baseline |
| Male | 1 to 4 | 0 | #NUM! | 0 | Baseline |
| Female | 5 to 14 | 0 | #NUM! | 0 | Baseline |
| Female | 5 to 14 | 0 | #NUM! | 0 | Baseline |
| Male | 5 to 14 | 0 | #NUM! | 0 | Baseline |
| Male | 5 to 14 | 0 | #NUM! | 0 | Baseline |
| Male | 5 to 14 | 0 | #NUM! | 0 | Baseline |
| Male | 1 to 4 | 0 | #NUM! | 0 | Baseline |
| Male | 5 to 14 | 0 | #NUM! | 0 | Baseline |
| Male | 1 to 4 | 0 | #NUM! | 0 | Baseline |
| Female | 5 to 14 | 0 | #NUM! | 0 | Baseline |
| Female | 5 to 14 | 0 | #NUM! | 0 | Baseline |
| Male | 5 to 14 | 0 | #NUM! | 0 | Baseline |
| Female | 5 to 14 | 0 | #NUM! | 0 | Baseline |
| Female | 5 to 14 | 3 | 0 | 0 | Baseline |
| Male | 5 to 14 | 0 | #NUM! | 0 | Baseline |
| Male | 5 to 14 | 0 | #NUM! | 0 | Baseline |
| Male | 5 to 14 | 0 | #NUM! | 0 | Baseline |
| Female | 22 to 35 | 0 | #NUM! | 0 | Baseline |
| Female | 15 to 21 | 0 | #NUM! | 0 | Baseline |
| Male | 15 to 21 | 0 | #NUM! | 0 | Baseline |
| Female | 5 to 14 | 0 | #NUM! | 0 | Baseline |
| Male | 5 to 14 | 0 | #NUM! | 0 | Baseline |
| Female | 5 to 14 | 0 | #NUM! | 0 | Baseline |
| Male | 5 to 14 | 0 | #NUM! | 0 | Baseline |
| Female | 5 to 14 | 0 | #NUM! | 0 | Baseline |
| Male | 5 to 14 | 0 | #NUM! | 0 | Baseline |
| Male | 5 to 14 | 0 | #NUM! | 0 | Baseline |
| Male | 5 to 14 | 0 | #NUM! | 0 | Baseline |
| Female | 5 to 14 | 0 | #NUM! | 0 | Baseline |
| Male | 5 to 14 | 0 | #NUM! | 0 | Baseline |
| Female | 5 to 14 | 0 | #NUM! | 0 | Baseline |
| Female | 5 to 14 | 0 | #NUM! | 0 | Baseline |
| Male | 5 to 14 | 0 | #NUM! | 0 | Baseline |
| Male | 5 to 14 | 0 | #NUM! | 0 | Baseline |
| Male | 5 to 14 | 0 | #NUM! | 0 | Baseline |
| Female | 5 to 14 | 0 | #NUM! | 0 | Baseline |
| Male | 5 to 14 | 0 | #NUM! | 0 | Baseline |
| Female | 5 to 14 | 0 | #NUM! | 0 | Baseline |
| Female | 5 to 14 | 0 | #NUM! | 0 | Baseline |
| Female | 5 to 14 | 0 | #NUM! | 0 | Baseline |
| Male | 15 to 21 | 0 | #NUM! | 0 | Baseline |
| Female | 15 to 21 | 0 | #NUM! | 0 | Baseline |
| Male | 5 to 14 | 0 | #NUM! | 0 | Baseline |
| Male | 1 to 4 | 0 | #NUM! | 0 | Baseline |
| Male | 1 to 4 | 0 | #NUM! | 0 | Baseline |
| Male | 1 to 4 | 0 | #NUM! | 0 | Baseline |

| Male | 1 to 4 | 0 | #NUM! | 0 | Baseline |
| --- | --- | --- | --- | --- | --- |
| Male | 1 to 4 | 0 | #NUM! | 0 | Baseline |
| Male | 1 to 4 | 0 | #NUM! | 0 | Baseline |
| Male | 5 to 14 | 0 | #NUM! | 0 | Baseline |
| Male | 1 to 4 | 0 | #NUM! | 0 | Baseline |
| Female | 22 to 35 | 0 | #NUM! | 0 | Baseline |
| Male | 5 to 14 | 0 | #NUM! | 0 | Baseline |
| Male | 5 to 14 | 0 | #NUM! | 0 | Baseline |
| Female | 22 to 35 | 0 | #NUM! | 0 | Baseline |
| Female | 15 to 21 | 0 | #NUM! | 0 | Baseline |
| Female | 5 to 14 | 0 | #NUM! | 0 | Baseline |
| Male | 5 to 14 | 0 | #NUM! | 0 | Baseline |
| Male | 1 to 4 | 0 | #NUM! | 0 | Baseline |
| Male | 1 to 4 | 0 | #NUM! | 0 | Baseline |
| Male | 5 to 14 | 0 | #NUM! | 0 | Baseline |
| Male | 5 to 14 | 0 | #NUM! | 0 | Baseline |
| Male | 1 to 4 | 0 | #NUM! | 0 | Baseline |
| Female | 5 to 14 | 0 | #NUM! | 0 | Baseline |
| Male | 1 to 4 | 0 | #NUM! | 0 | Baseline |
| Male | 1 to 4 | 0 | #NUM! | 0 | Baseline |
| Male | 1 to 4 | 0 | #NUM! | 0 | Baseline |
| Male | 1 to 4 | 0 | #NUM! | 0 | Baseline |
| Male | 1 to 4 | 0 | #NUM! | 0 | Baseline |
| Male | 1 to 4 | 0 | #NUM! | 0 | Baseline |
| Male | 1 to 4 | 0 | #NUM! | 0 | Baseline |
| Male | 1 to 4 | 0 | #NUM! | 0 | Baseline |
| Male | 1 to 4 | 0 | #NUM! | 0 | Baseline |
| Male | 1 to 4 | 0 | #NUM! | 0 | Baseline |
| Male | 1 to 4 | 0 | #NUM! | 0 | Baseline |
| Male | 1 to 4 | 0 | #NUM! | 0 | Baseline |
| Male | 1 to 4 | 0 | #NUM! | 0 | Baseline |
| Male | 1 to 4 | 0 | #NUM! | 0 | Baseline |
| Male | 1 to 4 | 0 | #NUM! | 0 | Baseline |
| Male | 1 to 4 | 0 | #NUM! | 0 | Baseline |
| Male | 1 to 4 | 0 | #NUM! | 0 | Baseline |
| Male | 1 to 4 | 0 | #NUM! | 0 | Baseline |
| Male | 1 to 4 | 0 | #NUM! | 0 | Baseline |
| Male | 1 to 4 | 0 | #NUM! | 0 | Baseline |
| Male | 1 to 4 | 0 | #NUM! | 0 | Baseline |
| Male | 1 to 4 | 0 | #NUM! | 0 | Baseline |
| Male | 1 to 4 | 0 | #NUM! | 0 | Baseline |
| Male | 1 to 4 | 0 | #NUM! | 0 | Baseline |
| Male | 1 to 4 | 0 | #NUM! | 0 | Baseline |
| Male | 1 to 4 | 0 | #NUM! | 0 | Baseline |
| Male | 1 to 4 | 0 | #NUM! | 0 | Baseline |
| Male | 1 to 4 | 0 | #NUM! | 0 | Baseline |
| Male | 1 to 4 | 0 | #NUM! | 0 | Baseline |
| Male | 1 to 4 | 0 | #NUM! | 0 | Baseline |
| Male | 1 to 4 | 0 | #NUM! | 0 | Baseline |
| Male | 1 to 4 | 0 | #NUM! | 0 | Baseline |

| Male | 1 to 4 | 0 | #NUM! | 0 | Baseline |
| --- | --- | --- | --- | --- | --- |
| Male | 1 to 4 | 0 | #NUM! | 0 | Baseline |
| Male | 1 to 4 | 0 | #NUM! | 0 | Baseline |
| Male | 1 to 4 | 0 | #NUM! | 0 | Baseline |
| Male | 1 to 4 | 0 | #NUM! | 0 | Baseline |
| Male | 1 to 4 | 0 | #NUM! | 0 | Baseline |
| Male | 1 to 4 | 0 | #NUM! | 0 | Baseline |
| Male | 1 to 4 | 0 | #NUM! | 0 | Baseline |
| Male | 1 to 4 | 0 | #NUM! | 0 | Baseline |
| Male | 1 to 4 | 0 | #NUM! | 0 | Baseline |
| Male | 1 to 4 | 0 | #NUM! | 0 | Baseline |
| Male | 1 to 4 | 0 | #NUM! | 0 | Baseline |
| Male | 1 to 4 | 0 | #NUM! | 0 | Baseline |
| Male | 1 to 4 | 0 | #NUM! | 0 | Baseline |
| Male | 1 to 4 | 0 | #NUM! | 0 | Baseline |
| Male | 1 to 4 | 0 | #NUM! | 0 | Baseline |
| Male | 1 to 4 | 0 | #NUM! | 0 | Baseline |
| Male | 1 to 4 | 0 | #NUM! | 0 | Baseline |
| Male | 1 to 4 | 0 | #NUM! | 0 | Baseline |
| Male | 1 to 4 | 0 | #NUM! | 0 | Baseline |
| Male | 1 to 4 | 0 | #NUM! | 0 | Baseline |
| Male | 1 to 4 | 0 | #NUM! | 0 | Baseline |
| Male | 1 to 4 | 0 | #NUM! | 0 | Baseline |
| Male | 1 to 4 | 0 | #NUM! | 0 | Baseline |
| Male | 1 to 4 | 0 | #NUM! | 0 | Baseline |
| Male | 1 to 4 | 0 | #NUM! | 0 | Baseline |
| Male | 1 to 4 | 0 | #NUM! | 0 | Baseline |
| Male | 1 to 4 | 0 | #NUM! | 0 | Baseline |
| Male | 1 to 4 | 0 | #NUM! | 0 | Baseline |
| Male | 1 to 4 | 0 | #NUM! | 0 | Baseline |
| Male | 1 to 4 | 0 | #NUM! | 0 | Baseline |
| Male | 1 to 4 | 0 | #NUM! | 0 | Baseline |
| Male | 1 to 4 | 0 | #NUM! | 0 | Baseline |
| Male | 1 to 4 | 0 | #NUM! | 0 | Baseline |
| Male | 1 to 4 | 0 | #NUM! | 0 | Baseline |
| Male | 1 to 4 | 0 | #NUM! | 0 | Baseline |
| Male | 1 to 4 | 0 | #NUM! | 0 | Baseline |
| Male | 1 to 4 | 0 | #NUM! | 0 | Baseline |
| Male | 1 to 4 | 0 | #NUM! | 0 | Baseline |
| Male | 1 to 4 | 0 | #NUM! | 0 | Baseline |
| Male | 1 to 4 | 0 | #NUM! | 0 | Baseline |
| Male | 1 to 4 | 0 | #NUM! | 0 | Baseline |
| Male | 1 to 4 | 0 | #NUM! | 0 | Baseline |
| Male | 1 to 4 | 0 | #NUM! | 0 | Baseline |
| Male | 1 to 4 | 0 | #NUM! | 0 | Baseline |
| Male | 1 to 4 | 0 | #NUM! | 0 | Baseline |
| Male | 1 to 4 | 0 | #NUM! | 0 | Baseline |
| Male | 1 to 4 | 0 | #NUM! | 0 | Baseline |
| Male | 1 to 4 | 0 | #NUM! | 0 | Baseline |
| Male | 1 to 4 | 0 | #NUM! | 0 | Baseline |

| Male | 1 to 4 | 0 | #NUM! | 0 | Baseline |
| --- | --- | --- | --- | --- | --- |
| Male | 1 to 4 | 0 | #NUM! | 0 | Baseline |
| Male | 1 to 4 | 0 | #NUM! | 0 | Baseline |
| Male | 1 to 4 | 0 | #NUM! | 0 | Baseline |
| Male | 1 to 4 | 0 | #NUM! | 0 | Baseline |
| Male | 1 to 4 | 0 | #NUM! | 0 | Baseline |
| Male | 1 to 4 | 0 | #NUM! | 0 | Baseline |
| Male | 1 to 4 | 0 | #NUM! | 0 | Baseline |
| Male | 1 to 4 | 0 | #NUM! | 0 | Baseline |
| Male | 1 to 4 | 0 | #NUM! | 0 | Baseline |
| Male | 1 to 4 | 0 | #NUM! | 0 | Baseline |
| Male | 1 to 4 | 0 | #NUM! | 0 | Baseline |
| Male | 1 to 4 | 0 | #NUM! | 0 | Baseline |
| Male | 1 to 4 | 0 | #NUM! | 0 | Baseline |
| Male | 1 to 4 | 0 | #NUM! | 0 | Baseline |
| Male | 1 to 4 | 0 | #NUM! | 0 | Baseline |
| Male | 1 to 4 | 0 | #NUM! | 0 | Baseline |
| Male | 1 to 4 | 0 | #NUM! | 0 | Baseline |
| Male | 1 to 4 | 0 | #NUM! | 0 | Baseline |
| Male | 1 to 4 | 0 | #NUM! | 0 | Baseline |
| Male | 1 to 4 | 0 | #NUM! | 0 | Baseline |
| Male | 1 to 4 | 0 | #NUM! | 0 | Baseline |
| Male | 1 to 4 | 0 | #NUM! | 0 | Baseline |
| Male | 1 to 4 | 0 | #NUM! | 0 | Baseline |
| Male | 1 to 4 | 0 | #NUM! | 0 | Baseline |
| Male | 1 to 4 | 0 | #NUM! | 0 | Baseline |
| Male | 1 to 4 | 0 | #NUM! | 0 | Baseline |
| Male | 1 to 4 | 0 | #NUM! | 0 | Baseline |
| Male | 1 to 4 | 0 | #NUM! | 0 | Baseline |
| Male | 1 to 4 | 0 | #NUM! | 0 | Baseline |
| Male | 1 to 4 | 0 | #NUM! | 0 | Baseline |
| Male | 1 to 4 | 0 | #NUM! | 0 | Baseline |
| Male | 1 to 4 | 0 | #NUM! | 0 | Baseline |
| Male | 1 to 4 | 0 | #NUM! | 0 | Baseline |
| Male | 1 to 4 | 0 | #NUM! | 0 | Baseline |
| Male | 1 to 4 | 0 | #NUM! | 0 | Baseline |
| Male | 1 to 4 | 0 | #NUM! | 0 | Baseline |
| Male | 1 to 4 | 0 | #NUM! | 0 | Baseline |
| Male | 1 to 4 | 0 | #NUM! | 0 | Baseline |
| Male | 1 to 4 | 0 | #NUM! | 0 | Baseline |
| Male | 1 to 4 | 0 | #NUM! | 0 | Baseline |
| Male | 1 to 4 | 0 | #NUM! | 0 | Baseline |
| Male | 1 to 4 | 0 | #NUM! | 0 | Baseline |
| Male | 1 to 4 | 0 | #NUM! | 0 | Baseline |
| Male | 1 to 4 | 0 | #NUM! | 0 | Baseline |
| Male | 1 to 4 | 0 | #NUM! | 0 | Baseline |
| Male | 1 to 4 | 0 | #NUM! | 0 | Baseline |
| Male | 1 to 4 | 0 | #NUM! | 0 | Baseline |
| Male | 1 to 4 | 0 | #NUM! | 0 | Baseline |
| Male | 1 to 4 | 0 | #NUM! | 0 | Baseline |

| Male | 1 to 4 | 0 | #NUM! | 0 | Baseline |
| --- | --- | --- | --- | --- | --- |
| Male | 1 to 4 | 0 | #NUM! | 0 | Baseline |
| Male | 1 to 4 | 0 | #NUM! | 0 | Baseline |
| Male | 1 to 4 | 0 | #NUM! | 0 | Baseline |
| Male | 1 to 4 | 0 | #NUM! | 0 | Baseline |
| Male | 1 to 4 | 0 | #NUM! | 0 | Baseline |
| Male | 1 to 4 | 0 | #NUM! | 0 | Baseline |
| Male | 1 to 4 | 0 | #NUM! | 0 | Baseline |
| Male | 1 to 4 | 0 | #NUM! | 0 | Baseline |
| Male | 1 to 4 | 0 | #NUM! | 0 | Baseline |
| Male | 1 to 4 | 0 | #NUM! | 0 | Baseline |
| Male | 1 to 4 | 0 | #NUM! | 0 | Baseline |
| Male | 1 to 4 | 0 | #NUM! | 0 | Baseline |
| Male | 1 to 4 | 0 | #NUM! | 0 | Baseline |
| Male | 1 to 4 | 0 | #NUM! | 0 | Baseline |
| Male | 1 to 4 | 0 | #NUM! | 0 | Baseline |
| Male | 1 to 4 | 0 | #NUM! | 0 | Baseline |
| Male | 1 to 4 | 0 | #NUM! | 0 | Baseline |
| Male | 1 to 4 | 0 | #NUM! | 0 | Baseline |
| Male | 1 to 4 | 0 | #NUM! | 0 | Baseline |
| Male | 1 to 4 | 0 | #NUM! | 0 | Baseline |
| Male | 1 to 4 | 0 | #NUM! | 0 | Baseline |
| Male | 1 to 4 | 0 | #NUM! | 0 | Baseline |
| Male | 1 to 4 | 0 | #NUM! | 0 | Baseline |
| Male | 1 to 4 | 0 | #NUM! | 0 | Baseline |
| Male | 1 to 4 | 0 | #NUM! | 0 | Baseline |
| Male | 1 to 4 | 0 | #NUM! | 0 | Baseline |
| Male | 1 to 4 | 0 | #NUM! | 0 | Baseline |
| Male | 1 to 4 | 0 | #NUM! | 0 | Baseline |
| Male | 1 to 4 | 0 | #NUM! | 0 | Baseline |
| Male | 1 to 4 | 0 | #NUM! | 0 | Baseline |
| Male | 1 to 4 | 0 | #NUM! | 0 | Baseline |
| Male | 1 to 4 | 0 | #NUM! | 0 | Baseline |
| Male | 1 to 4 | 0 | #NUM! | 0 | Baseline |
| Male | 1 to 4 | 0 | #NUM! | 0 | Baseline |
| Male | 1 to 4 | 0 | #NUM! | 0 | Baseline |
| Male | 1 to 4 | 0 | #NUM! | 0 | Baseline |
| Male | 1 to 4 | 0 | #NUM! | 0 | Baseline |
| Male | 1 to 4 | 0 | #NUM! | 0 | Baseline |
| Male | 1 to 4 | 0 | #NUM! | 0 | Baseline |
| Male | 1 to 4 | 0 | #NUM! | 0 | Baseline |
| Male | 1 to 4 | 0 | #NUM! | 0 | Baseline |
| Male | 1 to 4 | 0 | #NUM! | 0 | Baseline |
| Male | 1 to 4 | 0 | #NUM! | 0 | Baseline |
| Male | 1 to 4 | 0 | #NUM! | 0 | Baseline |
| Male | 1 to 4 | 0 | #NUM! | 0 | Baseline |
| Male | 1 to 4 | 0 | #NUM! | 0 | Baseline |
| Male | 1 to 4 | 0 | #NUM! | 0 | Baseline |
| Male | 1 to 4 | 0 | #NUM! | 0 | Baseline |
| Male | 1 to 4 | 0 | #NUM! | 0 | Baseline |

| Male | 1 to 4 | 0 | #NUM! | 0 | Baseline |
| --- | --- | --- | --- | --- | --- |
| Male | 1 to 4 | 0 | #NUM! | 0 | Baseline |
| Male | 1 to 4 | 0 | #NUM! | 0 | Baseline |
| Male | 1 to 4 | 0 | #NUM! | 0 | Baseline |
| Male | 1 to 4 | 0 | #NUM! | 0 | Baseline |
| Male | 1 to 4 | 0 | #NUM! | 0 | Baseline |
| Male | 1 to 4 | 0 | #NUM! | 0 | Baseline |
| Male | 1 to 4 | 0 | #NUM! | 0 | Baseline |
| Male | 1 to 4 | 0 | #NUM! | 0 | Baseline |
| Male | 1 to 4 | 0 | #NUM! | 0 | Baseline |
| Male | 1 to 4 | 0 | #NUM! | 0 | Baseline |
| Male | 1 to 4 | 0 | #NUM! | 0 | Baseline |
| Male | 1 to 4 | 0 | #NUM! | 0 | Baseline |
| Male | 1 to 4 | 0 | #NUM! | 0 | Baseline |
| Male | 1 to 4 | 0 | #NUM! | 0 | Baseline |
| Male | 1 to 4 | 0 | #NUM! | 0 | Baseline |
| Male | 1 to 4 | 0 | #NUM! | 0 | Baseline |
| Male | 1 to 4 | 0 | #NUM! | 0 | Baseline |
| Male | 1 to 4 | 0 | #NUM! | 0 | Baseline |
| Male | 1 to 4 | 0 | #NUM! | 0 | Baseline |
| Male | 1 to 4 | 0 | #NUM! | 0 | Baseline |
| Male | 1 to 4 | 0 | #NUM! | 0 | Baseline |
| Male | 1 to 4 | 0 | #NUM! | 0 | Baseline |
| Male | 1 to 4 | 0 | #NUM! | 0 | Baseline |
| Male | 1 to 4 | 0 | #NUM! | 0 | Baseline |
| Male | 1 to 4 | 0 | #NUM! | 0 | Baseline |
| Male | 1 to 4 | 0 | #NUM! | 0 | Baseline |
| Male | 1 to 4 | 0 | #NUM! | 0 | Baseline |
| Male | 1 to 4 | 0 | #NUM! | 0 | Baseline |
| Male | 1 to 4 | 0 | #NUM! | 0 | Baseline |
| Male | 1 to 4 | 0 | #NUM! | 0 | Baseline |
| Male | 1 to 4 | 0 | #NUM! | 0 | Baseline |
| Male | 1 to 4 | 0 | #NUM! | 0 | Baseline |
| Male | 1 to 4 | 0 | #NUM! | 0 | Baseline |
| Male | 1 to 4 | 0 | #NUM! | 0 | Baseline |
| Male | 1 to 4 | 0 | #NUM! | 0 | Baseline |
| Male | 1 to 4 | 0 | #NUM! | 0 | Baseline |
| Male | 1 to 4 | 0 | #NUM! | 0 | Baseline |
| Male | 1 to 4 | 0 | #NUM! | 0 | Baseline |
| Male | 1 to 4 | 0 | #NUM! | 0 | Baseline |
| Male | 1 to 4 | 0 | #NUM! | 0 | Baseline |
| Male | 1 to 4 | 0 | #NUM! | 0 | Baseline |
| Male | 1 to 4 | 0 | #NUM! | 0 | Baseline |
| Male | 1 to 4 | 0 | #NUM! | 0 | Baseline |
| Male | 1 to 4 | 0 | #NUM! | 0 | Baseline |
| Male | 1 to 4 | 0 | #NUM! | 0 | Baseline |
| Male | 1 to 4 | 0 | #NUM! | 0 | Baseline |
| Male | 1 to 4 | 0 | #NUM! | 0 | Baseline |
| Male | 1 to 4 | 0 | #NUM! | 0 | Baseline |
| Male | 1 to 4 | 0 | #NUM! | 0 | Baseline |

| Male | 1 to 4 | 0 | #NUM! | 0 | Baseline |
| --- | --- | --- | --- | --- | --- |
| Male | 1 to 4 | 0 | #NUM! | 0 | Baseline |
| Male | 1 to 4 | 0 | #NUM! | 0 | Baseline |
| Male | 1 to 4 | 0 | #NUM! | 0 | Baseline |
| Male | 1 to 4 | 0 | #NUM! | 0 | Baseline |
| Male | 1 to 4 | 0 | #NUM! | 0 | Baseline |
| Male | 1 to 4 | 0 | #NUM! | 0 | Baseline |
| Male | 1 to 4 | 0 | #NUM! | 0 | Baseline |
| Male | 1 to 4 | 0 | #NUM! | 0 | Baseline |
| Male | 1 to 4 | 0 | #NUM! | 0 | Baseline |
| Male | 1 to 4 | 0 | #NUM! | 0 | Baseline |
| Male | 1 to 4 | 0 | #NUM! | 0 | Baseline |
| Male | 1 to 4 | 0 | #NUM! | 0 | Baseline |
| Male | 1 to 4 | 0 | #NUM! | 0 | Baseline |
| Male | 1 to 4 | 0 | #NUM! | 0 | Baseline |
| Male | 1 to 4 | 0 | #NUM! | 0 | Baseline |
| Male | 1 to 4 | 0 | #NUM! | 0 | Baseline |
| Male | 1 to 4 | 0 | #NUM! | 0 | Baseline |
| Male | 1 to 4 | 0 | #NUM! | 0 | Baseline |
| Male | 1 to 4 | 0 | #NUM! | 0 | Baseline |
| Male | 1 to 4 | 0 | #NUM! | 0 | Baseline |
| Male | 1 to 4 | 0 | #NUM! | 0 | Baseline |
| Male | 1 to 4 | 0 | #NUM! | 0 | Baseline |
| Male | 1 to 4 | 0 | #NUM! | 0 | Baseline |
| Male | 1 to 4 | 0 | #NUM! | 0 | Baseline |
| Male | 1 to 4 | 0 | #NUM! | 0 | Baseline |
| Male | 1 to 4 | 0 | #NUM! | 0 | Baseline |
| Male | 1 to 4 | 0 | #NUM! | 0 | Baseline |
| Male | 1 to 4 | 0 | #NUM! | 0 | Baseline |
| Male | 1 to 4 | 0 | #NUM! | 0 | Baseline |
| Male | 1 to 4 | 0 | #NUM! | 0 | Baseline |
| Male | 1 to 4 | 0 | #NUM! | 0 | Baseline |
| Male | 1 to 4 | 0 | #NUM! | 0 | Baseline |
| Male | 1 to 4 | 0 | #NUM! | 0 | Baseline |
| Male | 1 to 4 | 0 | #NUM! | 0 | Baseline |
| Male | 1 to 4 | 0 | #NUM! | 0 | Baseline |
| Male | 1 to 4 | 0 | #NUM! | 0 | Baseline |
| Male | 1 to 4 | 0 | #NUM! | 0 | Baseline |
| Male | 1 to 4 | 0 | #NUM! | 0 | Baseline |
| Male | 1 to 4 | 0 | #NUM! | 0 | Baseline |
| Male | 1 to 4 | 0 | #NUM! | 0 | Baseline |
| Male | 1 to 4 | 0 | #NUM! | 0 | Baseline |
| Male | 1 to 4 | 0 | #NUM! | 0 | Baseline |
| Male | 1 to 4 | 0 | #NUM! | 0 | Baseline |
| Male | 1 to 4 | 0 | #NUM! | 0 | Baseline |
| Male | 1 to 4 | 0 | #NUM! | 0 | Baseline |
| Male | 1 to 4 | 0 | #NUM! | 0 | Baseline |
| Male | 1 to 4 | 0 | #NUM! | 0 | Baseline |
| Male | 1 to 4 | 0 | #NUM! | 0 | Baseline |
| Male | 1 to 4 | 0 | #NUM! | 0 | Baseline |

| Male | 1 to 4 | 0 | #NUM! | 0 | Baseline |
| --- | --- | --- | --- | --- | --- |
| Male | 1 to 4 | 0 | #NUM! | 0 | Baseline |
| Male | 1 to 4 | 0 | #NUM! | 0 | Baseline |
| Male | 1 to 4 | 0 | #NUM! | 0 | Baseline |
| Male | 1 to 4 | 0 | #NUM! | 0 | Baseline |
| Male | 1 to 4 | 0 | #NUM! | 0 | Baseline |
| Male | 1 to 4 | 0 | #NUM! | 0 | Baseline |
| Male | 1 to 4 | 0 | #NUM! | 0 | Baseline |
| Male | 1 to 4 | 0 | #NUM! | 0 | Baseline |
| Male | 1 to 4 | 0 | #NUM! | 0 | Baseline |
| Male | 1 to 4 | 0 | #NUM! | 0 | Baseline |
| Male | 1 to 4 | 0 | #NUM! | 0 | Baseline |
| Male | 1 to 4 | 0 | #NUM! | 0 | Baseline |
| Male | 1 to 4 | 0 | #NUM! | 0 | Baseline |
| Male | 1 to 4 | 0 | #NUM! | 0 | Baseline |
| Male | 1 to 4 | 0 | #NUM! | 0 | Baseline |
| Male | 1 to 4 | 0 | #NUM! | 0 | Baseline |
| Male | 1 to 4 | 0 | #NUM! | 0 | Baseline |
| Male | 1 to 4 | 0 | #NUM! | 0 | Baseline |
| Male | 1 to 4 | 0 | #NUM! | 0 | Baseline |
| Male | 1 to 4 | 0 | #NUM! | 0 | Baseline |
| Male | 1 to 4 | 0 | #NUM! | 0 | Baseline |
| Male | 1 to 4 | 0 | #NUM! | 0 | Baseline |
| Male | 1 to 4 | 0 | #NUM! | 0 | Baseline |
| Male | 1 to 4 | 0 | #NUM! | 0 | Baseline |
| Male | 1 to 4 | 0 | #NUM! | 0 | Baseline |
| Male | 1 to 4 | 0 | #NUM! | 0 | Baseline |
| Male | 1 to 4 | 0 | #NUM! | 0 | Baseline |
| Male | 1 to 4 | 0 | #NUM! | 0 | Baseline |
| Male | 1 to 4 | 0 | #NUM! | 0 | Baseline |
| Male | 1 to 4 | 0 | #NUM! | 0 | Baseline |
| Male | 1 to 4 | 0 | #NUM! | 0 | Baseline |
| Male | 1 to 4 | 0 | #NUM! | 0 | Baseline |
| Male | 1 to 4 | 0 | #NUM! | 0 | Baseline |
| Male | 1 to 4 | 0 | #NUM! | 0 | Baseline |
| Male | 1 to 4 | 0 | #NUM! | 0 | Baseline |
| Male | 1 to 4 | 0 | #NUM! | 0 | Baseline |
| Male | 1 to 4 | 0 | #NUM! | 0 | Baseline |
| Male | 1 to 4 | 0 | #NUM! | 0 | Baseline |
| Male | 1 to 4 | 0 | #NUM! | 0 | Baseline |
| Male | 1 to 4 | 0 | #NUM! | 0 | Baseline |
| Male | 1 to 4 | 0 | #NUM! | 0 | Baseline |
| Male | 1 to 4 | 0 | #NUM! | 0 | Baseline |
| Male | 1 to 4 | 0 | #NUM! | 0 | Baseline |
| Male | 1 to 4 | 0 | #NUM! | 0 | Baseline |
| Male | 1 to 4 | 0 | #NUM! | 0 | Baseline |
| Male | 1 to 4 | 0 | #NUM! | 0 | Baseline |
| Male | 1 to 4 | 0 | #NUM! | 0 | Baseline |
| Male | 1 to 4 | 0 | #NUM! | 0 | Baseline |
| Male | 1 to 4 | 0 | #NUM! | 0 | Baseline |

| Male | 1 to 4 | 0 | #NUM! | 0 | Baseline |
| --- | --- | --- | --- | --- | --- |
| Male | 1 to 4 | 0 | #NUM! | 0 | Baseline |
| Male | 1 to 4 | 0 | #NUM! | 0 | Baseline |
| Male | 1 to 4 | 0 | #NUM! | 0 | Baseline |
| Male | 1 to 4 | 0 | #NUM! | 0 | Baseline |
| Male | 1 to 4 | 0 | #NUM! | 0 | Baseline |
| Male | 1 to 4 | 0 | #NUM! | 0 | Baseline |
| Male | 1 to 4 | 0 | #NUM! | 0 | Baseline |
| Male | 1 to 4 | 0 | #NUM! | 0 | Baseline |
| Male | 1 to 4 | 0 | #NUM! | 0 | Baseline |
| Male | 15 to 21 | 0 | #NUM! | 0 | Baseline |
| Male | 1 to 4 | 0 | #NUM! | 0 | Baseline |
| Male | 1 to 4 | 0 | #NUM! | 0 | Baseline |
| Male | 1 to 4 | 0 | #NUM! | 0 | Baseline |
| Male | 1 to 4 | 0 | #NUM! | 0 | Baseline |
| Male | 1 to 4 | 0 | #NUM! | 0 | Baseline |
| Male | 1 to 4 | 0 | #NUM! | 0 | Baseline |
| Male | 1 to 4 | 0 | #NUM! | 0 | Baseline |
| Male | 1 to 4 | 0 | #NUM! | 0 | Baseline |
| Male | 1 to 4 | 0 | #NUM! | 0 | Baseline |
| Male | 1 to 4 | 0 | #NUM! | 0 | Baseline |
| Male | 1 to 4 | 0 | #NUM! | 0 | Baseline |
| Male | 1 to 4 | 0 | #NUM! | 0 | Baseline |
| Male | 1 to 4 | 0 | #NUM! | 0 | Baseline |
| Male | 1 to 4 | 0 | #NUM! | 0 | Baseline |
| Male | 1 to 4 | 0 | #NUM! | 0 | Baseline |
| Male | 1 to 4 | 0 | #NUM! | 0 | Baseline |
| Male | 1 to 4 | 0 | #NUM! | 0 | Baseline |
| Male | 1 to 4 | 0 | #NUM! | 0 | Baseline |
| Male | 1 to 4 | 0 | #NUM! | 0 | Baseline |
| Male | 1 to 4 | 0 | #NUM! | 0 | Baseline |
| Male | 1 to 4 | 0 | #NUM! | 0 | Baseline |
| Male | 1 to 4 | 0 | #NUM! | 0 | Baseline |
| Male | 1 to 4 | 0 | #NUM! | 0 | Baseline |
| Male | 1 to 4 | 0 | #NUM! | 0 | Baseline |
| Male | 1 to 4 | 0 | #NUM! | 0 | Baseline |
| Male | 1 to 4 | 0 | #NUM! | 0 | Baseline |
| Male | 1 to 4 | 0 | #NUM! | 0 | Baseline |
| Male | 1 to 4 | 0 | #NUM! | 0 | Baseline |
| Male | 1 to 4 | 0 | #NUM! | 0 | Baseline |
| Male | 1 to 4 | 0 | #NUM! | 0 | Baseline |
| Male | 1 to 4 | 0 | #NUM! | 0 | Baseline |
| Male | 1 to 4 | 0 | #NUM! | 0 | Baseline |
| Male | 1 to 4 | 0 | #NUM! | 0 | Baseline |
| Male | 1 to 4 | 0 | #NUM! | 0 | Baseline |
| Male | 1 to 4 | 0 | #NUM! | 0 | Baseline |
| Male | 1 to 4 | 0 | #NUM! | 0 | Baseline |
| Male | 1 to 4 | 0 | #NUM! | 0 | Baseline |
| Male | 1 to 4 | 0 | #NUM! | 0 | Baseline |
| Male | 1 to 4 | 0 | #NUM! | 0 | Baseline |

| Male | 1 to 4 | 0 | #NUM! | 0 | Baseline |
| --- | --- | --- | --- | --- | --- |
| Male | 1 to 4 | 0 | #NUM! | 0 | Baseline |
| Male | 1 to 4 | 0 | #NUM! | 0 | Baseline |
| Male | 1 to 4 | 0 | #NUM! | 0 | Baseline |
| Male | 1 to 4 | 0 | #NUM! | 0 | Baseline |
| Male | 1 to 4 | 0 | #NUM! | 0 | Baseline |
| Male | 1 to 4 | 0 | #NUM! | 0 | Baseline |
| Male | 1 to 4 | 0 | #NUM! | 0 | Baseline |
| Male | 1 to 4 | 0 | #NUM! | 0 | Baseline |
| Male | 1 to 4 | 0 | #NUM! | 0 | Baseline |
| Male | 1 to 4 | 0 | #NUM! | 0 | Baseline |
| Male | 1 to 4 | 0 | #NUM! | 0 | Baseline |
| Male | 5 to 14 | 0 | #NUM! | 0 | Baseline |
| Female | 5 to 14 | 0 | #NUM! | 0 | Baseline |
| Female | 15 to 21 | 0 | #NUM! | 0 | Baseline |
| Female | 15 to 21 | 0 | #NUM! | 0 | Baseline |
| Female | 15 to 21 | 0 | #NUM! | 0 | Baseline |
| Female | 35+ | 0 | #NUM! | 0 | Baseline |
| Female | 35+ | 0 | #NUM! | 0 | Baseline |
| Female | 22 to 35 | 0 | #NUM! | 0 | Baseline |
| Female | 1 to 4 | 0 | #NUM! | 0 | Baseline |
| Male | 1 to 4 | 0 | #NUM! | 0 | Baseline |
| Female | 22 to 35 | 0 | #NUM! | 0 | Baseline |
| Male | 1 to 4 | 0 | #NUM! | 0 | Baseline |
| Female | 1 to 4 | 0 | #NUM! | 0 | Baseline |
| Male | 1 to 4 | 0 | #NUM! | 0 | Baseline |
| Female | 1 to 4 | 0 | #NUM! | 0 | Baseline |
| Female | 5 to 14 | 0 | #NUM! | 0 | Baseline |
| Female | 5 to 14 | 0 | #NUM! | 0 | Baseline |
| Female | 15 to 21 | 0 | #NUM! | 0 | Baseline |
| Male | 5 to 14 | 0 | #NUM! | 0 | Baseline |
| Male | 5 to 14 | 0 | #NUM! | 0 | Baseline |
| Male | 15 to 21 | 0 | #NUM! | 0 | Baseline |
| Male | 15 to 21 | 0 | #NUM! | 0 | Baseline |
| Female | 15 to 21 | 0 | #NUM! | 0 | Baseline |
| Male | 5 to 14 | 0 | #NUM! | 0 | Baseline |
| Female | 15 to 21 | 0 | #NUM! | 0 | Baseline |
| Female | 15 to 21 | 0 | #NUM! | 0 | Baseline |
| Male | 5 to 14 | 0 | #NUM! | 0 | Baseline |
| Female | 15 to 21 | 0 | #NUM! | 0 | Baseline |
| Male | 15 to 21 | 0 | #NUM! | 0 | Baseline |
| Male | 15 to 21 | 0 | #NUM! | 0 | Baseline |
| Female | 15 to 21 | 0 | #NUM! | 0 | Baseline |
| Male | 5 to 14 | 0 | #NUM! | 0 | Baseline |
| Male | 15 to 21 | 0 | #NUM! | 0 | Baseline |
| Female | 22 to 35 | 0 | #NUM! | 0 | Baseline |
| Female | 35+ | 0 | #NUM! | 0 | Baseline |
| Male | 35+ | 0 | #NUM! | 0 | Baseline |
| Male | 35+ | 0 | #NUM! | 0 | Baseline |
| Female | 1 to 4 | 0 | #NUM! | 0 | Baseline |

| Male | 35+ | 0 | #NUM! | 0 | Baseline |
| --- | --- | --- | --- | --- | --- |
| Female | 35+ | 0 | #NUM! | 0 | Baseline |
| Female | 22 to 35 | 0 | #NUM! | 0 | Baseline |
| Female | 22 to 35 | 0 | #NUM! | 0 | Baseline |
| Female | 1 to 4 | 0 | #NUM! | 0 | Baseline |
| Female | 5 to 14 | 0 | #NUM! | 0 | Baseline |
| Male | 15 to 21 | 0 | #NUM! | 0 | Baseline |
| Female | 15 to 21 | 0 | #NUM! | 0 | Baseline |
| Male | 5 to 14 | 0 | #NUM! | 0 | Baseline |
| Female | 15 to 21 | 0 | #NUM! | 0 | Baseline |
| Female | 5 to 14 | 0 | #NUM! | 0 | Baseline |
| Male | 1 to 4 | 0 | #NUM! | 0 | Baseline |
| Male | 15 to 21 | 0 | #NUM! | 0 | Baseline |
| Male | 5 to 14 | 0 | #NUM! | 0 | Baseline |
| Male | 5 to 14 | 0 | #NUM! | 0 | Baseline |
| Male | 5 to 14 | 0 | #NUM! | 0 | Baseline |
| Male | 5 to 14 | 0 | #NUM! | 0 | Baseline |
| Female | 15 to 21 | 0 | #NUM! | 0 | Baseline |
| Male | 5 to 14 | 0 | #NUM! | 0 | Baseline |
| Female | 15 to 21 | 0 | #NUM! | 0 | Baseline |
| Male | 5 to 14 | 0 | #NUM! | 0 | Baseline |
| Female | 5 to 14 | 0 | #NUM! | 0 | Baseline |
| Female | 5 to 14 | 0 | #NUM! | 0 | Baseline |
| Female | 15 to 21 | 0 | #NUM! | 0 | Baseline |
| Male | 5 to 14 | 0 | #NUM! | 0 | Baseline |
| Male | 5 to 14 | 0 | #NUM! | 0 | Baseline |
| Female | 22 to 35 | 0 | #NUM! | 0 | Baseline |
| Female | 22 to 35 | 0 | #NUM! | 0 | Baseline |
| Male | 22 to 35 | 0 | #NUM! | 0 | Baseline |
| Male | 35+ | 0 | #NUM! | 0 | Baseline |
| Female | 22 to 35 | 0 | #NUM! | 0 | Baseline |
| Male | 1 to 4 | 0 | #NUM! | 0 | Baseline |
| Male | 22 to 35 | 0 | #NUM! | 0 | Baseline |
| Male | 35+ | 0 | #NUM! | 0 | Baseline |
| Male | 35+ | 0 | #NUM! | 0 | Baseline |
| Male | 35+ | 0 | #NUM! | 0 | Baseline |
| Female | 1 to 4 | 0 | #NUM! | 0 | Baseline |
| Female | 35+ | 0 | #NUM! | 0 | Baseline |
| Male | 35+ | 0 | #NUM! | 0 | Baseline |
| Male | 1 to 4 | 0 | #NUM! | 0 | Baseline |
| Female | 22 to 35 | 0 | #NUM! | 0 | Baseline |
| Female | 35+ | 0 | #NUM! | 0 | Baseline |
| Male | 35+ | 0 | #NUM! | 0 | Baseline |
| Female | 35+ | 0 | #NUM! | 0 | Baseline |
| Male | 22 to 35 | 0 | #NUM! | 0 | Baseline |
| Female | 35+ | 0 | #NUM! | 0 | Baseline |
| Male | 35+ | 0 | #NUM! | 0 | Baseline |
| Female | 5 to 14 | 0 | #NUM! | 0 | Baseline |
| Female | 5 to 14 | 0 | #NUM! | 0 | Baseline |
| Female | 35+ | 0 | #NUM! | 0 | Baseline |

| Female | 1 to 4 | 0 | #NUM! | 0 | Baseline |
| --- | --- | --- | --- | --- | --- |
| Female | 5 to 14 | 0 | #NUM! | 0 | Baseline |
| Female | 22 to 35 | 0 | #NUM! | 0 | Baseline |
| Female | 35+ | 0 | #NUM! | 0 | Baseline |
| Male | 5 to 14 | 0 | #NUM! | 0 | Baseline |
| Female | 5 to 14 | 0 | #NUM! | 0 | Baseline |
| Male | 5 to 14 | 0 | #NUM! | 0 | Baseline |
| Female | 15 to 21 | 0 | #NUM! | 0 | Baseline |
| Male | 5 to 14 | 0 | #NUM! | 0 | Baseline |
| Male | 1 to 4 | 0 | #NUM! | 0 | Baseline |
| Male | 1 to 4 | 0 | #NUM! | 0 | Baseline |
| Male | 1 to 4 | 0 | #NUM! | 0 | Baseline |
| Female | 35+ | 0 | #NUM! | 0 | Baseline |
| Female | 1 to 4 | 0 | #NUM! | 0 | Baseline |
| Male | 35+ | 0 | #NUM! | 0 | Baseline |
| Male | 5 to 14 | 0 | #NUM! | 0 | Baseline |
| Female | 35+ | 0 | #NUM! | 0 | Baseline |
| Male | 5 to 14 | 0 | #NUM! | 0 | Baseline |
| Male | 15 to 21 | 0 | #NUM! | 0 | Baseline |
| Female | 35+ | 0 | #NUM! | 0 | Baseline |
| Female | 35+ | 0 | #NUM! | 0 | Baseline |
| Male | 15 to 21 | 0 | #NUM! | 0 | Baseline |
| Male | 5 to 14 | 0 | #NUM! | 0 | Baseline |
| Female | 22 to 35 | 0 | #NUM! | 0 | Baseline |
| Female | 35+ | 0 | #NUM! | 0 | Baseline |
| Female | 5 to 14 | 0 | #NUM! | 0 | Baseline |
| Male | 35+ | 0 | #NUM! | 0 | Baseline |
| Female | 15 to 21 | 0 | #NUM! | 0 | Baseline |
| Female | 15 to 21 | 0 | #NUM! | 0 | Baseline |
| Male | 15 to 21 | 0 | #NUM! | 0 | Baseline |
| Female | 15 to 21 | 0 | #NUM! | 0 | Baseline |
| Female | 15 to 21 | 0 | #NUM! | 0 | Baseline |
| Male | 22 to 35 | 0 | #NUM! | 0 | Baseline |
| Female | 22 to 35 | 0 | #NUM! | 0 | Baseline |
| Male | 22 to 35 | 0 | #NUM! | 0 | Baseline |
| Female | 22 to 35 | 0 | #NUM! | 0 | Baseline |
| Male | 22 to 35 | 0 | #NUM! | 0 | Baseline |
| Male | 22 to 35 | 0 | #NUM! | 0 | Baseline |
| Male | 22 to 35 | 0 | #NUM! | 0 | Baseline |
| Female | 22 to 35 | 0 | #NUM! | 0 | Baseline |
| Female | 22 to 35 | 0 | #NUM! | 0 | Baseline |
| Female | 15 to 21 | 0 | #NUM! | 0 | Baseline |
| Female | 15 to 21 | 0 | #NUM! | 0 | Baseline |
| Male | 15 to 21 | 0 | #NUM! | 0 | Baseline |
| Female | 1 to 4 | 0 | #NUM! | 0 | Baseline |
| Male | 15 to 21 | 0 | #NUM! | 0 | Baseline |
| Female | 15 to 21 | 0 | #NUM! | 0 | Baseline |
| Female | 15 to 21 | 0 | #NUM! | 0 | Baseline |
| Male | 15 to 21 | 0 | #NUM! | 0 | Baseline |
| Female | 15 to 21 | 0 | #NUM! | 0 | Baseline |

| Male | 35+ | 0 | #NUM! | 0 | Baseline |
| --- | --- | --- | --- | --- | --- |
| Female | 5 to 14 | 0 | #NUM! | 0 | Baseline |
| Female | 35+ | 0 | #NUM! | 0 | Baseline |
| Male | 5 to 14 | 0 | #NUM! | 0 | Baseline |
| Female | 35+ | 0 | #NUM! | 0 | Baseline |
| Male | 5 to 14 | 0 | #NUM! | 0 | Baseline |
| Male | 5 to 14 | 0 | #NUM! | 0 | Baseline |
| Male | 15 to 21 | 0 | #NUM! | 0 | Baseline |
| Female | 15 to 21 | 0 | #NUM! | 0 | Baseline |
| Female | 1 to 4 | 0 | #NUM! | 0 | Baseline |
| Male | 5 to 14 | 0 | #NUM! | 0 | Baseline |
| Female | 5 to 14 | 0 | #NUM! | 0 | Baseline |
| Female | 5 to 14 | 0 | #NUM! | 0 | Baseline |
| Male | 35+ | 0 | #NUM! | 0 | Baseline |
| Female | 5 to 14 | 0 | #NUM! | 0 | Baseline |
| Male | 35+ | 0 | #NUM! | 0 | Baseline |
| Male | 5 to 14 | 0 | #NUM! | 0 | Baseline |
| Male | 5 to 14 | 0 | #NUM! | 0 | Baseline |
| Male | 5 to 14 | 0 | #NUM! | 0 | Baseline |
| Female | 35+ | 0 | #NUM! | 0 | Baseline |
| Male | 5 to 14 | 0 | #NUM! | 0 | Baseline |
| Female | 5 to 14 | 0 | #NUM! | 0 | Baseline |
| Male | 15 to 21 | 0 | #NUM! | 0 | Baseline |
| Female | 5 to 14 | 0 | #NUM! | 0 | Baseline |
| Male | 5 to 14 | 0 | #NUM! | 0 | Baseline |
| Male | 15 to 21 | 0 | #NUM! | 0 | Baseline |
| Female | 5 to 14 | 0 | #NUM! | 0 | Baseline |
| Female | 5 to 14 | 0 | #NUM! | 0 | Baseline |
| Female | 15 to 21 | 0 | #NUM! | 0 | Baseline |
| Male | 5 to 14 | 0 | #NUM! | 0 | Baseline |
| Male | 5 to 14 | 0 | #NUM! | 0 | Baseline |
| Male | 5 to 14 | 0 | #NUM! | 0 | Baseline |
| Male | 5 to 14 | 0 | #NUM! | 0 | Baseline |
| Male | 5 to 14 | 0 | #NUM! | 0 | Baseline |
| Male | 22 to 35 | 0 | #NUM! | 0 | Baseline |
| Male | 35+ | 0 | #NUM! | 0 | Baseline |
| Female | 35+ | 0 | #NUM! | 0 | Baseline |
| Male | 35+ | 0 | #NUM! | 0 | Baseline |
| Male | 35+ | 0 | #NUM! | 0 | Baseline |
| Female | 35+ | 0 | #NUM! | 0 | Baseline |
| Female | 1 to 4 | 0 | #NUM! | 0 | Baseline |
| Male | 1 to 4 | 0 | #NUM! | 0 | Baseline |
| Female | 5 to 14 | 0 | #NUM! | 0 | Baseline |
| Female | 5 to 14 | 0 | #NUM! | 0 | Baseline |
| Female | 5 to 14 | 0 | #NUM! | 0 | Baseline |
| Male | 1 to 4 | 0 | #NUM! | 0 | Baseline |
| Female | 15 to 21 | 0 | #NUM! | 0 | Baseline |
| Female | 1 to 4 | 0 | #NUM! | 0 | Baseline |
| Female | 15 to 21 | 0 | #NUM! | 0 | Baseline |
| Male | 5 to 14 | 0 | #NUM! | 0 | Baseline |

| Male | 15 to 21 | 0 | #NUM! | 0 | Baseline |
| --- | --- | --- | --- | --- | --- |
| Male | 15 to 21 | 0 | #NUM! | 0 | Baseline |
| Female | 22 to 35 | 0 | #NUM! | 0 | Baseline |
| Male | 35+ | 0 | #NUM! | 0 | Baseline |
| Female | 35+ | 0 | #NUM! | 0 | Baseline |
| Female | 22 to 35 | 0 | #NUM! | 0 | Baseline |
| Female | 35+ | 0 | #NUM! | 0 | Baseline |
| Male | 35+ | 0 | #NUM! | 0 | Baseline |
| Female | 22 to 35 | 0 | #NUM! | 0 | Baseline |
| Male | 35+ | 0 | #NUM! | 0 | Baseline |
| Female | 22 to 35 | 0 | #NUM! | 0 | Baseline |
| Female | 1 to 4 | 0 | #NUM! | 0 | Baseline |
| Female | 1 to 4 | 0 | #NUM! | 0 | Baseline |
| Female | 1 to 4 | 0 | #NUM! | 0 | Baseline |
| Male | 5 to 14 | 0 | #NUM! | 0 | Baseline |
| Male | 1 to 4 | 0 | #NUM! | 0 | Baseline |
| Male | 1 to 4 | 0 | #NUM! | 0 | Baseline |
| Female | 1 to 4 | 0 | #NUM! | 0 | Baseline |
| Male | 1 to 4 | 0 | #NUM! | 0 | Baseline |
| Male | 15 to 21 | 0 | #NUM! | 0 | Baseline |
| Male | 15 to 21 | 0 | #NUM! | 0 | Baseline |
| Female | 5 to 14 | 0 | #NUM! | 0 | Baseline |
| Male | 15 to 21 | 0 | #NUM! | 0 | Baseline |
| Male | 15 to 21 | 0 | #NUM! | 0 | Baseline |
| Male | 5 to 14 | 0 | #NUM! | 0 | Baseline |
| Male | 15 to 21 | 0 | #NUM! | 0 | Baseline |
| Female | 15 to 21 | 0 | #NUM! | 0 | Baseline |
| Male | 5 to 14 | 0 | #NUM! | 0 | Baseline |
| Male | 15 to 21 | 0 | #NUM! | 0 | Baseline |
| Male | 5 to 14 | 0 | #NUM! | 0 | Baseline |
| Female | 5 to 14 | 0 | #NUM! | 0 | Baseline |
| Male | 5 to 14 | 0 | #NUM! | 0 | Baseline |
| Female | 5 to 14 | 0 | #NUM! | 0 | Baseline |
| Male | 5 to 14 | 0 | #NUM! | 0 | Baseline |
| Female | 15 to 21 | 0 | #NUM! | 0 | Baseline |
| Male | 5 to 14 | 0 | #NUM! | 0 | Baseline |
| Female | 15 to 21 | 0 | #NUM! | 0 | Baseline |
| Male | 35+ | 0 | #NUM! | 0 | Baseline |
| Female | 35+ | 0 | #NUM! | 0 | Baseline |
| Male | 35+ | 0 | #NUM! | 0 | Baseline |
| Male | 22 to 35 | 0 | #NUM! | 0 | Baseline |
| Male | 22 to 35 | 0 | #NUM! | 0 | Baseline |
| Male | 22 to 35 | 0 | #NUM! | 0 | Baseline |
| Male | 22 to 35 | 0 | #NUM! | 0 | Baseline |
| Male | 35+ | 0 | #NUM! | 0 | Baseline |
| Male | 22 to 35 | 0 | #NUM! | 0 | Baseline |
| Male | 35+ | 0 | #NUM! | 0 | Baseline |
| Female | 22 to 35 | 0 | #NUM! | 0 | Baseline |
| Male | 22 to 35 | 0 | #NUM! | 0 | Baseline |
| Female | 35+ | 0 | #NUM! | 0 | Baseline |

| Female | 22 to 35 | 0 | #NUM! | 0 | Baseline |
| --- | --- | --- | --- | --- | --- |
| Female | 22 to 35 | 0 | #NUM! | 0 | Baseline |
| Male | 35+ | 0 | #NUM! | 0 | Baseline |
| Female | 35+ | 0 | #NUM! | 0 | Baseline |
| Female | 22 to 35 | 0 | #NUM! | 0 | Baseline |
| Female | 35+ | 0 | #NUM! | 0 | Baseline |
| Female | 15 to 21 | 0 | #NUM! | 0 | Baseline |
| Male | 5 to 14 | 0 | #NUM! | 0 | Baseline |
| Female | 15 to 21 | 0 | #NUM! | 0 | Baseline |
| Male | 15 to 21 | 0 | #NUM! | 0 | Baseline |
| Female | 5 to 14 | 0 | #NUM! | 0 | Baseline |
| Male | 5 to 14 | 0 | #NUM! | 0 | Baseline |
| Male | 5 to 14 | 0 | #NUM! | 0 | Baseline |
| Female | 15 to 21 | 0 | #NUM! | 0 | Baseline |
| Female | 5 to 14 | 0 | #NUM! | 0 | Baseline |
| Male | 15 to 21 | 0 | #NUM! | 0 | Baseline |
| Female | 15 to 21 | 0 | #NUM! | 0 | Baseline |
| Male | 5 to 14 | 0 | #NUM! | 0 | Baseline |
| Female | 5 to 14 | 0 | #NUM! | 0 | Baseline |
| Female | 5 to 14 | 0 | #NUM! | 0 | Baseline |
| Female | 5 to 14 | 0 | #NUM! | 0 | Baseline |
| Female | 15 to 21 | 0 | #NUM! | 0 | Baseline |
| Male | 15 to 21 | 0 | #NUM! | 0 | Baseline |
| Female | 35+ | 0 | #NUM! | 0 | Baseline |
| Male | 22 to 35 | 0 | #NUM! | 0 | Baseline |
| Male | 35+ | 0 | #NUM! | 0 | Baseline |
| Male | 22 to 35 | 0 | #NUM! | 0 | Baseline |
| Male | 22 to 35 | 0 | #NUM! | 0 | Baseline |
| Male | 15 to 21 | 0 | #NUM! | 0 | Baseline |
| Female | 22 to 35 | 0 | #NUM! | 0 | Baseline |
| Male | 15 to 21 | 0 | #NUM! | 0 | Baseline |
| Female | 22 to 35 | 0 | #NUM! | 0 | Baseline |
| Male | 35+ | 0 | #NUM! | 0 | Baseline |
| Female | 35+ | 0 | #NUM! | 0 | Baseline |
| Male | 22 to 35 | 0 | #NUM! | 0 | Baseline |
| Female | 22 to 35 | 0 | #NUM! | 0 | Baseline |
| Female | 22 to 35 | 0 | #NUM! | 0 | Baseline |
| Female | 22 to 35 | 0 | #NUM! | 0 | Baseline |
| Male | 5 to 14 | 0 | #NUM! | 0 | Baseline |
| Male | 35+ | 0 | #NUM! | 0 | Baseline |
| Female | 35+ | 0 | #NUM! | 0 | Baseline |
| Female | 22 to 35 | 0 | #NUM! | 0 | Baseline |
| Male | 22 to 35 | 0 | #NUM! | 0 | Baseline |
| Male | 15 to 21 | 0 | #NUM! | 0 | Baseline |
| Female | 22 to 35 | 0 | #NUM! | 0 | Baseline |
| Female | 22 to 35 | 0 | #NUM! | 0 | Baseline |
| Female | 22 to 35 | 0 | #NUM! | 0 | Baseline |
| Male | 22 to 35 | 0 | #NUM! | 0 | Baseline |
| Female | 35+ | 0 | #NUM! | 0 | Baseline |
| Female | 22 to 35 | 0 | #NUM! | 0 | Baseline |

| Male | 15 to 21 | 0 | #NUM! | 0 | Baseline |
| --- | --- | --- | --- | --- | --- |
| Male | 5 to 14 | 0 | #NUM! | 0 | Baseline |
| Female | 22 to 35 | 0 | #NUM! | 0 | Baseline |
| Male | 22 to 35 | 0 | #NUM! | 0 | Baseline |
| Male | 5 to 14 | 0 | #NUM! | 0 | Baseline |
| Male | 22 to 35 | 0 | #NUM! | 0 | Baseline |
| Female | 15 to 21 | 0 | #NUM! | 0 | Baseline |
| Female | 5 to 14 | 0 | #NUM! | 0 | Baseline |
| Female | 5 to 14 | 0 | #NUM! | 0 | Baseline |
| Male | 5 to 14 | 0 | #NUM! | 0 | Baseline |
| Male | 5 to 14 | 0 | #NUM! | 0 | Baseline |
| Male | 5 to 14 | 0 | #NUM! | 0 | Baseline |
| Male | 1 to 4 | 0 | #NUM! | 0 | Baseline |
| Female | 5 to 14 | 0 | #NUM! | 0 | Baseline |
| Female | 5 to 14 | 0 | #NUM! | 0 | Baseline |
| Male | 5 to 14 | 0 | #NUM! | 0 | Baseline |
| Female | 15 to 21 | 0 | #NUM! | 0 | Baseline |
| Female | 15 to 21 | 0 | #NUM! | 0 | Baseline |
| Female | 5 to 14 | 0 | #NUM! | 0 | Baseline |
| Male | 5 to 14 | 0 | #NUM! | 0 | Baseline |
| Female | 5 to 14 | 0 | #NUM! | 0 | Baseline |
| Male | 15 to 21 | 0 | #NUM! | 0 | Baseline |
| Female | 15 to 21 | 0 | #NUM! | 0 | Baseline |
| Male | 15 to 21 | 0 | #NUM! | 0 | Baseline |
| Female | 5 to 14 | 0 | #NUM! | 0 | Baseline |
| Male | 15 to 21 | 0 | #NUM! | 0 | Baseline |
| Male | 5 to 14 | 0 | #NUM! | 0 | Baseline |
| Female | 5 to 14 | 0 | #NUM! | 0 | Baseline |
| Male | 5 to 14 | 0 | #NUM! | 0 | Baseline |
| Female | 5 to 14 | 0 | #NUM! | 0 | Baseline |
| Male | 15 to 21 | 0 | #NUM! | 0 | Baseline |
| Female | 15 to 21 | 0 | #NUM! | 0 | Baseline |
| Female | 5 to 14 | 0 | #NUM! | 0 | Baseline |
| Male | 5 to 14 | 0 | #NUM! | 0 | Baseline |
| Male | 5 to 14 | 0 | #NUM! | 0 | Baseline |
| Male | 15 to 21 | 0 | #NUM! | 0 | Baseline |
| Male | 5 to 14 | 0 | #NUM! | 0 | Baseline |
| Male | 35+ | 0 | #NUM! | 0 | Baseline |
| Male | 22 to 35 | 0 | #NUM! | 0 | Baseline |
| Female | 5 to 14 | 0 | #NUM! | 0 | Baseline |
| Female | 5 to 14 | 0 | #NUM! | 0 | Baseline |
| Female | 35+ | 0 | #NUM! | 0 | Baseline |
| Female | 15 to 21 | 0 | #NUM! | 0 | Baseline |
| Female | 22 to 35 | 0 | #NUM! | 0 | Baseline |
| Female | 22 to 35 | 0 | #NUM! | 0 | Baseline |
| Male | 22 to 35 | 0 | #NUM! | 0 | Baseline |
| Male | 1 to 4 | 0 | #NUM! | 0 | Baseline |
| Male | 22 to 35 | 0 | #NUM! | 0 | Baseline |
| Male | 22 to 35 | 0 | #NUM! | 0 | Baseline |
| Female | 22 to 35 | 0 | #NUM! | 0 | Baseline |

| Female | 35+ | 0 | #NUM! | 0 | Baseline |
| --- | --- | --- | --- | --- | --- |
| Male | 35+ | 0 | #NUM! | 0 | Baseline |
| Female | 5 to 14 | 0 | #NUM! | 0 | Baseline |
| Female | 22 to 35 | 0 | #NUM! | 0 | Baseline |
| Male | 5 to 14 | 0 | #NUM! | 0 | Baseline |
| Female | 5 to 14 | 0 | #NUM! | 0 | Baseline |
| Female | 5 to 14 | 0 | #NUM! | 0 | Baseline |
| Male | 22 to 35 | 0 | #NUM! | 0 | Baseline |
| Male | 22 to 35 | 0 | #NUM! | 0 | Baseline |
| Female | 15 to 21 | 0 | #NUM! | 0 | Baseline |
| Male | 15 to 21 | 0 | #NUM! | 0 | Baseline |
| Male | 1 to 4 | 0 | #NUM! | 0 | Baseline |
| Male | 5 to 14 | 0 | #NUM! | 0 | Baseline |
| Female | 22 to 35 | 0 | #NUM! | 0 | Baseline |
| Male | 1 to 4 | 0 | #NUM! | 0 | Baseline |
| Female | 22 to 35 | 0 | #NUM! | 0 | Baseline |
| Male | 22 to 35 | 0 | #NUM! | 0 | Baseline |
| Female | 22 to 35 | 0 | #NUM! | 0 | Baseline |
| Male | 1 to 4 | 0 | #NUM! | 0 | Baseline |
| Male | 1 to 4 | 0 | #NUM! | 0 | Baseline |
| Female | 15 to 21 | 0 | #NUM! | 0 | Baseline |
| Male | 35+ | 0 | #NUM! | 0 | Baseline |
| Female | 5 to 14 | 0 | #NUM! | 0 | Baseline |
| Male | 35+ | 0 | #NUM! | 0 | Baseline |
| Female | 22 to 35 | 0 | #NUM! | 0 | Baseline |
| Male | 35+ | 0 | #NUM! | 0 | Baseline |
| Male | 1 to 4 | 0 | #NUM! | 0 | Baseline |
| Female | 22 to 35 | 0 | #NUM! | 0 | Baseline |
| Female | 15 to 21 | 0 | #NUM! | 0 | Baseline |
| Male | 15 to 21 | 0 | #NUM! | 0 | Baseline |
| Female | 15 to 21 | 0 | #NUM! | 0 | Baseline |
| Male | 5 to 14 | 0 | #NUM! | 0 | Baseline |
| Female | 22 to 35 | 0 | #NUM! | 0 | Baseline |
| Female | 35+ | 0 | #NUM! | 0 | Baseline |
| Female | 22 to 35 | 0 | #NUM! | 0 | Baseline |
| Female | 22 to 35 | 0 | #NUM! | 0 | Baseline |
| Female | 35+ | 0 | #NUM! | 0 | Baseline |
| Female | 15 to 21 | 0 | #NUM! | 0 | Baseline |
| Female | 22 to 35 | 0 | #NUM! | 0 | Baseline |
| Female | 15 to 21 | 0 | #NUM! | 0 | Baseline |
| Male | 15 to 21 | 0 | #NUM! | 0 | Baseline |
| Female | 15 to 21 | 0 | #NUM! | 0 | Baseline |
| Female | 15 to 21 | 0 | #NUM! | 0 | Baseline |
| Female | 15 to 21 | 0 | #NUM! | 0 | Baseline |
| Male | 15 to 21 | 0 | #NUM! | 0 | Baseline |
| Female | 15 to 21 | 0 | #NUM! | 0 | Baseline |
| Male | 15 to 21 | 0 | #NUM! | 0 | Baseline |
| Female | 15 to 21 | 0 | #NUM! | 0 | Baseline |
| Male | 15 to 21 | 0 | #NUM! | 0 | Baseline |
| Female | 5 to 14 | 0 | #NUM! | 0 | Baseline |

| Male | 5 to 14 | 0 | #NUM! | 0 | Baseline |
| --- | --- | --- | --- | --- | --- |
| Male | 5 to 14 | 0 | #NUM! | 0 | Baseline |
| Male | 5 to 14 | 0 | #NUM! | 0 | Baseline |
| Male | 5 to 14 | 0 | #NUM! | 0 | Baseline |
| Male | 5 to 14 | 0 | #NUM! | 0 | Baseline |
| Male | 5 to 14 | 0 | #NUM! | 0 | Baseline |
| Male | 5 to 14 | 0 | #NUM! | 0 | Baseline |
| Female | 5 to 14 | 0 | #NUM! | 0 | Baseline |
| Female | 5 to 14 | 0 | #NUM! | 0 | Baseline |
| Male | 5 to 14 | 0 | #NUM! | 0 | Baseline |
| Female | 5 to 14 | 0 | #NUM! | 0 | Baseline |
| Male | 5 to 14 | 0 | #NUM! | 0 | Baseline |
| Male | 5 to 14 | 0 | #NUM! | 0 | Baseline |
| Female | 5 to 14 | 0 | #NUM! | 0 | Baseline |
| Male | 15 to 21 | 0 | #NUM! | 0 | Baseline |
| Female | 15 to 21 | 0 | #NUM! | 0 | Baseline |
| Male | 15 to 21 | 0 | #NUM! | 0 | Baseline |
| Male | 15 to 21 | 0 | #NUM! | 0 | Baseline |
| Male | 15 to 21 | 0 | #NUM! | 0 | Baseline |
| Female | 15 to 21 | 0 | #NUM! | 0 | Baseline |
| Female | 15 to 21 | 0 | #NUM! | 0 | Baseline |
| Male | 5 to 14 | 0 | #NUM! | 0 | Baseline |
| Female | 5 to 14 | 0 | #NUM! | 0 | Baseline |
| Male | 5 to 14 | 0 | #NUM! | 0 | Baseline |
| Female | 15 to 21 | 0 | #NUM! | 0 | Baseline |
| Male | 5 to 14 | 0 | #NUM! | 0 | Baseline |
| Female | 5 to 14 | 0 | #NUM! | 0 | Baseline |
| Male | 1 to 4 | 0 | #NUM! | 0 | Baseline |
| Male | 5 to 14 | 0 | #NUM! | 0 | Baseline |
| Male | 5 to 14 | 0 | #NUM! | 0 | Baseline |
| Female | 5 to 14 | 0 | #NUM! | 0 | Baseline |
| Female | 5 to 14 | 0 | #NUM! | 0 | Baseline |
| Female | 5 to 14 | 0 | #NUM! | 0 | Baseline |
| Female | 5 to 14 | 0 | #NUM! | 0 | Baseline |
| Male | 1 to 4 |  | #NUM! | 0 | Baseline |
| Female | 15 to 21 | 0 | #NUM! | 0 | Baseline |
| Male | 5 to 14 | 0 | #NUM! | 0 | Baseline |
| Male | 5 to 14 | 0 | #NUM! | 0 | Baseline |
| Female | 5 to 14 | 0 | #NUM! | 0 | Baseline |
| Male | 15 to 21 | 0 | #NUM! | 0 | Baseline |
| Female | 5 to 14 | 0 | #NUM! | 0 | Baseline |
| Female | 15 to 21 | 0 | #NUM! | 0 | Baseline |
| Male | 1 to 4 | 0 | #NUM! | 0 | Baseline |
| Female | 35+ | 0 | #NUM! | 0 | Baseline |
| Male | 15 to 21 | 0 | #NUM! | 0 | Baseline |
| Male | 15 to 21 | 0 | #NUM! | 0 | Baseline |
| Male | 15 to 21 | 0 | #NUM! | 0 | Baseline |
| Female | 35+ | 0 | #NUM! | 0 | Baseline |
| Female | 35+ | 0 | #NUM! | 0 | Baseline |
| Female | 35+ | 0 | #NUM! | 0 | Baseline |

| Female | 35+ | 0 | #NUM! | 0 | Baseline |
| --- | --- | --- | --- | --- | --- |
| Male | 35+ | 0 | #NUM! | 0 | Baseline |
| Male | 35+ | 0 | #NUM! | 0 | Baseline |
| Male | 1 to 4 | 0 | #NUM! | 0 | Baseline |
| Female | 5 to 14 | 0 | #NUM! | 0 | Baseline |
| Female | 5 to 14 | 0 | #NUM! | 0 | Baseline |
| Male | 5 to 14 | 0 | #NUM! | 0 | Baseline |
| Female | 5 to 14 | 0 | #NUM! | 0 | Baseline |
| Female | 5 to 14 | 0 | #NUM! | 0 | Baseline |
| Male | 5 to 14 | 0 | #NUM! | 0 | Baseline |
| Male | 5 to 14 | 0 | #NUM! | 0 | Baseline |
| Female | 5 to 14 | 0 | #NUM! | 0 | Baseline |
| Male | 5 to 14 | 0 | #NUM! | 0 | Baseline |
| Male | 5 to 14 | 0 | #NUM! | 0 | Baseline |
| Female | 5 to 14 | 0 | #NUM! | 0 | Baseline |
| Male | 15 to 21 | 0 | #NUM! | 0 | Baseline |
| Female | 15 to 21 | 0 | #NUM! | 0 | Baseline |
| Male | 15 to 21 | 0 | #NUM! | 0 | Baseline |
| Male | 15 to 21 | 0 | #NUM! | 0 | Baseline |
| Male | 15 to 21 | 0 | #NUM! | 0 | Baseline |
| Female | 15 to 21 | 0 | #NUM! | 0 | Baseline |
| Female | 15 to 21 | 0 | #NUM! | 0 | Baseline |
| Male | 15 to 21 | 0 | #NUM! | 0 | Baseline |
| Male | 15 to 21 | 0 | #NUM! | 0 | Baseline |
| Male | 15 to 21 | 0 | #NUM! | 0 | Baseline |
| Male | 15 to 21 | 0 | #NUM! | 0 | Baseline |
| Female | 15 to 21 | 0 | #NUM! | 0 | Baseline |
| Female | 5 to 14 | 0 | #NUM! | 0 | Baseline |
| Male | 5 to 14 | 0 | #NUM! | 0 | Baseline |
| Female | 5 to 14 | 0 | #NUM! | 0 | Baseline |
| Male | 22 to 35 | 0 | #NUM! | 0 | Baseline |
| Female | 22 to 35 | 0 | #NUM! | 0 | Baseline |
| Female | 5 to 14 | 0 | #NUM! | 0 | Baseline |
| Female | 5 to 14 | 0 | #NUM! | 0 | Baseline |
| Female | 5 to 14 | 0 | #NUM! | 0 | Baseline |
| Female | 5 to 14 | 0 | #NUM! | 0 | Baseline |
| Male | 15 to 21 | 0 | #NUM! | 0 | Baseline |
| Female | 1 to 4 | 0 | #NUM! | 0 | Baseline |
| Male | 15 to 21 | 0 | #NUM! | 0 | Baseline |
| Male | 5 to 14 | 0 | #NUM! | 0 | Baseline |
| Male | 15 to 21 | 0 | #NUM! | 0 | Baseline |
| Male | 15 to 21 | 0 | #NUM! | 0 | Baseline |
| Female | 15 to 21 | 0 | #NUM! | 0 | Baseline |
| Female | 15 to 21 | 0 | #NUM! | 0 | Baseline |
| Female | 15 to 21 | 0 | #NUM! | 0 | Baseline |
| Male | 15 to 21 | 0 | #NUM! | 0 | Baseline |
| Female | 15 to 21 | 0 | #NUM! | 0 | Baseline |
| Male | 15 to 21 | 0 | #NUM! | 0 | Baseline |
| Female | 15 to 21 | 0 | #NUM! | 0 | Baseline |
| Female | 15 to 21 | 0 | #NUM! | 0 | Baseline |

| Female | 15 to 21 | 0 | #NUM! | 0 | Baseline |
| --- | --- | --- | --- | --- | --- |
| Female | 5 to 14 | 0 | #NUM! | 0 | Baseline |
| Female | 5 to 14 | 0 | #NUM! | 0 | Baseline |
| Female | 5 to 14 | 0 | #NUM! | 0 | Baseline |
| Female | 5 to 14 | 0 | #NUM! | 0 | Baseline |
| Female | 5 to 14 | 0 | #NUM! | 0 | Baseline |
| Male | 5 to 14 | 0 | #NUM! | 0 | Baseline |
| Male | 5 to 14 | 0 | #NUM! | 0 | Baseline |
| Male | 5 to 14 | 0 | #NUM! | 0 | Baseline |
| Male | 5 to 14 | 0 | #NUM! | 0 | Baseline |
| Male | 5 to 14 | 0 | #NUM! | 0 | Baseline |
| Male | 5 to 14 | 0 | #NUM! | 0 | Baseline |
| Female | 35+ | 0 | #NUM! | 0 | Baseline |
| Male | 35+ | 0 | #NUM! | 0 | Baseline |
| Male | 35+ | 0 | #NUM! | 0 | Baseline |
| Male | 22 to 35 | 0 | #NUM! | 0 | Baseline |
| Male | 35+ | 0 | #NUM! | 0 | Baseline |
| Female | 22 to 35 | 0 | #NUM! | 0 | Baseline |
| Female | 22 to 35 | 0 | #NUM! | 0 | Baseline |
| Male | 22 to 35 | 0 | #NUM! | 0 | Baseline |
| Male | 22 to 35 | 0 | #NUM! | 0 | Baseline |
| Male | 22 to 35 | 0 | #NUM! | 0 | Baseline |
| Male | 22 to 35 | 0 | #NUM! | 0 | Baseline |
| Female | 35+ | 0 | #NUM! | 0 | Baseline |
| Female | 35+ | 0 | #NUM! | 0 | Baseline |
| Male | 35+ | 0 | #NUM! | 0 | Baseline |
| Female | 22 to 35 | 0 | #NUM! | 0 | Baseline |
| Male | 35+ | 0 | #NUM! | 0 | Baseline |
| Female | 35+ | 0 | #NUM! | 0 | Baseline |
| Female | 35+ | 0 | #NUM! | 0 | Baseline |
| Female | 35+ | 0 | #NUM! | 0 | Baseline |
| Male | 35+ | 0 | #NUM! | 0 | Baseline |
| Female | 35+ | 0 | #NUM! | 0 | Baseline |
| Male | 5 to 14 | 0 | #NUM! | 0 | Baseline |
| Female | 5 to 14 | 0 | #NUM! | 0 | Baseline |
| Male | 1 to 4 | 0 | #NUM! | 0 | Baseline |
| Male | 35+ | 0 | #NUM! | 0 | Baseline |
| Male | 5 to 14 | 0 | #NUM! | 0 | Baseline |
| Female | 5 to 14 | 0 | #NUM! | 0 | Baseline |
| Male | 35+ | 0 | #NUM! | 0 | Baseline |
| Male | 35+ | 0 | #NUM! | 0 | Baseline |
| Male | 22 to 35 | 0 | #NUM! | 0 | Baseline |
| Female | 22 to 35 | 0 | #NUM! | 0 | Baseline |
| Male | 22 to 35 | 0 | #NUM! | 0 | Baseline |
| Male | 22 to 35 | 0 | #NUM! | 0 | Baseline |
| Female | 22 to 35 | 0 | #NUM! | 0 | Baseline |
| Female | 35+ | 0 | #NUM! | 0 | Baseline |
| Female | 22 to 35 | 0 | #NUM! | 0 | Baseline |
| Male | 35+ | 0 | #NUM! | 0 | Baseline |
| Female | 22 to 35 | 0 | #NUM! | 0 | Baseline |

| Male | 5 to 14 | 0 | #NUM! | 0 | Baseline |
| --- | --- | --- | --- | --- | --- |
| Female | 5 to 14 | 0 | #NUM! | 0 | Baseline |
| Male | 5 to 14 | 0 | #NUM! | 0 | Baseline |
| Male | 5 to 14 | 0 | #NUM! | 0 | Baseline |
| Male | 5 to 14 | 0 | #NUM! | 0 | Baseline |
| Male | 1 to 4 | 0 | #NUM! | 0 | Baseline |
| Male | 5 to 14 | 0 | #NUM! | 0 | Baseline |
| Male | 1 to 4 | 0 | #NUM! | 0 | Baseline |
| Male | 1 to 4 | 0 | #NUM! | 0 | Baseline |
| Male | 1 to 4 | 0 | #NUM! | 0 | Baseline |
| Male | 1 to 4 | 0 | #NUM! | 0 | Baseline |
| Male | 1 to 4 | 0 | #NUM! | 0 | Baseline |
| Male | 1 to 4 | 0 | #NUM! | 0 | Baseline |
| Male | 1 to 4 | 0 | #NUM! | 0 | Baseline |
| Male | 1 to 4 | 0 | #NUM! | 0 | Baseline |
| Male | 1 to 4 | 0 | #NUM! | 0 | Baseline |
| Male | 1 to 4 | 0 | #NUM! | 0 | Baseline |
| Male | 1 to 4 | 0 | #NUM! | 0 | Baseline |
| Male | 1 to 4 | 0 | #NUM! | 0 | Baseline |
| Male | 22 to 35 | 0 | #NUM! | 0 | Baseline |
| Male | 22 to 35 | 0 | #NUM! | 0 | Baseline |
| Female | 22 to 35 | 0 | #NUM! | 0 | Baseline |
| Male | 22 to 35 | 0 | #NUM! | 0 | Baseline |
| Male | 22 to 35 | 0 | #NUM! | 0 | Baseline |
| Male | 22 to 35 | 0 | #NUM! | 0 | Baseline |
| Male | 22 to 35 | 0 | #NUM! | 0 | Baseline |
| Female | 22 to 35 | 0 | #NUM! | 0 | Baseline |
| Female | 22 to 35 | 0 | #NUM! | 0 | Baseline |
| Male | 22 to 35 | 0 | #NUM! | 0 | Baseline |
| Male | 1 to 4 | 0 | #NUM! | 0 | Baseline |
| Male | 35+ | 0 | #NUM! | 0 | Baseline |
| Male | 22 to 35 | 0 | #NUM! | 0 | Baseline |
| Female | 15 to 21 | 0 | #NUM! | 0 | Baseline |
| Female | 15 to 21 | 0 | #NUM! | 0 | Baseline |
| Male | 22 to 35 | 0 | #NUM! | 0 | Baseline |
| Male | 15 to 21 | 0 | #NUM! | 0 | Baseline |
| Female | 15 to 21 | 0 | #NUM! | 0 | Baseline |
| Male | 15 to 21 | 0 | #NUM! | 0 | Baseline |
| Male | 15 to 21 | 0 | #NUM! | 0 | Baseline |
| Male | 15 to 21 | 0 | #NUM! | 0 | Baseline |
| Male | 22 to 35 | 0 | #NUM! | 0 | Baseline |
| Male | 5 to 14 | 0 | #NUM! | 0 | Baseline |
| Male | 5 to 14 | 0 | #NUM! | 0 | Baseline |
| Female | 5 to 14 | 0 | #NUM! | 0 | Baseline |
| Male | 1 to 4 | 0 | #NUM! | 0 | Baseline |
| Female | 5 to 14 | 0 | #NUM! | 0 | Baseline |
| Male | 1 to 4 | 0 | #NUM! | 0 | Baseline |
| Male | 1 to 4 | 0 | #NUM! | 0 | Baseline |
| Male | 5 to 14 | 0 | #NUM! | 0 | Baseline |
| Female | 35+ | 0 | #NUM! | 0 | Baseline |

| Male | 35+ | 0 | #NUM! | 0 | Baseline |
| --- | --- | --- | --- | --- | --- |
| Male | 22 to 35 | 0 | #NUM! | 0 | Baseline |
| Female | 35+ | 0 | #NUM! | 0 | Baseline |
| Male | 22 to 35 | 0 | #NUM! | 0 | Baseline |
[truncated: 210,370 more chars]
