# Supplementary material for: Comparing the accuracy of two diagnostic methods for detection of light Schistosoma haematobium infection in an elimination setting in Wolaita Zone, South Western Ethiopia
Source: PLoS One. 2022 Apr 29;17(4):e0267378. doi: 10.1371/journal.pone.0267378 (PMC9053789; doi:10.1371/journal.pone.0267378)
Supplement: Attachment — Submitted filename: Peer Review PONE-D-21-07166.docx [file pone.0267378.s003.docx]

**PLoS One Peer Review # PONE-D-21-07166**

1. **Summary of the research and your overall impression**

*This study is a relevant comparison of the diagnostic accuracy of detecting light Schistosoma haematobium infections which would go a long way towards addressing shortcomings in the monitoring and evaluation of intervention programs. The study compares the urinalysis dipstick method and the urine filtration method (reference gold-standard) statistically using a significantly large study population over a period of two years. The study was well designed to compare the two methods which revealed significantly different outcomes in terms of sensitivity and specificity. The study addresses a very relevant question of the accuracy of dipstick method and the possibility of its use as a proxy or adjunct diagnostic tool for the more involving and less sensitive gold-standard filtration method.*

*The study included a large cohort of study subjects at baseline with fewer subjects at two yearly follow-up points. This presented a robust population for analysis and conclusive findings. However, I struggled to understand how kappa statistics indicated an agreement between the disparate methods and outcomes as concluded by the authors.*

*It was not clear to this reviewer how exactly the urine filtration method was carried out in this study. The authors need to clearly state how they got two slide counts of S. haematobium eggs. Specifically, were the egg counts made from a single one-time point urine sample or from two samples taken on consecutive days? Egg shedding varies from day to day and can affect sensitivity of test, which is the reason why the WHO procedures usually recommend 3 separate samples taken on 3 consecutive days and taking the average egg count per 10 mL of urine.*

*I recommend publication with revisions to address method procedures and better clarification of statistics and their interpretation and limitations. Otherwise I defer to better statisticians.*

2. **Evidence and examples**

**Major Issues**

*It is of great concern that most microhematuria results of the dipstick test could not be associated with S. haematobium infection based on the reference filtration method implying little correlation between the two methods. If menses were the likely major source of this anomaly, then female subjects of reproductive age would have higher levels of non-specific microhematuria which is discordant with the presence of S. haematobium eggs. This possible outcome explanation could be verified or excluded by running the statistics by gender.*

**Minor Issues**

*There is a need to copy-edit the manuscript for language, grammar and consistency in the use of past tense particularly in the field and diagnostic procedure descriptions.*

*There are no results for the POC-CCA test which was described in the methods. This should not be referenced if the results were not recorded and had no relevance to the current study. A significant portion of the discussion was spent on this test with no evident results.*

3. **Other points** (**optional**)

*Will be available to review the revised manuscript.*
