# Supplementary material for: Comparing the accuracy of two diagnostic methods for detection of light Schistosoma haematobium infection in an elimination setting in Wolaita Zone, South Western Ethiopia
Source: PLoS One. 2022 Apr 29;17(4):e0267378. doi: 10.1371/journal.pone.0267378 (PMC9053789; doi:10.1371/journal.pone.0267378)
Supplement: Attachment — Submitted filename: letter and Respond to reviwers a point by point response.docx [file pone.0267378.s005.docx]

Editor-in-Chief

PLoS ONE Journal

July 5, 2021

Dear Editor,

Re manuscript: PONE-D-21-07166” **Comparing the diagnostic accuracy for detection of light *Schistosoma haematobium* infection in an elimination setting in Wolaita Zone, South Western Ethiopia”.**

Many thanks for the opportunity you gave us to revise and re-submit our manuscript. The reviewers gave positive and constructive feedback that will further enrich out manuscripts. We have addressed their comments in the attached manuscript with track changes and provided a detailed point-by-point response below. The revised manuscript addressed all the concern and suggestion of the reviewer’s which improved the manuscript. Please look at the detailed point-by-point response to reviewer report and hope the manuscript is now acceptable for potential publication.

With kind regards,

Hussein Mohammed, Researcher (BSc, MSc)

**Reviewer reports #1.Please include a copy of Table 3 which you refer to in your text.**

**Response:** *Thank you and due to clerical errors, we have corrected each table included table 3.*

**Reviewer report #2.** Please upload a Response to Reviewers letter which should include a point by point response to each of the points made by the Editor and / or Reviewers. (This should be uploaded as a 'Response to Reviewers' file type.) Please follow this link for more information: [http://blogs.PLOS.org/everyone/2011/05/10/how-to-submit-your-revised-manuscript/](http://blogs.plos.org/everyone/2011/05/10/how-to-submit-your-revised-manuscript/)

**Response**: *Thank you and we have now corrected per the reviewer suggestion.*

**Reviewer report#3. Thank you for updating your Data Availability Statement to: "The datasets supporting the conclusions of this article are available upon request. EPHI has a policy to share data to third party provided that MOU and data sharing policy has been signed."**

**Response.** *The data supporting the conclusions of this article are included within the article and it’s also found in additional file.*
