# Supplementary material for: Comparing the accuracy of two diagnostic methods for detection of light Schistosoma haematobium infection in an elimination setting in Wolaita Zone, South Western Ethiopia
Source: PLoS One. 2022 Apr 29;17(4):e0267378. doi: 10.1371/journal.pone.0267378 (PMC9053789; doi:10.1371/journal.pone.0267378)
Supplement: Attachment — Submitted filename: Letter and Response to editor-23-03-2022.docx [file pone.0267378.s007.docx]

To Audyssa Banlaygas Auditor	March 23, 2022
Editor
PLOS ONE

Subject: Submission of revised version of a manuscript that addresses the points raised during the review process
Manuscript Number: PONE-D-21-07166R1

Dear Audyssa Banlaygas

Many thanks for the opportunity you gave us to revise and re-submit our manuscript, entitled “***Comparing the accuracy of two diagnostic methods for detection of light Schistosoma haematobium infection in an elimination setting in Wolaita Zone, South Western Ethiopia”.*** We have considered all of the comments very carefully and have found them to be important to further enrich the manuscript. Please look at the detailed point-by-point response to the comments made by the academic editor.

With kind regards,

Hussein Mohammed, Researcher (BSc, MSc)

A. **Editor**

**Questions raised:**

1. Please amend the title either on the online submission form or in your manuscript so that they are identical.

**Author’s response #1:** *Thank you and we have now corrected the title in the online submission form as per the editor suggestion.*

### 2. Please include a separate legend for each figure in your manuscript.

### Author’s response #2: *Thank you and we have now corrected as per the editor comment. (Detection of urine for microhematuria subsection,detection of urine of microhaematuria subsetion on page6,line 7; result section (page 9 and line1;paragraph 3,page9,ine 4).*

3. Please include your tables as part of your main manuscript and remove the individual files. Please note that supplementary tables (should remain/ be uploaded) as separate "Supporting Information" files.

**Author’s response #3:** *Thank you and we have now included as per the editor* comment (*Page 8, line 8*)
