# Supplementary material for: Comparing the accuracy of two diagnostic methods for detection of light Schistosoma haematobium infection in an elimination setting in Wolaita Zone, South Western Ethiopia
Source: PLoS One. 2022 Apr 29;17(4):e0267378. doi: 10.1371/journal.pone.0267378 (PMC9053789; doi:10.1371/journal.pone.0267378)
Supplement: Attachment — Submitted filename: Reviewer comment to Author 04222021.docx [file pone.0267378.s004.docx]

**Review of manuscript:** Comparing the diagnostic accuracy for detection of light *Schistosoma haematobium* infection in an elimination setting in Wolaita Zone, South Western Ethiopia

**Reviewer’s Comments to Author**

**Comments to the Author**

The manuscript proposed by Mohammed et al. is titled: Comparing the diagnostic accuracy for detection of light *Schistosoma haematobium* infection in an elimination setting in Wolaita Zone, South Western Ethiopia

The study described in this manuscript was conducted in the Wolaita Zone, South Western Ethiopia and it provides information on the comparative analyses of two schistosomiasis diagnostic techniques – Urinalysis dipstick and urine filtration. The authors attempted to shed a new light on the use of these diagnostic techniques in order to fill gaps in our knowledge of schistosomiasis control. The study was conducted between 2018 and 2020 and a total of 16,780 children and adults participated in the study. A total of 12,102 samples were examined in the baseline study using dipstick and urine filtration; among them, 285 (2.4%) samples were positive for microhematuria and 21 (0.2%) positive for *S. haematobium* eggs by urine filtration. 501 urine samples were examined in 2019 and 10 (2.0%) were positive for microhematuria, and all of them were negative for *S. haematobium* infection. A total of 3875 individuals were examined in 2020 and 162 (4.2%) were positive for microhematuria and 2 (0.05%) were positive for *S. haematobium* eggs. The prevalence of microhematuria infection based on reagent dipstick was comparable between males (50.9%) and females (49.1%), and across the different age groups Pre-SAC (17.1%), SAC (25.0%), 15 to 20 (20.4%), 21 to 35 (20.2%) and 36+(17.3%) years. The prevalence of microhematuria was highest in SAC (4-15 years age) compared to other age groups. The sensitivity and specificity of reagent strip was 100% (95% CI:85.18%–100.00%) and 97.4% (95% CI: 97.10%-97.60%), respectively. The PPV was low 5.03% and NPV was high 100%.

Although the study provided some information on these diagnostic techniques, there are inconsistencies in the results provided in the study. I will recommend the manuscript undergo a major revision based on the comments addressed below.

**Title**

Comparing the diagnostic accuracy for detection of light *Schistosoma haematobium* infection in an elimination setting in Wolaita Zone, South Western Ethiopia

Suggested Title

Comparing the accuracy of two diagnostic methods for detection of light *Schistosoma haematobium* infection in an elimination setting in Wolaita Zone, South Western Ethiopia

**Abstract**

“A total of 48,088 participants were enrolled in the current study. Among them, 285 (2.4%) samples tested positive for microhematuria and 21 (0.2%) positive for *S. haematobium* eggs in the baseline survey.” This information is not correct based on the calculations. Information in the results section states “total of 16,780 children and adults were enrolled to participate in the baseline and cross-sectional surveys conducted between 2018 and 2020.” The abstract also states that “A total of 4376 samples were examined using urine dipsticks and urine filtration 172 (6.2%) and 2 (0.05%) were positive for microhematuria and S*. haematobium* eggs. The percentages are not correct and need to be revised.

**Introduction**

There are a few typographical errors in the introduction section that need to be corrected. I recommend that this section be revised.

**Materials and Methods
Sample size calculations**
“The sample size was estimated using at least 70% probability of woredas with true prevalence of schistosomiasis of 15% being classified as 10% or over, under the assumption that the lower

focality of STH was led to sufficient sample sizes for schistosomiasis between 26% and 75% of

kebeles were surveyed within each woreda [21], with a higher proportion of kebeles being

surveyed in small woreda.”

The sentence above needs revision for better comprehension.

The abbreviations pre-SAC and SAC should be defined if it was not defined in another part of the manuscript.

**Laboratory diagnosis**

**Detection of urine for microhematuria**
The subtitle above should read “Examination of urine for microhematuria.”

“POC-CCA test is carried out according to manufacturer’s instructions (Rapid Medical Diagnostics, Noordhoek, South Africa). POC-CCA test is carried out one drop of urine is added to the well of the cassette and after being fully absorbed, one drop of buffer is added to the same well. The result is read after 20 minutes of incubation, interpretation of results as followed: negative (only positive control line), trace result line extremely weak, +1 (result line easy to see but still weak), +2 (result line same as the control) and +3 (result line darker than the control line).”

The paragraph above needs revision.

**Results**

There is a great disparity between the samples analyzed during the baseline study, in year 1 and year 2 respectively? Why is this so? Why are there only 501 samples analyzed in year 1?

The information in table 1 should be represented graphically

**Discussion**

The discussion section also needs a careful revision. The data generated from the point-of-care urine circulating cathodic antigen (POC-CCA) assay was not presented in the results section but was mentioned briefly in the discussion section. Why is this so?

**Conclusion**

The overall conclusion from the study does not add anything new to what is already known in the field although the authors reported that the use of the reagent dipstick test is not frequently used in the area of study. Also, the conclusion does not mention the purpose of the study being detection of light infection. In view of the review provided here, I recommend a major revision of this study.
