# Supplementary material for: Comparing the accuracy of two diagnostic methods for detection of light Schistosoma haematobium infection in an elimination setting in Wolaita Zone, South Western Ethiopia
Source: PLoS One. 2022 Apr 29;17(4):e0267378. doi: 10.1371/journal.pone.0267378 (PMC9053789; doi:10.1371/journal.pone.0267378)
Supplement: Attachment — Submitted filename: Second Reviewer comment to Author 02032022.docx [file pone.0267378.s006.docx]

**Review of manuscript:** Comparing the diagnostic accuracy for detection of light *Schistosoma haematobium* infection in an elimination setting in Wolaita Zone, South Western Ethiopia

**Reviewer’s Comments to Author**

**Comments to the Author**

The manuscript proposed by Mohammed et al. is titled: Comparing the diagnostic accuracy for detection of light *Schistosoma haematobium* infection in an elimination setting in Wolaita Zone, South Western Ethiopia

The study described in this manuscript was conducted in the Wolaita Zone, South Western Ethiopia and it provides information on the comparative analyses of two schistosomiasis diagnostic techniques – Urinalysis dipstick and urine filtration. The authors attempted to shed a new light on the use of these diagnostic techniques in order to fill gaps in our knowledge of schistosomiasis control. The study was conducted between 2018 and 2020 and a total of 16,780 children and adults participated in the study. A total of 12,102 samples were examined in the baseline study using dipstick and urine filtration; among them, 285 (2.4%) samples were positive for microhematuria and 21 (0.2%) positive for *S. haematobium* eggs by urine filtration. 501 urine samples were examined in 2019 and 10 (2.0%) were positive for microhematuria, and all of them were negative for *S. haematobium* infection. A total of 3875 individuals were examined in 2020 and 162 (4.2%) were positive for microhematuria and 2 (0.05%) were positive for *S. haematobium* eggs. The prevalence of microhematuria infection based on reagent dipstick was comparable between males (50.9%) and females (49.1%), and across the different age groups Pre-SAC (17.1%), SAC (25.0%), 15 to 20 (20.4%), 21 to 35 (20.2%) and 36+(17.3%) years. The prevalence of microhematuria was highest in SAC (4-15 years age) compared to other age groups. The sensitivity and specificity of reagent strip was 100% (95% CI:85.18%–100.00%) and 97.4% (95% CI: 97.10%-97.60%), respectively. The PPV was low 5.03% and NPV was high 100%.

Most of the concerns addressed in my initial review have been addressed.

**Abstract**

The calculations are still not correct. 285/16,780 is not equal to 2.4% (285/12,102 = 2.4%). Also, 172/16,654 is not equal to 6.2%. The calculations need to be checked again.

**Materials and Methods
Sample size calculations**
“The sample size was estimated using at least 70% probability of woredas with true prevalence of schistosomiasis of 15% being classified as 10% or over, under the assumption that the lower

focality of STH was led to sufficient sample sizes for schistosomiasis between 26% and 75% of

kebeles were surveyed within each woreda [21], with a higher proportion of kebeles being

surveyed in small woreda.”

The sentence above has still not been revised for better comprehension.

**Results**

There is a great disparity between the samples analyzed during the baseline study, in year 1 and year 2 respectively? Why is this so? Why are there only 501 samples analyzed in year 1?

The information in table 1 should be represented graphically

**Discussion**

The discussion section still needs a careful revision to correct typographical errors. The sentence “Using kappa value evaluation between the two tests was revealed slight agreement” should be corrected.

**Conclusion**

It is still difficult to understand the conclusion from the study based on the wording of the sentences. The authors need to compose the information in a way that is clear and understandable.

The manuscript as revised is an improvement on the previous version but it still requires further revision.
